# [2 + 2 + 1] Cycloaddition of *N*-tosylhydrazones, *tert*-butyl nitrite and alkenes: a general and practical access to isoxazolines†

**DOI:** 10.1039/d1sc02352g

**Published:** 2021-06-22

**Authors:** Liang Ma, Feng Jin, Xionglve Cheng, Suyan Tao, Gangzhong Jiang, Xingxing Li, Jinwei Yang, Xiaoguang Bao, Xiaobing Wan

**Affiliations:** Key Laboratory of Organic Synthesis of Jiangsu Province, College of Chemistry, Chemical Engineering and Materials Science, Soochow University Suzhou 215123 China xgbao@suda.edu.cn wanxb@suda.edu.cn

## Abstract

*N*-Tosylhydrazones have proven to be versatile synthons over the past several decades. However, to our knowledge, the construction of isoxazolines based on *N*-tosylhydrazones has not been examined. Herein, we report the first demonstrations of [2 + 2 + 1] cycloaddition reactions that allow the facile synthesis of isoxazolines, employing *N*-tosylhydrazones, *tert*-butyl nitrite (TBN) and alkenes as reactants. This process represents a new type of cycloaddition reaction with a distinct mechanism that does not involve the participation of nitrile oxides. This approach is both general and practical and exhibits a wide substrate scope, nearly universal functional group compatibility, tolerance of moisture and air, the potential for functionalization of complex bioactive molecules and is readily scaled up. Both control experiments and theoretical calculations indicate that this transformation proceeds *via* the *in situ* generation of a nitronate from the coupling of *N*-tosylhydrazone and TBN, followed by cycloaddition with an alkene and subsequent elimination of a *tert*-butyloxy group to give the desired isoxazoline.

## Abstract 2

A novel [2 + 2 + 1] cycloaddition of *N*-tosylhydrazones, *tert*-butyl nitrite and alkenes was successfully established, which allowed facile construction of a wide range of isoxazolines.

## Graphical abstract

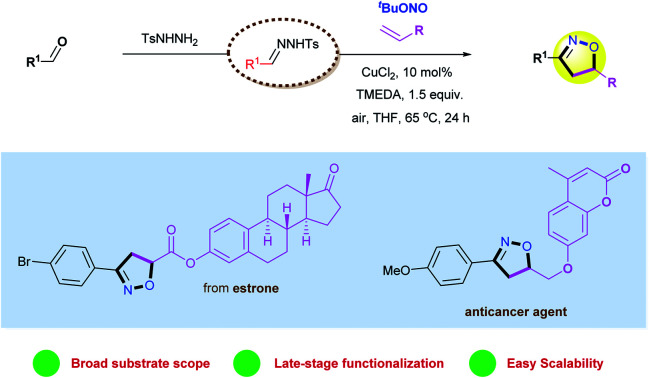

## Introduction

The electrophilicity of the carbonyl carbon and the acidity of the α-hydrogen in ketones and aldehydes allow these compounds to play important roles in synthetic chemistry.^1^ Over the past several decades, *N*-tosylhydrazones, which are readily obtained from ketones or aldehydes, have rapidly become a new type of versatile synthon for transition metal-catalysed or metal-free reactions.^2^ Since the pioneering work by Barluenga,^3*m*–3*p*^ a wide range of transformations using *N*-tosylhydrazones has been established, including coupling,^3^ insertion,^4^ cyclization,^2*e*,5^ olefination^6^ and alkynylation^7^ reactions, and these are now standard methods in organic synthesis. Although this unconventional manipulation of carbonyl compounds provides powerful and indispensable protocols for the facile construction of diverse molecular frameworks, this rapidly evolving area is still far from mature and there is ample opportunity for further development. As an example, the use of *N*-tosylhydrazones in the synthesis of isoxazolines has not been explored to date.

Isoxazolines^8^ are important structural motifs that are ubiquitous in bioactive molecules, pharmaceuticals and chiral ligands,^9^ and are also versatile intermediates^10^ in organic synthesis. Thus, the development of efficient approaches to the construction of these valuable molecules has received increasing attention. State-of-the-art synthetic methods have shown that the formation of isoxazolines proceeds primarily *via* the [3 + 2] cycloaddition of olefins with nitrile oxides generated *in situ*. However, the formation of furoxan (a nitrile oxide dimer) is an undesired side reaction that is difficult to avoid during such syntheses. The nitrile oxides that are employed in these procedures are typically derived from aldoximes^11^ or nitro compounds,^12^ although the use of various additives (including oxidants, bases, Grignard reagents and dehydrating agents) can cause environmental, cost and safety issues, especially when employed on an industrial scale. Consequently, alternative synthetic methods have been examined, such as the intramolecular cyclization of β,γ-unsaturated oximes,^13^ the iminoxyl radical promoted formation of isoxazolines^14^ and others.^15^ Despite the significant achievements to date, the methods that have been reported often have limited substrate scopes, especially with regard to the range of possible functional groups. Thus, the development of new and versatile techniques for the synthesis of isoxazolines remains challenging.

**Scheme 1:**
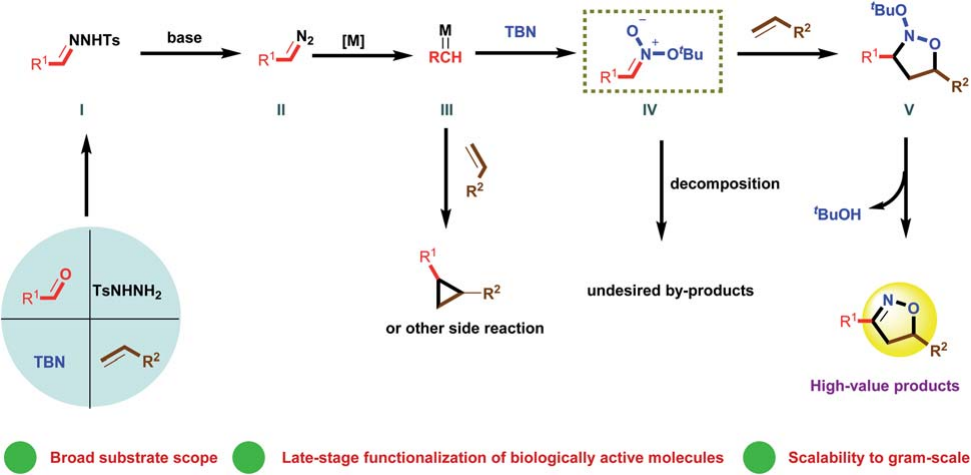
Reaction design: [2 + 2 + 1] cycloaddition to construct isoxazoline based upon nitronates.

Recently, our group demonstrated the synthesis of isoxazolines from diazo compounds bearing electron withdrawing groups.^15*a*,15*d*^ Unfortunately, these diazo compounds have to be pre-synthesized using a process comprising several time-consuming steps. More importantly, diazo compounds without electron withdrawing substituents are usually unstable and difficult to handle, which limits their synthetic value when constructing complex structures. Consequently, the present work employed *N*-tosylhydrazones as more stable surrogates for unstable diazo compounds. These compounds were generated *in situ* from readily obtainable commercial aldehydes and TsNHNH_2_, thus providing a new platform for the construction of isoxazolines. To illustrate the feasibility of this new approach, we also propose a plausible mechanism herein, as shown in Scheme 1. In this mechanism, the condensation of aldehydes with TsNHNH_2_ generates *N*-tosylhydrazones **I**, which are easily transformed to the diazo compounds **II** by adding a suitable base. The loss of a diatomic nitrogen molecule from the diazo in the presence of a transition metal catalyst affords the carbene **III**. Subsequently, **III** reacts with *tert*-butyl nitrite (TBN) to generate the nitronate **IV**, which then undergoes a [3 + 2] cycloaddition with an alkene to afford the cycloadduct **V**. Finally, the elimination of ^*t*^BuOH delivers the desired isoxazoline. However, it is very challenging to realize the [2 + 2 + 1] cycloaddition step in this process because the mechanism involves several highly reactive intermediates that tend to undergo undesired side reactions. As an example, the cyclopropanation reaction^2*e*,5*h*^ between a carbene and an alkene is a well-known process that can potentially interrupt the formation of the unstable nitronate. Typically, an electron withdrawing group must be attached to the α-carbon to both form and stabilize **IV**.^16^ Thus, a lack of suitable electron-withdrawing groups may not permit the successful *in situ* generation of **IV** in this transformation. Moreover, this nitronate is prone to decomposition instead of the desired cycloaddition with an alkene to deliver the cycloadduct **V**.

Herein, we report the first-ever construction of isoxazolines from *N*-tosylhydrazones. The most important features of this methodology are the ability to use readily-available aldehydes and alkenes, a wide substrate scope, significant functional group compatibility, the potential for the late-stage modification of bioactive molecules and scalability to the 20 mmol level. This new approach to isoxazolines formation proceeds through a pathway presumably involving unstable nitronates, in sharp contrast to the classical [3 + 2] cycloaddition of nitrile oxides with alkenes.

## Results and discussion

**Scheme 2:**
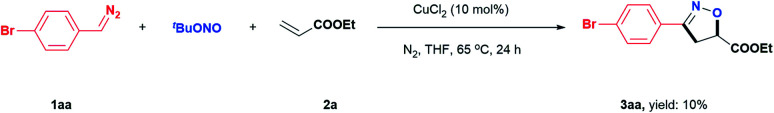
Initial attempt for the synthesis of isoxazoline.

In an initial study, we examined the reaction of the diazo compound **1aa** with TBN and ethyl acrylate, **2a**, catalysed by CuCl_2_. The desired isoxazoline **3aa** was obtained in 10% yield (Scheme 2). Although the yield was very low, the above results demonstrated the feasibility of our design. The efficiency of this isoxazoline formation reaction was enhanced by developing a one-pot, two-step procedure using commercially available aldehydes as starting materials. After exploring a wide range of reaction parameters, we determined that the use of CuCl_2_ (10 mol%) and *N*,*N*,*N*,*N*,-tetramethylethylenediamine (TMEDA, 1.5 equiv.) gave **3aa** in an 88% yield (Table 1, entry 1). It is important to note that this [2 + 2 + 1] cycloaddition reaction was able to tolerate both air and moisture. In addition, **3aa** could still be obtained in a good yield even in the absence of CuCl_2_ (entry 2). In sharp contrast, a control experiment showed that TMEDA was indispensable to achieving optimal reaction efficiency (entry 3). Similar results were also observed when the reaction was performed using a variety of nitrites or different copper catalysts (entries 10–16). Other bases, including representative inorganic and organic bases, were less effective than TMEDA (entries 17–24), although the atmosphere under which the reaction was performed had little effect on efficiency (entries 25 and 26). When the reaction was performed at 80 °C (entry 27), the yield was nearly identical to that obtained at 65 °C, while very little **3aa** was produced at room temperature (entry 28).

**Table 1:** Synthesis of isoxazoline: effect of reaction parameters^a^

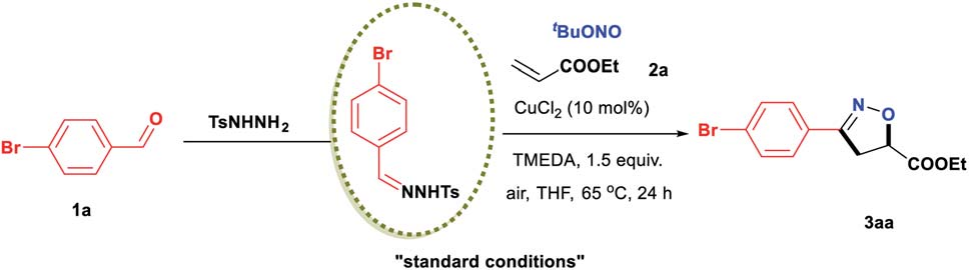		
Entry	Variation from the “standard conditions” catalyst	Yield^b^ (%)
1	None	88
2	No CuCl_2_	61
3	No TMEDA	<1
4	Acetone instead of THF	84
5	MeCN instead of THF	54
6	EA instead of THF	31
7	Toluene instead of THF	53
8	CHCl_3_ instead of THF	81
9	DMF instead of THF	72
10	^*i*^PrONO instead of ^*t*^BuONO	79
11	^*n*^BuONO instead of ^*t*^BuONO	68
12	^*i*^BuONO instead of ^*t*^BuONO	76
13	CuCl instead of CuCl_2_	77
14	CuBr instead of CuCl_2_	83
15	CuI instead of CuCl_2_	56
16	Cu(OAc)_2_ instead of CuCl_2_	58
17	Cs_2_CO_3_ instead of TMEDA	<1
18	DABCO instead of TMEDA	68
19	^*t*^BuOLi instead of TMEDA	<1
20	K_2_CO_3_ instead of TMEDA	<1
21	^*t*^BuOK instead of TMEDA	<1
22	NaOH instead of TMEDA	<1
23	K_3_PO_4_ instead of TMEDA	<1
24	Na_2_CO_3_ instead of TMEDA	16
25	N_2_ instead of air	76
26	O_2_ instead of air	84
27	At 80 °C	87
28	At room temperature	Trace

a Reaction conditions: *N*-tosylhydrazones (0.65 mmol, generated *in situ* from 4-bromobenzaldehyde **1a** and TsNHNH_2_), ethyl acrylate **2a** (0.50 mmol), CuCl_2_ (10 mol%), TMEDA (0.75 mmol), and TBN (2.0 mmol) in 4.0 mL THF at 65 °C for 24 h.

b Isolated yields of the average of two experiments.

With the optimal catalyst and reaction conditions identified, we next assessed the scope of aldehyde that could be used in this process (Table 2). Numerous mono-substituted benzaldehydes containing both electron-donating and electron-withdrawing groups were all found to be viable substrates, delivering the desired products in moderate to excellent yields. The exact structure of **3ab** was confirmed by single crystal X-ray diffraction (for details, see the ESI†). Of note, the position of the substituent (*para*-, *meta*- or *ortho*-) had no significant effect on the [2 + 2 + 1] cycloaddition reaction. This method also showed broad compatibility with common functional groups, including alkyl (**3ac**, **3at**), halide (**3af**, **3ag**, **3ar**, **3as**, **3ax**, **3ay**, **3ha**), ether (**3ad**, **3au**, **3aw**, **3ba**), amide (**3an**), cyano (**3ak**), nitro (**3al**, **3ap**), sulfonyl (**3aj**) and trifluoromethyl (**3ah**, **3aq**) groups. Remarkably, aldehydes with more reactive functional groups such as hydroxyl (**3ao**, **3av**, **3aw**, **3ay**), thioether (**3ae**), amine (**3ai**) and carboxyl (**3am**) groups, which are typically challenging to use as substrates in known methods, reacted smoothly with TBN and **2a** to provide the corresponding products in satisfactory yields. Naphthyl-substituted aldehydes were also readily transformed into the desired products in high yields (**3az**, **3da**). In addition, polysubstituted aldehydes were accommodated under the optimal conditions, giving the corresponding products in good yields (**3ax**, **3ay**, **3ba**, **3ga**, **3ha**). The example of **3fa** is especially noteworthy, because the double bond in the alkyl chain was retained and therefore could be utilized for further modifications. Biologically active heterocycles, including benzo[*b*]thiophene (**3ca**), chromone (**3ea**), antipyrine (**3ga**) and pyrazole (**3ha**), could be converted into the corresponding isoxazoline products in good yields. Cyclopropanecarboxaldehyde also delivered the desired product **3ia** in moderate yield.

**Table 2:** Evaluation of aldehydes^a^

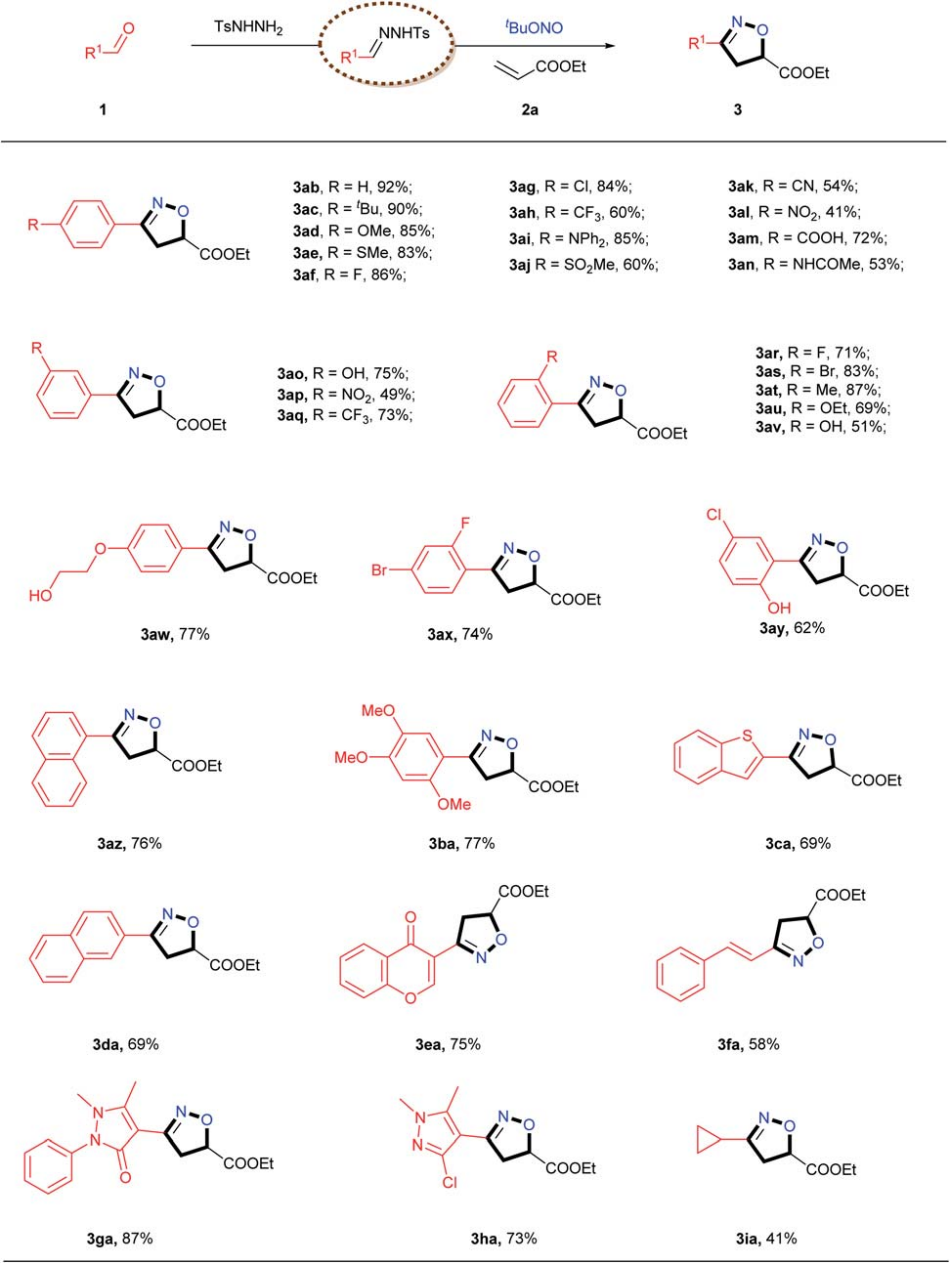

a Reaction conditions: *N*-tosylhydrazones (0.65 mmol, generated *in situ* from aldehyde **1** and TsNHNH_2_), ethyl acrylate **2a** (0.50 mmol), CuCl_2_ (10 mol%), TMEDA (0.75 mmol), and TBN (2.0 mmol) in 4.0 mL THF at 65 °C for 24 h under air atmosphere. For details, see the ESI.

Subsequently, the scope of alkenes was evaluated, as illustrated in Table 3. Not surprisingly, a variety of acrylates reacted smoothly to afford the desired products in good yields. A wide range of synthetically versatile functional groups, including ester (**4aa–4ae**, **4aj**, **4ja**), trifluoromethyl (**4ae**) and alkyne (**4aj**) moieties, were accommodated under the present conditions. For the reaction of acrylamide, including free amine (**4af**), primary amine (**4ah**) and secondary amine (**4ag** and **4ai**), the target products were obtained in good yields. Interestingly, the use of a triene also gave the corresponding isoxazoline **4ja**, albeit in a relatively low yield, and pentafluorostyrene delivered the desired product **4as** in a reasonable yield under the standard conditions. This [2 + 2 + 1] cycloaddition reaction was also found to be compatible with unactivated aliphatic alkenes (**4aw**, **4ay**, **4ga**, **4ka**, **4ma–4oa**). In addition, styrene derivatives carrying both electron-donating and electron-withdrawing groups afforded the corresponding isoxazoline products (**4ak–4ar**) in acceptable yields. Under the identical conditions, the reaction with diethyl vinylphosphonate resulted in the desired product **4ea** in a good yield. Silylated alkenes, including vinyltrimethylsilane (**4ca**) and vinylphenyldimethylsilane (**4av**), were also viable substrates for this transformation. Notably, even enamides (**4au**), enol esters (**4la**) and enol ethers (**4az**) were smoothly transformed into the corresponding products in synthetically useful yields. In addition, both internal alkenes (**4ga**) and 1,1-disubstitued alkenes (**4at**, **4ha**, **4ma**) also underwent smooth cyclization with satisfactory yields, and two spiro products (**4at** and **4ha**) were obtained in good yields. Biologically active heterocycles, including saccharin (**4ka**) and phthalimide (**4na**), remained intact during the [2 + 2 + 1] cycloaddition reaction. It is particularly noteworthy that this protocol could generate a wide range of isoxazolines, including those bearing less stable functional groups such as hydroxyl (**4ah**, **4aw**), NHBoc (**4ay**), free N–H (**4af**, **4ah**), thioether (**4da**) and acetal (**4fa**) moieties, which are typically challenging substrates in traditional nitrile oxide cycloadditions. The retention of these versatile functional groups provides the option for further modification of the corresponding isoxazolines products.

**Table 3:** Evaluation of alkenes^a^

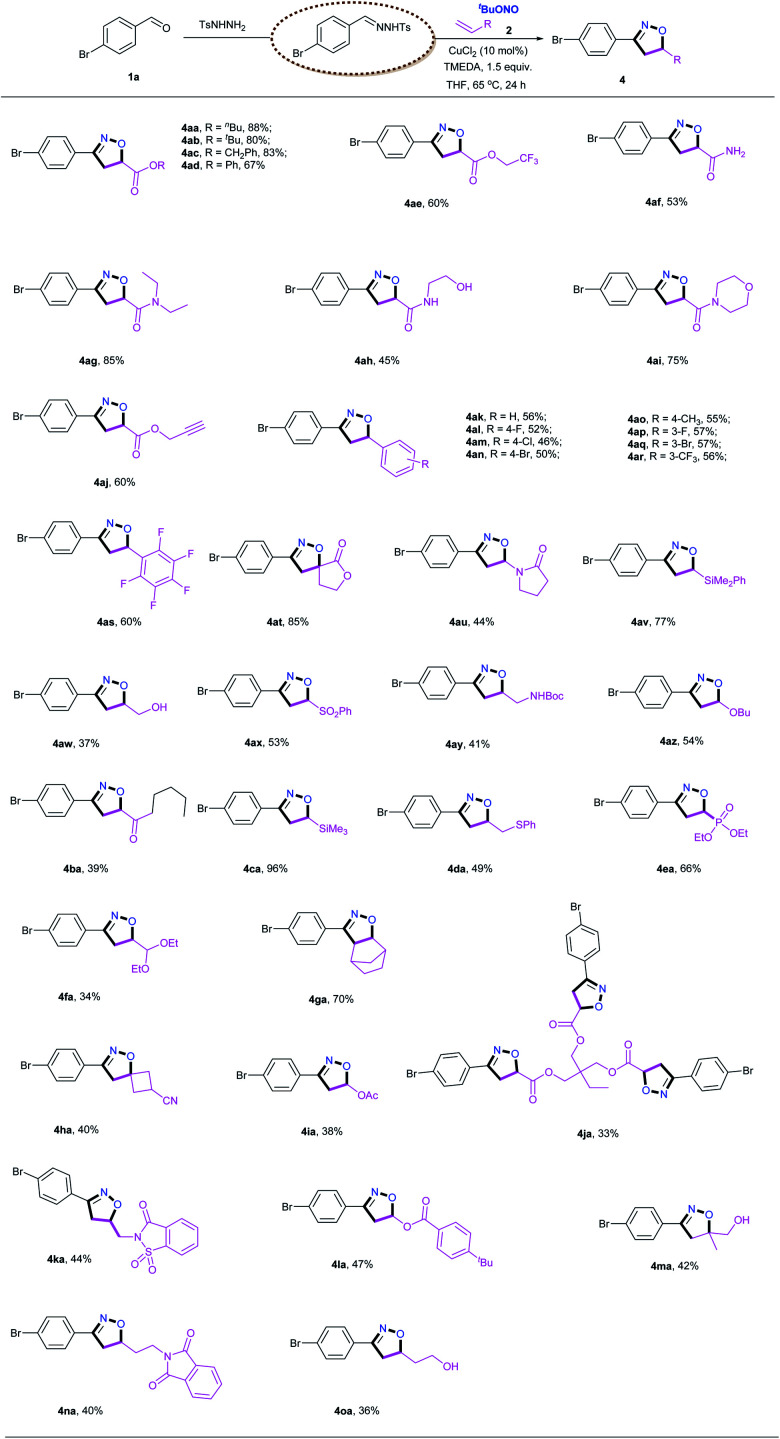

a Reaction conditions: *N*-tosylhydrazones (0.65 mmol, generated *in situ* from 4-bromobenzaldehyde **1a** and TsNHNH_2_), alkenes **2** (0.50 mmol), CuCl_2_ (10 mol%), TMEDA (0.75 mmol), and TBN (2.0 mmol) in 4.0 mL THF at 65 °C for 24 h under air atmosphere.

The excellent functional group tolerance exhibited by this process prompted us to apply this methodology to bioactive and pharmaceutical molecules (Table 4). The reactions of *N*-tosylhydrazones with suitable alkenes provided drug candidates such as carbonic anhydrase inhibitors (**5**),^8*a*^ anticancer agents (**6**)^8*c*^ and antibacterial agents (**8**)^8*g*^ in one-pot processes with moderate yields. Furthermore, various complex bioactive molecules (indomethacin **7**, naproxen **9**, pregnenolone **10**, estrone **11**, ezetemibe **12**) reacted with the *N*-tosylhydrazone obtained from 4-bromobenzaldehyde, **1a**, under the established conditions, and were transformed into the corresponding isoxazolines in moderate to good yields. These examples highlight the wide applicability of this new method, and we therefore envision that this protocol could have potential applications in drug development.

**Table 4:** Synthetic application for biologically important derivatives^a^

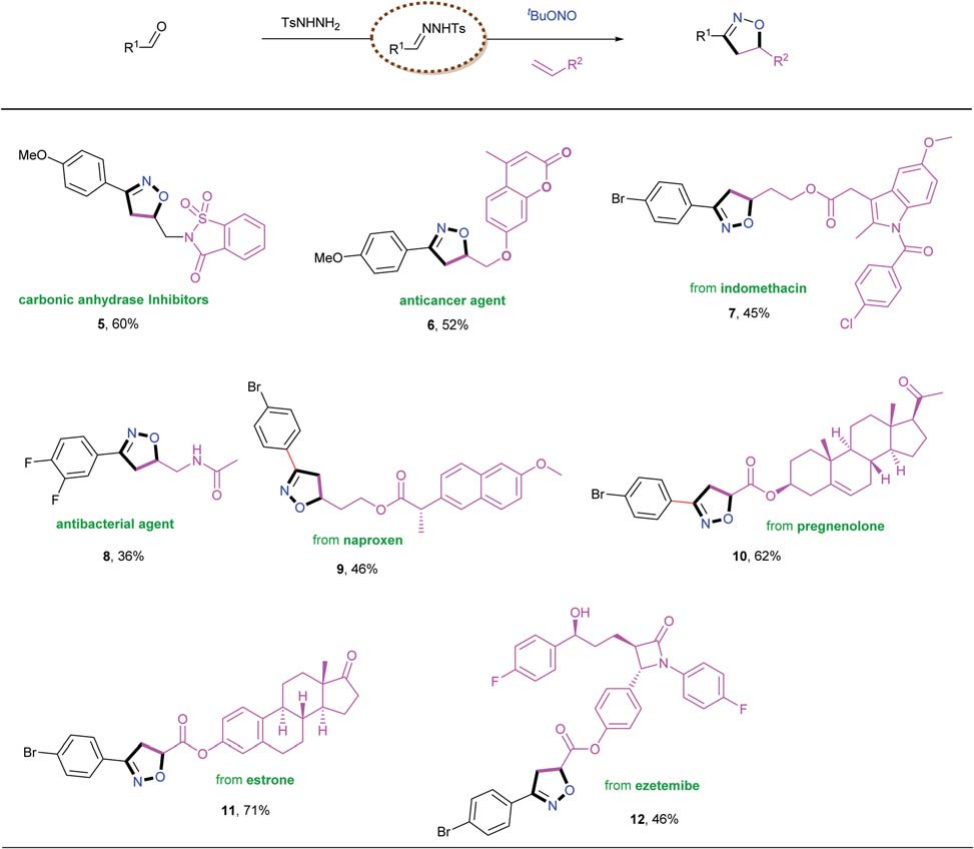

a Reaction conditions: *N*-tosylhydrazones (0.65 mmol, generated *in situ* from aldehydes **1** and TsNHNH_2_), alkenes **2** (0.50 mmol), CuCl_2_ (10 mol%), TMEDA (0.75 mmol), and TBN (2.0 mmol) in 4.0 mL THF at 65 °C for 24 h under air atmosphere.

**Scheme 3:**
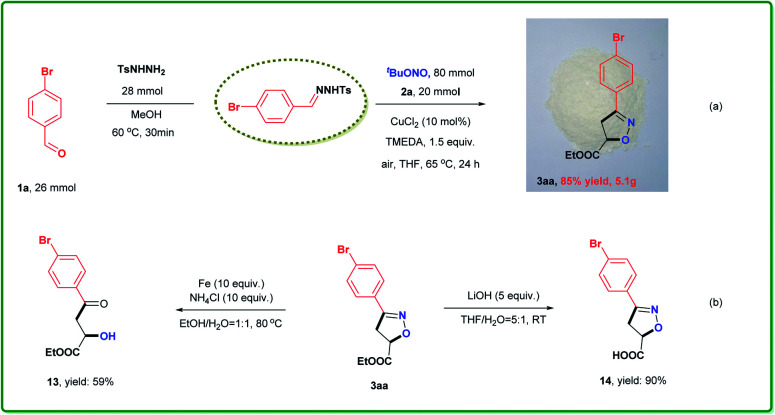
Scale-up experiment and further transformations.

Another advantage of our cycloaddition approach is ready scalability, as shown in Scheme 3. The reaction of 26 mmol **1a**, 28 mmol TsNHNH_2_, 20 mmol **2a** and 80 mmol TBN with 10 mol% CuCl_2_ at 65 °C for 24 h, followed by filtration through a pad of silica gel, afforded **3aa** in 85% yield (Scheme 3a). Thus, there was no significant loss in efficiency compared with the reaction on the 0.5 mmol scale. We also conducted further transformations of the isoxazoline as shown in Scheme 3b. Both reductive cleavage (**13**) and hydrolysis (**14**) proceeded smoothly to give the corresponding high-value derivatives in good yields.

**Scheme 4:**
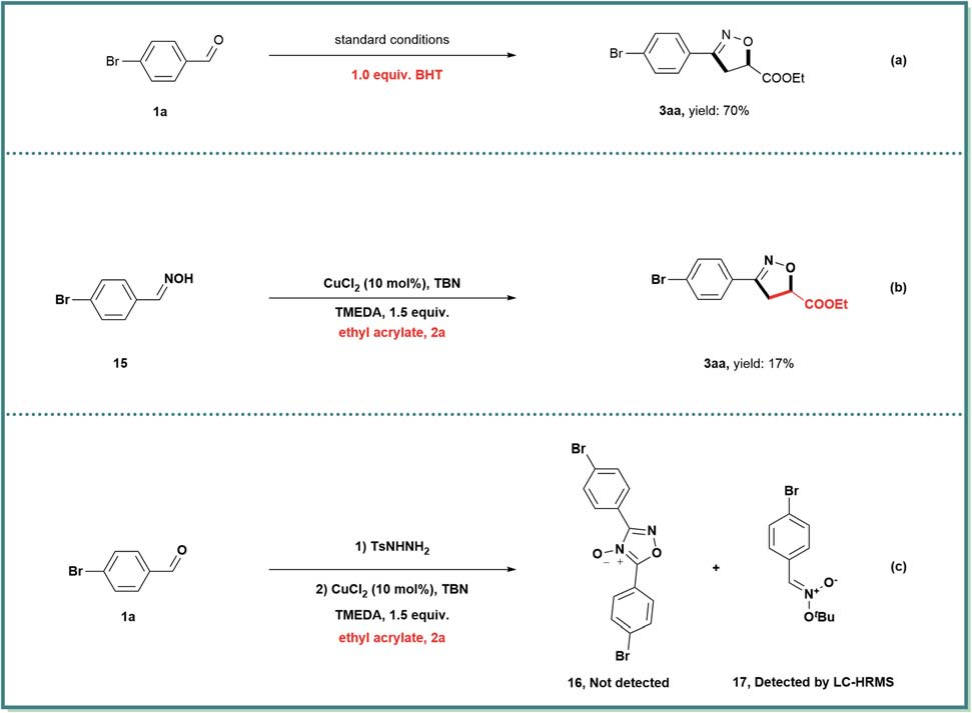
Probe for the possible mechanism.

A variety of control experiments were performed to gain preliminary insights into the possible mechanistic pathways (Scheme 4). The addition of butylated hydroxytoluene (BHT) only minimally inhibited the formation of isoxazoline **3aa**, indicating that radical intermediates were not involved in this transformation (Scheme 4a). When oxime (**15**), a precursor of nitrile oxide, was subjected to this cycloaddition reaction, only a small amount of **3aa** was obtained (Scheme 4b). In addition, furoxan **16** in Scheme 4c was not detected during the reaction. Because the proposed nitronate intermediate did not incorporate suitable electron-withdrawing groups, and was therefore not sufficiently stable, it could not be isolated. Fortunately, a trace amount of nitronate **17** was detected after careful analysis using liquid chromatography-high resolution mass spectrometry (for details, see the ESI†). Together, these data indicate that nitronates rather than nitrile oxides were involved in the isoxazoline formation reaction.

**Fig. 1:**
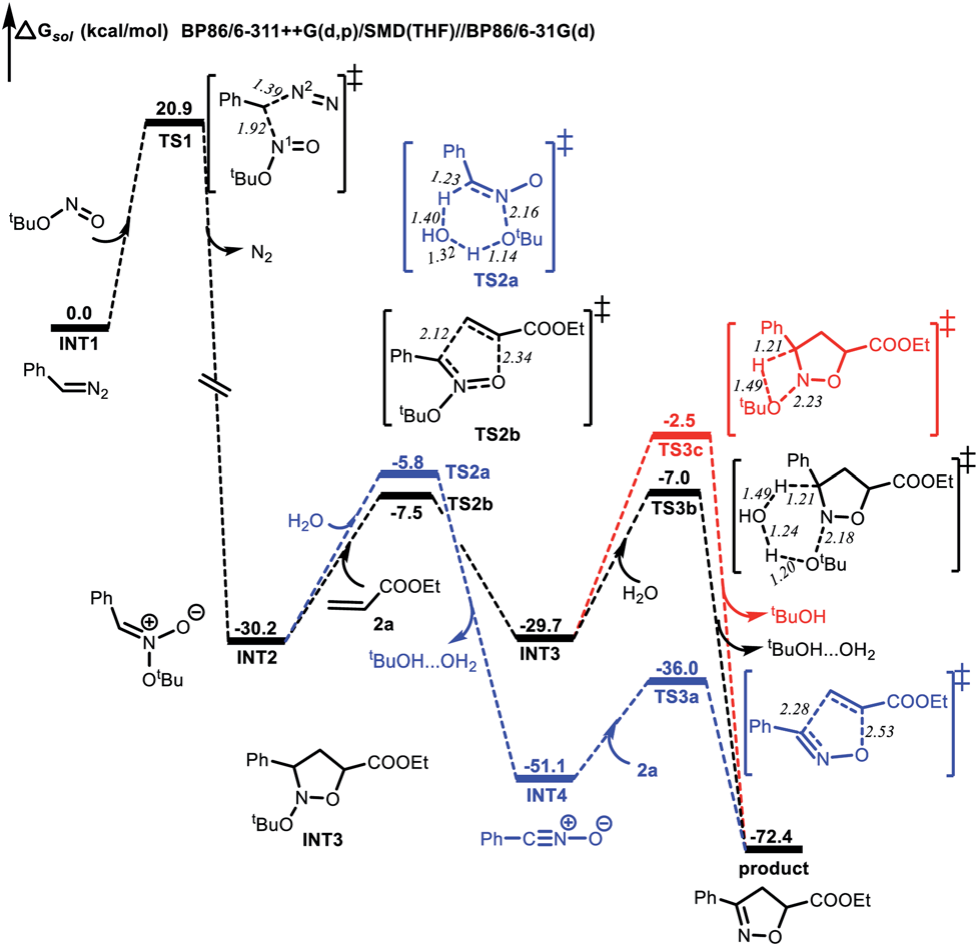
Energy profiles (in kcal mol^−1^) for the formation of isoxazolines in the absence of the CuCl_2_ catalyst. Bond lengths are shown in Å.

**Fig. 2:**
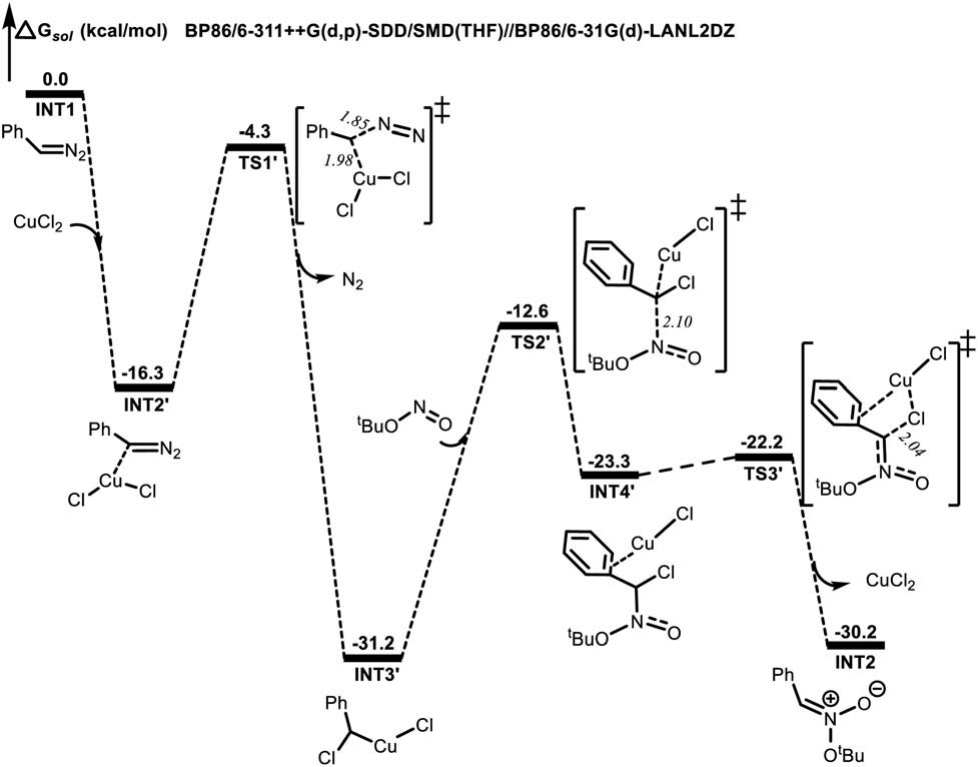
Energy profile (in kcal mol^−1^) for the formation of nitronate with the CuCl_2_ catalyst. Bond lengths are shown in Å.

Computational studies were also performed to gain further insight into the isoxazoline formation mechanism. The results showed that, in the absence of CuCl_2_ as a catalyst, the nucleophilic substitution reaction between a diazo compound (**INT1**) generated *in situ* from the *N*-tosylhydrazone and TBN produces transition state **TS1**. Here, the C^1^⋯N^1^ distance is shortened to 1.92 Å, while the C^1^⋯N^2^ distance is lengthened to 1.39 Å (Fig. 1). Subsequently, a nitronate (**INT2**) is afforded along with the release of N_2_. It is noteworthy that, in the presence of the copper catalyst, **INT1** might coordinate with the CuCl_2_ to form complex **INT2′**. This species can subsequently release N_2_*via***TS1′** to generate intermediate **INT3′** exergonically (Fig. 2). Following this, the nucleophilic attack of TBN on **INT3′***via***TS2′** could afford intermediate **INT4′**, after which a nitronate (**INT2**) is generated *via***TS3′** along with the regeneration of CuCl_2_. Similarly, employing CuCl as the catalyst could also promote the formation of nitronate **INT2***via* analogous steps (Fig. S3 in the ESI†). This nitronate could then undergo an intermolecular [3 + 2] cycloaddition with an alkene (**2a**) to produce a cyclized intermediate. Both regioselective cycloaddition pathways were considered, and the corresponding transition states were determined to be **TS2b** (Fig. 1) and **TS2c** (Fig. S2 in the ESI†), respectively. In the case of **TS2b**, the C^1^⋯C^2^ and O^1^⋯C^3^ distances are shortened to 2.12 and 2.34 Å, respectively. Thus, the five-membered ring intermediate **INT3** is generated in a concerted manner. The computational results also suggested that the energy barrier to the formation of **INT3***via***TS2b** is 1.6 kcal mol^−1^ lower than that associated with the intermolecular [3 + 2] cycloaddition *via***TS2c**. Thus, the formation of **INT3***via***TS2b** should occur more readily. Finally, the elimination of ^*t*^BuOH *via***TS3c** would be expected to give the desired product. This process could be assisted by a water molecule acting as a shuttle to transfer a proton *via***TS3b** (Fig. 1). The possible elimination of ^*t*^BuOH from nitronate **INT2** to afford a nitrile oxide intermediate (**INT4**) was also considered. When a water molecule is present to assist the process, the corresponding TS is **TS2a**, and the activation energy calculated for this step is 1.7 kcal mol^−1^ higher than that for the intermolecular [3 + 2] cycloaddition *via***TS2b**. Thus, the formation of the nitrile oxide intermediate is not suggested in this reaction, although the subsequent intermolecular [3 + 2] cycloaddition with **2a***via***TS3a** should proceed to give the desired product. At present, we suggest that the product is most likely obtained through intermediate **INT3** followed by the elimination of ^*t*^BuOH.

**Scheme 5:**
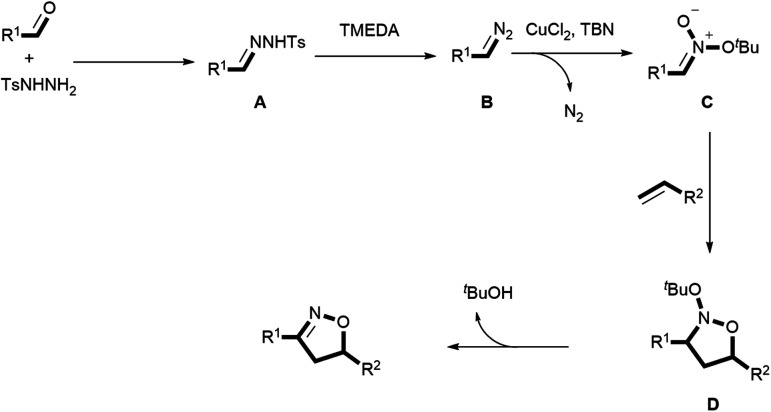
Proposed catalytic cycle.

Based on both our experimental results and density functional theory (DFT) calculations, a plausible catalytic cycle was proposed, as shown in Scheme 5. Initially, a condensation reaction between the aldehyde and the TsNHNH_2_ generates the *N*-tosylhydrazone **A**. **A** then produces the diazo compound **B** in the presence of TMEDA, which is subsequently captured by TBN to form the nitronate **C**, aided by CuCl_2_. Next, the [3 + 2] cycloaddition of **C** with the alkene gives rise to the nitroso acetal **D**.^15*a*,15*e*,17^ Finally, the release of a *tert*-butyloxy group from **D** delivers the desired isoxazoline.

## Conclusions

A novel CuCl_2_-catalysed [2 + 2 + 1] cycloaddition strategy based on the reaction of *N*-tosylhydrazones, TBN and alkenes has been developed and characterized. This modular protocol offers a practical and flexible approach to the synthesis of isoxazolines, and a vast array of aldehydes and alkenes have been found to be suitable substrates. Thus, this method represents a highly valuable complement to classical nitrile oxide-based cycloaddition. We believe that this technique may have potential uses in drug discovery because the synthesis is both facile and compatible with a wide range of functional groups. DFT calculations and experimental work provided preliminary mechanistic insights, and the proposed mechanism evidently involves the generation of nitronates and subsequent cycloaddition with alkenes. Further, more detailed investigations of the reaction mechanism and synthetic applications are in progress in our laboratory.

## Data availability

All data is in the ESI.† There is no more to deposit.

## Author contributions

L. M. performed the experiments and prepared the ESI.† X. C., S. T., G. J., X. L. and J. Y. prepared some substrates and repeated experiments. F. J. and X. B. carried out all computational work. X. B. and X. W. conceived and directed the project and wrote the paper. All the authors discussed the results and commented on the manuscript.

## Conflicts of interest

There are no conflicts to declare.

## Supplementary Material

SC-012-D1SC02352G-s001

SC-012-D1SC02352G-s002
