# Supplementary material for: [2 + 2 + 1] Cycloaddition of N-tosylhydrazones, tert-butyl nitrite and alkenes: a general and practical access to isoxazolines
Source: Chem Sci. 2021 Jun 22;12(28):9823–30. doi: 10.1039/d1sc02352g (PMC8293996; doi:10.1039/d1sc02352g)
Supplement: SC-012-D1SC02352G-s001 [file SC-012-D1SC02352G-s001.pdf]

Electronic Supplementary Material (ESI) for Chemical Science.
This journal is © The Royal Society of Chemistry 2021

SUPPORTING INFORMATION

[2+2+1] Cycloaddition of *N*-Tosylhydrazones, *tert*-Butyl Nitrite and Alkenes: A General and Practical Access to Isoxazolines

Liang Ma, Feng Jin, Xionglve Cheng, Suyan Tao, Gangzhong Jiang, Xingxing Li, Jinwei Yang, Xiaoguang Bao,* Xiaobing Wan*

Key Laboratory of Organic Synthesis of Jiangsu Province, College of Chemistry, Chemical Engineering and Materials Science, Soochow University, Suzhou 215123, P. R. China

Email: xgbao@suda.edu.cn, wanxb@suda.edu.cn

List of Contents

General information **S2**

Fig. S1 Nitronate detected by LC-HRMS **S4**

Figure S2. Energy profile for the unfavorable cycloaddition of nitronate and **2a** **S5**

Figure S3. Energy profile (in kcal/mol) for the formation of nitronate with CuCl catalyst **S5**

X-Ray diffraction analysis of compound **3ab** **S6**

Compound characterizations **S8**

Spectroscopic data for products **S36**

Computational methods **S126**

Cartesian Coordinates and Energies **S128**

General information

Column chromatography was performed on silica gel (300-400 mesh) and reactions were monitored by thin layer chromatography (TLC) using UV light to visualize the course of the reactions. ^{1}H NMR (400 MHz), ^{13}C NMR (100 MHz) and ^{19}F NMR (376 MHz) spectra were acquired at various field strengths as indicated, and were referenced to $CHCl_3$ (7.26 and 77.0 ppm for ^{1}H and ^{13}C respectively) or DMSO (2.50 and 39.6 ppm for ^{1}H and ^{13}C respectively). The spectra were collected at 25 °C. ^{1}H NMR coupling constants are reported in Hertz and refer to apparent multiplicities and not true coupling constants. Collection parameters: Relaxation Delay- 1.0; Pulse Width- 10.0; Number of Scans- 1~16; Sweep Width- -3.84~16.19 (20.03) ppm. Data are reported as follows: chemical shift, multiplicity (s = singlet, br s = broad singlet, d = doublet, t = triplet, q = quartet, m = multiplet, dd = doublet of doublet, etc.), coupling constant and integration. All ^{13}C NMR spectra are ^{1}H decoupled. Collection parameters: Relaxation Delay- 2.0; Pulse Width- 8.8; Number of Scans- 10~100; Sweep Width- -19.47~219.45 (238.90) ppm. IR and HRMS were performed by the State-authorized Analytical Center in Soochow University.

General procedure for "one-pot" [3+2] cycloaddtion of N-tosylhydrazone, TBN and alkenes

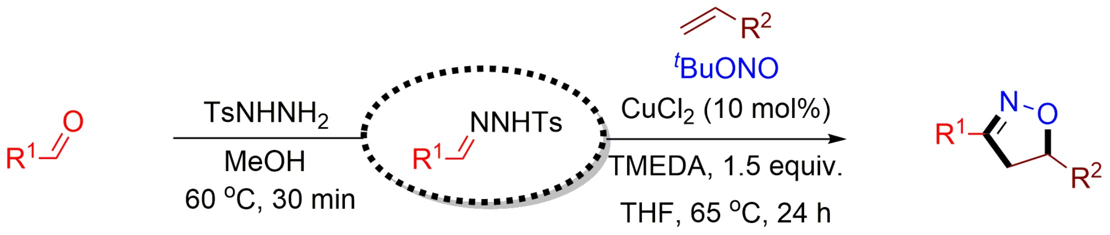

To a test tube with magnetic stirring bar, aldehyde (0.65 mmol), tosylhydrazide (0.7 mmol) and MeOH (1 mL) were added, the mixture was stirred at 60 °C for 30 minutes. After the solvent was removed under vacuum, $CuCl_2$ (0.05 mmol), THF (2.0 mL), alkenes (0.5 mmol), TMEDA (0.75 mmol), TBN (2.0 mmol) and THF (2.0 mL) were added in turn. The tube was sealed with parafilm and stirred at 65 oC for 24 h. After confirming the finish of the reaction by TLC, the resultant was concentrated in vacuum and purified by column chromatography on silica gel (ethyl acetate/petroleum ether = 1/100 to 7/1, v/v) to afford the desired products.

The procedure for the reductive cleavage of compound 3aa

To a stirred solution of ethyl 3-(4-bromophenyl)-4,5-dihydroisoxazole-5-carboxylate (**3aa**) (89.4 mg, 0.3 mmol, 1 equiv.) and NH_4Cl (161 mg, 3.0 mmol, 10 equiv.) in EtOH and Water (1:1, 15 mL) was added Fe powder (168 mg, 3.0 mmol, 10 equiv.). The mixture was stirred at 80 °C until the complete consumption of **3aa**. The reaction mixture was then cooled to room temperature, diluted with ethyl acetate, and filtered through a silica pad. The filtrate was washed with brine, and the organic layer was

separated, dried over Na_2SO_4, and evaporated in vacuo. The crude product was purified by column chromatography on silica gel with ethyl acetate/ petroleum ether to afford the product **13** as a yellow solid, 53.5 mg, 59% yield.

The procedure for the hydrolysis of compound 3aa

To a solution of LiOH (5 equiv) in water:THF (1:5, 3 mL) at room temperature was slowly added the ethyl 3-(4-bromophenyl)-4,5-dihydroisoxazole-5-carboxylate (**3aa**) (59.6 mg, 0.2 mmol, 1 equiv). The mixture was stirred at room temperature for overnight. Solvent was removed under reduced pressure. The residue was dissolved in water and acidified with 2M HCl to pH 1 followed by extraction with DCM. The crude product was purified by column chromatography on silica gel with ethyl acetate to afford the product **14** as a white solid, 48.7 mg, 90% yield.

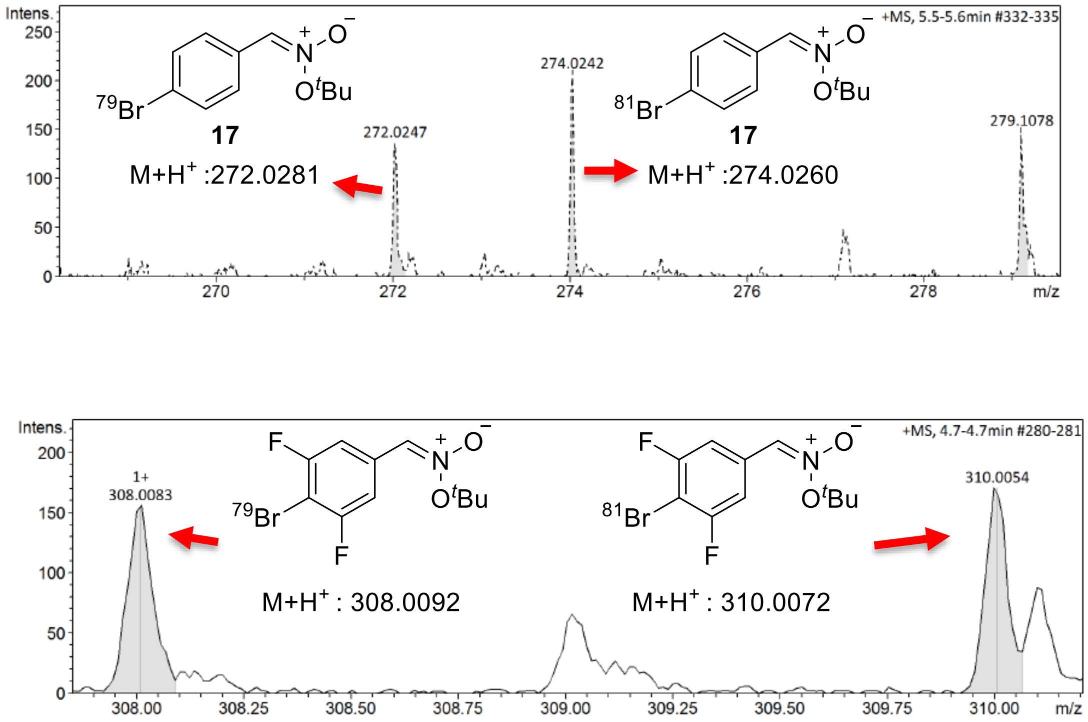

Fig. S1 Nitronate detected by LC-HRMS

DFT calculations

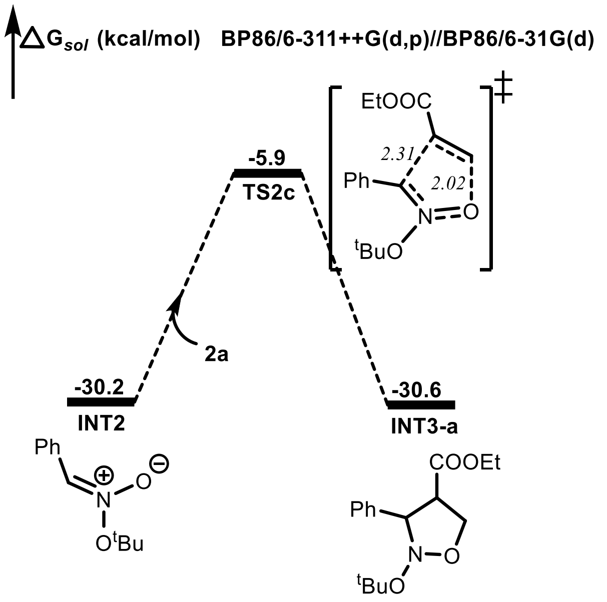

Figure S2. Energy profile (in kcal/mol) for the unfavorable cycloaddition of nitronate and **2a**. Bond lengths are shown in Å.

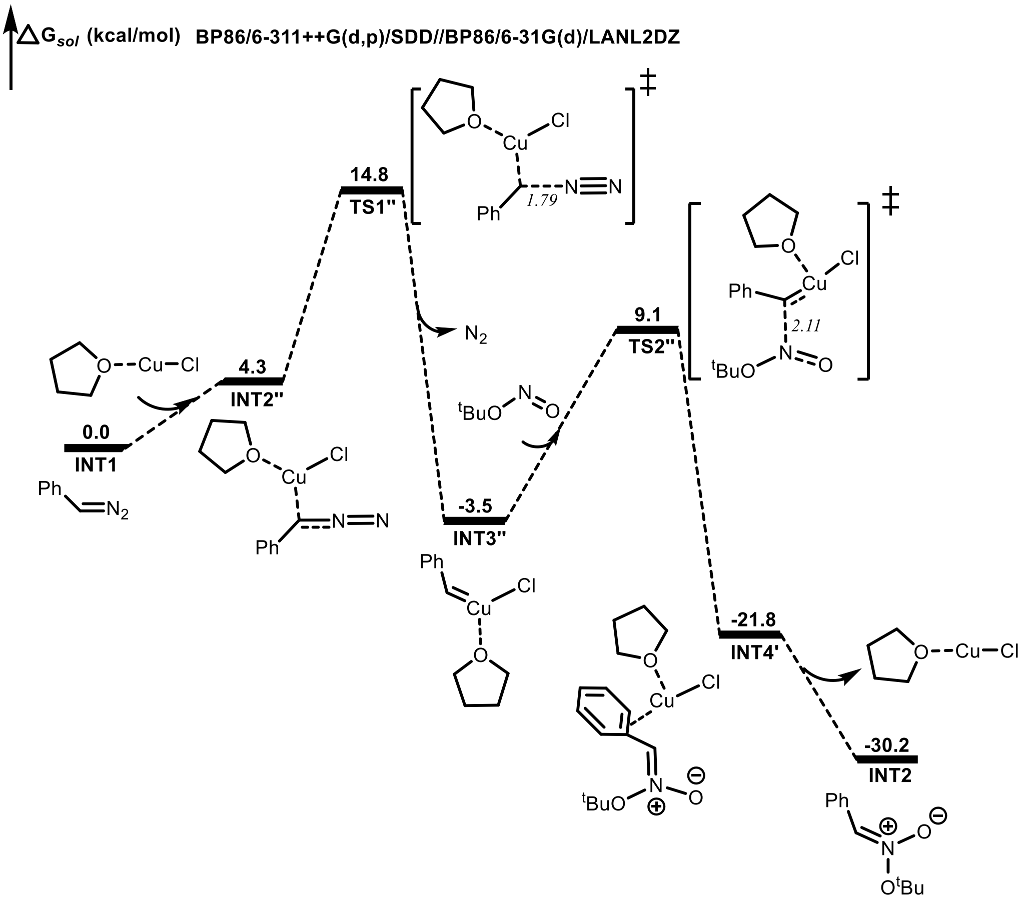

Figure S3. Energy profile (in kcal/mol) for the formation of nitronate with CuCl catalyst. Bond lengths are shown in Å.

X-Ray diffraction analysis of compound 3ab

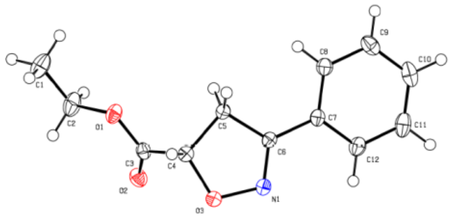

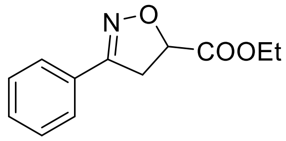

Structure of **3ab** (CCDC 2083541)

Table S1: Crystal data and structure refinement for **3ab (**Molecular structure of **3ab** with ellipsoids at the 50% probability level.**)**

Empirical formula	$C_{12}H_{13}NO_3$
Formula weight	219.23
Temperature/K	120.03
Crystal system	triclinic
Space group	P-1
a/Å	7.4807(3)
b/Å	8.6220(4)
c/Å	9.8229(4)
α/°	68.5450(10)
β/°	71.4920(10)
γ/°	84.1620(10)
Volume/Å^3	559.09(4)
Z	2
ρ_{calc}g/cm^3	1.302
μ/mm^{-1}	0.094
F(000)	232.0
Crystal size/mm^3	0.4 × 0.3 × 0.2
Radiation	MoKα (λ = 0.71073)
2Θ range for data collection/°	4.674 to 55.096
Index ranges	$-9 \le h \le 9$, $-11 \le k \le 11$, $-12 \le l \le 12$
Reflections collected	9291
Independent reflections	2472 [R_{int} = 0.0621, R_{sigma} = 0.0494]
Data/restraints/parameters	2472/0/150
Goodness-of-fit on F^2	1.048
Final R indexes [I>=2σ (I)]	R_1 = 0.0473, wR_2 = 0.1272
Final R indexes [all data]	R_1 =0.0505, wR_2 = 0.1314
Largest diff. peak/hole / e Å^{-3}	0.49/-0.23

Table S2: Bond lengths [Å] and angles [°] for **3ab**

O1-C3	1.3290(14)	C12-C11	1.3857(18)
O1-C2	1.4580(14)	C6-C5	1.5071(15)
O3-N1	1.4138(13)	C8-C9	1.3910(17)
O3-C4	1.4441(13)	C3-C4	1.5226(15)
O2-C3	1.2032(15)	C5-C4	1.5342(16)
N1-C6	1.2809(15)	C10-C9	1.387(2)
C7-C12	1.4025(16)	C10-C11	1.392(2)
C7-C6	1.4681(15)	C2-C1	1.4942(19)
C7-C8	1.3978(16)		
C3-O1-C2	116.37(9)	O1-C3-C4	108.49(9)
N1-O3-C4	109.61(8)	O2-C3-O1	126.01(11)
C6-N1-O3	109.79(9)	O2-C3-C4	125.50(11)
C12-C7-C6	121.03(11)	C6-C5-C4	100.44(9)
C8-C7-C12	118.99(11)	O3-C4-C3	109.59(9)
C8-C7-C6	119.98(10)	O3-C4-C5	105.92(9)
C11-C12-C7	120.15(12)	C3-C4-C5	112.73(9)
N1-C6-C7	120.87(10)	C9-C10-C11	119.72(12)
N1-C6-C5	114.21(10)	C10-C9-C8	120.19(12)
C7-C6-C5	124.92(10)	C12-C11-C10	120.50(12)
C9-C8-C7	120.44(11)	O1-C2-C1	107.79(11)

Compound characterisations

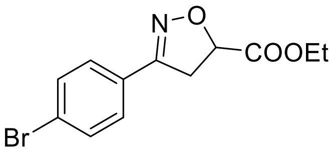

3aa

Ethyl 3-(4-bromophenyl)-4,5-dihydroisoxazole-5-carboxylate (3aa)

ethyl acetate/ petroleum ether =1:6; light yellow solid; 88% yield (131.6 mg); 20.0 mmol scale: 85% yield (5.1 g); mp: 66-68 ºC; ^{1}H NMR (400 MHz, $CDCl_3$) δ 7.51 (s, 4H), 5.15 (dd, *J* = 10.5, 7.9 Hz, 1H), 4.24 (q, *J* = 7.1 Hz, 2H), 3.592 (d, *J* = 7.9 Hz, 1H), 3.586 (d, *J* = 10.5 Hz, 1H), 1.30 (t, *J* = 7.1 Hz, 3H); ^{13}C NMR (100 MHz, $CDCl_3$) δ 169.9, 155.1, 131.9, 128.2, 127.4, 124.7, 78.2, 62.0, 38.5, 14.0; HRMS (ESI-TOF): Anal. Calcd. For $C_{12}H_{12}{}^{79}BrNNaO_3^+$: 319.9893, $C_{12}H_{12}{}^{81}BrNNaO_3^+$: 321.9872, Found: 319.9881, 321.9890; IR (neat, cm^{-1}): υ 2972, 2933, 1738, 1195, 1160, 1008, 890, 821.

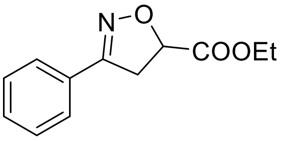

3ab

Ethyl 3-phenyl-4,5-dihydroisoxazole-5-carboxylate (3ab)

ethyl acetate/ petroleum ether =1:6; white solid; 92% yield (100.9 mg); 10.0 mmol scale: 92% yield (2.0 g); mp: 35-36 ºC; ^{1}H NMR (400 MHz, $CDCl_3$) δ 7.69 – 7.63 (m, 2H), 7.43 – 7.35 (m, 3H), 5.14 (dd, *J* = 10.4, 7.9 Hz, 1H), 4.24 (q, *J* = 7.1 Hz, 2H), 3.623 (d, *J* = 7.9 Hz, 1H), 3.617 (d, *J* = 10.4 Hz, 1H), 1.30 (t, *J* = 7.1 Hz, 3H); ^{13}C NMR (100 MHz, $CDCl_3$) δ 170.0, 155.9, 130.3, 128.6, 128.4, 126.8, 78.0, 61.8, 38.7, 14.0; Anal. Calcd. For $C_{12}H_{13}NNaO_3^+$: 242.0788, Found: 242.0778; IR (neat, cm^{-1}): υ 2976, 2936, 2906, 1750, 1209, 1182, 1035, 901, 762, 695.

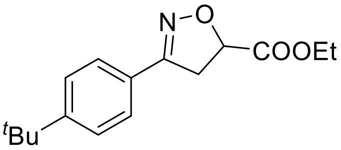

3ac

Ethyl 3-(4-(tert-butyl)phenyl)-4,5-dihydroisoxazole-5-carboxylate (3ac)

ethyl acetate/ petroleum ether =1:8; colorless liquid; 90% yield (123.9 mg); ^{1}H NMR (400 MHz, $CDCl_3$) δ 7.60 (d, *J* = 8.2 Hz, 2H), 7.41 (d, *J* = 8.2 Hz, 2H), 5.12 (dd, *J* = 10.2, 8.0 Hz, 1H), 4.23 (q, *J* = 7.1 Hz, 2H), 3.64 – 3.59 (m, 2H), 1.31 (s, 9H), 1.29 (t, *J* = 7.1 Hz, 3H); ^{13}C NMR (100 MHz, $CDCl_3$) δ 170.1, 155.7, 153.7, 126.6, 125.6, 125.5, 77.8, 61.7, 38.8, 34.7, 31.0, 13.9; HRMS (ESI-TOF): Anal. Calcd. For $C_{16}H_{21}NNaO_3^+$: 298.1414, Found: 298.1427; IR (neat, cm^{-1}): υ 2983, 2938, 1735, 1513, 1203, 1158, 893, 836.

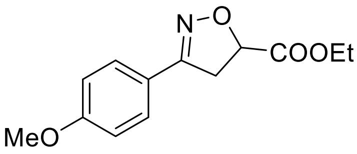

3ad

Ethyl 3-(4-methoxyphenyl)-4,5-dihydroisoxazole-5-carboxylate (3ad)

ethyl acetate/ petroleum ether =1:5; light yellow liquid; 85% yield (106.0 mg); ^{1}H NMR (400 MHz, $CDCl_3$) δ 7.59 (d, *J* = 8.9 Hz, 2H), 6.89 (d, *J* = 8.9 Hz, 2H), 5.11 (dd, *J* = 10.3, 8.0 Hz, 1H), 4.24 (q, *J* = 7.1 Hz, 2H), 3.81 (s, 3H), 3.593 (d, *J* = 8.0 Hz, 1H), 3.587 (d, *J* = 10.3 Hz, 1H), 1.30 (t, *J* = 7.1 Hz, 3H); ^{13}C NMR (100 MHz, $CDCl_3$) δ 170.3, 161.2, 155.4, 128.4, 121.0, 114.1, 77.8, 61.8, 55.3, 39.0, 14.0; HRMS (ESI-TOF): Anal. Calcd. For $C_{13}H_{15}NNaO_4^+$: 272.0893, Found: 272.0890; IR (neat, cm^{-1}): υ 2980, 2938, 2840, 1735, 1608, 1516, 1252, 1202, 1177, 1019, 888, 832.

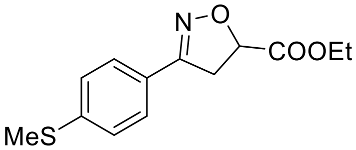

3ae

Ethyl 3-(4-(methylthio)phenyl)-4,5-dihydroisoxazole-5-carboxylate (3ae)

ethyl acetate/ petroleum ether =1:5; white solid; 83% yield (110.1 mg); mp: 63-65 °C; ^{1}H NMR (400 MHz, $CDCl_3$) δ 7.54 (d, *J* = 8.6 Hz, 2H), 7.20 (d, *J* = 8.6 Hz, 2H), 5.12 (dd, *J* = 10.3, 8.1 Hz, 1H), 4.23 (q, *J* = 7.1 Hz, 2H), 3.67 – 3.48 (m, 2H), 2.46 (s, 3H), 1.29 (t, *J* = 7.1 Hz, 3H); ^{13}C NMR (100 MHz, $CDCl_3$) δ 170.0, 155.4, 141.9, 127.0, 125.6, 124.7, 77.9, 61.8, 38.6, 14.9, 14.0; HRMS (ESI-TOF): Anal. Calcd. For $C_{13}H_{15}NNaO_3S^+$: 288.0665, Found: 288.0675; IR (neat, cm^{-1}): υ 2989, 2921, 1748, 1199, 1032, 1022, 894, 817.

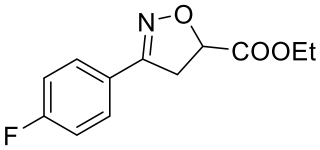

3af

Ethyl 3-(4-fluorophenyl)-4,5-dihydroisoxazole-5-carboxylate (3af)

ethyl acetate/ petroleum ether =1:6; white solid; 86% yield (101.9 mg); mp: 45-47 °C; ^{1}H NMR (400 MHz, $CDCl_3$) δ 7.72 – 7.60 (m, 2H), 7.12 – 7.03 (m, 2H), 5.14 (dd, *J* = 10.4, 8.0 Hz, 1H), 4.24 (q, *J* = 7.1 Hz, 2H), 3.69 – 3.47 (m, 2H), 1.30 (t, *J* = 7.1 Hz, 3H); ^{13}C NMR (100 MHz, $CDCl_3$) δ 170.0, 163.8 (d, *J* = 251.3 Hz), 154.9, 128.8 (d, *J* = 8.6 Hz), 124.8 (d, *J* = 3.4 Hz), 115.8 (d, *J* = 22.0 Hz), 78.1, 61.9, 38.7, 14.0; ^{19}F NMR (376 MHz, $CDCl_3$) δ -109.3; HRMS (ESI-TOF): Anal. Calcd. For $C_{12}H_{12}FNNaO_3^+$: 260.0693, Found: 260.0683; IR (neat, cm^{-1}): υ 2983, 2938, 1735, 1603, 1513, 1203, 1158, 894, 836.

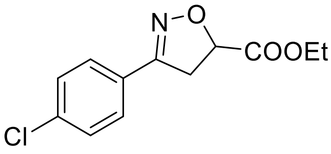

3ag

Ethyl 3-(4-chlorophenyl)-4,5-dihydroisoxazole-5-carboxylate (3ag)

ethyl acetate/ petroleum ether =1:6; white solid; 84% yield (106.3 mg); mp: 57-58 °C; ^{1}H NMR (400 MHz, $CDCl_3$) δ 7.58 (d, *J* = 8.6 Hz, 2H), 7.35 (d, *J* = 8.6 Hz, 2H), 5.15 (dd, *J* = 10.5, 7.9 Hz, 1H), 4.24 (q, *J* = 7.1 Hz, 2H), 3.60 (d, *J* = 7.9 Hz, 1H), 3.59 (d, *J* = 10.5 Hz, 1H), 1.30 (t, *J* = 7.1 Hz, 3H); ^{13}C NMR (100 MHz, $CDCl_3$) δ 169.9, 155.0, 136.3, 128.9, 128.0, 127.0, 78.2, 62.0, 38.5, 14.0; HRMS (ESI-TOF): Anal. Calcd. For $C_{12}H_{12}{}^{35}ClNNaO_3^+$: 276.0398, Found: 276.0393; IR (neat, cm^{-1}): υ 2987, 2963, 2906, 1749, 1340, 1215, 1203, 1032, 908, 839, 827.

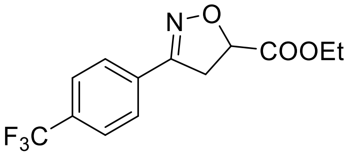

3ah

Ethyl 3-(4-(trifluoromethyl)phenyl)-4,5-dihydroisoxazole-5-carboxylate (3ah)

ethyl acetate/ petroleum ether =1:6; white solid; 60% yield (86.1 mg); mp: 90-91 °C; ^{1}H NMR (400 MHz, $CDCl_3$) δ 7.78 (d, *J* = 8.2 Hz, 2H), 7.66 (d, *J* = 8.2 Hz, 2H), 5.21 (dd, *J* = 11.0, 7.5 Hz, 1H), 4.27 (q, *J* = 7.2 Hz, 2H), 3.65 (d, *J* = 7.5 Hz, 1H), 3.64 (d, *J* = 11.0 Hz, 1H), 1.32 (t, *J* = 7.2 Hz, 3H); ^{13}C NMR (100 MHz, $CDCl_3$) δ 169.8, 155.0, 132.1 (q, *J* = 32.7 Hz), 132.0, 127.1, 125.7 (q, *J* = 3.7 Hz), 123.7 (q, *J* = 272.2 Hz), 78.5, 62.1, 38.4, 14.0; ^{19}F NMR (376 MHz, $CDCl_3$) δ -63.0; HRMS (ESI-TOF): Anal. Calcd. For $C_{13}H_{12}F_3NNaO_3^+$: 310.0661, Found: 310.0674; IR (neat, cm^{-1}): υ 2977, 2939, 1739, 1321, 1161, 1120, 1066, 886, 840.

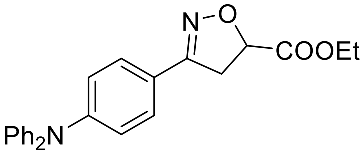

3ai

Ethyl 3-(4-(diphenylamino)phenyl)-4,5-dihydroisoxazole-5-carboxylate (3ai)

ethyl acetate/ petroleum ether =1:6; yellow liquid; 85% yield (164.3 mg); ^{1}H NMR (400 MHz, $CDCl_3$) δ 7.50 (d, *J* = 8.7 Hz, 2H), 7.31 – 7.23 (m, 4H), 7.15 – 6.99 (m, 8H), 5.12 (dd, *J* = 10.5, 7.5 Hz, 1H), 4.26 (q, *J* = 7.1 Hz, 2H), 3.59 (d, *J* = 7.5 Hz, 1H), 3.58 (d, *J* = 10.5 Hz, 1H), 1.32 (t, *J* = 7.1 Hz, 3H); ^{13}C NMR (100 MHz, $CDCl_3$) δ 170.3, 155.5, 149.8, 146.9, 129.4, 127.9, 125.2, 123.9, 121.7, 121.3, 77.8, 61.8, 39.0, 14.1; HRMS (ESI-TOF): Anal. Calcd. For $C_{24}H_{23}N_2O_3^+$: 387.1703, Found: 387.1706; IR (neat, cm^{-1}): υ 2952, 2939, 1736, 1589, 1487, 1272, 1028, 754, 696.

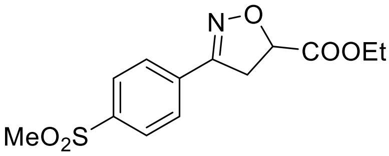

3aj

Ethyl 3-(4-(methylsulfonyl)phenyl)-4,5-dihydroisoxazole-5-carboxylate (3aj)

ethyl acetate/ petroleum ether =2:1; white solid; 59% yield (88.0 mg); mp: 98-100 °C; ^{1}H NMR (400 MHz, $CDCl_3$) δ 7.91 (d, *J* = 8.5 Hz, 2H), 7.81 (d, *J* = 8.5 Hz, 2H), 5.19 (t, *J* = 9.3 Hz, 1H), 4.23 (q, *J* = 7.1 Hz, 2H), 3.63 (d, *J* = 9.3 Hz, 2H), 3.03 (s, 3H), 1.28 (t, *J* = 7.1 Hz, 3H); ^{13}C NMR (100 MHz, $CDCl_3$)

δ 169.5, 154.7, 141.6, 133.6, 127.7, 127.5, 78.6, 62.0, 44.2, 38.1, 13.9; HRMS (ESI-TOF): Anal. Calcd. For $C_{13}H_{15}NNaO_5S^+$: 320.0563, Found: 320.0565; IR (neat, cm^{-1}): υ 3026, 3007, 2927, 1731, 1308, 1225, 1150, 892, 846, 777.

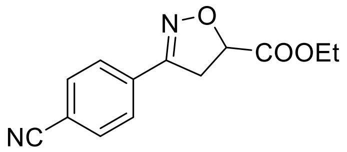

3ak

Ethyl 3-(4-cyanophenyl)-4,5-dihydroisoxazole-5-carboxylate (3ak)

ethyl acetate/ petroleum ether =1:4; white solid; 54% yield (66.2 mg); mp: 98-99 °C; ^{1}H NMR (400 MHz, $CDCl_3$) δ 7.77 (d, *J* = 8.1 Hz, 2H), 7.69 (d, *J* = 8.1 Hz, 2H), 5.21 (dd, *J* = 11.0, 7.6 Hz, 1H), 4.26 (q, *J* = 7.2 Hz, 2H), 3.71 – 3.57 (m, 2H), 1.31 (t, *J* = 7.2 Hz, 3H); ^{13}C NMR (100 MHz, $CDCl_3$) δ 169.5, 154.7, 132.8, 132.5, 127.3, 118.1, 113.7, 78.7, 62.2, 38.0, 14.0; HRMS (ESI-TOF): Anal. Calcd. For $C_{13}H_{12}N_2NaO_3^+$: 267.0740, Found: 267.0751; IR (neat, cm^{-1}): υ 2985, 2936, 2226, 1721, 1284, 1018, 889, 868, 849, 832.

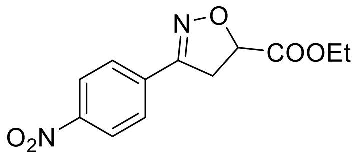

3al

Ethyl 3-(4-nitrophenyl)-4,5-dihydroisoxazole-5-carboxylate (3al)

ethyl acetate/ petroleum ether =1:4; yellow solid; 41% yield (54.0 mg); mp: 80-81 °C; ^{1}H NMR (400 MHz, $CDCl_3$) δ 8.25 (d, *J* = 8.8 Hz, 2H), 7.84 (d, *J* = 8.8 Hz, 2H), 5.24 (dd, *J* = 11.0, 7.6 Hz, 1H), 4.27 (q, *J* = 7.1 Hz, 2H), 3.68 (d, *J* = 7.6 Hz, 1H), 3.66 (d, *J* = 11.0 Hz, 1H), 1.32 (t, *J* = 7.1 Hz, 3H); ^{13}C NMR (100 MHz, $CDCl_3$) δ 169.5, 154.5, 148.6, 134.5, 127.6, 124.0, 78.9, 62.2, 38.1, 14.0; HRMS (ESI-TOF): Anal. Calcd. For $C_{12}H_{12}N_2NaO_5^+$: 287.0638, Found: 287.0649; IR (neat, cm^{-1}): υ 3115, 2988, 2965, 1728, 1513, 1342, 1256, 910, 848.

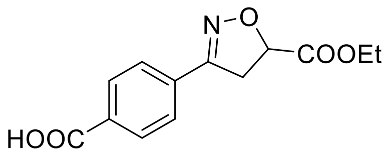

3am

4-(5-(Ethoxycarbonyl)-4,5-dihydroisoxazol-3-yl)benzoic acid (3am)

ethyl acetate/ petroleum ether =1:1; white solid; 72% yield (95.2 mg); mp: 94-96 °C; ^{1}H NMR (400 MHz, DMSO-d6) δ 8.00 (d, *J* = 8.1 Hz, 2H), 7.81 (d, *J* = 8.1 Hz, 2H), 5.31 (dd, *J* = 11.8, 6.8 Hz, 1H), 4.17 (q, *J* = 7.1 Hz, 2H), 3.81 (dd, *J* = 17.4, 11.8 Hz, 1H), 3.66 (dd, *J* = 17.4, 6.8 Hz, 1H), 1.23 (t, *J* = 7.1 Hz, 3H); ^{13}C NMR (100 MHz, DMSO-d6) δ 169.9, 166.8, 156.0, 132.4, 132.3, 129.8, 127.1, 78.1, 61.4, 38.3, 14.0; HRMS (ESI-TOF): Anal. Calcd. For $C_{13}H_{13}NNaO_5^+$: 286.0686, Found: 286.0673; IR (neat, cm^{-1}): υ 3071, 2980, 2921, 2673, 2553, 1754, 1681, 1288, 1197, 1025, 862, 771.

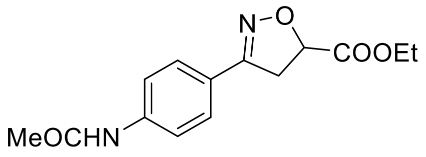

3an

Ethyl 3-(4-acetamidophenyl)-4,5-dihydroisoxazole-5-carboxylate (3an)

ethyl acetate/ petroleum ether =3:1; white solid; 53% yield (73.6 mg); mp: 135-136 °C; ^{1}H NMR (400 MHz, DMSO-d6) δ 10.15 (s, 1H), 7.68 (d, *J* = 8.6 Hz, 2H), 7.62 (d, *J* = 8.6 Hz, 2H), 5.22 (dd, *J* = 11.6, 6.6 Hz, 1H), 4.16 (q, *J* = 7.1 Hz, 2H), 3.72 (dd, *J* = 17.2, 11.6 Hz, 1H), 3.58 (dd, *J* = 17.2, 6.6 Hz, 1H), 2.07 (s, 3H), 1.22 (t, *J* = 7.1 Hz, 3H); ^{13}C NMR (100 MHz, DMSO-d6) δ 170.2, 168.8, 155.8, 141.3, 127.7, 122.9, 118.9, 77.5, 61.3, 38.7, 24.2, 14.0; HRMS (ESI-TOF): Anal. Calcd. For $C_{14}H_{16}N_2NaO_4^+$: 299.1002, Found: 299.1001; IR (neat, cm^{-1}): υ 3305, 3039, 2994, 2947, 1749, 1668, 1522, 1201, 1022, 896, 829.

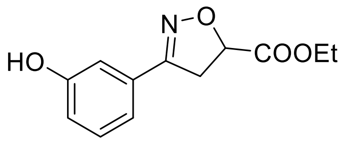

3ao

Ethyl 3-(3-hydroxyphenyl)-4,5-dihydroisoxazole-5-carboxylate (3ao)

ethyl acetate/ petroleum ether =1:4; white solid; 75% yield (87.9 mg); mp: 79-80 °C; ^{1}H NMR (400 MHz, CDCl$_3$) δ 7.27 – 7.19 (m, 3H), 7.09 (d, *J* = 8.0 Hz, 1H), 6.94 (dd, *J* = 8.0, 2.5 Hz, 1H), 5.15 (dd, *J* = 10.0, 8.4 Hz, 1H), 4.25 (q, *J* = 7.1 Hz, 2H), 3.65 – 3.57 (m, 2H), 1.29 (t, *J* = 7.1 Hz, 3H); ^{13}C NMR (100 MHz, CDCl$_3$) δ 170.5, 156.4, 156.3, 130.0, 129.2, 119.1, 118.2, 113.5, 77.9, 62.2, 39.0, 14.0; HRMS (ESI-TOF): Anal. Calcd. For $C_{12}H_{13}NNaO_4^+$: 258.0737, Found: 258.0724; IR (neat, cm^{-1}): υ 3451, 2987, 2960, 1737, 1450, 1191, 1160, 1024, 837, 789, 685.

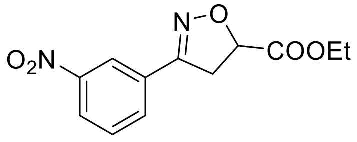

3ap

Ethyl 3-(3-nitrophenyl)-4,5-dihydroisoxazole-5-carboxylate (3ap)

ethyl acetate/ petroleum ether =1:4; white solid; 49% yield (64.3 mg); mp: 50-51 °C; ^{1}H NMR (400 MHz, CDCl$_3$) δ 8.43 – 8.41 (m, 1H), 8.28 – 8.24 (m, 1H), 8.08 – 8.04 (m, *1*H), 7.63 – 7.58 (m, 1H), 5.24 (dd, *J* = 10.8, 7.7 Hz, 1H), 4.27 (q, *J* = 7.1 Hz, 2H), 3.69 (d, *J* = 7.7 Hz, 1H), 3.68 (d, *J* = 10.8 Hz, 1H), 1.32 (t, *J* = 7.1 Hz, 3H); ^{13}C NMR (100 MHz, CDCl$_3$) δ 169.5, 154.4, 148.3, 132.4, 130.4, 129.9, 124.9, 121.6, 78.7, 62.2, 38.2, 14.0; HRMS (ESI-TOF): Anal. Calcd. For $C_{12}H_{12}N_2NaO_5^+$: 287.0638, Found: 287.0627; IR (neat, cm^{-1}): υ 3089, 2974, 2924, 1745, 1528, 1352, 1337, 1205, 891, 739, 679.

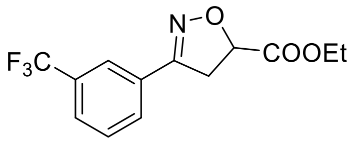

3aq

Ethyl 3-(3-(trifluoromethyl)phenyl)-4,5-dihydroisoxazole-5-carboxylate (3aq)

ethyl acetate/ petroleum ether =1:6; white solid; 73% yield (104.9 mg); mp: 34-35 ^{o}C; ^{1}H NMR (400 MHz, $CDCl_3$) δ 7.89 (s, 1H), 7.86 (d, *J* = 7.8 Hz, 1H), 7.66 (d, *J* = 7.8 Hz, 1H), 7.53 (t, *J* = 7.8 Hz, 1H), 5.20 (dd, *J* = 10.5, 7.9 Hz, 1H), 4.26 (q, *J* = 7.1 Hz, 2H), 3.65 (d, *J* = 7.9 Hz, 1H), 3.65 (d, *J* = 10.5 Hz, 1H), 1.31 (t, *J* = 7.1 Hz, 3H); ^{13}C NMR (100 MHz, $CDCl_3$) δ 169.7, 154.9, 131.2 (q, *J* = 32.7 Hz), 129.9, 129.4, 129.3, 126.9 (q, *J* = 3.7 Hz), 123.6 (q, *J* = 272.3 Hz), 123.6 (q, *J* = 3.8 Hz), 78.4, 62.1, 38.4, 14.0; ^{19}F NMR (376 MHz, $CDCl_3$) δ -62.9; HRMS (ESI-TOF): Anal. Calcd. For $C_{13}H_{12}F_3NNaO_3^+$: 310.0661, Found: 310.0648; IR (neat, cm^{-1}): υ 2979, 2938, 2907, 1738, 1311, 1165, 1122, 1098, 900, 803, 693.

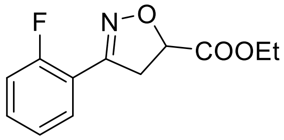

3ar

Ethyl 3-(2-fluorophenyl)-4,5-dihydroisoxazole-5-carboxylate (3ar)

ethyl acetate/ petroleum ether =1:8; colorless liquid; 71% yield (84.3 mg); ^{1}H NMR (400 MHz, $CDCl_3$) δ 7.87 – 7.82 (m, 1H), 7.43 – 7.35 (m, 1H), 7.18 – 7.13 (m, 1H), 7.12 – 7.06 (m, 1H), 5.14 (dd, *J* = 10.0, 8.6 Hz, 1H), 4.25 (q, *J* = 7.1 Hz, 2H), 3.72 – 3.68 (m, 2H), 1.30 (t, *J* = 7.2 Hz, 3H); ^{13}C NMR (100 MHz, $CDCl_3$) δ 170.0, 160.3 (d, *J* = 252.7 Hz), 152.7 (d, *J* = 3.0 Hz), 132.1 (d, *J* = 8.6 Hz), 129.1 (d, *J* = 3.0 Hz), 124.4 (d, *J* = 3.4 Hz), 116.6 (d, *J* = 11.5 Hz), 116.3 (d, *J* = 22.0 Hz), 78.1 (d, *J* = 2.3 Hz), 61.8, 40.4 (d, *J* = 7.7 Hz), 14.0; ^{19}F NMR (376 MHz, $CDCl_3$) δ -112.5; HRMS (ESI-TOF): Anal. Calcd. For $C_{12}H_{12}FNNaO_3^+$: 260.0693, Found: 260.0684; IR (neat, cm^{-1}): υ 2983, 2929, 2854, 1736, 1454, 1203, 1027, 898, 758.

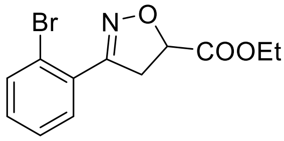

3as

Ethyl 3-(2-bromophenyl)-4,5-dihydroisoxazole-5-carboxylate (3as)

ethyl acetate/ petroleum ether =1:6; colorless liquid; 83% yield (124.1 mg); ^{1}H NMR (400 MHz, $CDCl_3$) δ 7.62 (dd, *J* = 7.6, 1.3 Hz, 1H), 7.53 (dd, *J* = 7.6, 1.8 Hz, 1H), 7.38 – 7.33 (m, 1H), 7.31 – 7.26 (m, 1H), 5.20 (dd, *J* = 11.0, 7.0 Hz, 1H), 4.28 (q, *J* = 7.1 Hz, 2H), 3.83 (dd, *J* = 17.3, 11.0 Hz, 1H), 3.76 (dd, *J* = 17.3, 7.0 Hz, 1H), 1.33 (t, *J* = 7.1 Hz, 3H); ^{13}C NMR (100 MHz, $CDCl_3$) δ 169.9, 156.9, 133.6, 131.2, 130.9, 130.1, 127.5, 121.7, 78.4, 61.8, 41.2, 14.0; HRMS (ESI-TOF): Anal. Calcd. For $C_{12}H_{12}{}^{79}BrNNaO_3^+$: 319.9893, $C_{12}H_{12}{}^{81}BrNNaO_3^+$: 321.9872, Found: 319.9874, 321.9912; IR (neat, cm^{-1}): υ 2982, 2938, 1736, 1341, 1200, 1026, 1016, 852, 756.

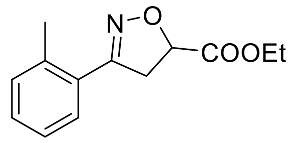

3at

Ethyl 3-(o-tolyl)-4,5-dihydroisoxazole-5-carboxylate (3at)

ethyl acetate/ petroleum ether =1:8; colorless liquid; 87% yield (101.5 mg); ^{1}H NMR (400 MHz, $CDCl_3$) δ 7.35 – 7.20 (m, 4H), 5.10 (t, *J* = 9.0 Hz, 1H), 4.26 (q, *J* = 7.1 Hz, 2H), 3.67 (d, *J* = 9.0 Hz, 2H), 2.55 (s, 3H), 1.31 (t, *J* = 7.1 Hz, 3H); ^{13}C NMR (100 MHz, $CDCl_3$) δ 170.2, 156.7, 138.1, 131.5, 129.6, 128.8, 127.6, 125.7, 77.0, 61.8, 41.3, 22.8, 14.0; HRMS (ESI-TOF): Anal. Calcd. For $C_{13}H_{15}NNaO_3^+$: 256.0944, Found: 256.0940; IR (neat, cm^{-1}): υ 2981, 2928, 1735, 1336, 1200, 1030, 889, 852, 758.

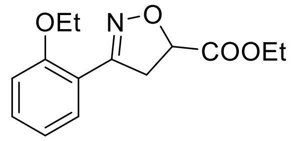

3au

Ethyl 3-(2-ethoxyphenyl)-4,5-dihydroisoxazole-5-carboxylate (3au)

ethyl acetate/ petroleum ether =1:6; white solid; 69% yield (91.2 mg); mp: 47-49 °C; ^{1}H NMR (400 MHz, $CDCl_3$) δ 7.77 (dd, *J* = 7.8, 1.7 Hz, 1H), 7.38 – 7.33 (m, 1H), 6.98 – 6.88 (m, 2H), 5.10 (dd, *J* = 11.0, 7.2 Hz, 1H), 4.26 (q, *J* = 7.1 Hz, 2H), 4.07 (q, *J* = 7.0 Hz, 2H), 3.86 – 3.68 (m, 2H), 1.43 (t, *J* = 7.0 Hz, 3H), 1.31 (t, *J* = 7.1 Hz, 3H); ^{13}C NMR (100 MHz, $CDCl_3$) δ 170.6, 156.8, 155.6, 131.5, 129.4, 120.6, 117.6, 112.0, 78.0, 63.9, 61.6, 41.5, 14.6, 14.0; HRMS (ESI-TOF): Anal. Calcd. For $C_{14}H_{17}NNaO_4^+$: 286.1050, Found: 286.1063; IR (neat, cm^{-1}): υ 2983, 2950, 2939, 2890, 1730, 1453, 1282, 1255, 1030, 888, 760.

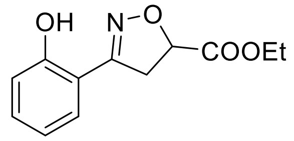

3av

Ethyl 3-(2-hydroxyphenyl)-4,5-dihydroisoxazole-5-carboxylate (3av)

ethyl acetate/ petroleum ether =1:6; red liquid; 51% yield (60.1 mg); ^{1}H NMR (400 MHz, $CDCl_3$) δ 9.54 (s, 1H), 7.37 – 7.30 (m, 1H), 7.19 (d, *J* = 7.8 Hz, 1H), 7.03 (d, *J* = 8.1 Hz, 1H), 6.95 – 6.89 (m, 1H), 5.13 (dd, *J* = 10.8, 7.4 Hz, 1H), 4.27 (q, *J* = 7.2 Hz, 2H), 3.79 – 3.68 (m, 2H), 1.32 (t, *J* = 7.2 Hz, 3H); ^{13}C NMR (100 MHz, $CDCl_3$) δ 169.6, 157.8, 157.3, 132.1, 128.5, 119.6, 117.1, 113.0, 76.7, 62.2, 39.0, 14.0; HRMS (ESI-TOF): Anal. Calcd. For $C_{12}H_{13}NNaO_4^+$: 258.0737, Found: 258.0730; IR (neat, cm^{-1}): υ 3211, 3057, 2984, 2939, 1737, 1494, 1258, 1201, 1157, 754, 655.

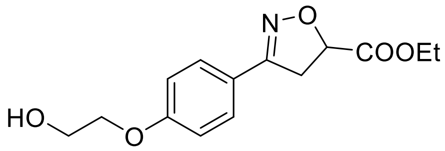

3aw

Ethyl 3-(4-(2-hydroxyethoxy)phenyl)-4,5-dihydroisoxazole-5-carboxylate (3aw)

ethyl acetate/ petroleum ether =1:1; white solid; 77% yield (107.1 mg); ^{1}H NMR (400 MHz, $CDCl_3$) δ 7.55 (d, *J* = 8.8 Hz, 2H), 6.88 (d, *J* = 8.8 Hz, 2H), 5.09 (dd, *J* = 9.8, 8.5 Hz, 1H), 4.22 (q, *J* = 7.1 Hz, 2H), 4.09 – 4.04 (m, 2H), 3.94 (t, *J* = 4.5 Hz, 2H), 3.67 – 3.45 (m, 2H), 2.65 (s, 1H), 1.28 (t, *J* = 7.1 Hz, 3H); ^{13}C NMR (100 MHz, $CDCl_3$) δ 170.2, 160.3, 155.4, 128.4, 121.2, 114.6, 77.7, 69.2, 61.8, 61.0, 38.9, 14.0; HRMS (ESI-TOF): Anal. Calcd. For $C_{14}H_{17}NNaO_5^+$: 302.0999, Found: 302.0989; IR (neat, cm^{-1}): υ 3412, 2993, 2944, 1742, 1257, 1210, 1169, 1076, 1026, 890, 837, 818.

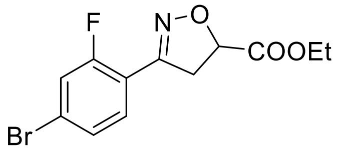

3ax

Ethyl 3-(4-bromo-2-fluorophenyl)-4,5-dihydroisoxazole-5-carboxylate (3ax)

ethyl acetate/ petroleum ether =1:10; white solid; 74% yield (117.0 mg); mp: 77-78 ºC; ^{1}H NMR (400 MHz, $CDCl_3$) δ 7.76 – 7.71 (m, 1H), 7.33 – 7.26 (m, 2H), 5.15 (t, *J* = 9.3 Hz, 1H), 4.25 (q, *J* = 7.1 Hz, 2H), 3.68 (d, *J* = 9.3 Hz, 1H), 3.67 (d, *J* = 9.3 Hz, 1H), 1.30 (t, *J* = 7.1 Hz, 3H); ^{13}C NMR (100 MHz, $CDCl_3$) δ 169.8, 159.8 (d, *J* = 257.1 Hz), 152.0 (d, *J* = 3.3 Hz), 130.0 (d, *J* = 3.6 Hz), 128.0 (d, *J* = 3.4 Hz), 124.9 (d, *J* = 9.8 Hz), 120.0 (d, *J* = 25.4 Hz), 115.8 (d, *J* = 11.7 Hz), 78.3 (d, *J* = 2.5 Hz), 61.9, 40.1 (d, *J* = 7.7 Hz), 14.0; ^{19}F NMR (376 MHz, $CDCl_3$) δ -100.3; HRMS (ESI-TOF): Anal. Calcd. For $C_{12}H_{11}{}^{79}BrFNNaO_3^+$: 337.9799, $C_{12}H_{11}{}^{81}BrFNNaO_3^+$: 339.9778, Found: 337.9795, 339.9771; IR (neat, cm^{-1}): υ 3072, 2985, 2929, 2855, 1726, 1594, 1203, 1170, 908, 878, 869, 823.

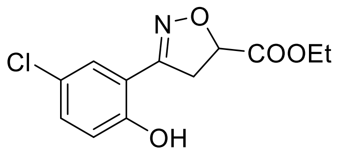

3ay

Ethyl 3-(5-chloro-2-hydroxyphenyl)-4,5-dihydroisoxazole-5-carboxylate (3ay)

ethyl acetate/ petroleum ether =1:8; pale pink solid; 62% yield (83.7 mg); mp: 83-84 ºC; ^{1}H NMR (400 MHz, $CDCl_3$) δ 9.55 (s, 1H), 7.28 (d, *J* = 8.9 Hz, 1H), 7.16 (s, 1H), 6.98 (d, *J* = 8.9 Hz, 1H), 5.16 (dd, *J* = 11.0, 7.2 Hz, 1H), 4.29 (q, *J* = 7.1 Hz, 2H), 3.81 – 3.61 (m, 2H), 1.34 (t, *J* = 7.1 Hz, 3H); ^{13}C NMR (100 MHz, $CDCl_3$) δ 169.3, 156.9, 155.9, 131.9, 127.7, 124.3, 118.6, 114.3, 77.0, 62.4, 38.8, 14.1; HRMS (ESI-TOF): Anal. Calcd. For $C_{12}H_{12}{}^{35}ClNNaO_4^+$: 292.0347, Found: 292.0329; IR (neat, cm^{-1}): υ 3072, 2996, 2967, 2930, 2911, 1751, 1384, 1204, 1193, 1170, 812, 667.

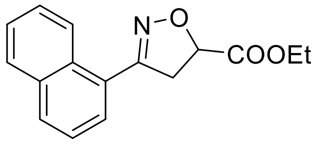

3az

Ethyl 3-(naphthalen-1-yl)-4,5-dihydroisoxazole-5-carboxylate (3az)

ethyl acetate/ petroleum ether =1:6; colorless liquid; 76% yield (102.4 mg); ^{1}H NMR (400 MHz, $CDCl_3$) δ 8.99 (d, *J* = 8.6 Hz, 1H), 7.91 – 7.85 (m, 2H), 7.62 – 7.57 (m, 1H), 7.56 – 7.50 (m, 2H), 7.45 (dd, *J* = 8.1, 7.3 Hz, 1H), 5.17 (dd, *J* = 9.8, 8.2 Hz, 1H), 4.29 (q, *J* = 7.1 Hz, 2H), 3.85 – 3.78 (m, 2H), 1.33 (t, *J*

= 7.1 Hz, 3H); ^{13}C NMR (100 MHz, $CDCl_3$) δ 170.1, 156.4, 133.8, 131.1, 130.4, 128.4, 127.8, 127.5, 126.9, 126.3, 125.4, 124.6, 77.0, 61.9, 41.6, 14.0; HRMS (ESI-TOF): Anal. Calcd. For $C_{16}H_{15}NNaO_3^+$: 292.0944, Found: 292.0934; IR (neat, cm^{-1}): υ 3050, 2982, 2938, 1735, 1318, 1202, 1024, 891, 801, 773.

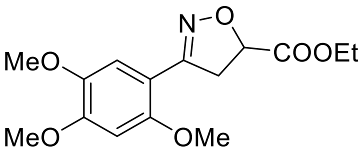

3ba

Ethyl 3-(2,4,5-trimethoxyphenyl)-4,5-dihydroisoxazole-5-carboxylate (3ba)

ethyl acetate/ petroleum ether =1:2; light yellow solid; 77% yield (119.4 mg); mp: 72-74 °C; ^{1}H NMR (400 MHz, $CDCl_3$) δ 7.31 (s, 1H), 6.45 (s, 1H), 5.02 (dd, *J* = 11.2, 7.1 Hz, 1H), 4.20 (q, *J* = 7.1 Hz, 2H), 3.86 (s, 3H), 3.79 (s, 3H), 3.78 (s, 3H), 3.75 – 3.63 (m, 2H), 1.26 (t, *J* = 7.1 Hz, 3H); ^{13}C NMR (100 MHz, $CDCl_3$) δ 170.6, 154.9, 152.5, 151.7, 143.0, 111.1, 108.6, 96.9, 77.8, 61.5, 56.14, 56.11, 55.8, 41.4, 13.9; HRMS (ESI-TOF): Anal. Calcd. For $C_{15}H_{19}NNaO_6^+$: 332.1105, Found: 332.1104; IR (neat, cm^{-1}): υ 3003, 2978, 2939, 2839, 1715, 1462, 1271, 1207, 1162, 1025, 797.

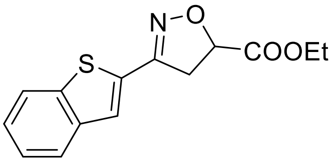

3ca

Ethyl 3-(benzo[b]thiophen-2-yl)-4,5-dihydroisoxazole-5-carboxylate (3ca)

ethyl acetate/ petroleum ether =1:5; light yellow solid; 69% yield (94.7 mg); mp: 97-99 °C; ^{1}H NMR (400 MHz, $CDCl_3$) δ 7.83 – 7.74 (m, 2H), 7.42 – 7.32 (m, 3H), 5.20 (dd, *J* = 11.2, 7.2 Hz, 1H), 4.28 (q, *J* = 7.0 Hz, 2H), 3.80 – 3.62 (m, 2H), 1.33 (t, *J* = 7.0 Hz, 3H); ^{13}C NMR (100 MHz, $CDCl_3$) δ 169.7, 152.3, 140.5, 138.8, 131.0, 126.14, 126.12, 124.7, 124.2, 122.4, 78.6, 62.1, 39.0, 14.0; HRMS (EI-TOF): Anal. Calcd. For $C_{14}H_{13}NO_3S$: 275.0616, Found: 275.0614; IR (neat, cm^{-1}): υ 2983, 2961, 2922, 1747, 1193, 1163, 1153, 898, 832, 749, 727.

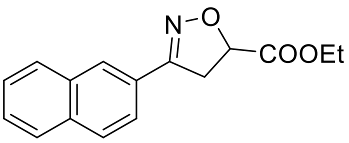

3da

Ethyl 3-(naphthalen-2-yl)-4,5-dihydroisoxazole-5-carboxylate (3da)

ethyl acetate/ petroleum ether =1:6; white solid; 69% yield (92.9 mg); mp: 63-64 °C; ^{1}H NMR (400 MHz, $CDCl_3$) δ 7.94 (dd, *J* = 8.7, 1.8 Hz, 1H), 7.88 (s, 1H), 7.85 – 7.78 (m, 3H), 7.54 – 7.46 (m, 2H), 5.20 (dd, *J* = 11.3, 7.1 Hz, 1H), 4.27 (q, *J* = 7.1 Hz, 2H), 3.76 (dd, *J* = 16.8, 7.1 Hz, 1H), 3.69 (dd, *J* = 16.8, 11.3 Hz, 1H), 1.32 (t, *J* = 7.1 Hz, 3H); ^{13}C NMR (100 MHz, $CDCl_3$) δ 170.1, 156.0, 134.0, 132.7, 128.4, 128.3, 127.7, 127.2, 127.1, 126.6, 126.0, 123.4, 78.1, 61.9, 38.6, 14.0; HRMS (ESI-TOF): Anal. Calcd. For $C_{16}H_{15}NNaO_3^+$: 292.0944, Found: 292.0940; IR (neat, cm^{-1}): υ 3062, 2983, 2955, 1743, 1200, 1191, 1162, 898, 821, 749.

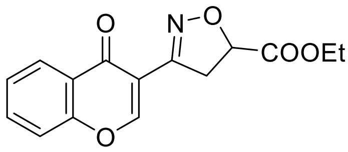

3ea

Ethyl 3-(4-oxo-4H-chromen-3-yl)-4,5-dihydroisoxazole-5-carboxylate (3ea)

ethyl acetate/ petroleum ether =1:4; light yellow solid; 75% yield (107.5 mg); mp: 99-101 ^oC; ^1H NMR (400 MHz, $CDCl_3$) δ 8.46 (s, 1H), 8.16 (dd, *J* = 8.1, 1.7 Hz, 1H), 7.67 (ddd, *J* = 8.7, 7.1, 1.7 Hz, 1H), 7.48 – 7.37 (m, 2H), 5.10 (dd, *J* = 11.8, 6.9 Hz, 1H), 4.22 (q, *J* = 7.1 Hz, 2H), 3.90 (dd, *J* = 18.1, 11.8 Hz, 1H), 3.75 (dd, *J* = 18.1, 6.9 Hz, 1H), 1.27 (t, *J* = 7.1 Hz, 3H); ^{13}C NMR (100 MHz, $CDCl_3$) δ 175.0, 170.0, 155.8, 154.9, 151.7, 134.2, 125.9, 125.8, 123.9, 118.2, 114.4, 77.9, 61.7, 40.4, 14.0; HRMS (ESI-TOF): Anal. Calcd. For $C_{15}H_{13}NNaO_5^+$: 310.0686, Found: 310.0690; IR (neat, cm^{-1}): υ 2977, 2924, 1754, 1650, 1615, 1465, 1189, 1034, 813, 759.

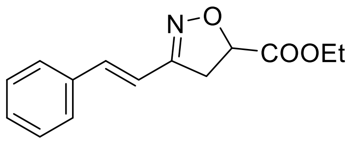

3fa

Ethyl (E)-3-styryl-4,5-dihydroisoxazole-5-carboxylate (3fa)

ethyl acetate/ petroleum ether =1:6; white solid; 58% yield (71.4 mg); mp: 64-66 ^oC; ^1H NMR (400 MHz, $CDCl_3$) δ 7.49 – 7.42 (m, 2H), 7.39 – 7.29 (m, 3H), 7.06 (d, *J* = 16.5 Hz, 1H), 6.77 (d, *J* = 16.5 Hz, 1H), 5.10 (dd, *J* = 10.8, 7.5 Hz, 1H), 4.26 (q, *J* = 7.1 Hz, 2H), 3.49 (d, *J* = 7.5 Hz, 1H), 3.48 (d, *J* = 10.8 Hz, 1H), 1.32 (t, *J* = 7.1 Hz, 3H); ^{13}C NMR (100 MHz, $CDCl_3$) δ 170.0, 156.9, 137.4, 135.4, 129.1, 128.8, 127.0, 116.8, 78.0, 61.9, 37.4, 14.0; HRMS (ESI-TOF): Anal. Calcd. For $C_{14}H_{15}NNaO_3^+$: 268.0944, Found: 268.0936; IR (neat, cm^{-1}): υ 3069, 2978, 2924, 1737, 1589, 1362, 1340, 1162, 1073, 894, 882, 785, 691.

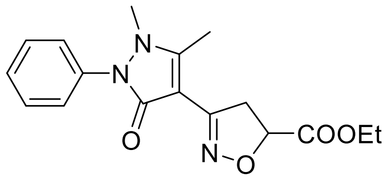

3ga

Ethyl 3-(1,5-dimethyl-3-oxo-2-phenyl-2,3-dihydro-1H-pyrazol-4-yl)-4,5-dihydroisoxazole-5-carboxylate (3ga)

ethyl acetate/ petroleum ether =4:1; white solid; 87% yield (143.9 mg); mp: 148-149 ^oC; ^1H NMR (400 MHz, $CDCl_3$) δ 7.48 – 7.42 (m, 2H), 7.36 – 7.27 (m, 3H), 4.96 (dd, *J* = 10.5, 7.6 Hz, 1H), 4.19 (q, *J* = 7.1 Hz, 2H), 3.85 – 3.76 (m, 2H), 3.23 (s, 3H), 2.56 (s, 3H), 1.25 (t, *J* = 7.1 Hz, 3H); ^{13}C NMR (100 MHz, $CDCl_3$) δ 170.6, 163.6, 153.4, 151.3, 134.1, 129.3, 127.7, 125.3, 97.0, 76.2, 61.5, 40.7, 34.5, 14.0, 13.1; HRMS (ESI-TOF): Anal. Calcd. For $C_{17}H_{20}N_3O_4^+$: 330.1448, Found: 330.1468; IR (neat, cm^{-1}): υ 2988, 2918, 2852, 1738, 1650, 1548, 1310, 1281, 1089, 1032, 976, 854, 751.

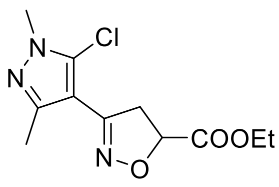

3ha

Ethyl 3-(5-chloro-1,3-dimethyl-1H-pyrazol-4-yl)-4,5-dihydroisoxazole-5-carboxylate (3ha)

ethyl acetate/ petroleum ether =1:2; white solid; 73% yield (99.1 mg); mp: 107-108 °C; ^{1}H NMR (400 MHz, $CDCl_3$) δ 5.02 (dd, *J* = 10.7, 7.5 Hz, 1H), 4.21 (q, *J* = 7.1 Hz, 2H), 3.75 (s, 3H), 3.65 (d, *J* = 7.5 Hz, 1H), 3.64 (d, *J* = 10.7 Hz, 1H), 2.36 (s, 3H), 1.27 (t, *J* = 7.1 Hz, 3H); ^{13}C NMR (100 MHz, $CDCl_3$) δ 170.1, 149.5, 148.3, 127.1, 106.3, 76.9, 61.8, 40.0, 36.1, 15.1, 14.0; HRMS (ESI-TOF): Anal. Calcd. For $C_{11}H_{14}{}^{35}ClN_3NaO_3^+$: 294.0616, $C_{11}H_{14}{}^{37}ClN_3NaO_3^+$: 296.0586, Found: 294.0625, 296.0592; IR (neat, cm^{-1}): υ 2996, 2937, 2851, 1729, 1529, 1367, 1275, 1028, 892, 808.

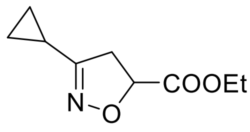

3ia

Ethyl 3-cyclopropyl-4,5-dihydroisoxazole-5-carboxylate (3ia)

ethyl acetate/ petroleum ether =1:5; colorless liquid; 41% yield (37.9 mg); ^{1}H NMR (400 MHz, $CDCl_3$) δ 4.93 (dd, *J* = 9.4, 8.5 Hz, 1H), 4.22 (q, *J* = 7.1 Hz, 2H), 3.08 – 3.04 (m, 2H), 1.80 – 1.73 (m, 1H), 1.29 (t, *J* = 7.1 Hz, 3H), 0.93 – 0.88 (m, 2H), 0.80 – 0.75 (m, 2H); ^{13}C NMR (100 MHz, $CDCl_3$) δ 170.4, 160.4, 77.0, 61.8, 39.1, 14.0, 8.5, 6.4, 6.0; HRMS (ESI-TOF): Anal. Calcd. For $C_9H_{13}NNaO_3^+$: 206.0788, Found: 206.0785; IR (neat, cm^{-1}): υ 3092, 2984, 2939, 2876, 1734, 1200, 1029, 874, 848.

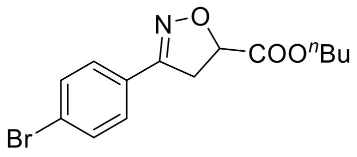

4aa

Butyl 3-(4-bromophenyl)-4,5-dihydroisoxazole-5-carboxylate (4aa)

ethyl acetate/ petroleum ether =1:20; white solid; 88% yield (131.4 mg); mp: 58-59 °C; ^{1}H NMR (400 MHz, $CDCl_3$) δ 7.52 (s, 4H), 5.16 (dd, *J* = 10.4, 8.0 Hz, 1H), 4.19 (t, *J* = 6.7 Hz, 2H), 3.63 – 3.56 (m, 2H), 1.70 – 1.61 (m, 2H), 1.43– 1.32 (m, 2H), 0.92 (t, *J* = 7.4 Hz, 3H); ^{13}C NMR (100 MHz, $CDCl_3$) δ 169.9, 155.1, 131.9, 128.2, 127.5, 124.7, 78.2, 65.8, 38.5, 30.4, 18.9, 13.6; ^{1}HRMS (ESI-TOF): Anal. Calcd. For $C_{14}H_{16}{}^{79}BrNNaO_3^+$: 348.0206, $C_{14}H_{16}{}^{81}BrNNaO_3^+$: 350.0185, Found: 348.0188, 350.0204; IR (neat, cm^{-1}): υ 2958, 2931, 2872, 1738, 1210, 1058, 1006, 888, 861, 819.

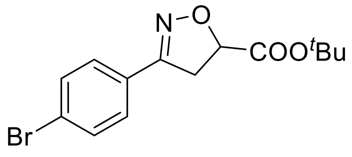

4ab

tert-Butyl 3-(4-bromophenyl)-4,5-dihydroisoxazole-5-carboxylate (4ab)

ethyl acetate/ petroleum ether =1:25; white solid; 80% yield (130.6 mg); mp: 66-68 °C; ^{1}H NMR (400 MHz, CDCl$_3$) δ 7.50 (s, 4H), 5.03 (t, *J* = 9.4 Hz, 1H), 3.53 (d, *J* = 9.4 Hz, 2H), 1.48 (s, 9H); ^{13}C NMR (100 MHz, CDCl$_3$) δ 168.8, 154.9, 131.8, 128.2, 127.6, 124.5, 82.7, 78.9, 38.3, 27.8; ^{1}HRMS (ESI-TOF): Anal. Calcd. For $C_{14}H_{16}{}^{79}BrNNaO_3^+$: 348.0206, $C_{14}H_{16}{}^{81}BrNNaO_3^+$: 350.0185, Found: 348.0195, 350.0200; IR (neat, cm^{-1}): υ 2976, 2935, 1733, 1590, 1346, 1149, 1007, 894, 866, 834, 820.

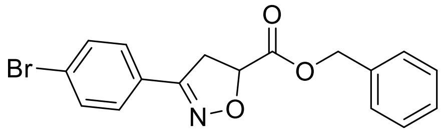

4ac

Benzyl 3-(4-bromophenyl)-4,5-dihydroisoxazole-5-carboxylate (4ac)

ethyl acetate/ petroleum ether =1:6; white solid; 83% yield (149.6 mg); mp: 74-76 °C; ^{1}H NMR (400 MHz, CDCl$_3$) δ 7.51 (s, 4H), 7.39 – 7.32 (m, 5H), 5.24 – 5.17 (m, 3H), 3.59 (d, *J* = 7.7 Hz, 1H), 3.58 (d, *J* = 10.7 Hz, 1H); ^{13}C NMR (100 MHz, CDCl$_3$) δ 169.6, 155.1, 134.8, 131.8, 128.5, 128.4, 128.2, 127.3, 124.7, 78.1, 67.4, 38.4; ^{1}HRMS (ESI-TOF): Anal. Calcd. For $C_{17}H_{14}{}^{79}BrNNaO_3^+$: 382.0049, $C_{17}H_{14}{}^{81}BrNNaO_3^+$: 384.0029, Found: 382.0042, 384.0030; IR (neat, cm^{-1}): υ 3068, 3037, 2955, 1755, 1174, 1162, 881, 825, 734, 693.

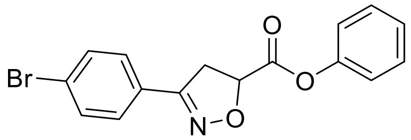

4ad

Phenyl 3-(4-bromophenyl)-4,5-dihydroisoxazole-5-carboxylate (4ad)

ethyl acetate/ petroleum ether =1:6; yellow solid; 67% yield (115.6 mg); mp: 109-110 °C; ^{1}H NMR (400 MHz, CDCl$_3$) δ 7.59 – 7.50 (m, 4H), 7.43 – 7.33 (m, 2H), 7.28 – 7.21 (m, 1H), 7.16 – 7.10 (m, 2H), 5.41 (dd, *J* = 11.3, 7.0 Hz, 1H), 3.77 (dd, *J* = 17.0, 7.0 Hz, 1H), 3.70 (dd, *J* = 17.0, 11.3 Hz, 1H); ^{13}C NMR (100 MHz, CDCl$_3$) δ 168.3, 155.2, 150.1, 131.9, 129.4, 128.3, 126.2, 124.8, 121.0, 78.1, 38.5; ^{1}HRMS (ESI-TOF): Anal. Calcd. For $C_{16}H_{12}{}^{79}BrNNaO_3^+$: 367.9893, $C_{16}H_{12}{}^{81}BrNNaO_3^+$: 369.9872, Found: 367.9881, 369.9859; IR (neat, cm^{-1}): υ 3070, 3043, 2976, 2927, 1773, 1589, 1489, 1193, 1162, 1069, 818, 748.

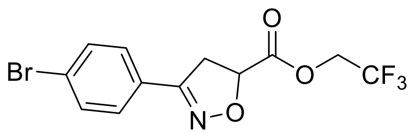

4ae

2,2,2-Trifluoroethyl 3-(4-bromophenyl)-4,5-dihydroisoxazole-5-carboxylate (4ae)

ethyl acetate/ petroleum ether =1:6; white solid; 60% yield (105.5 mg); mp: 72-74 °C; ^{1}H NMR (400 MHz, CDCl$_3$) δ 7.58 – 7.52 (m, 4H), 5.28 (dd, *J* = 11.1, 7.0 Hz, 1H), 4.65 (dq, *J* = 12.6, 8.3 Hz, 1H), 4.53 (dq, *J* = 12.6, 8.3 Hz, 1H), 3.76 – 3.56 (m, 2H); ^{13}C NMR (100 MHz, CDCl$_3$) δ 168.4, 155.1, 132.1, 128.4, 127.1, 125.1, 122.5 (q, *J* = 277.2 Hz), 77.5, 61.2 (q, *J* = 37.1 Hz), 38.7; ^{19}F NMR (376 MHz,

$CDCl_3$) δ -73.7; HRMS (ESI-TOF): Anal. Calcd. For $C_{12}H_9{}^{79}BrF_3NNaO_3{}^+$: 373.9610, $C_{12}H_9{}^{81}BrF_3NNaO_3{}^+$: 375.9590, Found: 373.9605, 375.9593; IR (neat, cm^{-1}): υ 2991, 2977, 2923, 1772, 1274, 1176, 1161, 1054, 971, 894, 824.

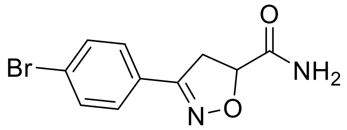

4af

3-(4-Bromophenyl)-4,5-dihydroisoxazole-5-carboxamide (4af)

ethyl acetate/ petroleum ether =2:1; white solid; 53% yield (71.5 mg); mp: >200 ºC; ^{1}H NMR (400 MHz, DMSO-d6) δ 7.71 – 7.58 (s, 5H), 7.44 (s, 1H), 5.07 (dd, *J* = 11.6, 7.0 Hz, 1H), 3.66 (dd, *J* = 17.3, 11.7 Hz, 1H), 3.53 (dd, *J* = 17.3, 7.0 Hz, 1H); ^{13}C NMR (100 MHz, DMSO-d6) δ 171.9, 155.8, 131.9, 128.8, 128.0, 123.8, 79.3, 38.3; HRMS (ESI-TOF): Anal. Calcd. For $C_{10}H_9{}^{79}BrN_2NaO_2{}^+$: 290.9740, $C_{10}H_9{}^{81}BrN_2NaO_2{}^+$: 292.9719, Found: 290.9724, 292.9711; IR (neat, cm^{-1}): υ 3410, 3185, 2969, 2928, 1650, 1591, 1401, 1010, 896, 820.

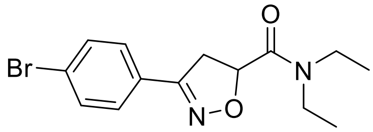

4ag

3-(4-Bromophenyl)-N,N-diethyl-4,5-dihydroisoxazole-5-carboxamide (4ag)

ethyl acetate/ petroleum ether =1:2; white solid; 85% yield (137.9 mg); mp: 114-116 ºC; ^{1}H NMR (400 MHz, Benzene-d6) δ 7.22 (d, *J* = 8.4 Hz, 2H), 7.14 (d, *J* = 8.4 Hz, 2H), 4.78 (dd, *J* = 11.2, 7.7 Hz, 1H), 4.11 (dd, *J* = 16.6, 7.7 Hz, 1H), 3.18 – 3.07 (m, 3H), 3.00 (dq, *J* = 14.4, 7.1 Hz, 1H), 2.63 (dd, *J* = 16.6, 11.2 Hz, 1H), 0.93 (t, *J* = 7.1 Hz, 3H), 0.88 (t, *J* = 7.1 Hz, 3H); ^{13}C NMR (100 MHz, Benzene-d6) δ 166.3, 156.3, 132.0, 128.7, 128.6, 124.3, 78.8, 41.8, 40.7, 36.7, 14.4, 12.8; HRMS (ESI-TOF): Anal. Calcd. For $C_{14}H_{17}{}^{79}BrN_2NaO_2{}^+$: 347.0366, $C_{14}H_{17}{}^{81}BrN_2NaO_2{}^+$: 349.0345, Found: 347.0332, 349.0329; IR (neat, cm^{-1}): υ 2968, 2930, 2871, 1633, 1261, 1068, 1005, 895, 830.

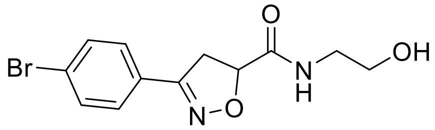

4ah

3-(4-Bromophenyl)-N-(2-hydroxyethyl)-4,5-dihydroisoxazole-5-carboxamide (4ah)

ethyl acetate/ petroleum ether =6:1; white solid; 45% yield (69.8 mg); mp: 135-137 ºC; ^{1}H NMR (400 MHz, DMSO-d6) δ 8.14 (t, *J* = 5.7 Hz, 1H), 7.68 – 7.60 (m, 4H), 5.12 (dd, *J* = 11.6, 7.0 Hz, 1H), 4.71 (t, *J* = 5.5 Hz, 1H), 3.68 (dd, *J* = 17.2, 11.6 Hz, 1H), 3.54 (dd, *J* = 17.2, 7.0 Hz, 1H), 3.45 – 3.40 (m, 2H), 3.26 – 3.10 (m, 2H); ^{13}C NMR (100 MHz, DMSO-d6) δ 169.7, 156.0, 131.9, 128.8, 128.0, 123.8, 79.4, 59.6, 41.6, 38.5; HRMS (ESI-TOF): Anal. Calcd. For $C_{12}H_{13}{}^{79}BrN_2NaO_3{}^+$: 335.0002, $C_{12}H_{13}{}^{81}BrN_2NaO_3{}^+$: 336.9981, Found: 334.9983, 336.9962; IR (neat, cm^{-1}): υ 3240, 2950, 2924, 2887, 1641, 1535, 1204, 1058, 1038, 902, 825.

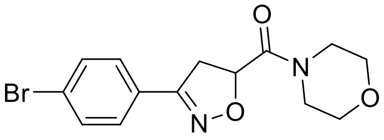

4ai

(3-(4-Bromophenyl)-4,5-dihydroisoxazol-5-yl)(morpholino)methanone (4ai)

ethyl acetate/ petroleum ether =1:1; white solid; 75% yield (127.8 mg); mp: 120-121 ºC; ^{1}H NMR (400 MHz, DMSO-d6) δ 7.73 – 7.59 (m, 4H), 5.62 (dd, *J* = 11.4, 7.2 Hz, 1H), 3.83 (dd, *J* = 17.0, 7.2 Hz, 1H), 3.73 – 3.43 (m, 9H); ^{13}C NMR (100 MHz, DMSO-d6) δ 166.2, 156.2, 131.9, 128.8, 128.0, 123.8, 77.2, 66.2, 66.1, 45.8, 42.3, 36.6; HRMS (ESI-TOF): Anal. Calcd. For $C_{14}H_{15}{}^{79}BrN_2NaO_3^+$: 361.0158, $C_{14}H_{15}{}^{81}BrN_2NaO_3^+$: 363.0138, Found: 361.0139, 363.0139; IR (neat, cm^{-1}): υ 2969, 2930, 2860, 1637, 1422, 1234, 1113, 1024, 891, 826.

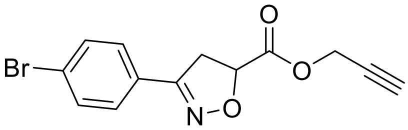

4aj

Prop-2-yn-1-yl 3-(4-bromophenyl)-4,5-dihydroisoxazole-5-carboxylate (4aj)

ethyl acetate/ petroleum ether =1:6; light yellow solid; 60% yield (92.7 mg); mp: 57-58 ºC; ^{1}H NMR (400 MHz, $CDCl_3$) δ 7.51 (s, 4H), 5.21 (t, *J* = 9.2 Hz, 1H), 4.81 (dd, *J* = 15.5, 2.5 Hz, 1H), 4.75 (dd, *J* = 15.5, 2.5 Hz, 1H), 3.62 (d, *J* = 9.2 Hz, 2H), 2.52 (t, *J* = 2.5 Hz, 1H); ^{13}C NMR (100 MHz, $CDCl_3$) δ 169.1, 155.1, 132.0, 128.3, 127.2, 124.8, 77.8, 76.6, 75.8, 53.2, 38.5; HRMS (ESI-TOF): Anal. Calcd. For $C_{13}H_{10}{}^{79}BrNNaO_3^+$: 329.9736, $C_{13}H_{10}{}^{81}BrNNaO_3^+$: 331.9716, Found: 329.9721, 331.9710; IR (neat, cm^{-1}): υ 3277, 2977, 2945, 1742, 1219, 1204, 1069, 1021, 895, 884, 821, 638.

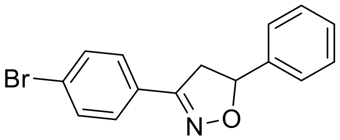

4ak

3-(4-Bromophenyl)-5-phenyl-4,5-dihydroisoxazole (4ak)

ethyl acetate/ petroleum ether =1:30; white solid; 56% yield (84.7 mg); mp: 104-106 ºC; ^{1}H NMR (400 MHz, $CDCl_3$) δ 7.58 – 7.51 (m, 4H), 7.41 – 7.30 (m, 5H), 5.75 (dd, *J* = 11.0, 8.3 Hz, 1H), 3.75 (dd, *J* = 16.6, 11.0 Hz, 1H), 3.31 (dd, *J* = 16.6, 8.3 Hz, 1H); ^{13}C NMR (100 MHz, $CDCl_3$) δ 155.3, 140.6, 131.9, 128.8, 128.4, 128.3, 128.1, 125.8, 124.4, 82.8, 42.9; HRMS (ESI-TOF): Anal. Calcd. For $C_{15}H_{12}{}^{79}BrNNaO^+$: 323.9994, $C_{15}H_{12}{}^{81}BrNNaO^+$: 325.9974, Found: 323.9984, 325.9967; IR (neat, cm^{-1}): υ 3068, 3041, 2968, 2921, 2851, 1587, 1335, 1159, 1007, 901, 830, 757, 697, 671.

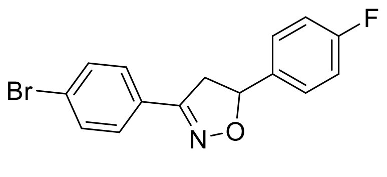

4al

3-(4-Bromophenyl)-5-(4-fluorophenyl)-4,5-dihydroisoxazole (4al)

ethyl acetate/ petroleum ether =1:30; white solid; 52% yield (83.1 mg); mp: 104-105 ºC; ^{1}H NMR (400 MHz, $CDCl_3$) δ 7.55 (s, 4H), 7.40 – 7.32 (m, 2H), 7.10 – 7.02 (m, 2H), 5.73 (dd, *J* = 11.0, 8.3 Hz, 1H), 3.74 (dd, *J* = 16.6, 11.0 Hz, 1H), 3.27 (dd, *J* = 16.6, 8.3 Hz, 1H); ^{13}C NMR (100 MHz, $CDCl_3$) δ 162.6 (d, *J* = 247.0 Hz), 155.3, 136.4 (d, *J* = 3.2 Hz), 132.0, 128.3, 128.1, 127.7 (d, *J* = 8.2 Hz), 124.5, 115.7 (d, *J* = 21.6 Hz), 82.2, 42.9; ^{19}F NMR (376 MHz, $CDCl_3$) δ -113.6; HRMS (ESI-TOF): Anal. Calcd. For $C_{15}H_{11}{}^{79}BrFNNaO^+$: 341.9900, $C_{15}H_{11}{}^{81}BrFNNaO^+$: 343.9880, Found: 341.9896, 343.9894; IR (neat, cm^{-1}): υ 2975, 2927, 1591, 1347, 1193, 1161, 1009, 889, 820.

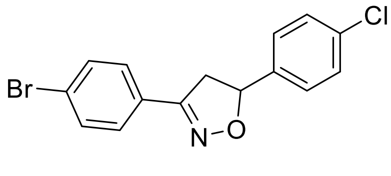

4am

3-(4-Bromophenyl)-5-(4-chlorophenyl)-4,5-dihydroisoxazole (4am)

ethyl acetate/ petroleum ether =1:30; white solid; 46% yield (77.1 mg); mp: 111-113 ºC; ^{1}H NMR (400 MHz, $CDCl_3$) δ 7.53 (s, 4H), 7.37 – 7.28 (m, 4H), 5.71 (dd, *J* = 11.0, 8.2 Hz, 1H), 3.74 (dd, *J* = 16.6, 11.0 Hz, 1H), 3.25 (dd, *J* = 16.6, 8.2 Hz, 1H); ^{13}C NMR (100 MHz, $CDCl_3$) δ 155.2, 139.1, 134.1, 131.9, 128.9, 128.1, 127.2, 124.5, 82.0, 42.9; HRMS (ESI-TOF): Anal. Calcd. For $C_{15}H_{12}{}^{79}BrClNO^+$: 335.9785, $C_{15}H_{12}{}^{81}BrClNO^+$: 337.9765, Found: 335.9819, 337.9777; IR (neat, cm^{-1}): υ 3068, 2959, 2922, 1589, 1488, 1348, 1093, 1009, 908, 837, 823.

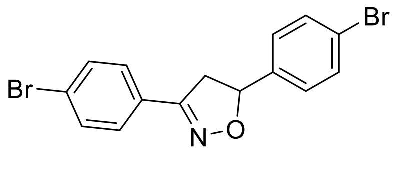

4an

3,5-Bis(4-bromophenyl)-4,5-dihydroisoxazole (4an)

ethyl acetate/ petroleum ether =1:30; white solid; 50% yield (95.8 mg); mp: 130-131 ºC; ^{1}H NMR (400 MHz, $CDCl_3$) δ 7.52 (s, 4H), 7.48 (d, *J* = 8.4 Hz, 2H), 7.24 (d, *J* = 8.4 Hz, 2H), 5.68 (dd, *J* = 11.0, 8.1 Hz, 1H), 3.74 (dd, *J* = 16.7, 11.0 Hz, 1H), 3.24 (dd, *J* = 16.7, 8.1 Hz, 1H); ^{13}C NMR (100 MHz, $CDCl_3$) δ 155.2, 139.6, 131.9, 131.8, 128.09, 128.07, 127.5, 124.5, 122.2, 82.0, 42.8; HRMS (ESI-TOF): Anal. Calcd. For $C_{15}H_{12}{}^{79}Br^{79}Br\ NO^+$: 379.9280, $C_{15}H_{12}{}^{79}Br^{81}Br\ NO^+$: 381.9260, $C_{15}H_{12}{}^{81}Br^{81}Br\ NO^+$: 383.9239, Found: 379.9261, 381.9252, 383.9222; IR (neat, cm^{-1}): υ 3062, 2969, 2917, 1589, 1488, 1348, 1071, 1008, 909, 837, 820.

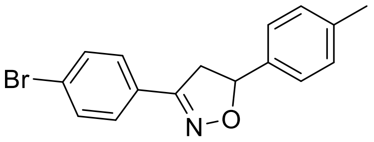

4ao

3-(4-Bromophenyl)-5-(p-tolyl)-4,5-dihydroisoxazole (4ao)

ethyl acetate/ petroleum ether =1:30; white solid; 55% yield (87.3 mg); mp: 126-128 ^{o}C; ^{1}H NMR (400 MHz, $CDCl_3$) δ 7.60 – 7.48 (m, 4H), 7.26 (d, *J* = 8.0 Hz, 2H), 7.18 (d, *J* = 8.0 Hz, 2H), 5.70 (dd, *J* = 11.0, 8.5 Hz, 1H), 3.70 (dd, *J* = 16.7, 11.0 Hz, 1H), 3.28 (dd, *J* = 16.7, 8.5 Hz, 1H), 2.34 (s, 3H); ^{13}C NMR (100 MHz, $CDCl_3$) δ 155.3, 138.1, 137.5, 131.9, 129.4, 128.5, 128.1, 125.8, 124.3, 82.9, 42.7, 21.1; HRMS (ESI-TOF): Anal. Calcd. For $C_{16}H_{14}{}^{79}BrNNaO^+$: 338.0151, $C_{16}H_{14}{}^{81}BrNNaO^+$: 340.0131, Found: 338.0157, 340.0122; IR (neat, cm^{-1}): υ 2977, 2917, 2860, 1587, 1397, 1344, 1070, 1006, 905, 831, 813.

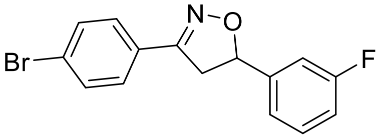

4ap

3-(4-Bromophenyl)-5-(3-fluorophenyl)-4,5-dihydroisoxazole (4ap)

ethyl acetate/ petroleum ether =1:30; white solid; 57% yield (91.2 mg); mp: 85-86 ^{o}C; ^{1}H NMR (400 MHz, $CDCl_3$) δ 7.54 (s, 4H), 7.37 – 7.31 (m, 1H), 7.18 – 7.07 (m, 2H), 7.04 – 6.98 (m, 1H), 5.74 (dd, *J* = 11.1, 8.0 Hz, 1H), 3.77 (dd, *J* = 16.6, 11.1 Hz, 1H), 3.28 (dd, *J* = 16.6, 8.0 Hz, 1H); ^{13}C NMR (100 MHz, $CDCl_3$) δ 163.0 (d, *J* = 246.7 Hz), 155.2, 143.3 (d, *J* = 6.9 Hz), 132.0 , 130.4 (d, *J* = 8.1 Hz), 128.14 , 128.10 , 124.5, 121.3 (d, *J* = 3.0 Hz), 115.2 (d, *J* = 21.2 Hz), 112.8 (d, *J* = 22.4 Hz), 81.9 (d, *J* = 1.8 Hz), 42.9; ^{19}F NMR (376 MHz, $CDCl_3$) δ -112.0; HRMS (ESI-TOF): Anal. Calcd. For $C_{15}H_{11}{}^{79}BrFNNaO^+$: 341.9900, $C_{15}H_{11}{}^{81}BrFNNaO^+$: 343.9880, Found: 341.9906, 343.9900; IR (neat, cm^{-1}): υ 2974, 2925, 2891, 1588, 1485, 1454, 1251, 1143, 1071, 1053, 897, 873, 830, 784, 692.

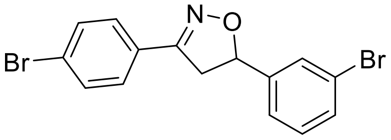

4aq

5-(3-Bromophenyl)-3-(4-bromophenyl)-4,5-dihydroisoxazole (4aq)

ethyl acetate/ petroleum ether =1:30; white solid; 57% yield (109.2 mg); mp: 73-74 ^{o}C; ^{1}H NMR (400 MHz, $CDCl_3$) δ 7.53 (s, 5H), 7.46 – 7.42 (m, 1H), 7.32 – 7.28 (m, 1H), 7.26 – 7.21 (m, 1H), 5.70 (dd, *J* = 11.1, 8.0 Hz, 1H), 3.75 (dd, *J* = 16.7, 11.1 Hz, 1H), 3.27 (dd, *J* = 16.7, 8.0 Hz, 1H); ^{13}C NMR (100 MHz, $CDCl_3$) δ 155.2, 143.0, 131.9, 131.3, 130.3, 128.8, 128.1, 128.0, 124.5, 124.3, 122.8, 81.8, 42.9; HRMS (ESI-TOF): Anal. Calcd. For $C_{15}H_{12}{}^{79}Br^{79}Br\ NO^+$: 379.9280, $C_{15}H_{12}{}^{79}Br^{81}Br\ NO^+$: 381.9260, $C_{15}H_{12}{}^{81}Br^{81}Br\ NO^+$: 383.9239, Found: 379.9266, 381.9236, 383.9210; IR (neat, cm^{-1}): υ 3093, 3071, 2974, 2919, 1589, 1569, 1340, 1161, 1072, 894, 881, 830, 818, 785, 691, 661.

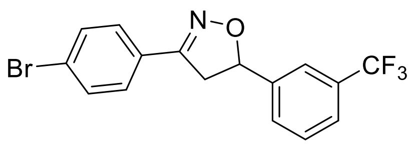

4ar

3-(4-Bromophenyl)-5-(3-(trifluoromethyl)phenyl)-4,5-dihydroisoxazole (4ar)

ethyl acetate/ petroleum ether =1:30; white solid; 56% yield (103.8 mg); mp: 81-83 ºC; ^{1}H NMR (400 MHz, $CDCl_3$) δ 7.65 (s, 1H), 7.60 – 7.47 (m, 7H), 5.80 (dd, *J* = 11.1, 8.1 Hz, 1H), 3.81 (dd, *J* = 16.7, 11.1 Hz, 1H), 3.30 (dd, *J* = 16.7, 8.1 Hz, 1H); ^{13}C NMR (100 MHz, $CDCl_3$) δ 155.2, 141.7, 132.0, 131.1 (q, *J* = 32.5 Hz), 129.3, 129.1, 128.1, 128.0, 125.1 (q, *J* = 3.7 Hz), 124.6, 122.6 (q, *J* = 4.0 Hz), 81.9, 43.0; ^{19}F NMR (376 MHz, $CDCl_3$) δ -62.6; HRMS (ESI-TOF): Anal. Calcd. For $C_{16}H_{11}{}^{79}BrF_3NNaO^+$: 391.9868, $C_{16}H_{11}{}^{81}BrF_3NNaO^+$: 393.9848, Found: 391.9885, 393.9844; IR (neat, cm^{-1}): υ 3062, 3006, 2949, 1324, 1171, 1117, 1072, 905, 895, 837, 802, 701, 661.

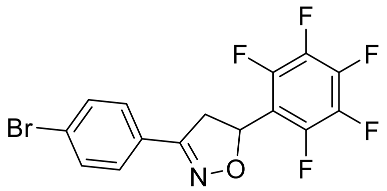

4as

3-(4-Bromophenyl)-5-(perfluorophenyl)-4,5-dihydroisoxazole (4as)

ethyl acetate/ petroleum ether =1:30; white solid; 53% yield (104.1 mg); mp: 127-128 ºC; ^{1}H NMR (400 MHz, $CDCl_3$) δ 7.55 (s, 4H), 6.03 (dd, *J* = 12.0, 8.5 Hz, 1H), 3.78 (dd, *J* = 16.8, 12.0 Hz, 1H), 3.50 (dd, *J* = 16.8, 8.5 Hz, 1H); ^{13}C NMR (100 MHz, $CDCl_3$) δ 154.9, 132.0, 128.2, 127.6, 124.8, 72.4, 40.6; ^{19}F NMR (376 MHz, $CDCl_3$) δ -141.35 – -142.79 (m, 2F), -152.46 – -152.70 (m, 1F), -160.98 – -161.22 (m, 2F); HRMS (ESI-TOF): Anal. Calcd. For $C_{15}H_7{}^{79}BrF_5NNaO^+$: 413.9523, $C_{15}H_7{}^{81}BrF_5NNaO^+$: 415.9503, Found: 413.9508, 415.9506; IR (neat, cm^{-1}): υ 3061, 2977, 2930, 1523, 1503, 1130, 1012, 964, 894, 840, 824.

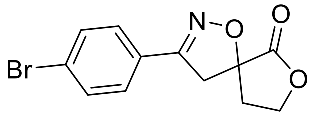

4at

3-(4-Bromophenyl)-1,7-dioxa-2-azaspiro[4.4]non-2-en-6-one (4at)

ethyl acetate/ petroleum ether =1:2; white solid; 85% yield (125.6 mg); mp: 147-148 ºC; ^{1}H NMR (400 MHz, DMSO-d6) δ 7.68 (d, *J* = 8.3 Hz, 2H), 7.62 (d, *J* = 8.3 Hz, 2H), 4.41 (t, *J* = 6.8 Hz, 2H), 3.76 (d, *J* = 17.6 Hz, 1H), 3.70 (d, *J* = 17.6 Hz, 1H), 2.66 – 2.53 (m, 2H); ^{13}C NMR (100 MHz, DMSO-d6) δ 174.5, 156.0, 132.0, 128.8, 127.6, 124.1, 85.1, 66.1, 41.6, 33.9; HRMS (ESI-TOF): Anal. Calcd. For $C_{12}H_{10}{}^{79}BrNNaO_3^+$: 317.9736, $C_{12}H_{10}{}^{81}BrNNaO_3^+$: 319.9716, Found: 317.9756, 319.9706; IR (neat, cm^{-1}): υ 2942, 2927, 1741, 1592, 1350, 1199, 1008, 892, 821.

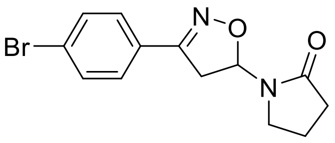

4au

1-(3-(4-Bromophenyl)-4,5-dihydroisoxazol-5-yl)pyrrolidin-2-one (4au)

ethyl acetate/ petroleum ether =4:1; white solid; 44% yield (68.5 mg); mp: 195-196 ºC; ^{1}H NMR (400 MHz, CDCl$_3$) δ 7.53 (s, 4H), 6.63 (dd, *J* = 9.9, 3.5 Hz, 1H), 3.51 (dd, *J* = 17.6, 9.9 Hz, 1H), 3.35 – 3.27 (m, 1H), 3.20 – 3.14 (m, 1H), 3.11 (dd, *J* = 17.6, 3.5 Hz, 1H), 2.42 – 2.36 (m, 2H), 2.08 – 1.92 (m, 2H); ^{13}C NMR (100 MHz, CDCl$_3$) δ 175.3, 154.6, 132.0, 128.1, 127.5, 124.8, 82.2, 41.5, 36.8, 30.9, 17.6; HRMS (ESI-TOF): Anal. Calcd. For $C_{13}H_{13}{}^{79}BrN_2NaO_2{}^+$: 331.0053, $C_{13}H_{13}{}^{81}BrN_2NaO_2{}^+$: 333.0032, Found: 331.0036, 333.0051; IR (neat, cm^{-1}): υ 2980, 2921, 2890, 1683, 1397, 1283, 1261, 1238, 886, 816, 790.

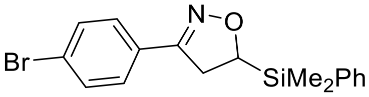

4av

3-(4-Bromophenyl)-5-(dimethyl(phenyl)silyl)-4,5-dihydroisoxazole (4av)

ethyl acetate/ petroleum ether =1:100; white solid; 77% yield (138.4 mg); mp: 92-93 ºC; ^{1}H NMR (400 MHz, CDCl$_3$) δ 7.65 – 7.60 (m, 2H), 7.55 – 7.48 (m, 4H), 7.46 – 7.36 (m, 3H), 4.32 (dd, *J* = 15.6, 11.3 Hz, 1H), 3.39 (dd, *J* = 15.6, 11.3 Hz, 1H), 3.09 (t, *J* = 15.6 Hz, 1H), 0.49 (s, 3H), 0.47 (s, 3H); ^{13}C NMR (100 MHz, CDCl$_3$) δ 155.7, 135.2, 133.9, 131.7, 129.7, 128.5, 128.2, 128.0, 123.9, 74.2, 37.7, -5.1, -5.7; HRMS (ESI-TOF): Anal. Calcd. For $C_{17}H_{18}{}^{79}BrNNaOSi^+$: 382.0233, $C_{17}H_{18}{}^{81}BrNNaOSi^+$: 384.0213, Found: 382.0235, 384.0212; IR (neat, cm^{-1}): υ 3052, 2956, 2902, 1588, 1251, 1115, 896, 821, 779, 736, 697.

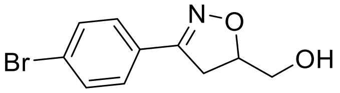

4aw

(3-(4-Bromophenyl)-4,5-dihydroisoxazol-5-yl)methanol (4aw)

ethyl acetate/ petroleum ether =1:1; white solid; 37% yield (47.4 mg); mp: 116-118 ºC; ^{1}H NMR (400 MHz, CDCl$_3$) δ 7.51 (s, 4H), 4.90 – 4.82 (m, 1H), 3.87 (dd, *J* = 12.3, 3.2 Hz, 1H), 3.67 (dd, *J* = 12.3, 4.4 Hz, 1H), 3.34 (dd, *J* = 16.6, 10.7 Hz, 1H), 3.26 (dd, *J* = 16.6, 8.0 Hz, 1H), 2.41 (s, 1H); ^{13}C NMR (100 MHz, CDCl$_3$) δ 156.2, 131.9, 128.2, 128.1, 124.4, 81.5, 63.5, 36.0; HRMS (ESI-TOF): Anal. Calcd. For $C_{10}H_{11}{}^{79}BrNO^+$: 255.9968, $C_{10}H_{11}{}^{81}BrNO^+$: 257.9947, Found: 255.9970, 257.9933; IR (neat, cm^{-1}): υ 3375, 2936, 2922, 2852, 1592, 1399, 1007, 927, 907, 827, 820, 805.

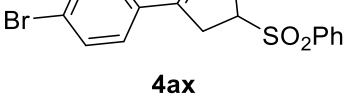

4ax

3-(4-Bromophenyl)-5-(phenylsulfonyl)-4,5-dihydroisoxazole (4ax)

ethyl acetate/ petroleum ether =1:4; white solid; 53% yield (96.6 mg); mp: >200 °C; ^{1}H NMR (400 MHz, $CDCl_3$) δ 7.99 (d, *J* = 7.5 Hz, 2H), 7.71 – 7.65 (m, 1H), 7.61 – 7.50 (m, 4H), 7.46 (d, *J* = 8.5 Hz, 2H), 5.56 (dd, *J* = 10.9, 4.5 Hz, 1H), 4.02 (dd, *J* = 18.3, 4.5 Hz, 1H), 3.77 (dd, *J* = 18.3, 10.9 Hz, 1H); ^{13}C NMR (100 MHz, $CDCl_3$) δ 156.1, 135.1, 134.6, 132.1, 129.7, 129.2, 128.4, 126.2, 125.5, 93.3, 36.5; HRMS (ESI-TOF): Anal. Calcd. For $C_{15}H_{12}{}^{79}BrNNaO_3S^+$: 387.9613, $C_{15}H_{12}{}^{81}BrNNaO_3S^+$: 389.9593, Found: 387.9625, 389.9600; IR (neat, cm^{-1}): υ 3006, 2940, 2895, 1592, 1310, 1089, 1010, 612.

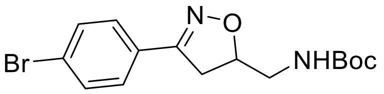

4ay

tert-Butyl ((3-(4-bromophenyl)-4,5-dihydroisoxazol-5-yl)methyl)carbamate (4ay)

ethyl acetate/ petroleum ether =1:5; white solid; 41% yield (73.2 mg); mp: 123-125 °C; ^{1}H NMR (400 MHz, $CDCl_3$) δ 7.50 (s, 4H), 4.98 (s, 1H), 4.89 – 4.80 (m, 1H), 3.50 – 3.26 (m, 3H), 3.11 (dd, *J* = 16.9, 7.5 Hz, 1H), 1.38 (s, 9H); ^{13}C NMR (100 MHz, $CDCl_3$) δ 156.2, 156.1, 131.9, 128.2, 128.1, 124.4, 80.4, 79.7, 43.5, 37.1, 28.2; HRMS (ESI-TOF): Anal. Calcd. For $C_{15}H_{19}{}^{79}BrN_2NaO_3^+$: 377.0471, $C_{15}H_{19}{}^{81}BrN_2NaO_3^+$: 379.0451, Found: 377.0466, 379.0445; IR (neat, cm^{-1}): υ 3374, 2978, 2941, 2872, 1708, 1512, 1492, 1245, 1157, 1009, 917, 885, 819.

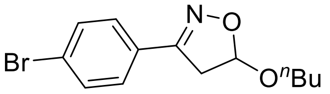

4az

3-(4-Bromophenyl)-5-butoxy-4,5-dihydroisoxazole (4az)

ethyl acetate/ petroleum ether =1:30; white solid; 54% yield (80.1 mg); mp: 58-59 °C; ^{1}H NMR (400 MHz, $CDCl_3$) δ 7.59 – 7.49 (m, 4H), 5.67 (dd, *J* = 6.7, 1.7 Hz, 1H), 3.85 (dt, *J* = 9.4, 6.6 Hz, 1H), 3.53 (dt, *J* = 9.4, 6.7 Hz, 1H), 3.36 (dd, *J* = 17.3, 6.7 Hz, 1H), 3.16 (dd, *J* = 17.3, 1.7 Hz, 1H), 1.60 – 1.51 (m, 2H), 1.41 – 1.30 (m, 2H), 0.91 (t, *J* = 7.3 Hz, 3H); ^{13}C NMR (100 MHz, $CDCl_3$) δ 156.1, 131.9, 128.22, 128.16, 124.5, 103.4, 68.2, 41.2, 31.5, 19.1, 13.8; HRMS (ESI-TOF): Anal. Calcd. For $C_{13}H_{17}{}^{79}BrNO_2^+$: 298.0437, $C_{13}H_{17}{}^{81}BrNO_2^+$: 300.0417, Found: 298.0426, 300.0415; IR (neat, cm^{-1}): υ 2957, 2930, 2871, 1591, 1350, 1189, 1093, 1071, 1009, 886, 841.

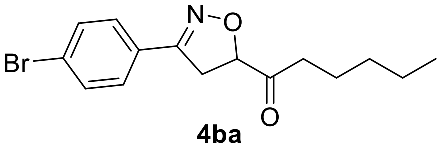

4ba

1-(3-(4-Bromophenyl)-4,5-dihydroisoxazol-5-yl)hexan-1-one (4ba)

ethyl acetate/ petroleum ether =1:10; white solid; 39% yield (63.2 mg); mp: 68-70 °C; ^{1}H NMR (400 MHz, $CDCl_3$) δ 7.54 (s, 4H), 5.05 (dd, *J* = 11.9, 6.3 Hz, 1H), 3.63 (dd, *J* = 17.0, 6.3 Hz, 1H), 3.45 (dd, *J* = 17.0, 11.9 Hz, 1H), 2.71 (t, *J* = 7.4 Hz, 2H), 1.66 – 1.55 (m, 2H), 1.36 – 1.24 (m, 4H), 0.88 (t, *J* = 6.8 Hz, 3H); ^{13}C NMR (100 MHz, $CDCl_3$) δ 209.2, 155.8, 132.0, 128.3, 124.9, 84.4, 38.9, 36.9, 31.3, 22.7, 22.4, 13.9; HRMS (ESI-TOF): Anal. Calcd. For $C_{15}H_{18}{}^{79}BrNNaO_2^+$: 346.0413, $C_{15}H_{18}{}^{81}BrNNaO_2^+$: 348.0393, Found: 346.0391, 348.0421; IR (neat, cm^{-1}): υ 2949, 2924, 2867, 2855, 1707, 1591, 896, 888, 823.

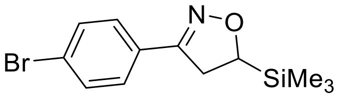

4ca

3-(4-Bromophenyl)-5-(trimethylsilyl)-4,5-dihydroisoxazole (4ca)

ethyl acetate/ petroleum ether =1:100; white solid; 96% yield (143.4 mg); mp: 77-79 ºC; ^{1}H NMR (400 MHz, $CDCl_3$) δ 7.59 – 7.48 (m, 4H), 4.10 (dd, *J* = 15.6, 11.4 Hz, 1H), 3.41 (dd, *J* = 15.6, 11.4 Hz, 1H), 3.06 (t, *J* = 15.6 Hz, 1H), 0.16 (s, 9H); ^{13}C NMR (100 MHz, $CDCl_3$) δ 155.6, 131.8, 128.8, 128.2, 124.0, 74.7, 37.5, -3.9; HRMS (ESI-TOF): Anal. Calcd. For $C_{12}H_{16}{}^{79}BrNNaOSi^+$: 320.0077, $C_{12}H_{16}{}^{81}BrNNaOSi^+$: 322.0056, Found: 320.0066, 322.0051; IR (neat, cm^{-1}): υ 2958, 2924, 1399, 1247, 1195, 1071, 1008, 889, 863, 823.

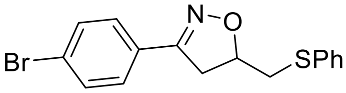

4da

3-(4-Bromophenyl)-5-((phenylthio)methyl)-4,5-dihydroisoxazole (4da)

ethyl acetate/ petroleum ether =1:30; white solid; 49% yield (85.9 mg); mp: >200 ºC; ^{1}H NMR (400 MHz, $CDCl_3$) δ 7.56 – 7.47 (m, 4H), 7.44 – 7.38 (m, 2H), 7.34 – 7.28 (m, 2H), 7.27 – 7.20 (m, 1H), 4.88 (dddd, *J* = 11.0, 8.7, 6.7, 4.5 Hz, 1H), 3.45 – 3.33 (m, 2H), 3.22 (dd, *J* = 16.8, 6.7 Hz, 1H), 2.99 (dd, *J* = 13.6, 8.7 Hz, 1H); ^{13}C NMR (100 MHz, $CDCl_3$) δ 155.4, 134.6, 131.9, 130.1, 129.1, 128.3, 128.1, 126.8, 124.4, 79.8, 39.2, 37.7; HRMS (ESI-TOF): Anal. Calcd. For $C_{16}H_{14}{}^{79}BrNNaOS^+$: 369.9872, $C_{16}H_{14}{}^{81}BrNNaOS^+$: 371.9851, Found: 369.9858, 371.9852; IR (neat, cm^{-1}): υ 3058, 3020, 2924, 1583, 1436, 1071, 1006, 895, 822, 735, 689.

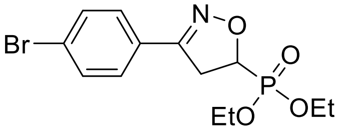

4ea

Diethyl (3-(4-bromophenyl)-4,5-dihydroisoxazol-5-yl)phosphonate (4ea)

ethyl acetate/ petroleum ether =1:1.5; white solid; 66% yield (119.9 mg); mp: 65-67 ºC; ^{1}H NMR (400 MHz, $CDCl_3$) δ 7.51 (s, 4H), 4.89 – 4.82 (m, 1H), 4.27 – 4.15 (m, 4H), 3.68 – 3.52 (m, 2H), 1.33 (t, *J* = 5.8 Hz, 3H), 1.30 (t, *J* = 7.0 Hz, 3H); ^{13}C NMR (100 MHz, $CDCl_3$) δ 155.4 (d, *J* = 6.3 Hz), 131.9, 128.2, 127.4, 124.8, 75.3 (d, *J* = 168.9 Hz), 63.5 (d, *J* = 7.0 Hz), 63.1 (d, *J* = 6.8 Hz), 37.4, 16.4 (d, *J* = 5.6 Hz); HRMS (ESI-TOF): Anal. Calcd. For $C_{13}H_{17}{}^{79}BrNNaO_4P^+$: 383.9971, $C_{13}H_{17}{}^{81}BrNNaO_4P^+$: 385.9950, Found: 383.9956, 385.9958; IR (neat, cm^{-1}): υ 2949, 2917, 1617, 1540, 1372, 1311, 1124, 1031, 911, 802, 694.

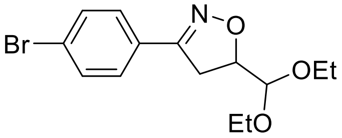

4fa

3-(4-Bromophenyl)-5-(diethoxymethyl)-4,5-dihydroisoxazole (4fa)

ethyl acetate/ petroleum ether =1:20; white solid; 34% yield (55.9 mg); mp: 64-65 ^oC; ^1H NMR (400 MHz, $CDCl_3$) δ 7.52 (s, 4H), 4.76 (ddd, *J* = 11.1, 7.1, 4.4 Hz, 1H), 4.54 (d, *J* = 4.4 Hz, 1H), 3.82 – 3.69 (m, 2H), 3.69 – 3.56 (m, 2H), 3.39 (dd, *J* = 16.9, 7.1 Hz, 1H), 3.28 (dd, *J* = 16.9, 11.1 Hz, 1H), 1.25 (t, *J* = 7.0 Hz, 3H), 1.16 (t, *J* = 7.0 Hz, 3H); ^{13}C NMR (100 MHz, $CDCl_3$) δ 155.9, 131.8, 128.4, 128.1, 124.2, 102.1, 81.6, 64.5, 63.6, 35.5, 15.3, 15.2; HRMS (ESI-TOF): Anal. Calcd. For $C_{14}H_{18}{}^{79}BrNNaO_3{}^+$: 350.0362, $C_{14}H_{18}{}^{81}BrNNaO_3{}^+$: 352.0342, Found: 350.0352, 352.0329; IR (neat, cm^{-1}): υ 2976, 2929, 2882, 1592, 1399, 1349, 1120, 1061, 1009, 899, 823.

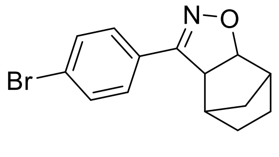

4ga

3-(4-bromophenyl)-3a,4,5,6,7,7a-hexahydro-4,7-methanobenzo[d]isoxazole (4ga)

ethyl acetate/ petroleum ether =1:100; white solid; 69% yield (100.9 mg); mp: 97-98 ^oC; ^1H NMR (400 MHz, $CDCl_3$) δ 7.56 (d, *J* = 8.4 Hz, 2H), 7.49 (d, *J* = 8.4 Hz, 2H), 4.62 (d, *J* = 8.3 Hz, 1H), 3.43 (d, *J* = 8.3 Hz, 1H), 2.62 – 2.58 (m, 1H), 2.50 – 2.43 (m, 1H), 1.62 – 1.51 (m, 2H), 1.50 – 1.44 (m, 1H), 1.39 – 1.29 (m, 1H), 1.23 – 1.11 (m, 2H); ^{13}C NMR (100 MHz, $CDCl_3$) δ 156.0, 131.8, 128.3, 128.2, 123.8, 88.1, 56.7, 42.9, 39.1, 32.2, 27.3, 22.6; HRMS (ESI-TOF): Anal. Calcd. For $C_{14}H_{14}{}^{79}BrNNaO^+$: 314.0151, $C_{14}H_{14}{}^{81}BrNNaO^+$: 316.0131, Found: 314.0153, 316.0113; IR (neat, cm^{-1}): υ 2960, 2948, 2872, 1588, 1399, 1068, 1007, 881, 814.

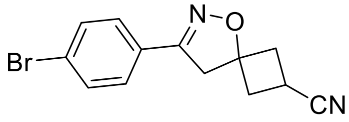

4ha

7-(4-Bromophenyl)-5-oxa-6-azaspiro[3.4]oct-6-ene-2-carbonitrile (4ha)

ethyl acetate/ petroleum ether =1:6; white solid; 40% yield (58.3 mg); mp: 135-137 ^oC; ^1H NMR (400 MHz, $CDCl_3$) δ 7.54 (d, *J* = 8.7 Hz, 2H), 7.51 (d, *J* = 8.7 Hz, 2H), 3.54 (s, 2H), 3.23 – 3.12 (m, 1H), 2.99 (dd, *J* = 13.3, 9.8 Hz, 2H), 2.67 (dd, *J* = 13.3, 5.1 Hz, 2H); ^{13}C NMR (100 MHz, $CDCl_3$) δ 156.0, 132.0, 128.0, 127.9, 124.7, 122.1, 84.5, 45.8, 40.8, 15.2; HRMS (ESI-TOF): Anal. Calcd. For $C_{13}H_{11}{}^{79}BrN_2NaO^+$: 312.9947, $C_{13}H_{11}{}^{81}BrN_2NaO^+$: 314.9927, Found: 312.9927, 314.9941; IR (neat, cm^{-1}): υ 2976, 2923, 2875, 2852, 2239, 1699, 1400, 1363, 1160, 1084, 1008, 894, 819.

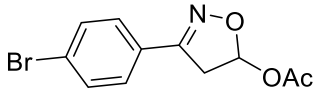

4ia

3-(4-Bromophenyl)-4,5-dihydroisoxazol-5-yl acetate (4ia)

ethyl acetate/ petroleum ether =1:4; white solid; 38% yield (54.0 mg); mp: 78-80 ^oC; ^1H NMR (400 MHz, $CDCl_3$) δ 7.55 (s, 4H), 6.82 (dd, *J* = 6.9, 1.4 Hz, 1H), 3.58 (dd, *J* = 17.8, 6.9 Hz, 1H), 3.31 (dd, *J* = 17.8, 1.4 Hz, 1H), 2.07 (s, 3H); ^{13}C NMR (100 MHz, $CDCl_3$) δ 169.6, 156.1, 132.1, 128.4, 127.2, 125.2, 95.9,

41.0, 21.0; HRMS (ESI-TOF): Anal. Calcd. For $C_{11}H_{10}{}^{79}BrNNaO_3{}^+$: 305.9736, $C_{11}H_{10}{}^{81}BrNNaO_3{}^+$: 307.9716, Found: 305.9746, 307.9719; IR (neat, cm^{-1}): υ 2956, 2919, 2851, 1750, 1363, 1218, 1172, 1063, 1029, 949, 845, 818.

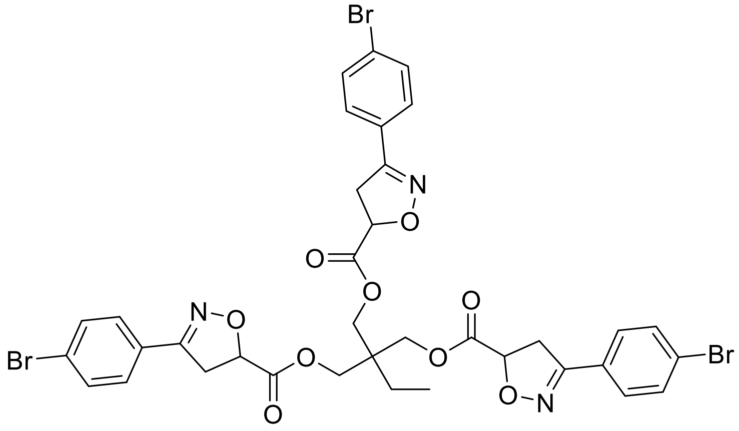

4ja

2-(((3-(4-Bromophenyl)-4,5-dihydroisoxazole-5-carbonyl)oxy)methyl)-2-ethylpropane-1,3-diyl bis(3-(4-bromophenyl)-4,5-dihydroisoxazole-5-carboxylate) (4ja)

ethyl acetate/ petroleum ether =1:1; white solid; 33% yield (149.0 mg); mp: 146-148 ºC; ^{1}H NMR (400 MHz, $CDCl_3$) δ 7.54 – 7.41 (m, 12H), 5.20 – 5.06 (m, 3H), 4.23 – 4.09 (m, 6H), 3.66 – 3.47 (m, 6H), 1.51 (q, *J* = 7.3 Hz, 2H), 0.87 (t, *J* = 7.3 Hz, 3H); ^{13}C NMR (100 MHz, $CDCl_3$) δ 169.4, 155.3, 131.9, 128.2, 127.2, 124.8, 78.0, 64.9, 41.1, 38.3, 23.1, 7.2; HRMS (ESI-TOF): Anal. Calcd. For $C_{36}H_{32}{}^{79}Br^{79}Br^{81}BrN_3NaO_9{}^+$: 911.9560, $C_{36}H_{32}{}^{79}Br^{81}Br^{81}BrN_3NaO_9{}^+$: 913.9540, Found: 911.9585, 913.9638; IR (neat, cm^{-1}): υ 2965, 2926, 1740, 1591, 1347, 1193, 1161, 1008, 891, 864, 820.

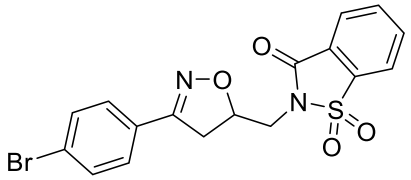

4ka

2-((3-(4-Bromophenyl)-4,5-dihydroisoxazol-5-yl)methyl)benzo[d]isothiazol-3(2H)-one 1,1-dioxide (4ka)

ethyl acetate/ petroleum ether =1:2; white solid; 44% yield (92.6 mg); mp: 153-154 ºC; ^{1}H NMR (400 MHz, DMSO-d6) δ 8.32 (d, *J* = 7.6 Hz, 1H), 8.12 (d, *J* = 7.5 Hz, 1H), 8.09 – 8.04 (m, 1H), 8.03 – 7.97 (m, 1H), 7.65 (d, *J* = 8.6 Hz, 2H), 7.60 (d, *J* = 8.6 Hz, 2H), 5.16 – 5.06 (m, 1H), 4.01 (dd, *J* = 15.2, 7.4 Hz, 1H), 3.88 (dd, *J* = 15.2, 4.8 Hz, 1H), 3.60 (dd, *J* = 17.2, 10.6 Hz, 1H), 3.39 – 3,31 (m, 1H); ^{13}C NMR (100 MHz, DMSO-d6) δ 159.0, 156.2, 136.7, 136.0, 135.4, 131.9, 128.7, 128.4, 126.2, 125.3, 123.6, 121.7, 77.8, 41.7, 37.9; HRMS (ESI-TOF): Anal. Calcd. For $C_{17}H_{13}{}^{79}BrN_2NaO_4S^+$: 442.9672, $C_{17}H_{13}{}^{81}BrN_2NaO_4S^+$: 444.9651, Found: 442.9660, 444.9621; IR (neat, cm^{-1}): υ 3088, 3027, 1732, 1326, 1265, 1179, 1160, 906, 869, 816, 754, 676.

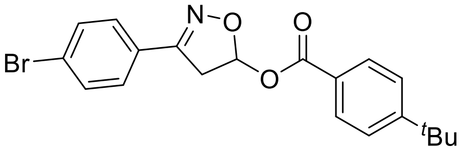

4la

3-(4-Bromophenyl)-4,5-dihydroisoxazol-5-yl 4-(tert-butyl)benzoate (4la)

ethyl acetate/ petroleum ether =1:8; white solid; 47% yield (95.2 mg); mp: 130-131 ºC; ^{1}H NMR (400 MHz, $CDCl_3$) δ 7.95 (d, *J* = 8.4 Hz, 2H), 7.63 – 7.56 (m, 4H), 7.43 (d, *J* = 8.4 Hz, 2H), 7.08 (d, *J* = 6.8 Hz, 1H), 3.70 (dd, *J* = 17.7, 6.8 Hz, 1H), 3.47 (d, *J* = 17.7 Hz, 1H), 1.32 (s, 9H); ^{13}C NMR (100 MHz, $CDCl_3$) δ 165.2, 157.5, 156.2, 132.2, 129.8, 128.5, 127.3, 126.2, 125.4, 125.2, 96.7, 41.4, 35.1, 31.0; HRMS (EI-TOF): Anal. Calcd. For $C_{20}H_{20}{}^{79}BrNO_3$: 401.0627, $C_{20}H_{20}{}^{81}BrNO_3$: 403.0606, Found: 401.0621, 403.0600; IR (neat, cm^{-1}): υ 2964, 2924, 2871, 1723, 1265, 1087, 1073, 1009, 948, 845, 821, 773, 706.

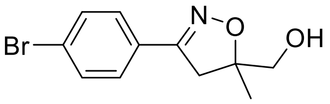

4ma

(3-(4-Bromophenyl)-5-methyl-4,5-dihydroisoxazol-5-yl)methanol (4ma)

ethyl acetate/ petroleum ether =1:3; colorless liquid; 42% yield (56.6 mg); ^{1}H NMR (400 MHz, $CDCl_3$) δ 7.54 – 7.47 (m, 4H), 3.74 (d, *J* = 12.0 Hz, 1H), 3.57 (d, *J* = 12.0 Hz, 1H), 3.47 (d, *J* = 16.6 Hz, 1H), 2.97 (d, *J* = 16.6 Hz, 1H), 2.36 (s, 1H), 1.42 (s, 3H); ^{13}C NMR (100 MHz, $CDCl_3$) δ 156.1, 131.8, 128.7, 128.0, 124.2, 87.9, 67.2, 41.7, 22.6; HRMS (ESI-TOF): Anal. Calcd. For $C_{11}H_{12}{}^{79}BrNNaO_2{}^{+}$: 291.9944, $C_{11}H_{12}{}^{81}BrNNaO_2{}^{+}$: 293.9923, Found: 291.9952, 293.9929; IR (neat, cm^{-1}): υ 3396, 2975, 2928, 2869, 1591, 1490, 1399, 1354, 1055, 1009, 909, 821, 795.

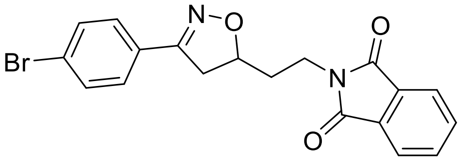

4na

2-(2-(3-(4-Bromophenyl)-4,5-dihydroisoxazol-5-yl)ethyl)isoindoline-1,3-dione (4na)

ethyl acetate/ petroleum ether =1:4; white solid; 40% yield (79.7 mg); mp: 149-151 ºC; ^{1}H NMR (400 MHz, $CDCl_3$) δ 7.84 – 7.79 (m, 2H), 7.73 – 7.66 (m, 2H), 7.48 (s, 4H), 4.83 – 4.74 (m, 1H), 3.93 – 3.77 (m, 2H), 3.45 (dd, *J* = 16.6, 10.5 Hz, 1H), 3.01 (dd, *J* = 16.6, 8.1 Hz, 1H), 2.24 – 2.13 (m, 1H), 2.04 – 1.95 (m, 1H); ^{13}C NMR (100 MHz, $CDCl_3$) δ 168.1, 155.4, 133.9, 131.9, 131.8, 128.4, 128.0, 124.2, 123.2, 79.4, 39.9, 34.7, 33.9; HRMS (ESI-TOF): Anal. Calcd. For $C_{19}H_{15}{}^{79}BrN_2NaO_3{}^{+}$: 421.0158, $C_{19}H_{15}{}^{81}BrN_2NaO_3{}^{+}$: 423.0138, Found: 421.0133, 423.0146; IR (neat, cm^{-1}): υ 3066, 2930, 2847, 1703, 1395, 1007, 906, 814, 716, 706.

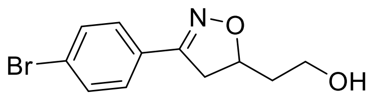

4oa

2-(3-(4-Bromophenyl)-4,5-dihydroisoxazol-5-yl)ethan-1-ol (4oa)

ethyl acetate/ petroleum ether =1:1; white solid; 36% yield (49.5 mg); mp: 99-101 ºC; ^{1}H NMR (400 MHz, $CDCl_3$) δ 7.51 (s, 4H), 4.97 – 4.88 (m, 1H), 3.84 (t, *J* = 5.9 Hz, 2H), 3.43 (dd, *J* = 16.5, 10.4 Hz, 1H), 3.03 (dd, *J* = 16.5, 8.0 Hz, 1H), 2.22 (s, 1H), 2.08 – 1.85 (m, 2H); ^{13}C NMR (100 MHz, $CDCl_3$) δ 156.0, 131.9, 128.4, 128.0, 124.3, 79.6, 59.6, 40.2, 37.7; HRMS (ESI-TOF): Anal. Calcd. For $C_{11}H_{12}{}^{79}BrNNaO_2{}^{+}$: 291.9944, $C_{11}H_{12}{}^{81}BrNNaO_2{}^{+}$: 293.9923, Found: 291.9949, 293.9922; IR (neat, cm^{-1}): υ 3293, 2937, 2880, 1591, 1399, 1348, 1041, 1033, 899, 824, 795.

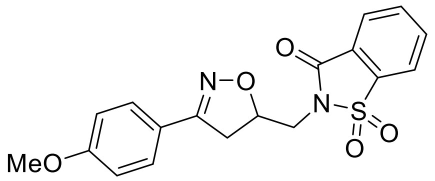

5

2-((3-(4-Methoxyphenyl)-4,5-dihydroisoxazol-5-yl)methyl)benzo[d]isothiazol-3(2H)-one 1,1-dioxide (5)

ethyl acetate/ petroleum ether =1:1.5; light yellow solid; 60% yield (111.7 mg); mp: 147-148 ºC; ^{1}H NMR (400 MHz, DMSO-d6) δ 8.32 (d, *J* = 7.6 Hz, 1H), 8.16 – 7.95 (m, 3H), 7.60 (d, *J* = 8.8 Hz, 2H), 7.00 (d, *J* = 8.8 Hz, 2H), 5.10 – 5.01 (m, 1H), 3.97 (dd, *J* = 15.1, 7.4 Hz, 1H), 3.89 – 3.76 (m, 4H), 3.56 (dd, *J* = 17.1, 10.5 Hz, 1H), 3.37 – 3.29 (m, 1H); ^{13}C NMR (100 MHz, DMSO-d6) δ 160.8, 159.0, 156.2, 136.8, 136.0, 135.4, 128.3, 126.3, 125.3, 121.7, 121.6, 114.3, 77.1, 55.4, 41.7, 38.3; HRMS (ESI-TOF): Anal. Calcd. For $C_{18}H_{17}N_2O_5S^{+}$: 373.0853, Found: 373.0859; IR (neat, cm^{-1}): υ 3069, 2963, 2920, 2848, 1739, 1321, 1300, 1248, 1178, 1162, 1018, 839, 747, 671.

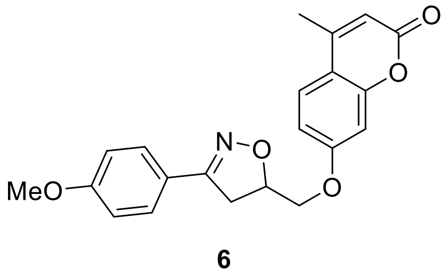

6

7-((3-(4-Methoxyphenyl)-4,5-dihydroisoxazol-5-yl)methoxy)-4-methyl-2H-chromen-2-one (6)

ethyl acetate/ petroleum ether =1:1; light yellow solid; 52% yield (94.8 mg); mp: 141-143 ºC; ^{1}H NMR (400 MHz, $CDCl_3$) δ 7.60 (d, *J* = 8.8 Hz, 2H), 7.45 (d, *J* = 8.8 Hz, 1H), 6.89 (d, *J* = 8.8 Hz, 2H), 6.84 (dd, *J* = 8.8, 2.5 Hz, 1H), 6.76 (d, *J* = 2.5 Hz, 1H), 6.08 (s, 1H), 5.12 – 5.04 (m, 1H), 4.17 (dd, *J* = 10.0, 5.3 Hz, 1H), 4.11 (dd, *J* = 10.0, 4.9 Hz, 1H), 3.81 (s, 3H), 3.50 (dd, *J* = 16.7, 10.7 Hz, 1H), 3.32 (dd, *J* = 16.7, 7.0 Hz, 1H), 2.35 (s, 3H); ^{13}C NMR (100 MHz, $CDCl_3$) δ 161.2, 161.1, 161.0, 155.9, 154.9, 152.4, 128.2, 125.5, 121.5, 114.0, 113.9, 112.3, 112.1, 101.6, 78.1, 69.0, 55.3, 37.6, 18.5; HRMS (ESI-TOF): Anal. Calcd. For $C_{21}H_{20}NO_5{}^{+}$: 366.1336, Found: 366.1354; IR (neat, cm^{-1}): υ 2961, 2932, 1731, 1607, 1251, 1155, 1071, 891, 827.

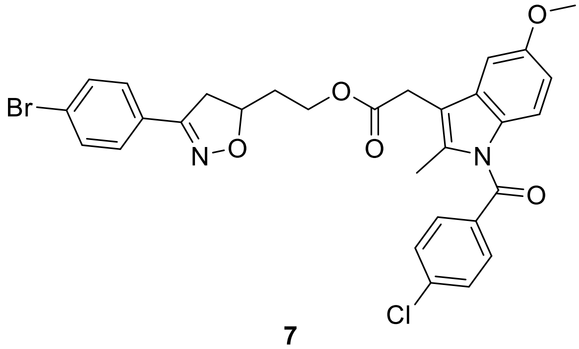

7

2-(3-(4-Bromophenyl)-4,5-dihydroisoxazol-5-yl)ethyl 2-(1-(4-chlorobenzoyl)-5-methoxy-2-methyl-1H-indol-3-yl)acetate (7)

ethyl acetate/ petroleum ether =1:3; yellow solid; 45% yield (137.8 mg); mp: 81-83 °C; ^{1}H NMR (400 MHz, $CDCl_3$) δ 7.63 (d, *J* = 8.3 Hz, 2H), 7.49 (d, *J* = 8.3 Hz, 2H), 7.46 – 7.41 (m, 4H), 6.96 (d, *J* = 2.4 Hz, 1H), 6.87 (d, *J* = 9.0 Hz, 1H), 6.66 (dd, *J* = 9.0, 2.4 Hz, 1H), 4.76 – 4.67 (m, 1H), 4.34 – 4.22 (m, 2H), 3.81 (s, 3H), 3.67 (s, 2H), 3.26 (dd, *J* = 16.5, 10.4 Hz, 1H), 2.88 (dd, *J* = 16.5, 8.0 Hz, 1H), 2.37 (s, 3H), 2.14 – 2.01 (m, 1H), 2.01 – 1.88 (m, 1H); ^{13}C NMR (100 MHz, $CDCl_3$) δ 170.6, 168.1, 155.9, 155.6, 139.2, 135.8, 133.7, 131.8, 131.0, 130.7, 130.5, 129.0, 128.3, 127.9, 124.2, 114.9, 112.3, 111.4, 101.2, 78.4, 61.5, 55.6, 39.7, 34.1, 30.2, 13.3; HRMS (EI-TOF): Anal. Calcd. For $C_{30}H_{26}{}^{79}BrClN_2O_5$: 608.0714, $C_{30}H_{26}{}^{81}BrClN_2O_5$: 610.0693, Found: 608.0717, 610.0674; IR (neat, cm^{-1}): υ 2978, 2957, 2929, 2832, 1733, 1679, 1591, 1477, 1355, 1315, 1219, 1068, 826, 751.

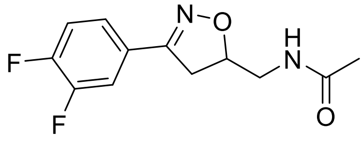

8

***N*-((3-(3,4-Difluorophenyl)-4,5-dihydroisoxazol-5-yl)methyl)acetamide (8)**

ethyl acetate/ petroleum ether =7:1; light yellow solid; 36% yield (46.1 mg); mp: 121-122 °C; ^{1}H NMR (400 MHz, $CDCl_3$) δ 7.57 – 7.45 (m, 1H), 7.34 – 7.28 (m, 1H), 7.21 – 7.13 (m, 1H), 6.14 (s, 1H), 4.91 – 4.83 (m, 1H), 3.63 – 3.46 (m, 2H), 3.34 (dd, *J* = 16.9, 10.7 Hz, 1H), 3.07 (dd, *J* = 16.9, 7.5 Hz, 1H), 1.97 (s, 3H); ^{13}C NMR (100 MHz, $CDCl_3$) δ 170.8, 155.4, 151.5 (dd, *J* = 253.5, 12.6 Hz), 150.3 (dd, *J* = 249.8, 13.2 Hz), 126.2 (dd, *J* = 6.3, 4.0 Hz), 123.2 (dd, *J* = 6.7, 3.7 Hz), 117.7 (d, *J* = 17.9 Hz), 115.6 (d, *J* = 18.8 Hz), 80.3, 42.2, 37.3, 23.1; ^{19}F NMR (376 MHz, $CDCl_3$) δ -134.0 (d, *J* = 22.0 Hz, 1F), -136.2 (d, *J* = 22.0 Hz, 1F); HRMS (ESI-TOF): Anal. Calcd. For $C_{12}H_{12}F_2N_2NaO_2^+$: 277.0759, Found: 277.0756; IR (neat, cm^{-1}): υ 3294, 2988, 2942, 2926, 1738, 1651, 1225, 1192, 1026, 1009, 822.

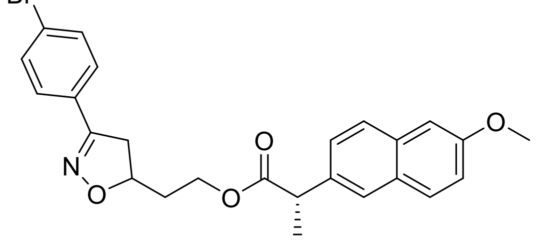

9

2-(3-(4-Bromophenyl)-4,5-dihydroisoxazol-5-yl)ethyl (2S)-2-(6-methoxynaphthalen-2-yl)propanoate (9)

ethyl acetate/ petroleum ether =1:5; white solid; 46% yield (110.6 mg); mp: 113-115 °C; ^{1}H NMR (400 MHz, $CDCl_3$) δ 7.81 – 7.71 (m, 3H), 7.54 – 7.42 (m, 3H), 7.40 – 7.31 (m, 2H), 7.25 – 7.19 (m, 1H), 7.18 – 7.13 (m, 1H), 4.75 – 4.61 (m, 1H), 4.43 – 4.23 (m, 2H), 4.01 – 3.85 (m, 4H), 3.14 – 3.03 (m, 1H), 2.84 – 2.73 (m, 1H), 2.19 – 2.05 (m, 1H), 2.03 – 1.89 (m, 1H), 1.68 – 1.62 (m, 3H); ^{13}C NMR (100 MHz, $CDCl_3$) δ 174.4, 174.3, 157.61, 157.59, 155.66, 155.64, 135.54, 135.49, 133.6, 131.7, 129.13, 129.12, 128.78, 128.77, 128.31, 128.28, 127.84, 127.83, 127.15, 127.12, 126.1, 126.0, 125.9, 125.8, 124.07, 124.06, 119.06, 119.04, 105.54, 105.50, 78.9, 78.6, 61.3, 61.2, 55.20, 55.19, 45.4, 45.3, 39.53, 39.50, 33.89, 33.87, 18.5, 18.3; HRMS (EI-TOF): Anal. Calcd. For $C_{25}H_{24}{}^{79}BrNO_4$: 481.0889, $C_{25}H_{24}{}^{81}BrNO_4$: 483.0868, Found: 481.0893, 483.0891; IR (neat, cm^{-1}): υ 2960, 2937, 1728, 1604, 1176, 1159, 1026, 858, 823, 813.

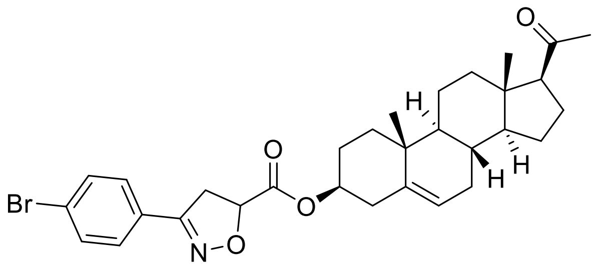

10

(3S,8S,9S,10R,13S,14S,17S)-17-Acetyl-10,13-dimethyl-2,3,4,7,8,9,10,11,12,13,14,15,16,17-tetradecahydro-1H-cyclopenta[a]phenanthren-3-yl 3-(4-bromophenyl)-4,5-dihydroisoxazole-5-carboxylate (10)

ethyl acetate/ petroleum ether =1:5; white solid; 62% yield (176.5 mg); mp: 180-182 °C; ^{1}H NMR (400 MHz, $CDCl_3$) δ 7.48 (s, 4H), 5.35 – 5.30 (m, 1H), 5.10 (t, *J* = 9.3 Hz, 1H), 4.71 – 4.60 (m, 1H), 3.56 (d, *J* = 9.3 Hz, 2H), 2.48 (t, *J* = 8.7 Hz, 1H), 2.36 – 2.29 (m, 2H), 2.25 – 1.84 (m, 8H), 1.68 – 1.51 (m, 5H), 1.46 – 1.38 (m, 3H), 1.20 – 1.08 (m, 3H), 1.00 – 0.92 (m, 4H), 0.57 (s, 3H); ^{13}C NMR (100 MHz, $CDCl_3$) δ 209.2, 169.1, 155.0, 139.0, 131.8, 128.2, 127.4, 124.5, 122.5, 78.2, 75.5, 63.4, 56.6, 49.6, 43.7, 38.5, 38.4, 37.64, 37.60, 36.7, 36.4, 31.6, 31.3, 27.3, 24.3, 22.6, 20.8, 19.1, 13.0; IR (neat, cm^{-1}): υ 2943, 2931, 2853, 1733, 1702, 1508, 1214, 1169, 1155, 1010, 904, 832.

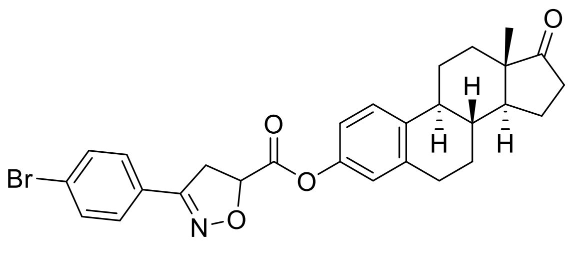

11

(8R,9S,13S,14S)-13-Methyl-17-oxo-7,8,9,11,12,13,14,15,16,17-decahydro-6H-cyclopenta[a]phenanthren-3-yl 3-(4-bromophenyl)-4,5-dihydroisoxazole-5-carboxylate (11)

ethyl acetate/ petroleum ether =1:3; light yellow solid; 71% yield (185.0 mg); mp: 161-162 °C; ^{1}H NMR (400 MHz, $CDCl_3$) δ 7.57 – 7.50 (m, 4H), 7.28 (s, 1H), 6.95 – 6.81 (m, 2H), 5.39 (dd, *J* = 11.2, 7.2 Hz,

1H), 3.83 – 3.63 (m, 2H), 2.92 – 2.84 (m, 2H), 2.54 – 2.45 (m, 1H), 2.41 – 2.34 (m, 1H), 2.29 – 2.20 (m, 1H), 2.17 – 1.94 (m, 4H), 1.66 – 1.42 (m, 6H), 0.89 (s, 3H); ^{13}C NMR (100 MHz, $CDCl_3$) δ 220.5, 168.5, 155.1, 147.9, 138.0, 137.8, 131.8, 128.2, 127.2, 126.3, 124.7, 120.9, 118.1, 78.1, 50.1, 47.7, 43.9, 38.5, 37.7, 35.6, 31.3, 29.1, 26.0, 25.5, 21.4, 13.6; HRMS (ESI-TOF): Anal. Calcd. For $C_{28}H_{29}{}^{79}BrNO_4^+$: 522.1274, $C_{28}H_{29}{}^{81}BrNO_4^+$: 524.1254, Found: 522.1256, 524.1250; IR (neat, cm^{-1}): υ 2930, 2876, 2854, 2820, 1766, 1744, 1220, 1189, 1176, 1002, 886, 817.

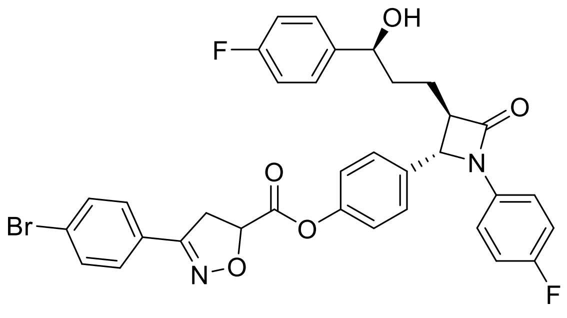

12

4-((2S,3R)-1-(4-Fluorophenyl)-3-((S)-3-(4-fluorophenyl)-3-hydroxypropyl)-4-oxoazetidin-2-yl)phenyl 3-(4-bromophenyl)-4,5-dihydroisoxazole-5-carboxylate (12)

ethyl acetate/ petroleum ether =1:2; yellow solid; 46% yield (122.0 mg); mp: 70-71 °C; ^{1}H NMR (400 MHz, $CDCl_3$) δ 7.54 (s, 4H), 7.32 (d, J = 8.3 Hz, 2H), 7.27 – 7.24 (m, 2H), 7.21 – 7.12 (m, 4H), 7.00 – 6.94 (m, 2H), 6.93 – 6.87 (m, 2H), 5.39 (dd, J = 10.9, 7.3 Hz, 1H), 4.68 (t, J = 6.0 Hz, 1H), 4.62 (d, J = 2.3 Hz, 1H), 3.80 – 3.67 (m, 2H), 3.14 – 2.93 (m, 2H), 1.99 – 1.84 (m, 4H); ^{13}C NMR (100 MHz, $CDCl_3$) δ 168.2, 167.2, 162.0 (d, J = 245.5 Hz), 158.9 (d, J = 243.6 Hz), 155.3, 150.2, 139.9 (d, J = 3.1 Hz), 135.6, 133.5 (d, J = 2.8 Hz), 132.0, 128.3, 127.3 (d, J = 8.0 Hz), 127.1, 127.0, 125.0, 122.0, 118.3 (d, J = 7.8 Hz), 115.8 (d, J = 22.7 Hz), 115.2 (d, J = 21.3 Hz), 78.1, 72.9, 60.6, 60.2, 38.6, 36.4, 24.9; ^{19}F NMR (376 MHz, $CDCl_3$) δ -114.9, -117.5; IR (neat, cm^{-1}): υ 3418, 3063, 2926, 2855, 1740, 1507, 1216, 1198, 1169, 1154, 831, 729.

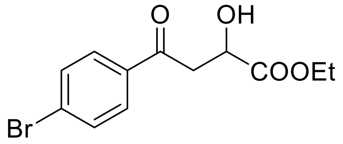

13

Ethyl 4-(4-bromophenyl)-2-hydroxy-4-oxobutanoate (13)

ethyl acetate/ petroleum ether =1:3; yellow solid; 59% yield (53.5 mg); mp: 64-65 °C; ^{1}H NMR (400 MHz, $CDCl_3$) δ 7.80 (d, J = 8.6 Hz, 2H), 7.61 (d, J = 8.6 Hz, 2H), 4.64 (dd, J = 6.0, 3.9 Hz, 1H), 4.26 (q, J = 7.1 Hz, 2H), 3.49 (dd, J = 17.4, 3.9 Hz, 1H), 3.40 (dd, J = 17.4, 6.0 Hz, 1H), 1.27 (t, J = 7.1 Hz, 3H); ^{13}C NMR (100 MHz, $CDCl_3$) δ 196.4, 173.6, 135.1, 132.0, 129.6, 128.8, 67.1, 61.9, 42.1, 14.1; HRMS (ESI-TOF): Anal. Calcd. For $C_{12}H_{13}{}^{79}BrNaO_4^+$: 322.9889, $C_{12}H_{13}{}^{81}BrNaO_4^+$: 324.9869, Found: 324.9883, 324.9874; IR (neat, cm^{-1}): υ 3430, 2987, 2924, 2854, 1737, 1681, 1585, 1216, 1193, 1175, 1039, 1012, 813.

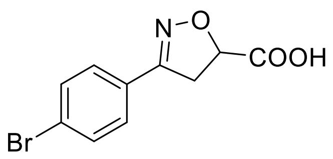

14

3-(4-Bromophenyl)-4,5-dihydroisoxazole-5-carboxylic acid (14)

ethyl acetate; white solid; 90% yield (48.7 mg); mp: 192-193 °C; ^{1}H NMR (400 MHz, DMSO-d6) δ 7.67 (d, *J* = 8.9 Hz, 2H), 7.63 (d, *J* = 8.9 Hz, 2H), 5.18 (dd, *J* = 11.8, 6.9 Hz, 1H), 3.72 (dd, *J* = 17.3, 11.8 Hz, 1H), 3.57 (dd, *J* = 17.3, 6.9 Hz, 1H); ^{13}C NMR (100 MHz, DMSO-d6) δ 171.6, 155.6, 132.0, 128.8, 127.9, 123.8, 78.1, 38.3; HRMS (ESI-TOF): Anal. Calcd. For $C_{10}H_8{}^{79}BrNNaO_3^+$: 291.9580, $C_{10}H_8{}^{81}BrNNaO_3^+$: 293.9559, Found: 291.9596, 293.9528; IR (neat, cm^{-1}): υ 3043, 2977, 2918, 2853, 1715, 1591, 1401, 1222, 894, 871, 837, 822.

Spectroscopic data for substrates and products

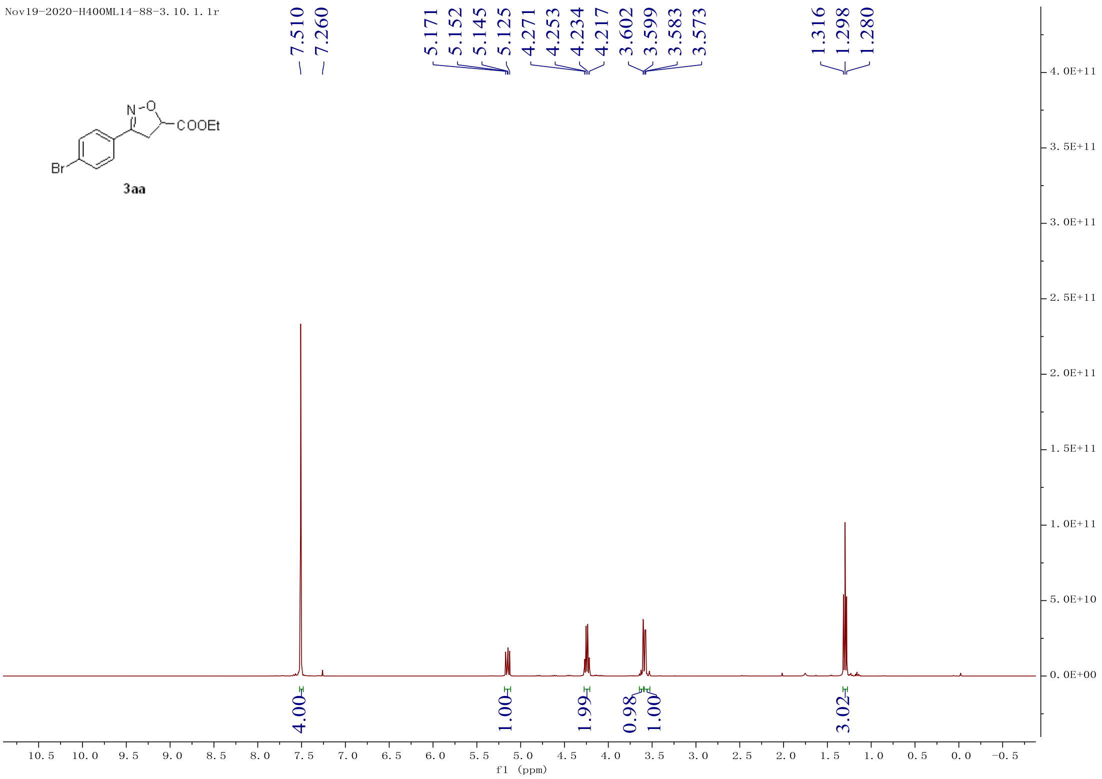

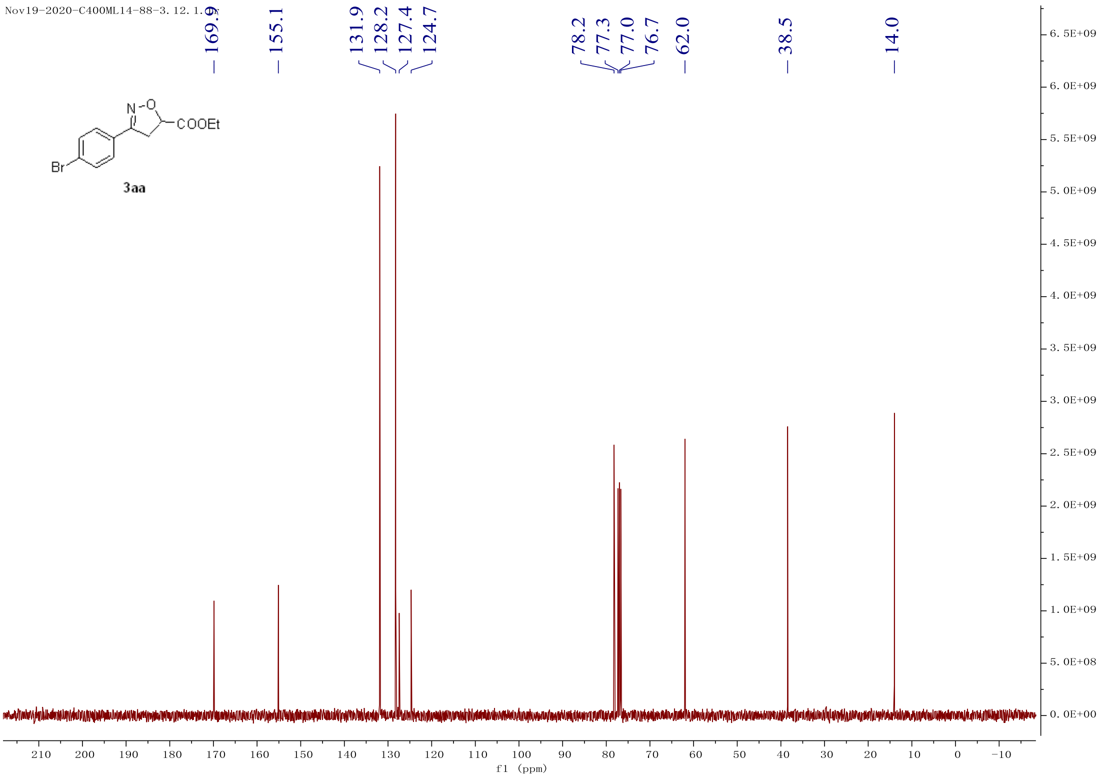

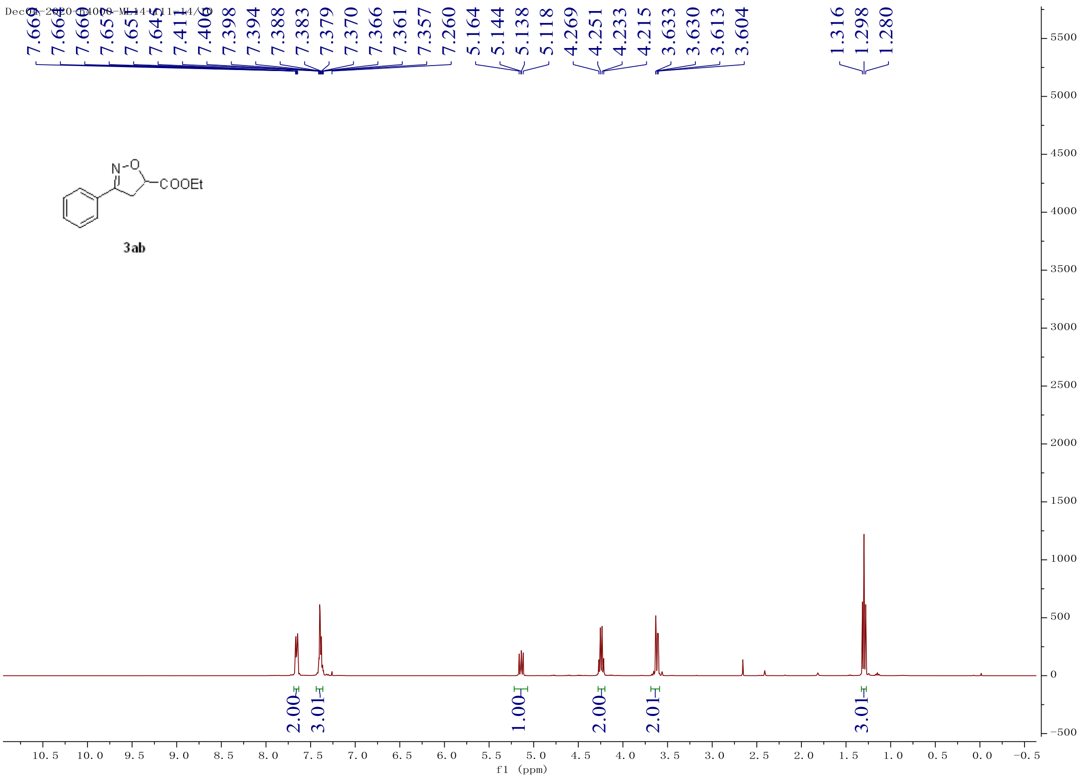
7.669 7.664 7.660 7.657 7.651 7.645 7.411 7.406 7.398 7.394 7.388 7.383 7.379 7.370 7.366 7.361 7.357 7.260 5.164 5.144 5.138 5.118 4.269 4.251 4.233 4.215 3.633 3.630 3.613 3.604 1.316 1.298 1.280
COOEt
3ab
2.00 3.01 1.00 2.00 2.01 3.01
f1 (ppm)

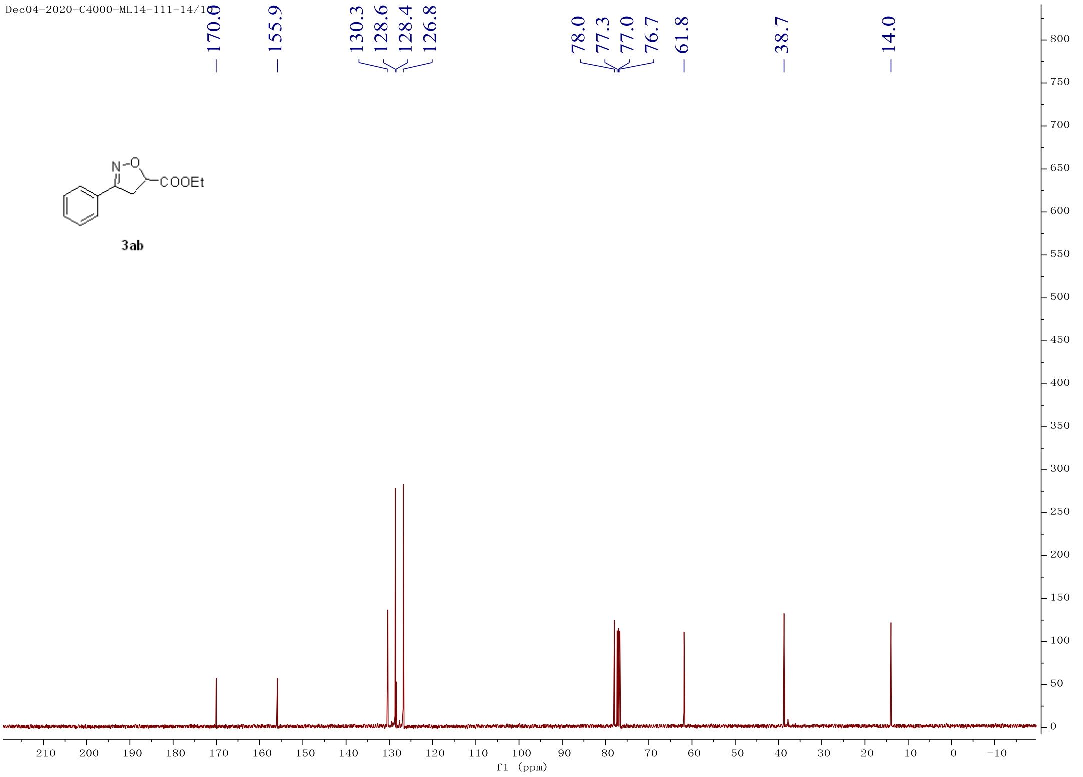
Dec04-2020-C4000-ML14-111-14/1
170.0 155.9 130.3 128.6 128.4 126.8 78.0 77.3 77.0 76.7 61.8 38.7 14.0
COOEt
3ab
f1 (ppm)

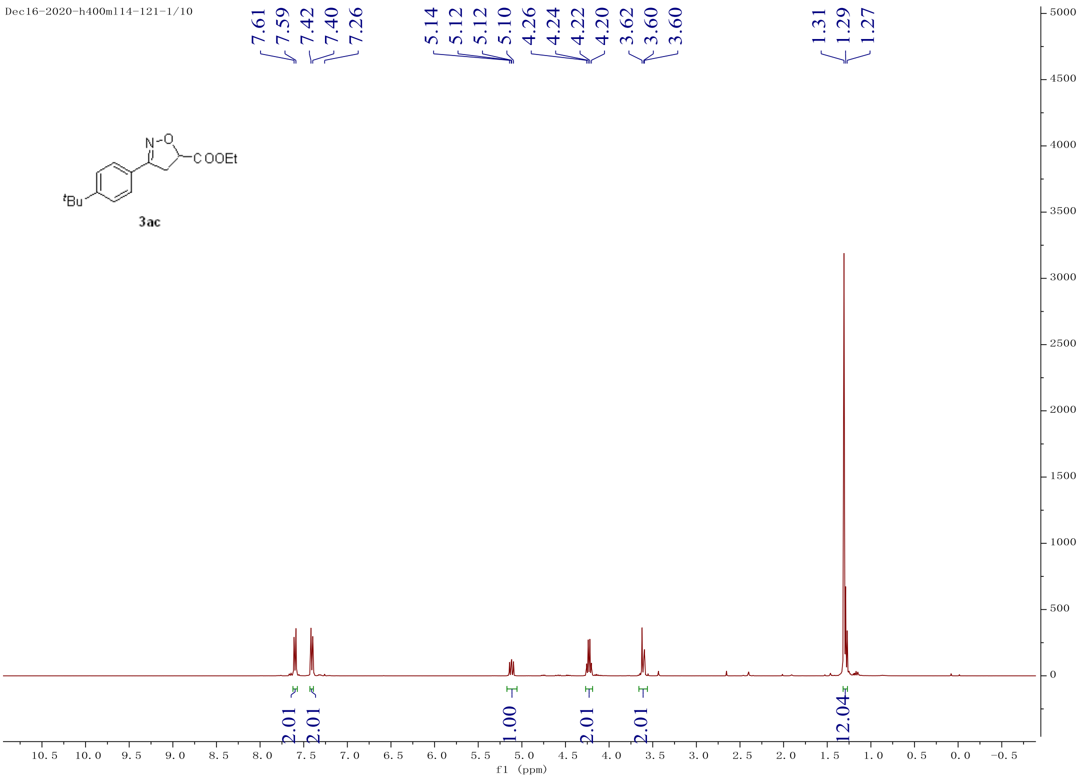

Dec16-2020-h400ml14-121-1/10
7.61
7.59
7.42
7.40
7.26
5.14
5.12
5.12
5.10
4.26
4.24
4.22
4.20
3.62
3.60
3.60
1.31
1.29
1.27
COOEt
^{t}Bu
3ac
2.01
2.01
1.00
2.01
2.01
12.04
f1 (ppm)

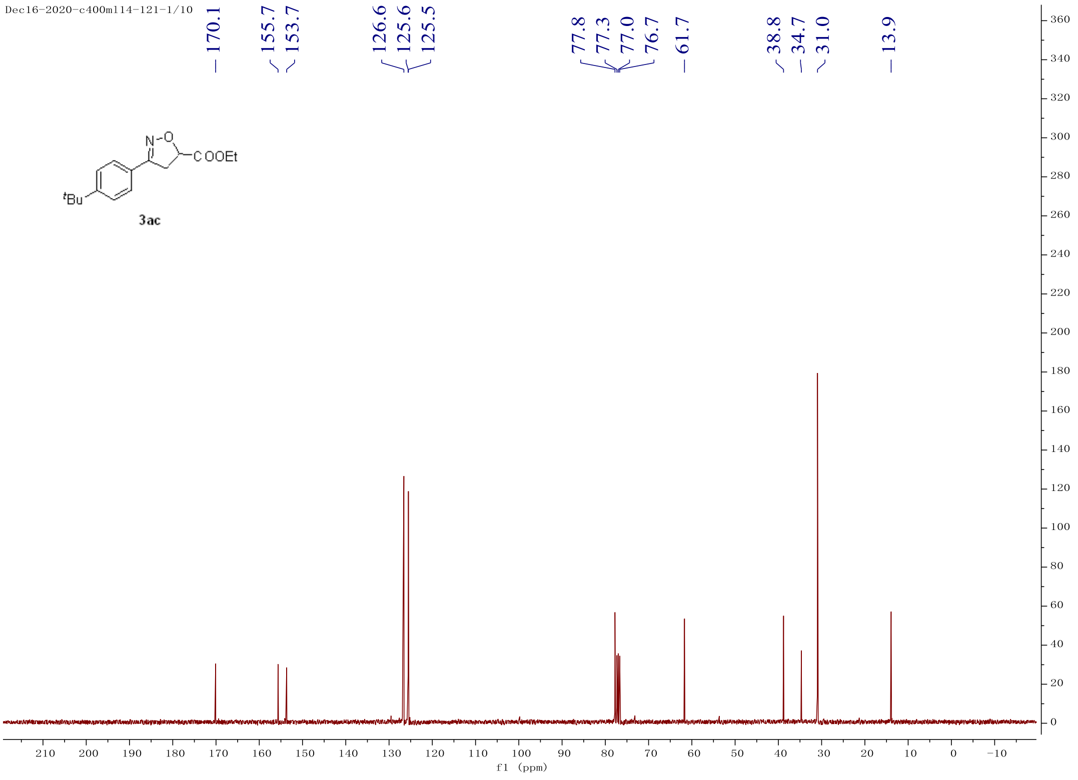

Dec16-2020-c400ml14-121-1/10
170.1
155.7
153.7
126.6
125.6
125.5
77.8
77.3
77.0
76.7
61.7
38.8
34.7
31.0
13.9
COOEt
^{t}Bu
3ac
f1 (ppm)

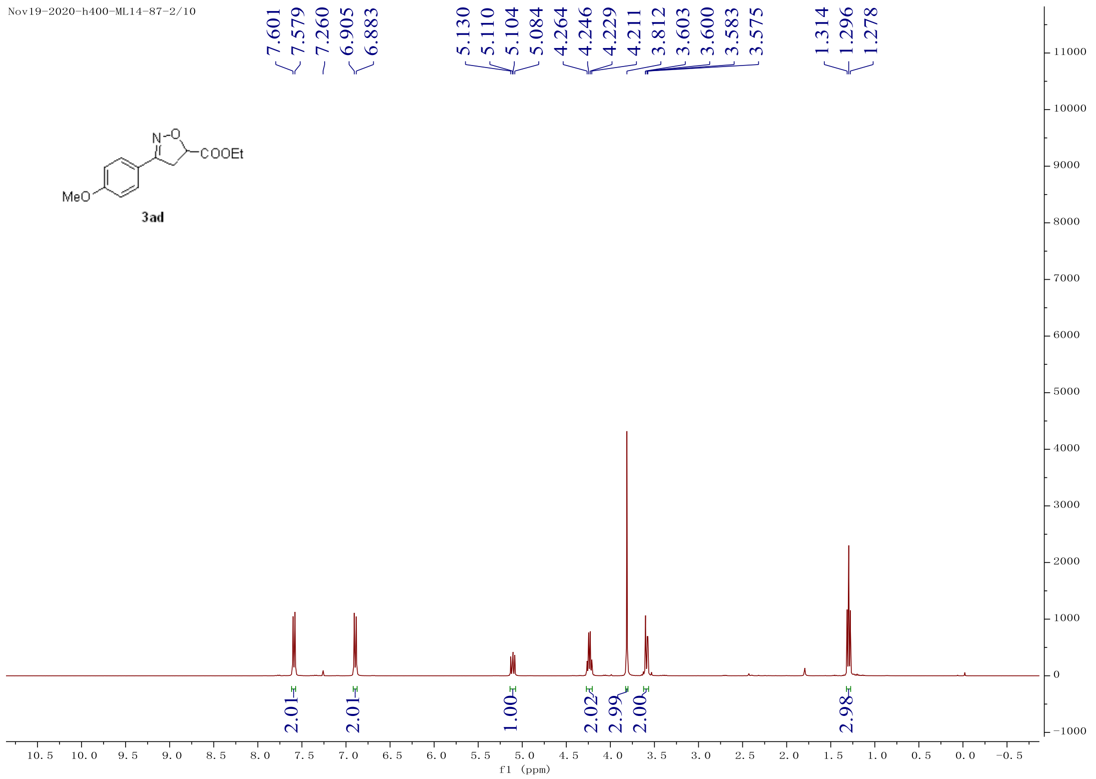
Nov19-2020-h400-ML14-87-2/10
7.601
7.579
7.260
6.905
6.883
5.130
5.110
5.104
5.084
4.264
4.246
4.229
4.211
3.812
3.603
3.600
3.583
3.575
1.314
1.296
1.278
MeO
N-O
COOEt
3ad
2.01
2.01
1.00
2.02
2.99
2.00
2.98
f1 (ppm)

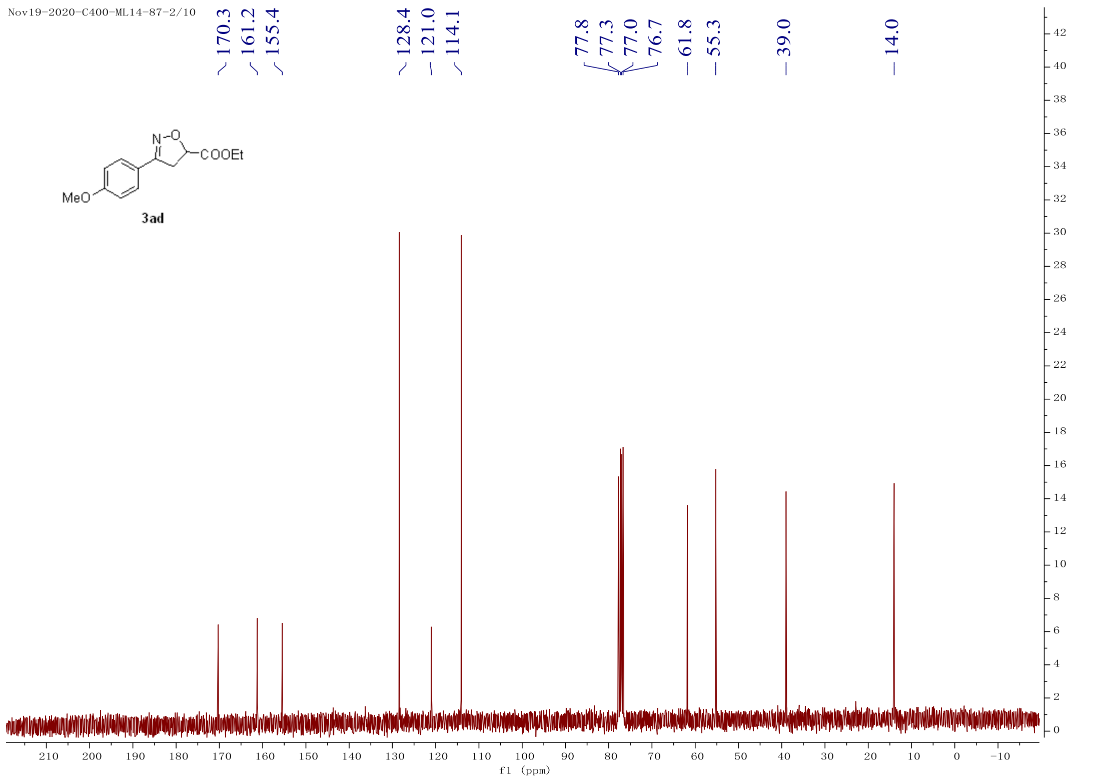
Nov19-2020-C400-ML14-87-2/10
170.3
161.2
155.4
128.4
121.0
114.1
77.8
77.3
77.0
76.7
61.8
55.3
39.0
14.0
MeO
N-O
COOEt
3ad
f1 (ppm)

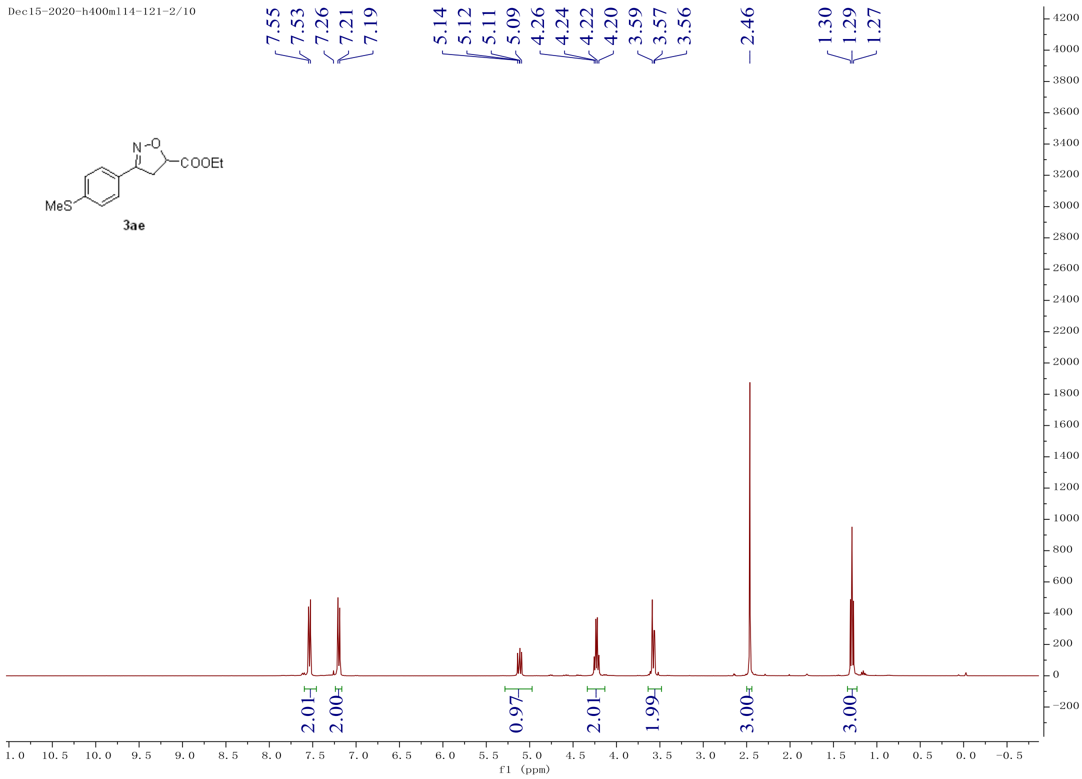
Dec15-2020-h400ml14-121-2/10
7.55 7.53 7.26 7.21 7.19 5.14 5.12 5.11 5.09 4.26 4.24 4.22 4.20 3.59 3.57 3.56 2.46 1.30 1.29 1.27
N-O
COOEt
MeS
3ae
2.01 2.00 0.97 2.01 1.99 3.00 3.00
f1 (ppm)

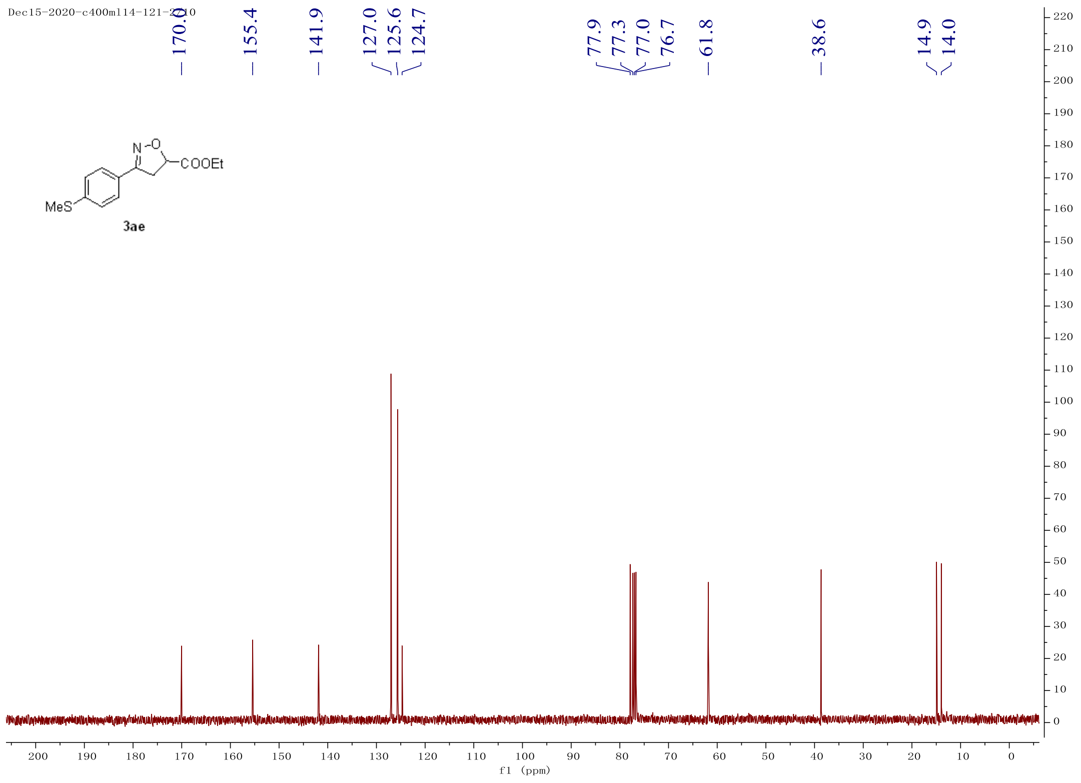
Dec15-2020-c400ml14-121-2/10
170.0 155.4 141.9 127.0 125.6 124.7 77.9 77.3 77.0 76.7 61.8 38.6 14.9 14.0
N-O
COOEt
MeS
3ae
f1 (ppm)

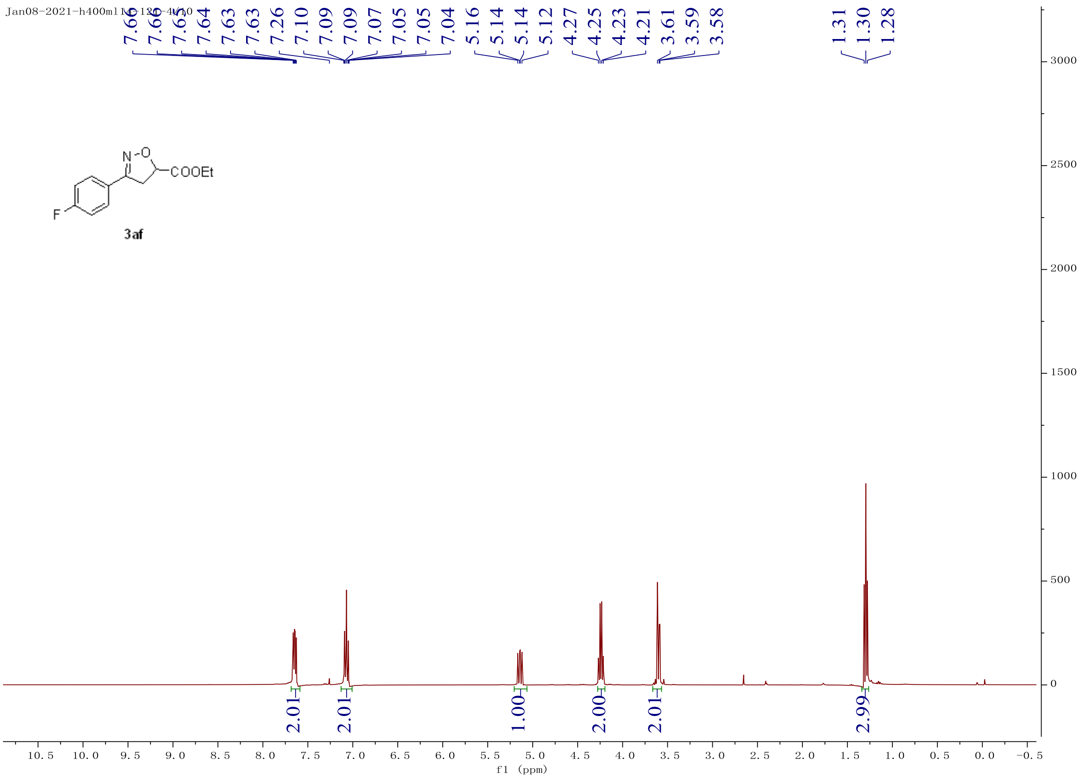
7.66
7.66
7.65
7.64
7.63
7.63
7.26
7.10
7.09
7.09
7.07
7.05
7.05
7.04
5.16
5.14
5.14
5.12
4.27
4.25
4.23
4.21
3.61
3.59
3.58
1.31
1.30
1.28
N-O
COOEt
F
3af
2.01
2.01
1.00
2.00
2.01
2.99
f1 (ppm)

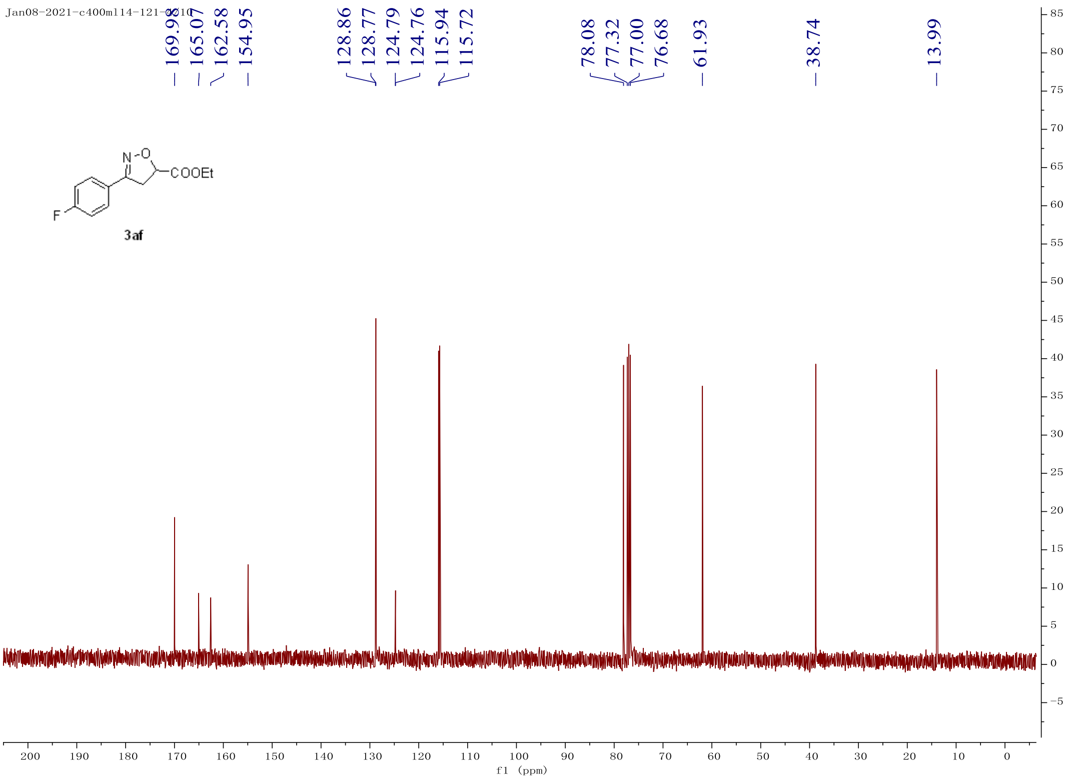
169.98
165.07
162.58
154.95
128.86
128.77
124.79
124.76
115.94
115.72
78.08
77.32
77.00
76.68
61.93
38.74
13.99
N-O
COOEt
F
3af
f1 (ppm)

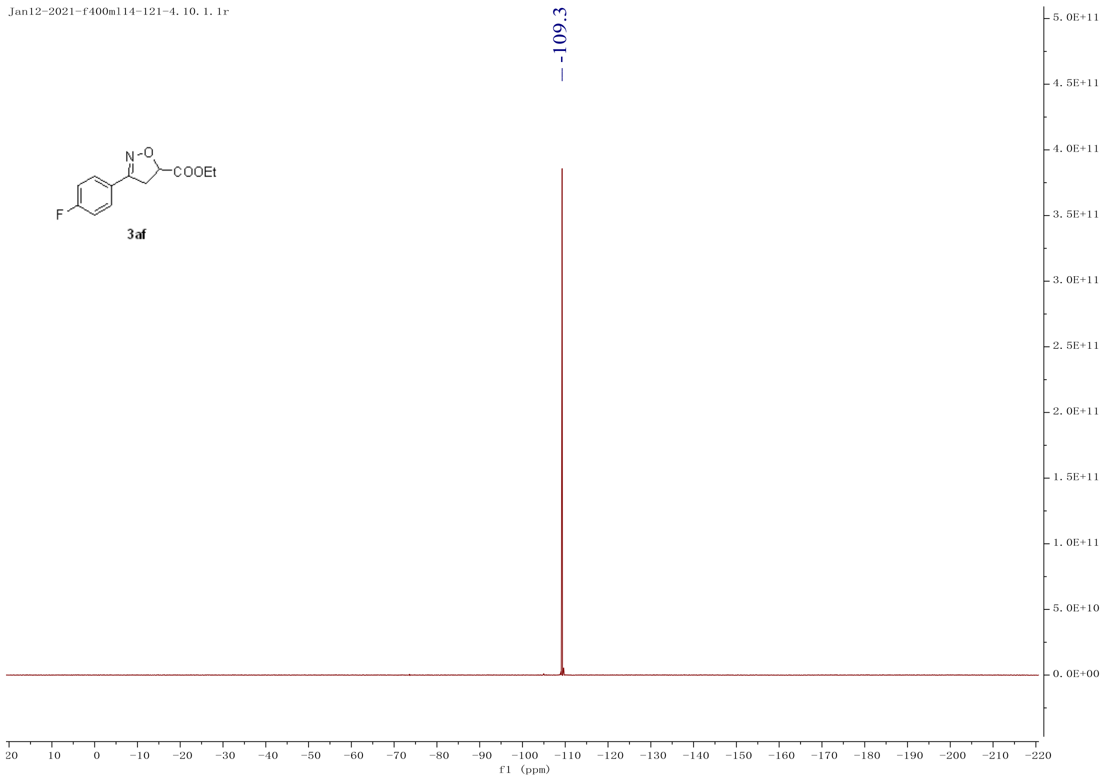
Jan12-2021-f400ml14-121-4.10.1.1r
-109.3
N-O
COOEt
F
3af
f1 (ppm)

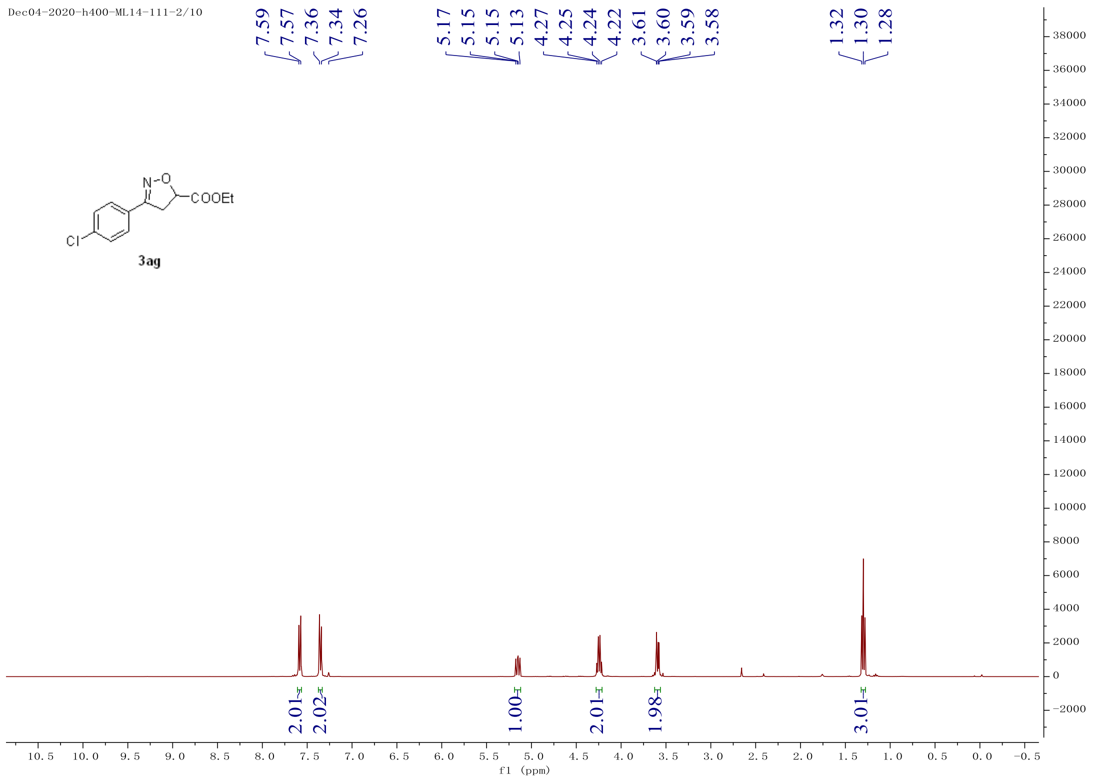
Dec04-2020-h400-ML14-111-2/10
7.59
7.57
7.36
7.34
7.26
5.17
5.15
5.15
5.13
4.27
4.25
4.24
4.22
3.61
3.60
3.59
3.58
1.32
1.30
1.28
N-O
COOEt
Cl
3ag
2.01
2.02
1.00
2.01
1.98
3.01
f1 (ppm)

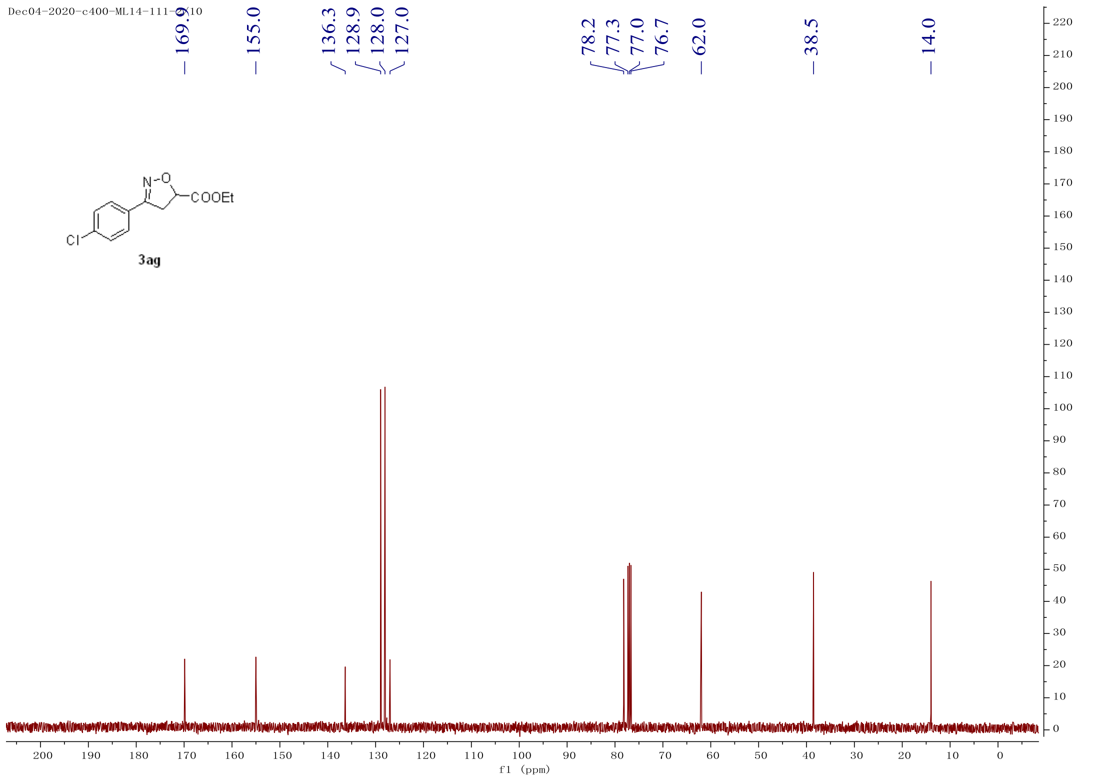
Dec04-2020-c400-ML14-111-C10
169.9
155.0
136.3
128.9
128.0
127.0
78.2
77.3
77.0
76.7
62.0
38.5
14.0
N-O
COOEt
Cl
3ag
f1 (ppm)

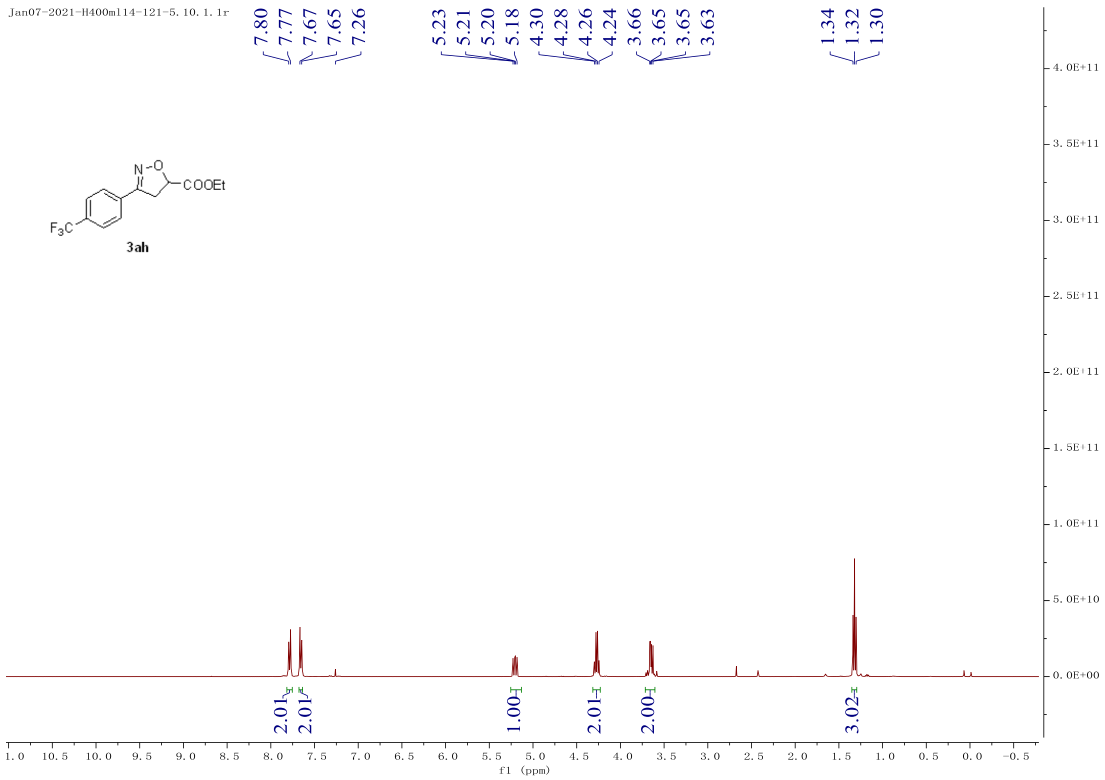
Jan07-2021-H400ml14-121-5.10.1.1r
7.80
7.77
7.67
7.65
7.26
5.23
5.21
5.20
5.18
4.30
4.28
4.26
4.24
3.66
3.65
3.65
3.63
1.34
1.32
1.30
N-O
COOEt
F3C
3ah
2.01
2.01
1.00
2.01
2.00
3.02
f1 (ppm)

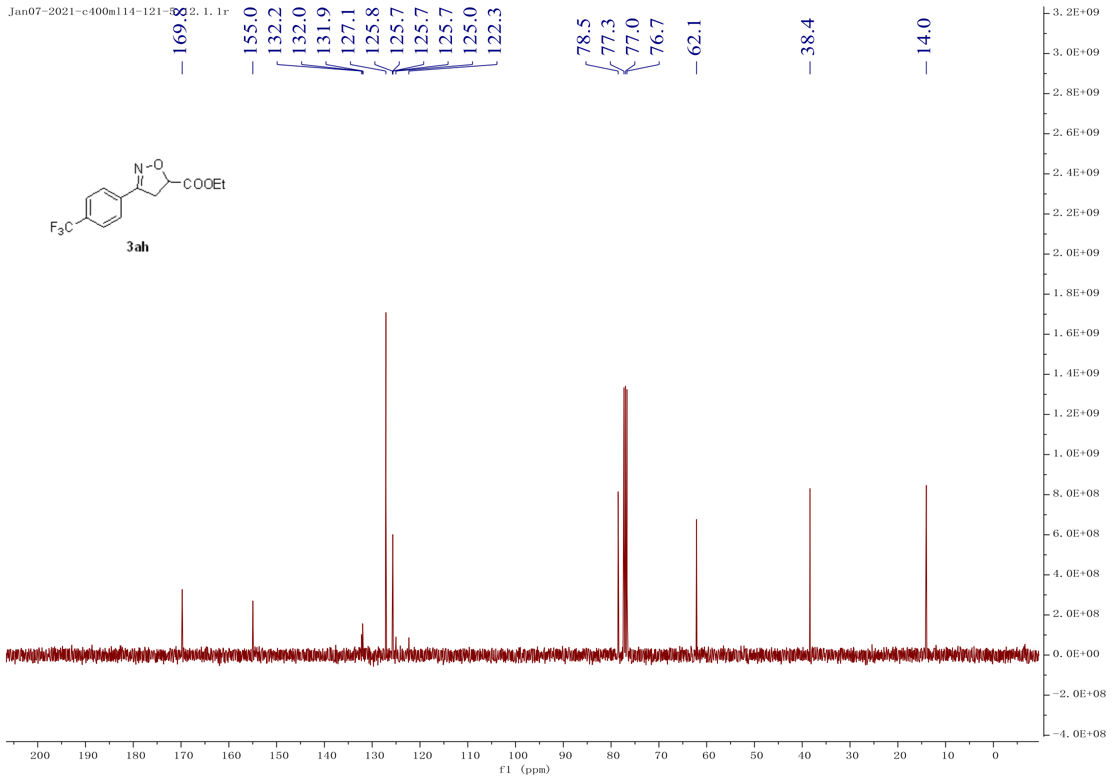
Jan07-2021-c400ml14-121-5.12.1.1r
169.8
155.0
132.2
132.0
131.9
127.1
125.8
125.7
125.7
125.7
125.0
122.3
78.5
77.3
77.0
76.7
62.1
38.4
14.0
COOEt
F3C
3ah
f1 (ppm)

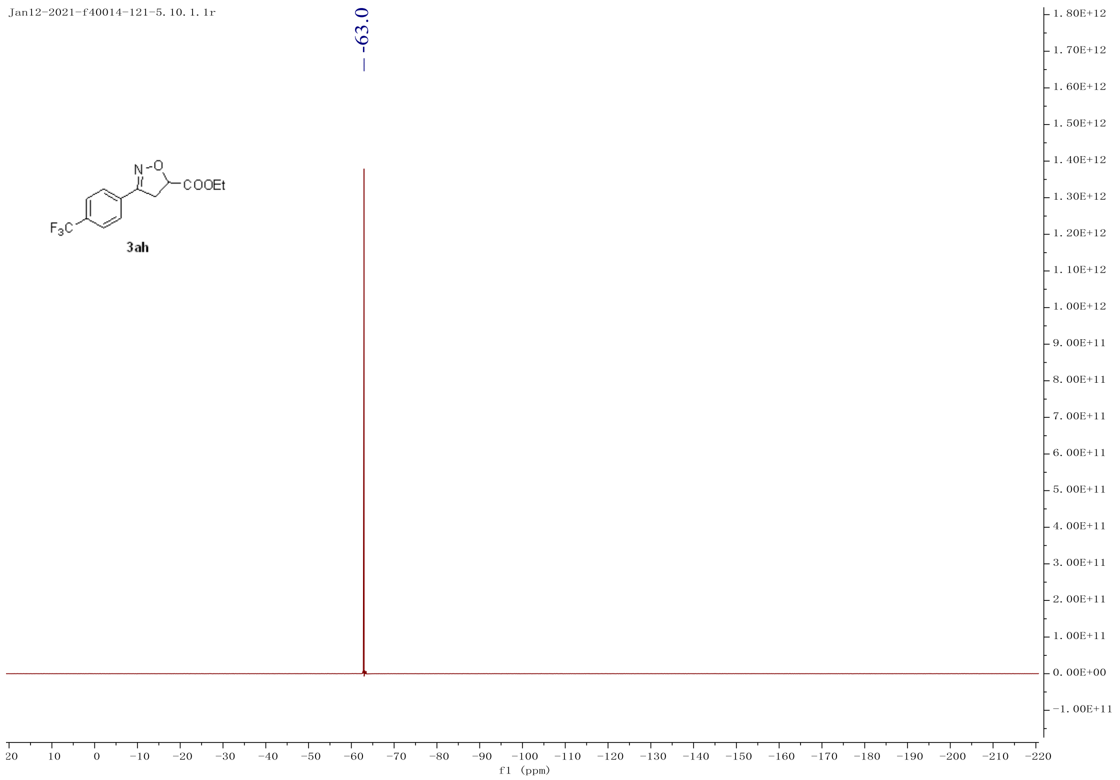
Jan12-2021-f40014-121-5.10.1.1r
-63.0
COOEt
F3C
3ah
f1 (ppm)

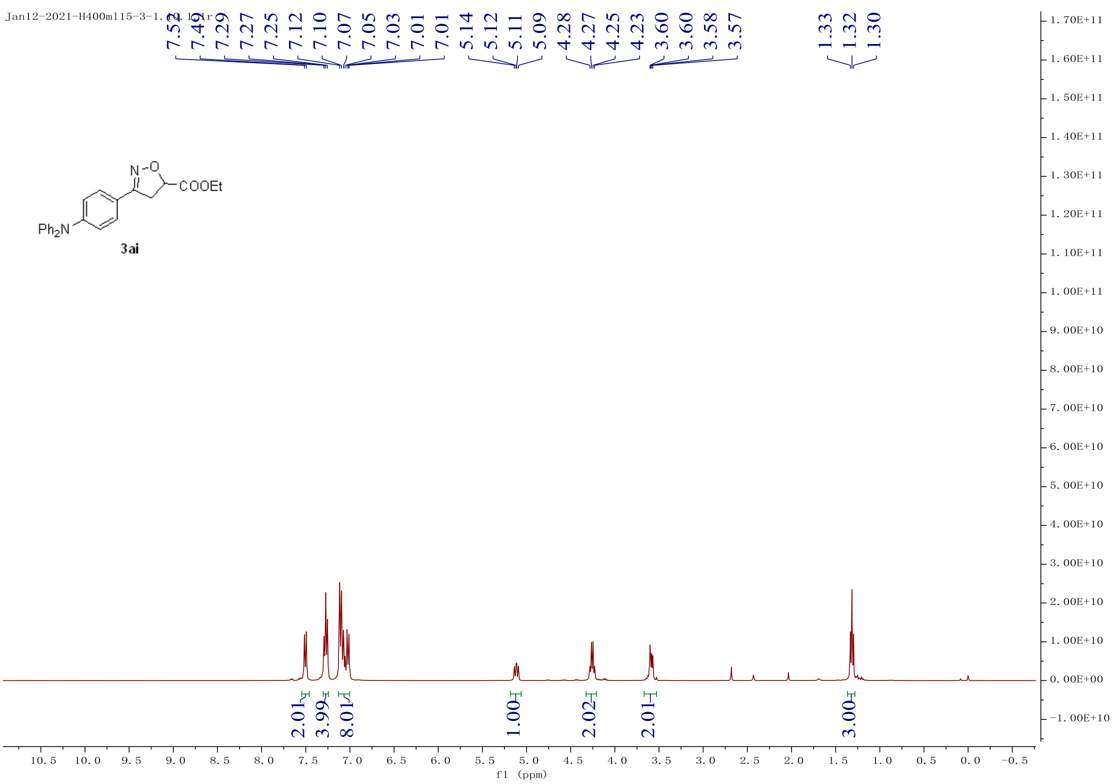
7.52 7.49 7.29 7.27 7.25 7.12 7.10 7.07 7.05 7.03 7.01 7.01 5.14 5.12 5.11 5.09 4.28 4.27 4.25 4.23 3.60 3.60 3.58 3.57 1.33 1.32 1.30
N-O
COOEt
Ph2N
3ai
2.01 3.99 8.01 1.00 2.02 2.01 3.00
f1 (ppm)

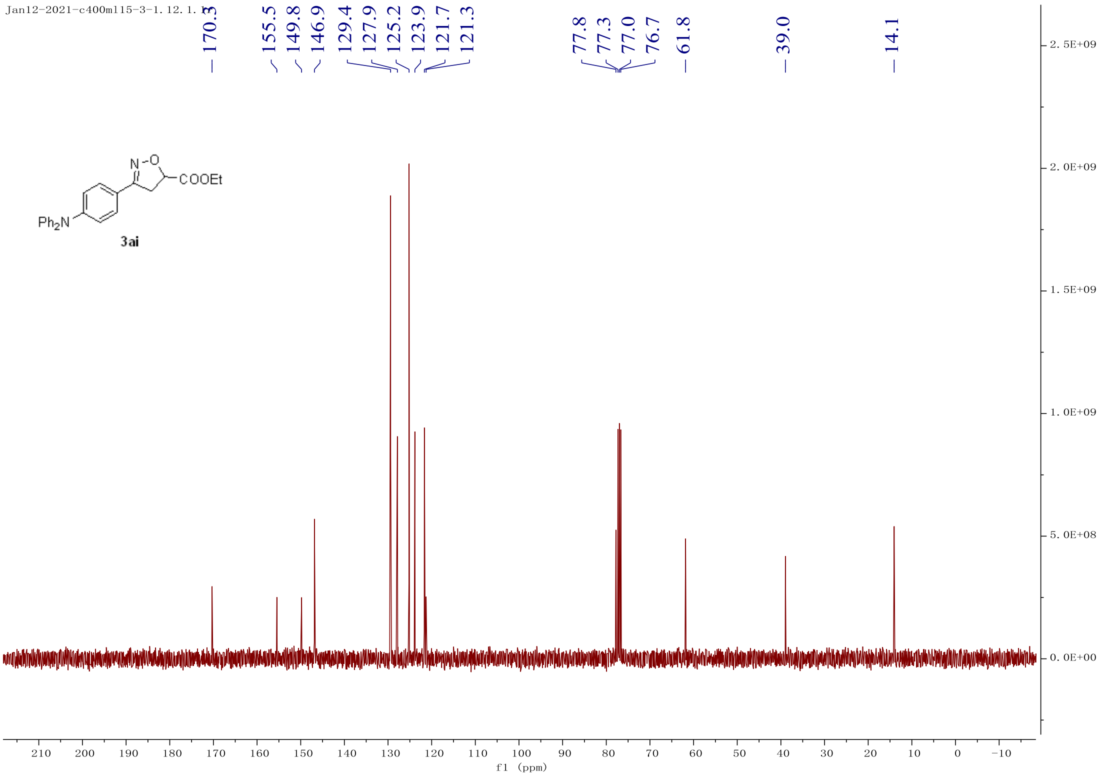
170.3 155.5 149.8 146.9 129.4 127.9 125.2 123.9 121.7 121.3 77.8 77.3 77.0 76.7 61.8 39.0 14.1
N-O
COOEt
Ph2N
3ai
f1 (ppm)

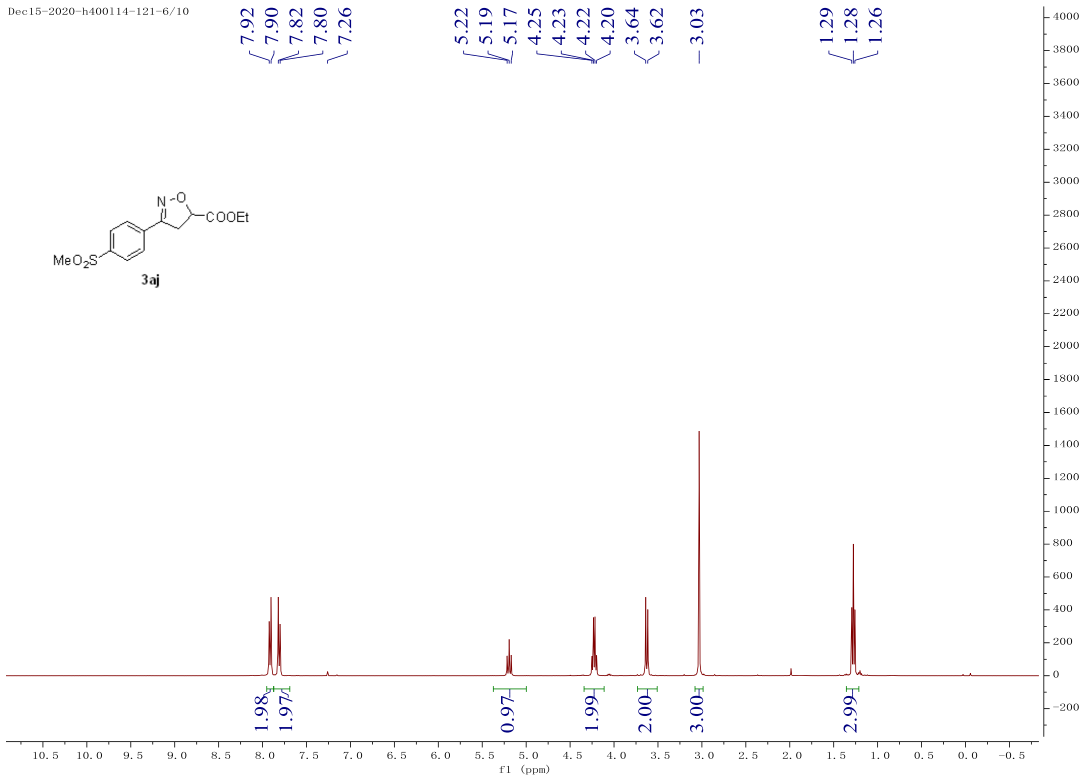
Dec15-2020-h400114-121-6/10
7.92
7.90
7.82
7.80
7.26
5.22
5.19
5.17
4.25
4.23
4.22
4.20
3.64
3.62
3.03
1.29
1.28
1.26
3aj
1.98
1.97
0.97
1.99
2.00
3.00
2.99
f1 (ppm)

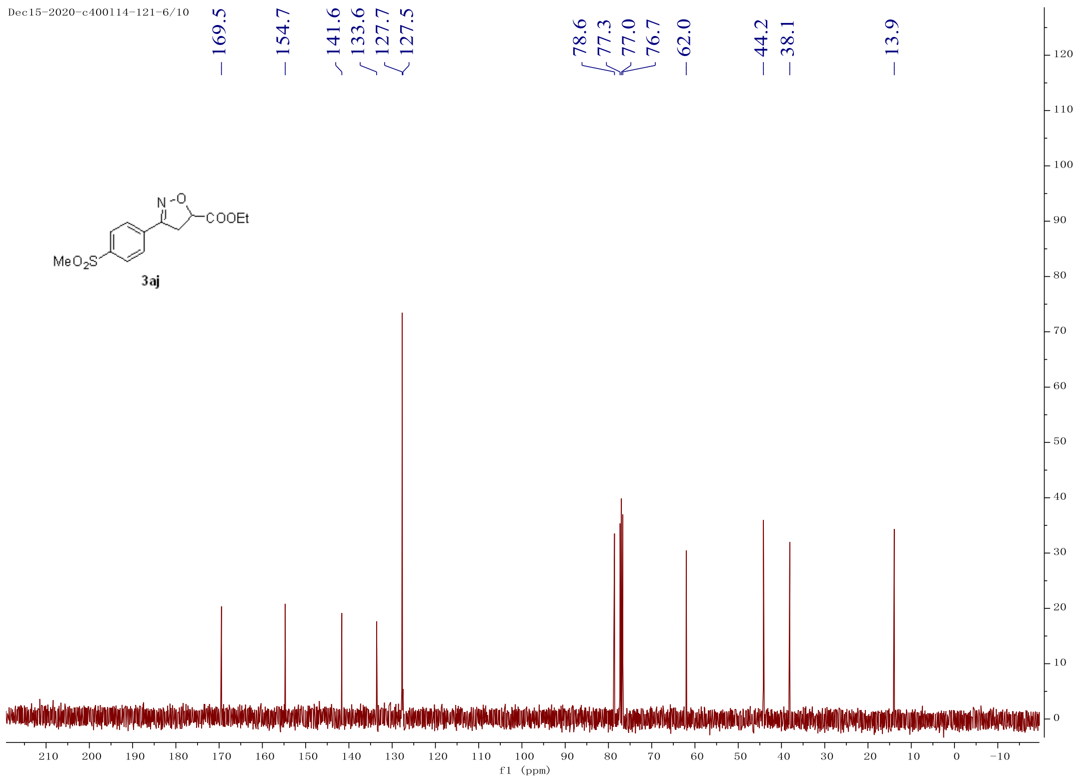
Dec15-2020-c400114-121-6/10
169.5
154.7
141.6
133.6
127.7
127.5
78.6
77.3
77.0
76.7
62.0
44.2
38.1
13.9
3aj
f1 (ppm)

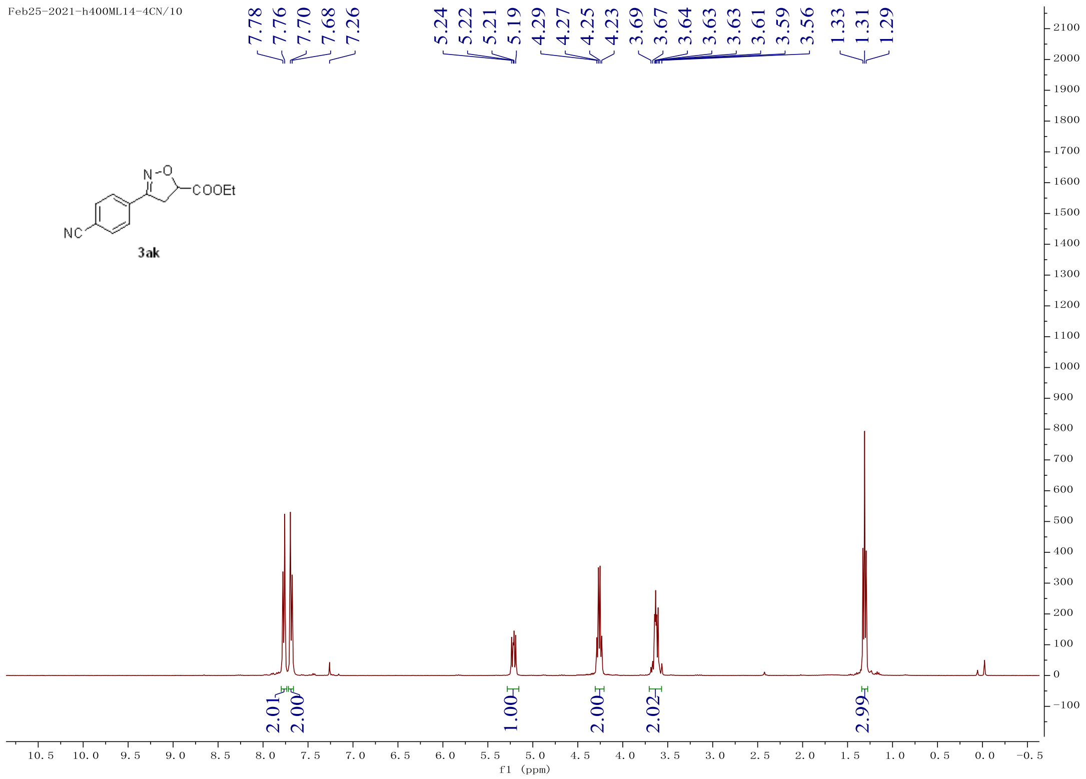
Feb25-2021-h400ML14-4CN/10
7.78
7.76
7.70
7.68
7.26
5.24
5.22
5.21
5.19
4.29
4.27
4.25
4.23
3.69
3.67
3.64
3.63
3.63
3.61
3.59
3.56
1.33
1.31
1.29
N-O
COOEt
NC
3ak
2.01
2.00
1.00
2.00
2.02
2.99
f1 (ppm)

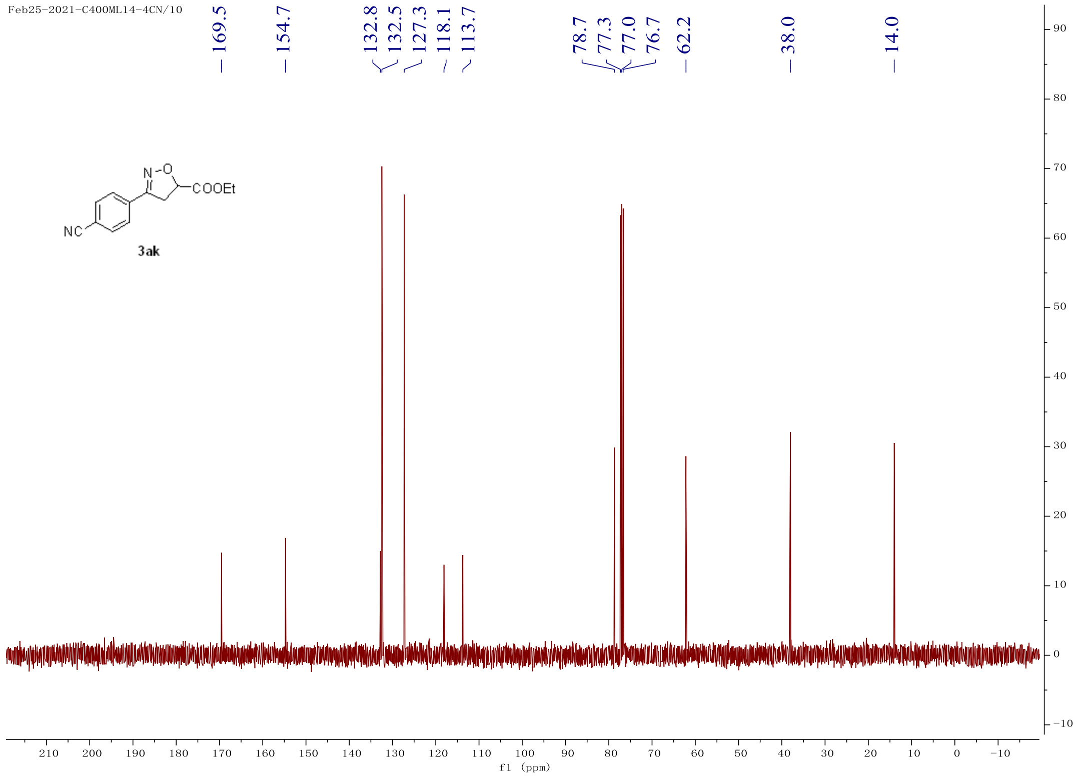
Feb25-2021-C400ML14-4CN/10
169.5
154.7
132.8
132.5
127.3
118.1
113.7
78.7
77.3
77.0
76.7
62.2
38.0
14.0
N-O
COOEt
NC
3ak
f1 (ppm)

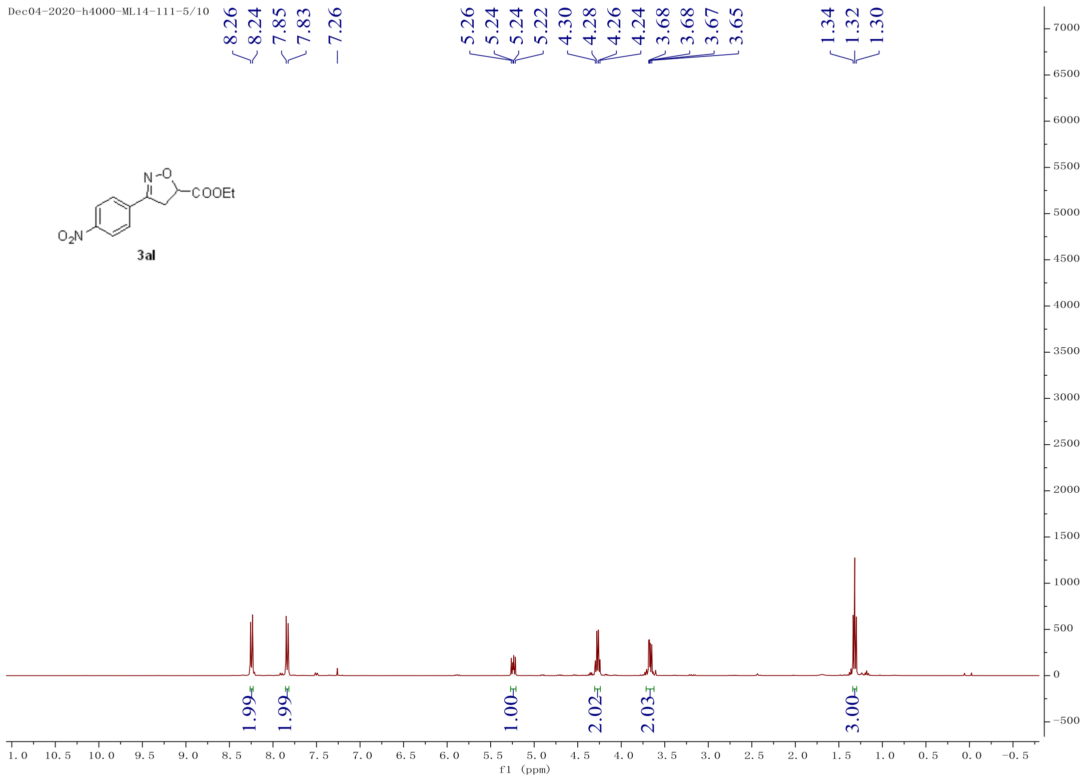
Dec04-2020-h4000-ML14-111-5/10
8.26
8.24
7.85
7.83
7.26
5.26
5.24
5.24
5.22
4.30
4.28
4.26
4.24
3.68
3.68
3.67
3.65
1.34
1.32
1.30
N-O
COOEt
O2N
3al
1.99
1.99
1.00
2.02
2.03
3.00
f1 (ppm)

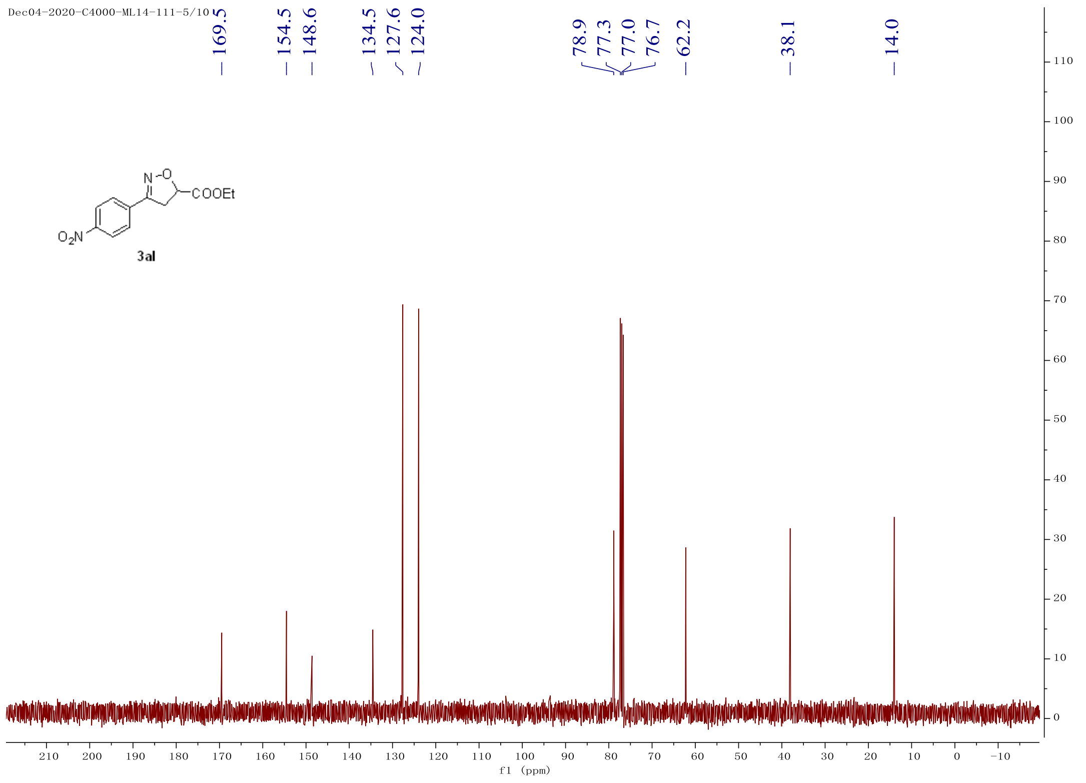
Dec04-2020-C4000-ML14-111-5/10
169.5
154.5
148.6
134.5
127.6
124.0
78.9
77.3
77.0
76.7
62.2
38.1
14.0
N-O
COOEt
O2N
3al
f1 (ppm)

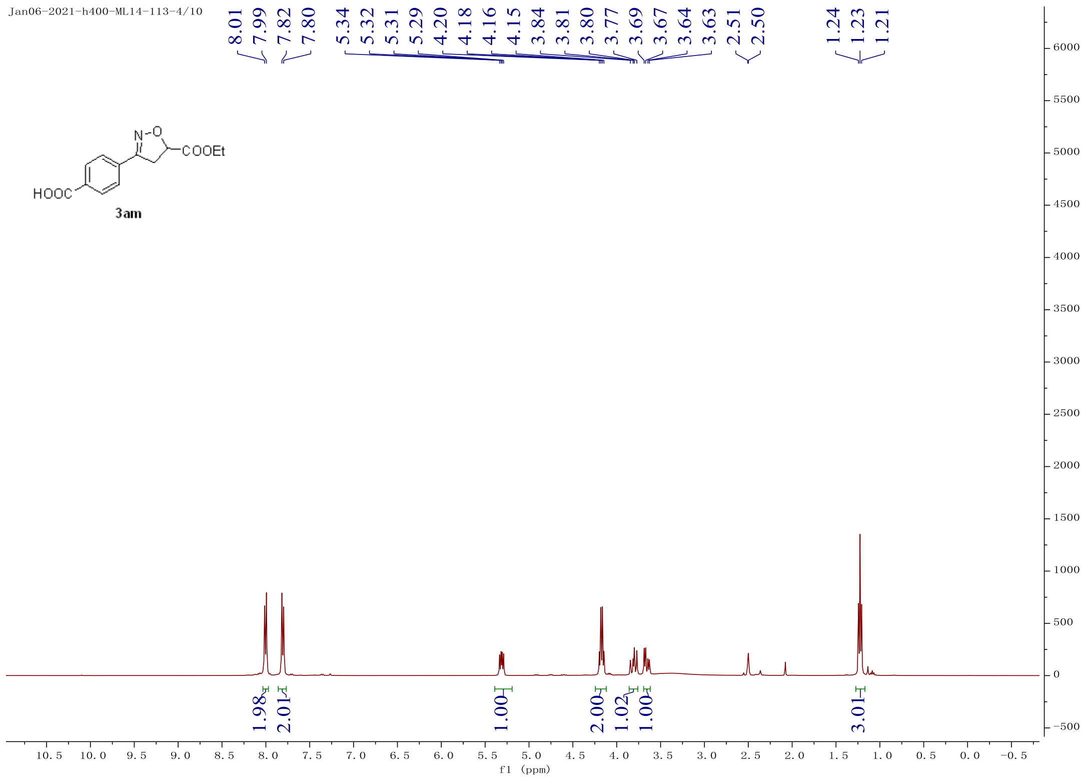

3am

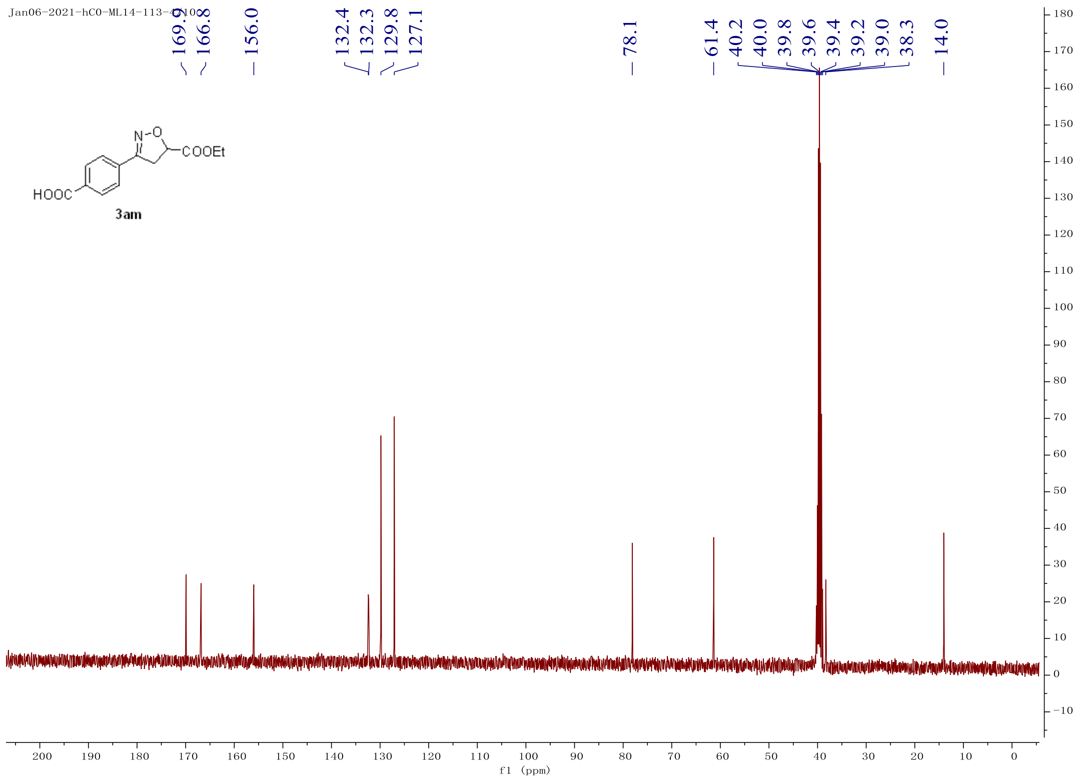

3am

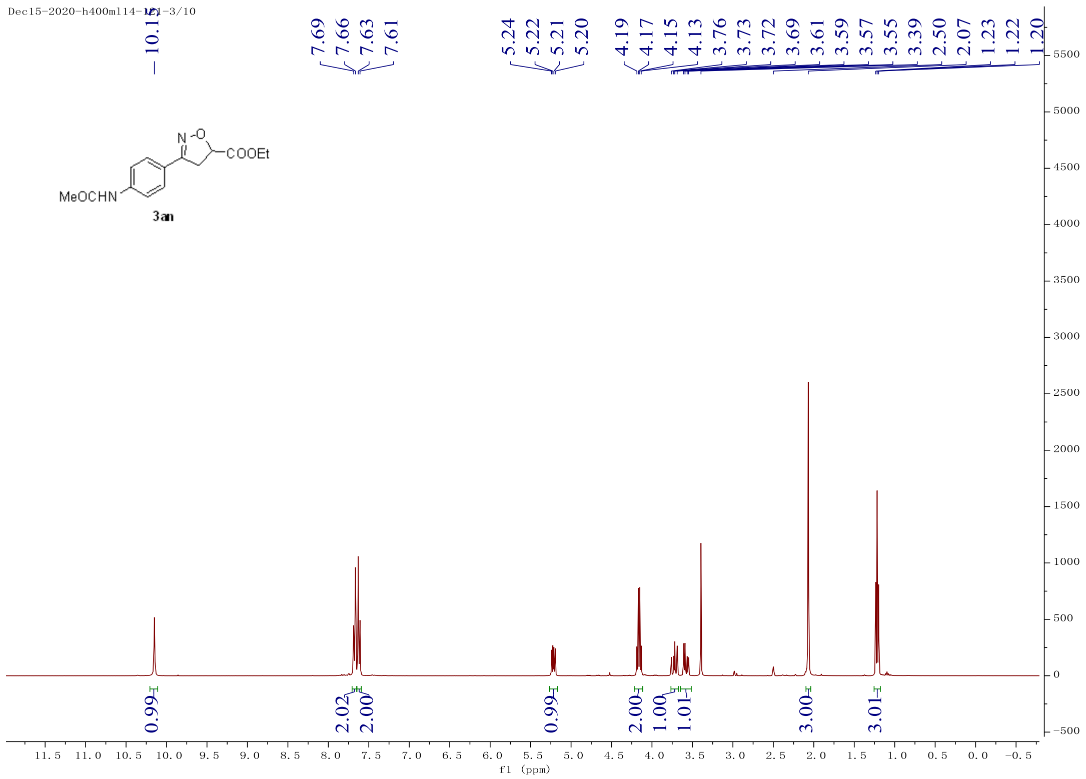
Dec15-2020-h400ml14-121-3/10
MeOCHN
COOEt
3an
f1 (ppm)

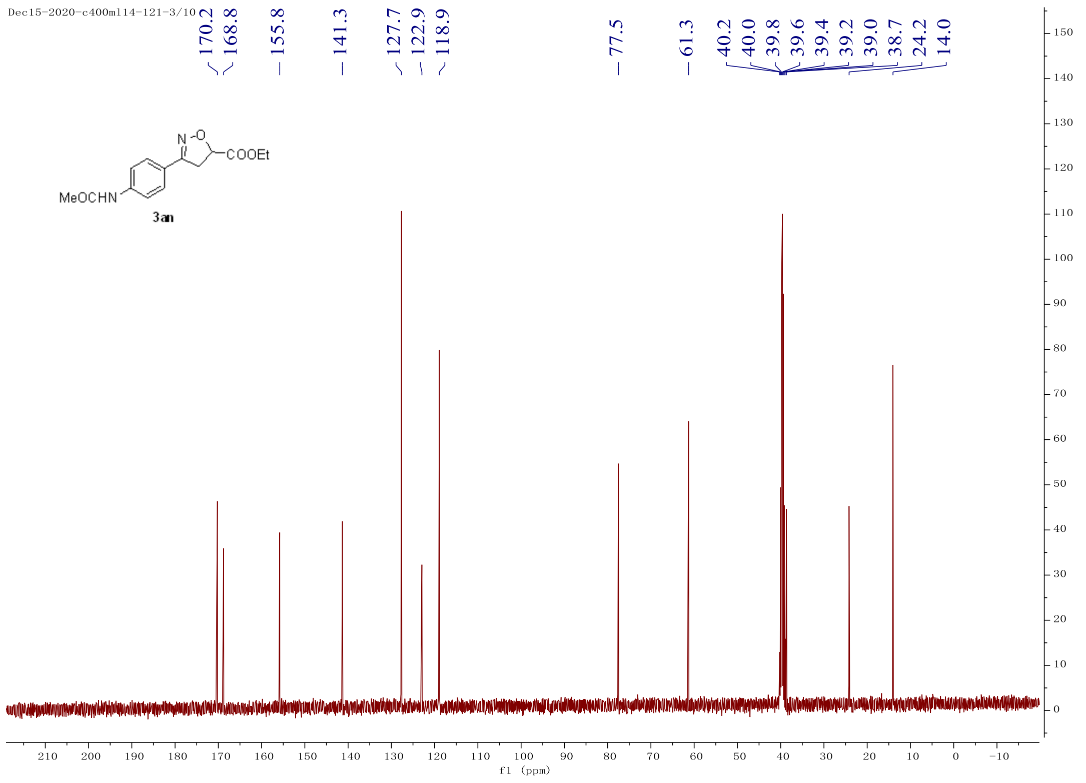
Dec15-2020-c400ml14-121-3/10
MeOCHN
COOEt
3an
f1 (ppm)

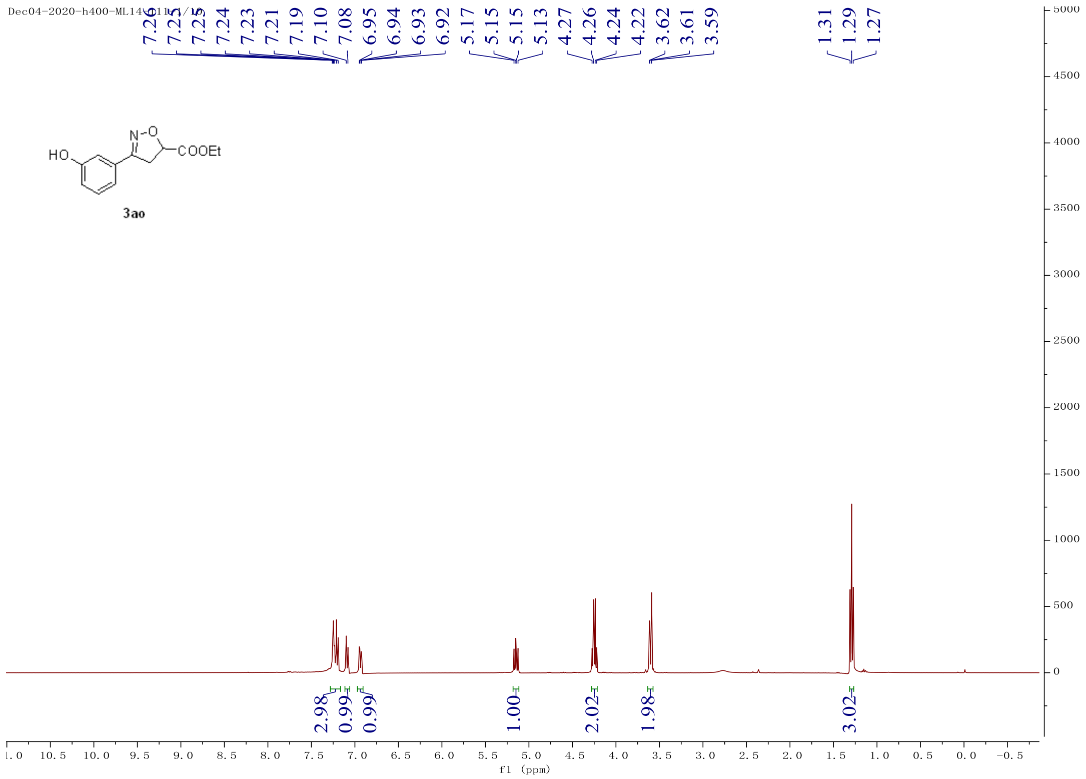
HO
N-O
COOEt
3ao
f1 (ppm)

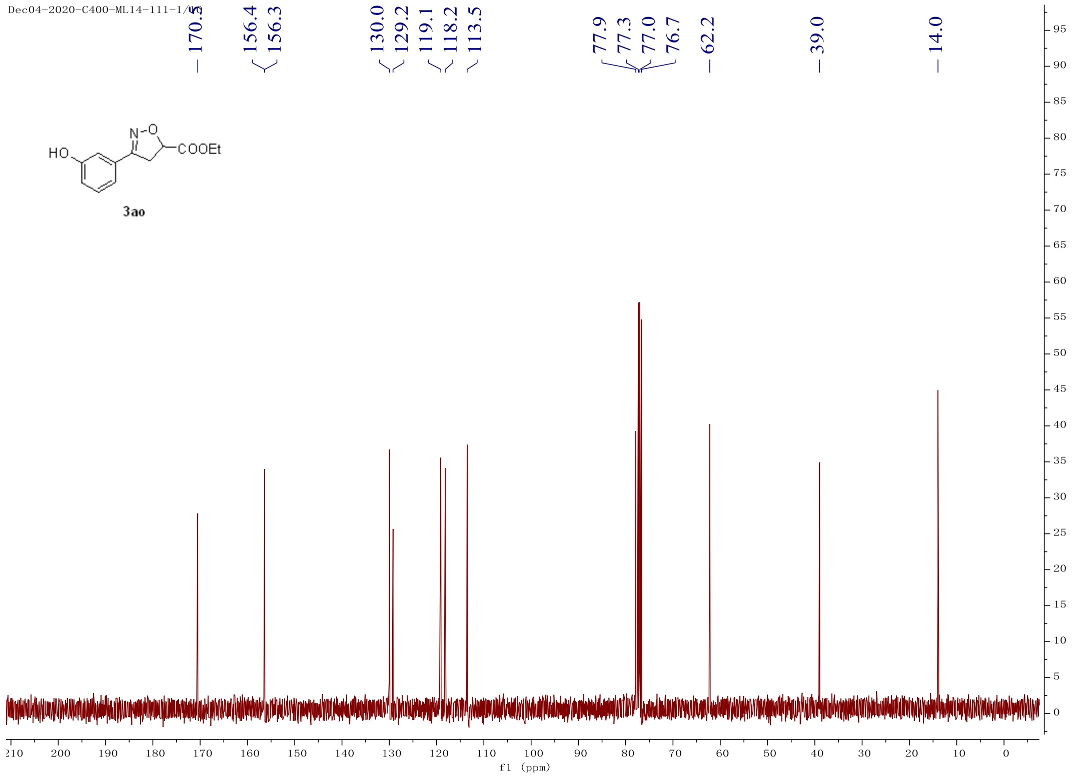
Dec04-2020-C400-ML14-111-1/45
HO
N-O
COOEt
3ao
170.5
156.4
156.3
130.0
129.2
119.1
118.2
113.5
77.9
77.3
77.0
76.7
62.2
39.0
14.0
f1 (ppm)

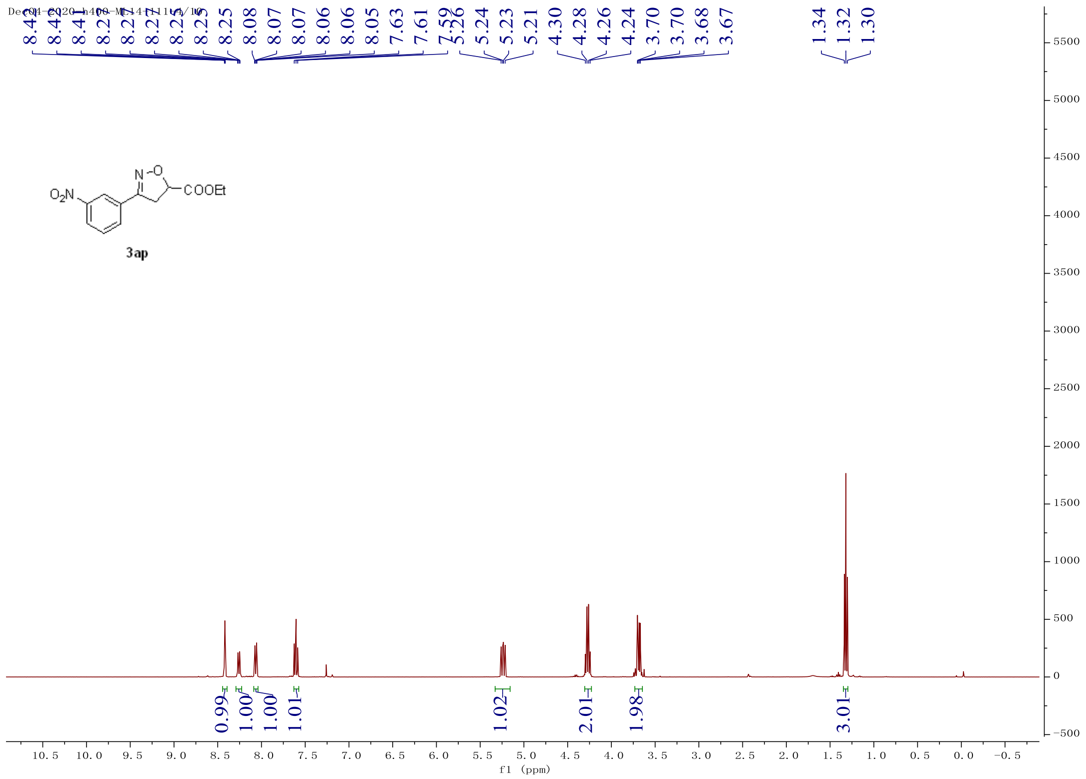
Dec04-2020-h400-ML14-111-4/10
8.42
8.41
8.41
8.27
8.27
8.25
8.25
8.25
8.08
8.07
8.07
8.06
8.06
8.05
7.63
7.61
7.59
5.26
5.24
5.23
5.21
4.30
4.28
4.26
4.24
3.70
3.70
3.68
3.67
1.34
1.32
1.30
0.99
1.00
1.00
1.01
1.02
2.01
1.98
3.01
f1 (ppm)

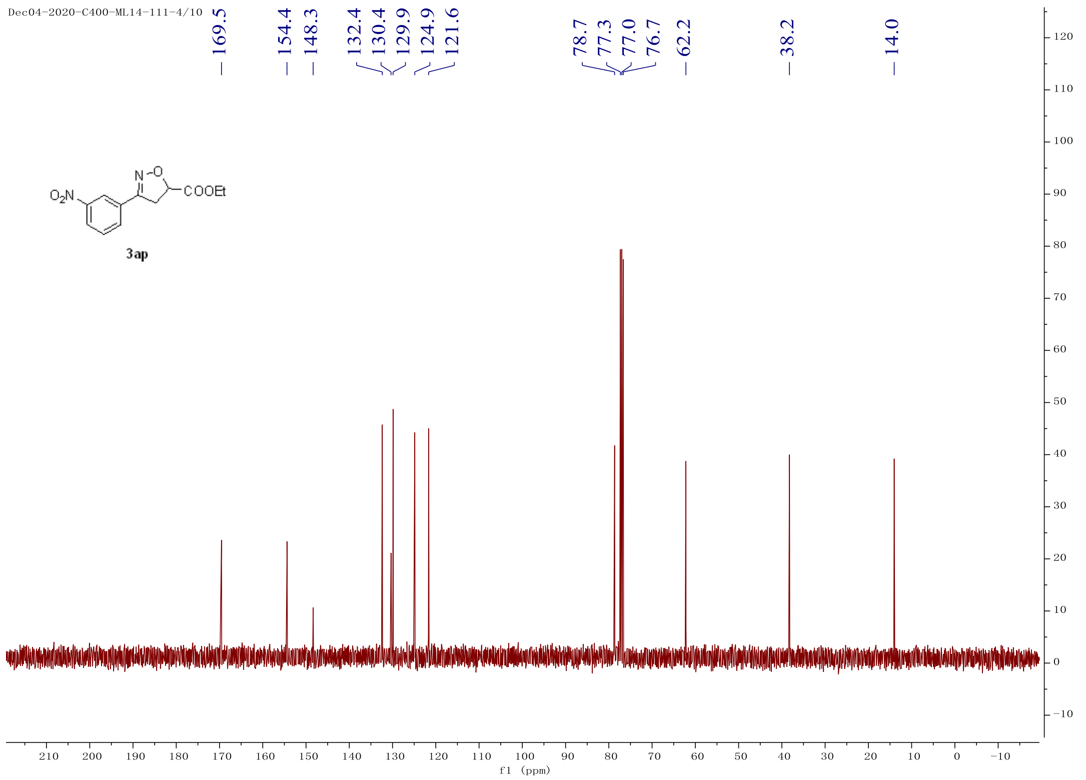
Dec04-2020-C400-ML14-111-4/10
169.5
154.4
148.3
132.4
130.4
129.9
124.9
121.6
78.7
77.3
77.0
76.7
62.2
38.2
14.0
f1 (ppm)

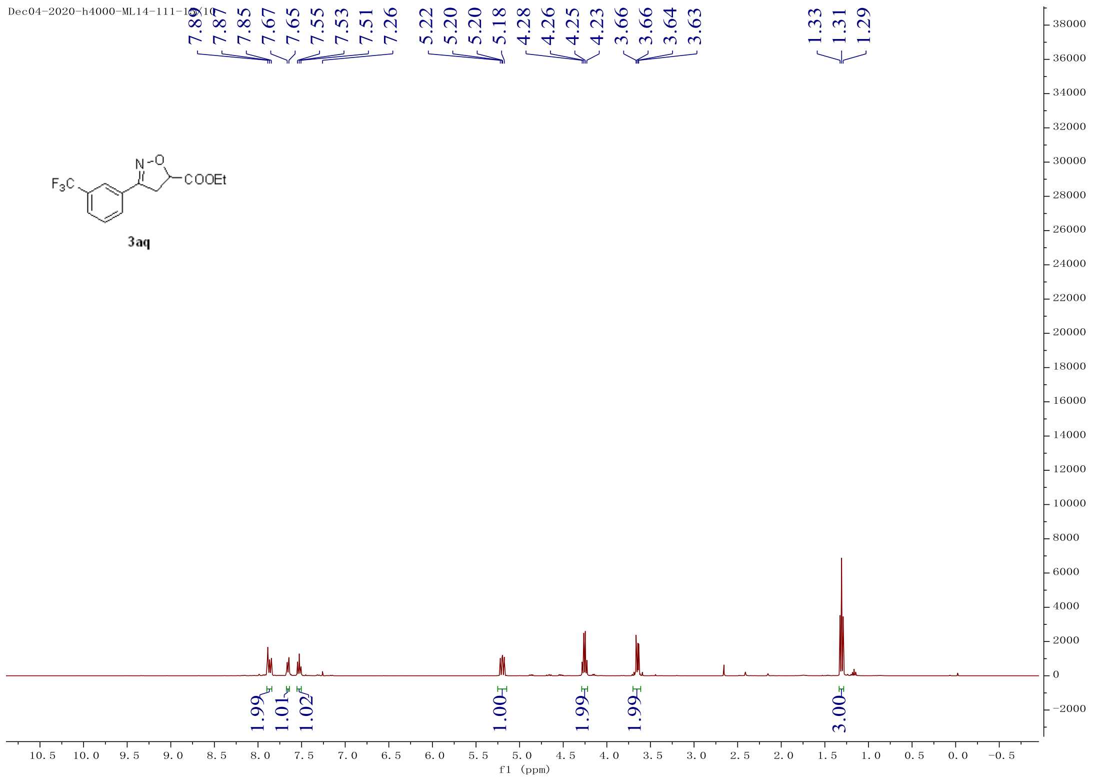
Dec04-2020-h4000-ML14-111-15/10
7.89
7.87
7.85
7.67
7.65
7.55
7.53
7.51
7.26
5.22
5.20
5.20
5.18
4.28
4.26
4.25
4.23
3.66
3.66
3.64
3.63
1.33
1.31
1.29
F3C
N-O
COOEt
3aq
1.99
1.01
1.02
1.00
1.99
1.99
3.00
f1 (ppm)

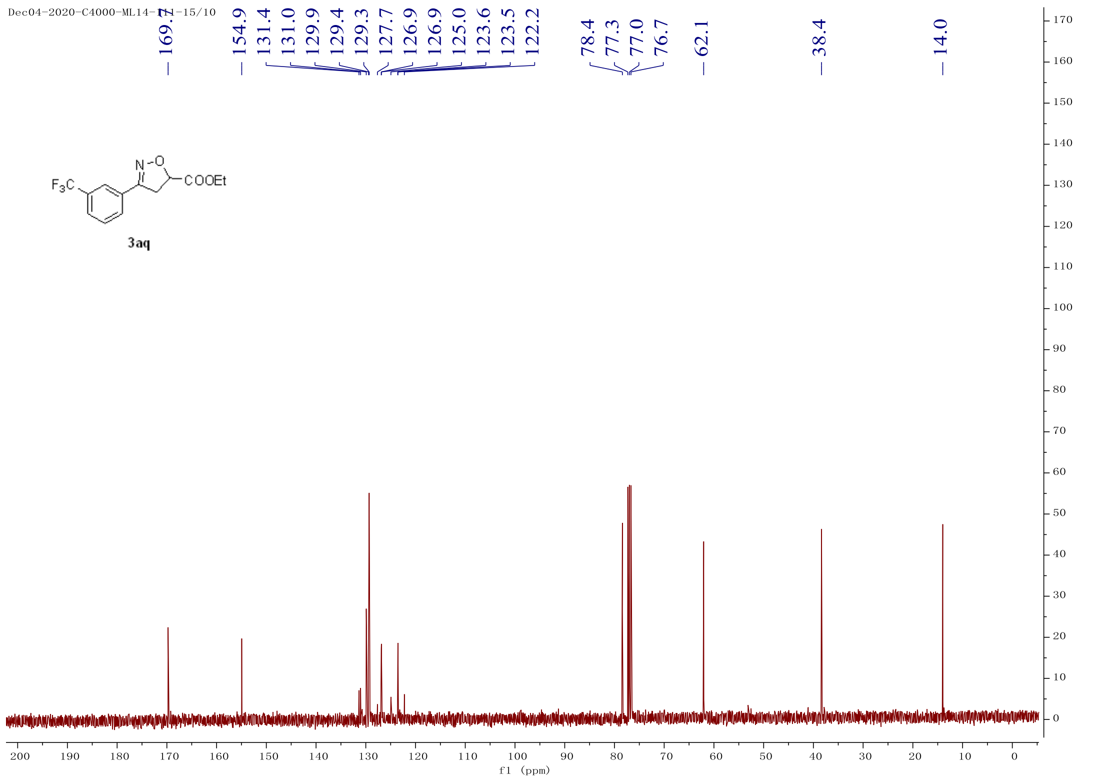
169.7
154.9
131.4
131.0
129.9
129.4
129.3
127.7
126.9
126.9
125.0
123.6
123.5
122.2
78.4
77.3
77.0
76.7
62.1
38.4
14.0
F3C
N-O
COOEt
3aq
f1 (ppm)

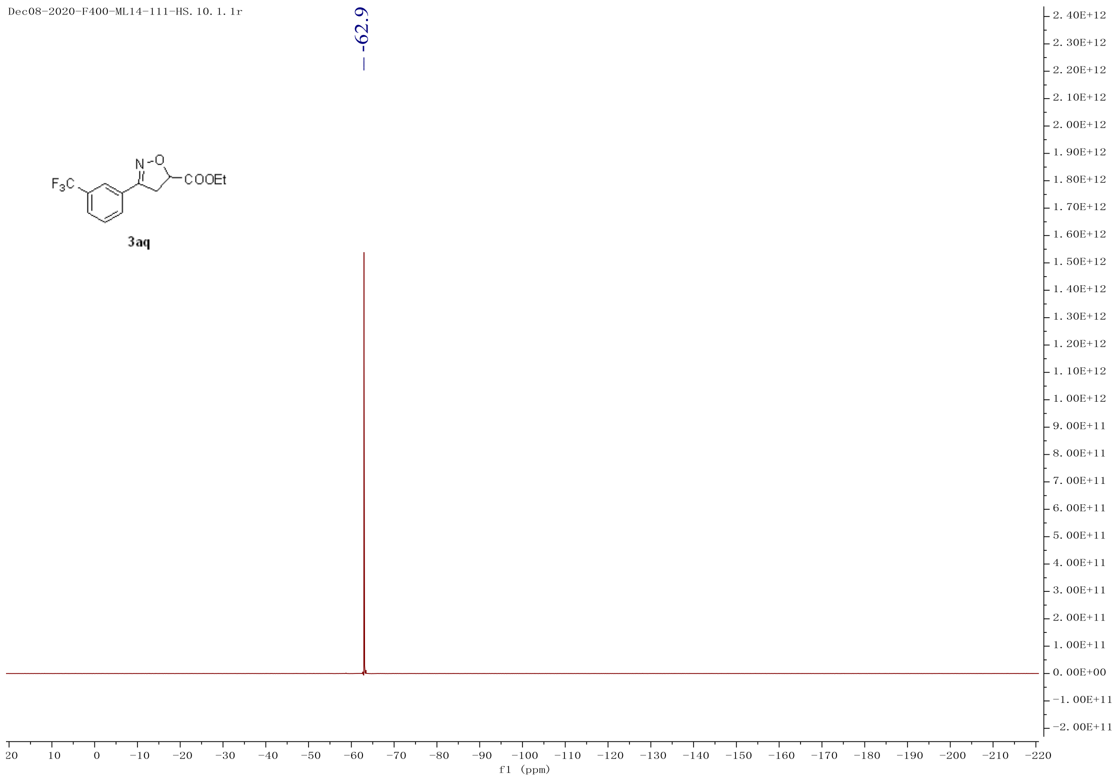

Dec08-2020-F400-ML14-111-HS.10.1.1r
-62.9
F3C
N-O
COOEt
3aq
f1 (ppm)

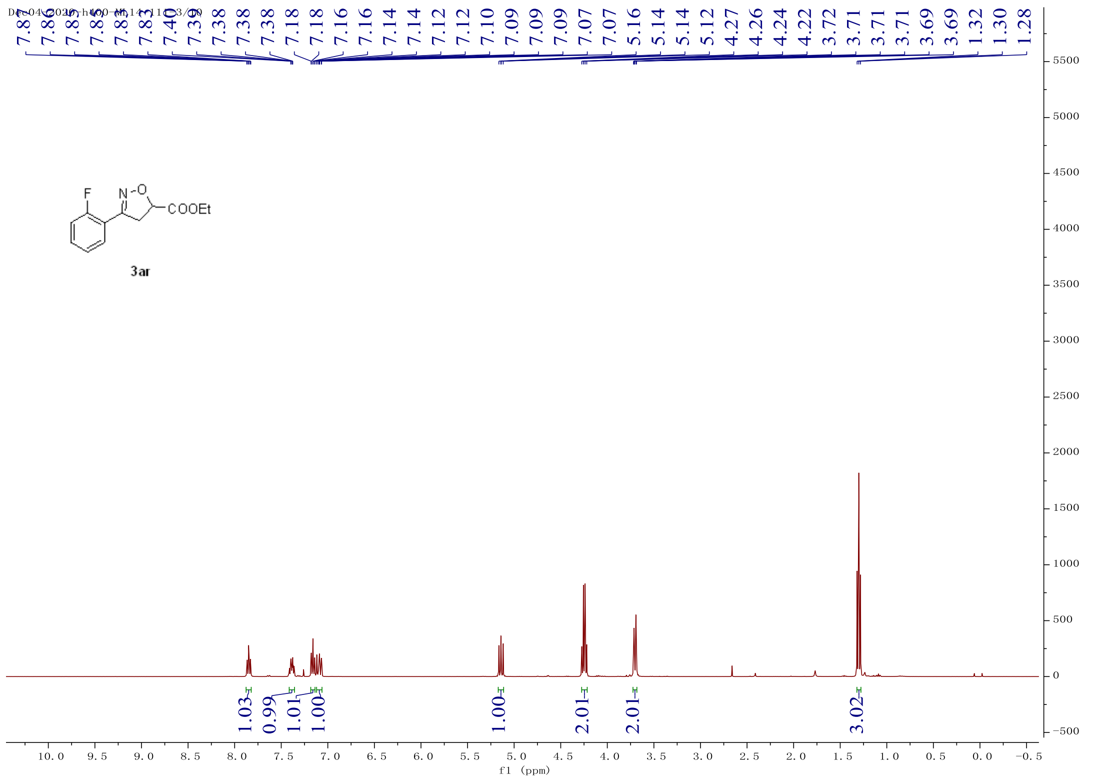

7.87 7.86 7.85 7.85 7.85 7.83 7.40 7.39 7.38 7.38 7.38 7.18 7.18 7.16 7.16 7.14 7.14 7.12 7.12 7.10 7.09 7.09 7.09 7.07 7.07 5.16 5.14 5.14 5.12 4.27 4.26 4.24 4.22 3.72 3.71 3.71 3.71 3.69 3.69 1.32 1.30 1.28
F
N-O
COOEt
3ar
1.03 0.99 1.01 1.00 1.00 2.01 2.01 3.02
f1 (ppm)

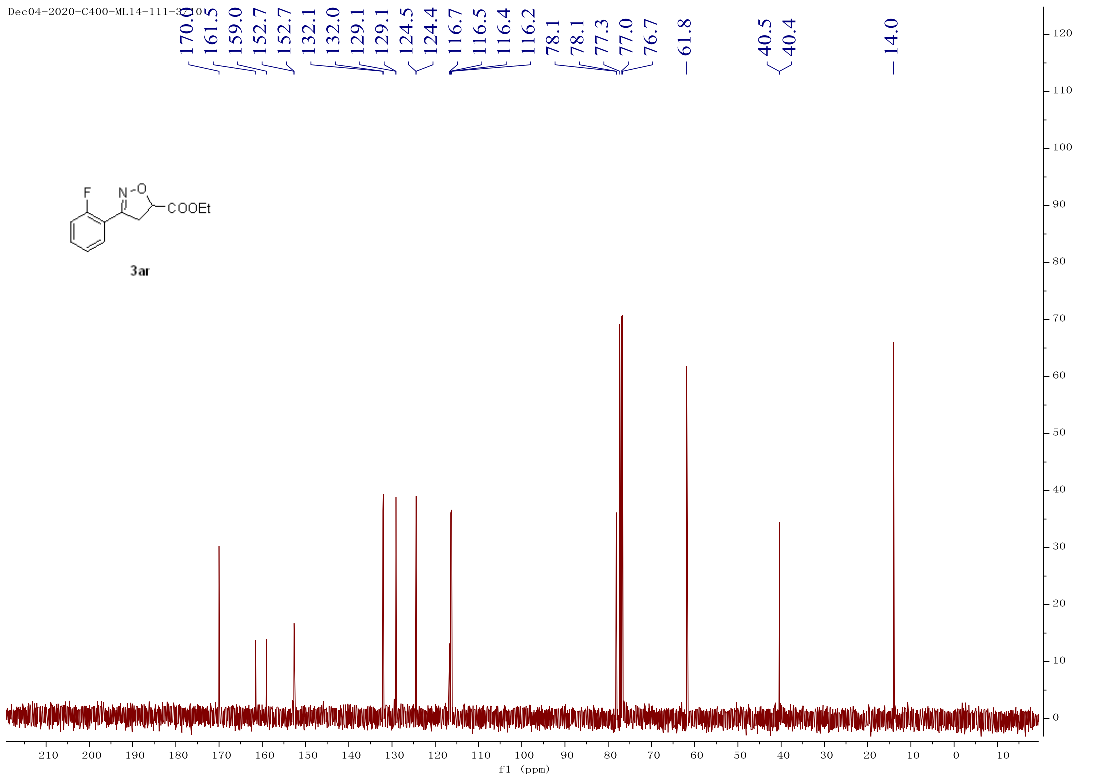
Dec04-2020-C400-ML14-111-3.10
170.0
161.5
159.0
152.7
152.7
132.1
132.0
129.1
129.1
124.5
124.4
116.7
116.5
116.4
116.2
78.1
78.1
77.3
77.0
76.7
61.8
40.5
40.4
14.0
F
N-O
COOEt
3ar
f1 (ppm)

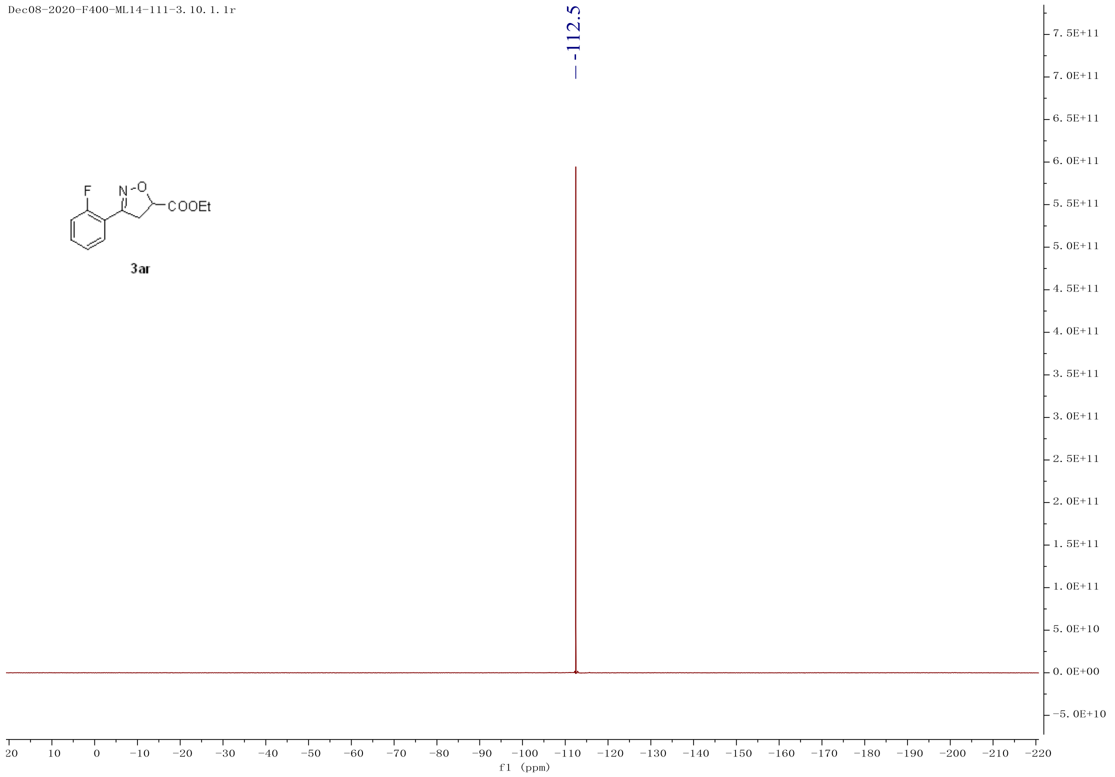
Dec08-2020-F400-ML14-111-3.10.1.1r
-112.5
F
N-O
COOEt
3ar
f1 (ppm)

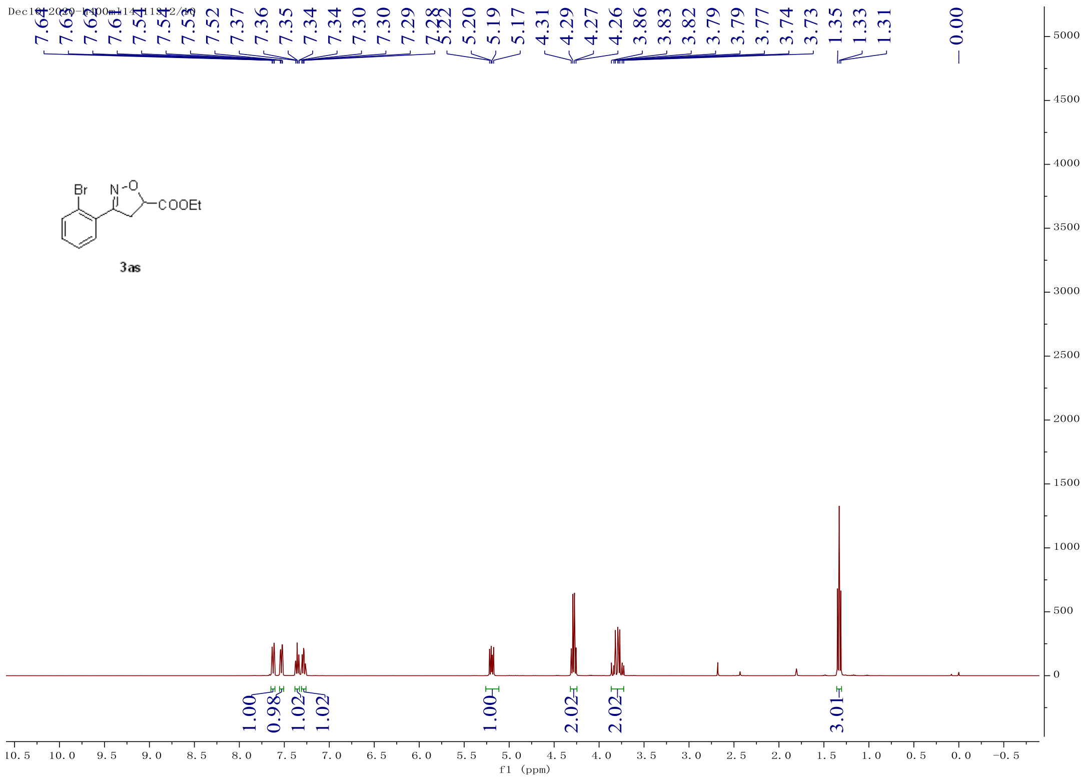
Dec10-2020-c400ml14-113-2/10
7.64
7.62
7.62
7.61
7.54
7.54
7.53
7.52
7.37
7.36
7.35
7.34
7.34
7.30
7.30
7.29
7.28
5.22
5.20
5.19
5.17
4.31
4.29
4.27
4.26
3.86
3.83
3.82
3.79
3.79
3.77
3.74
3.73
1.35
1.33
1.31
0.00
Br
N-O
COOEt
3as
1.00
0.98
1.02
1.02
1.00
2.02
2.02
3.01
f1 (ppm)

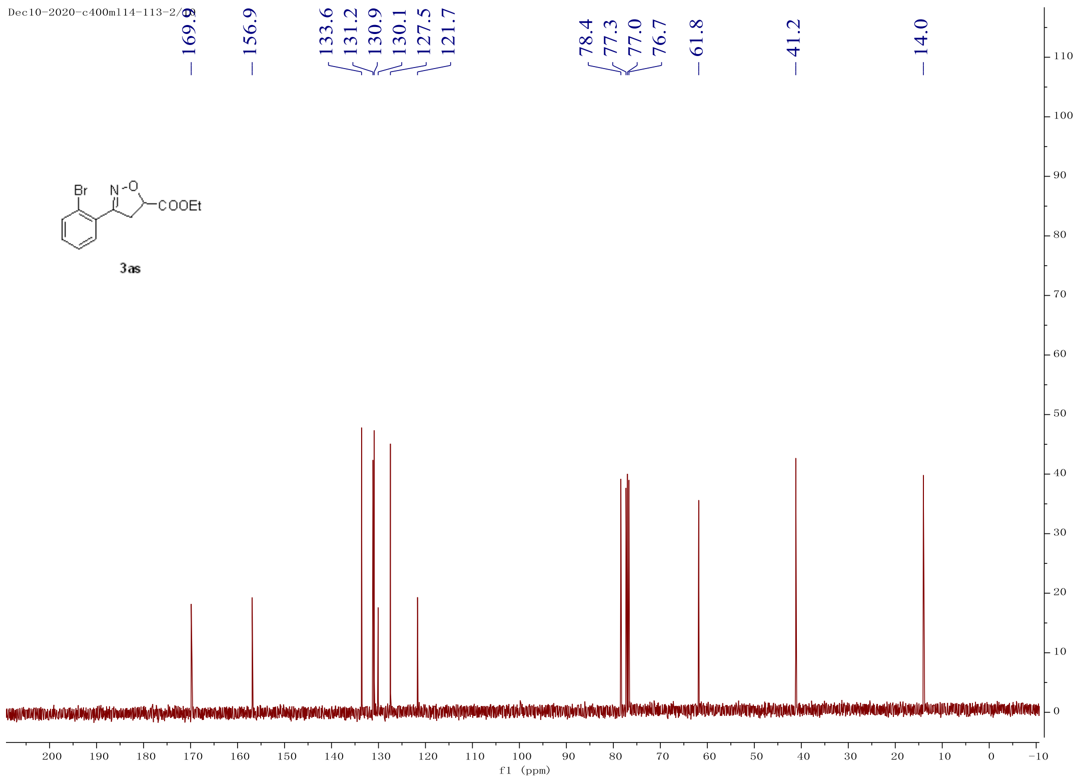
Dec10-2020-c400ml14-113-2/10
169.9
156.9
133.6
131.2
130.9
130.1
127.5
121.7
78.4
77.3
77.0
76.7
61.8
41.2
14.0
Br
N-O
COOEt
3as
f1 (ppm)

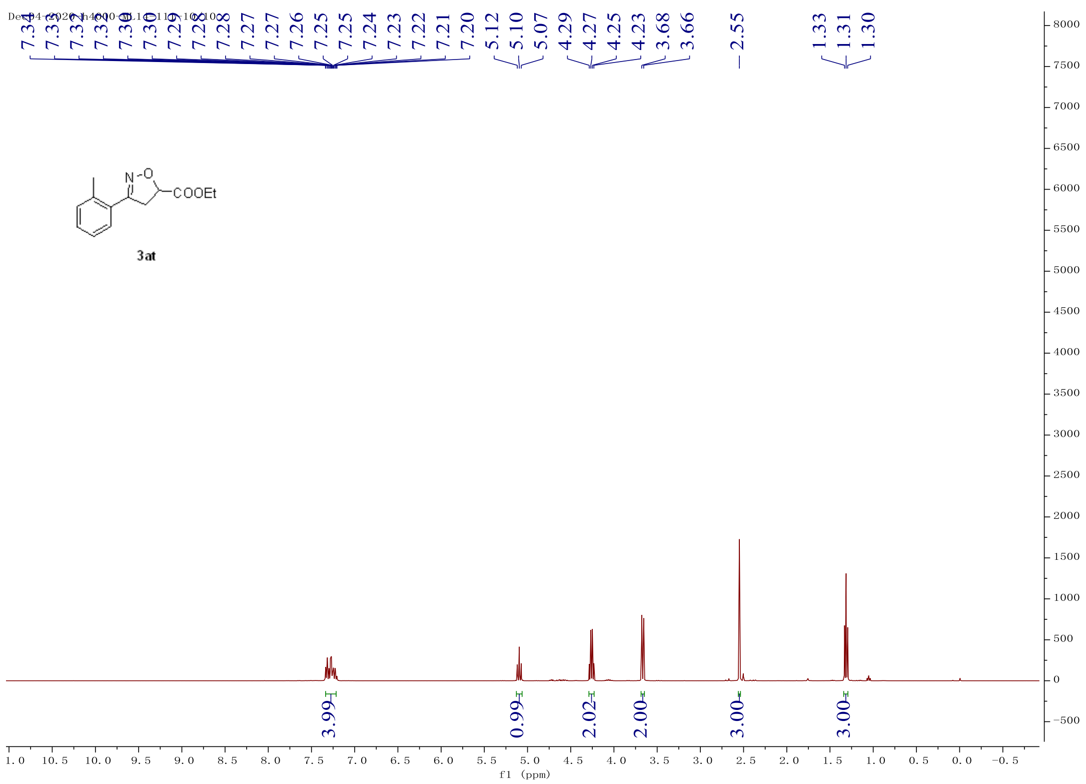
Dec04-2020-H4000-ML14-111-10/10
7.34
7.31
7.31
7.31
7.30
7.30
7.29
7.28
7.28
7.27
7.27
7.26
7.25
7.25
7.24
7.23
7.22
7.21
7.20
5.12
5.10
5.07
4.29
4.27
4.25
4.23
3.68
3.66
2.55
1.33
1.31
1.30
3.99
0.99
2.02
2.00
3.00
3.00
COOEt
3at
f1 (ppm)

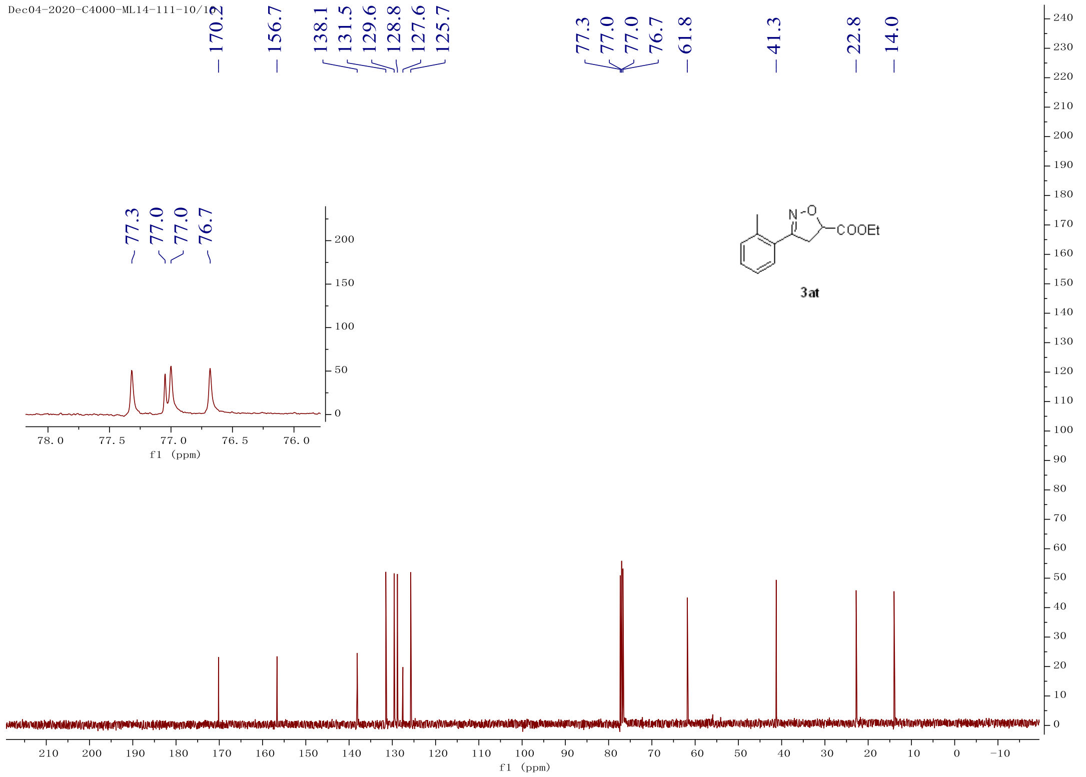
Dec04-2020-C4000-ML14-111-10/10
170.2
156.7
138.1
131.5
129.6
128.8
127.6
125.7
77.3
77.0
77.0
76.7
61.8
41.3
22.8
14.0
COOEt
3at
f1 (ppm)

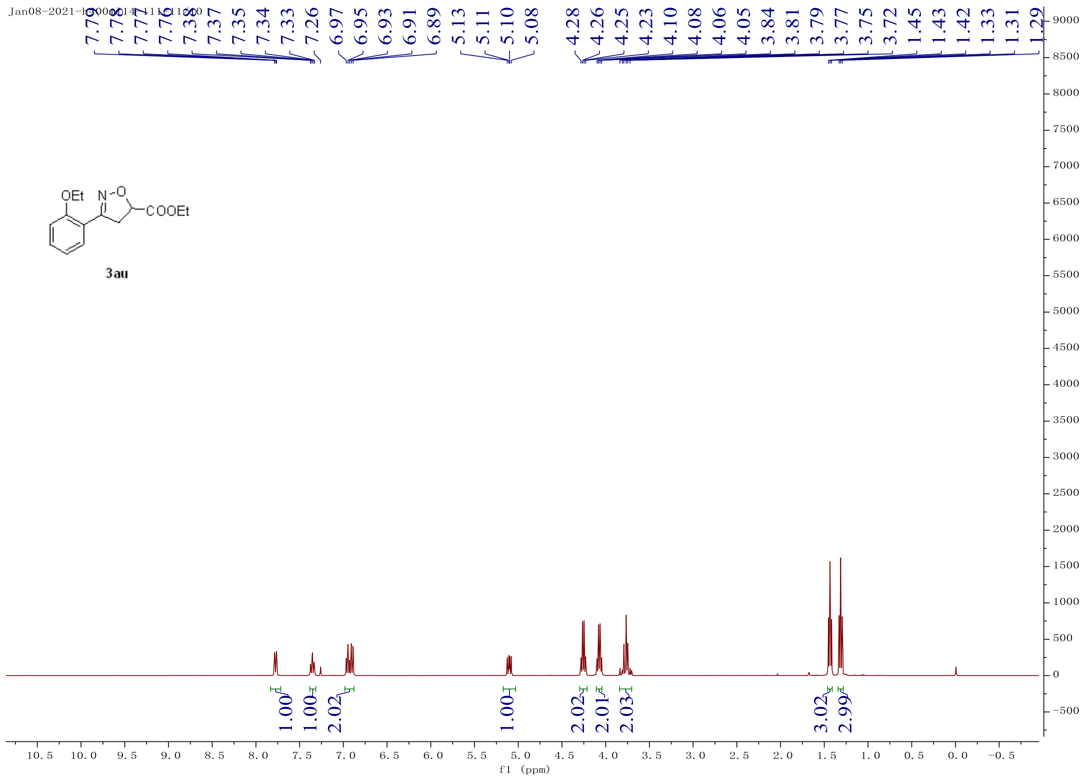
Jan08-2021-H000-ML14-111-11/10
7.79 7.77 7.77 7.76 7.38 7.37 7.35 7.34 7.33 7.26 6.97 6.95 6.93 6.91 6.89 5.13 5.11 5.10 5.08 4.28 4.26 4.25 4.23 4.10 4.08 4.06 4.05 3.84 3.81 3.79 3.77 3.75 3.72 1.45 1.43 1.42 1.33 1.31 1.29
OEt
N-O
COOEt
3au
1.00 1.00 2.02 1.00 2.02 2.01 2.03 3.02 2.99
f1 (ppm)

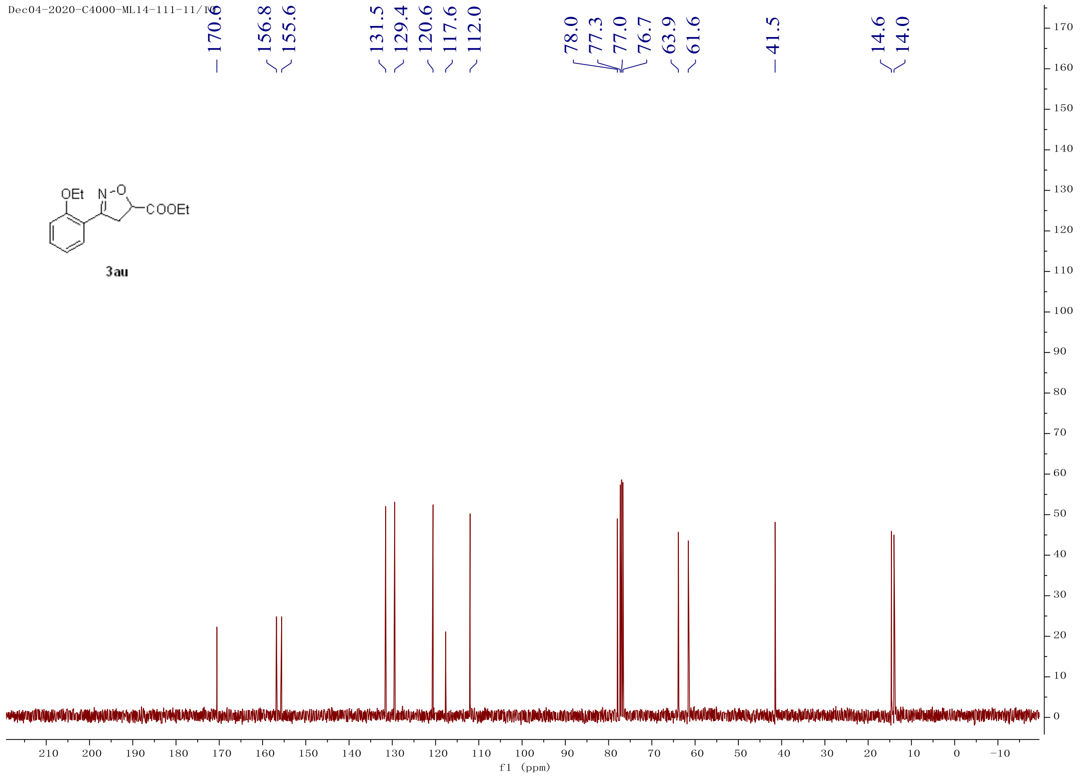
Dec04-2020-C4000-ML14-111-11/10
170.6 156.8 155.6 131.5 129.4 120.6 117.6 112.0 78.0 77.3 77.0 76.7 63.9 61.6 41.5 14.6 14.0
OEt
N-O
COOEt
3au
f1 (ppm)

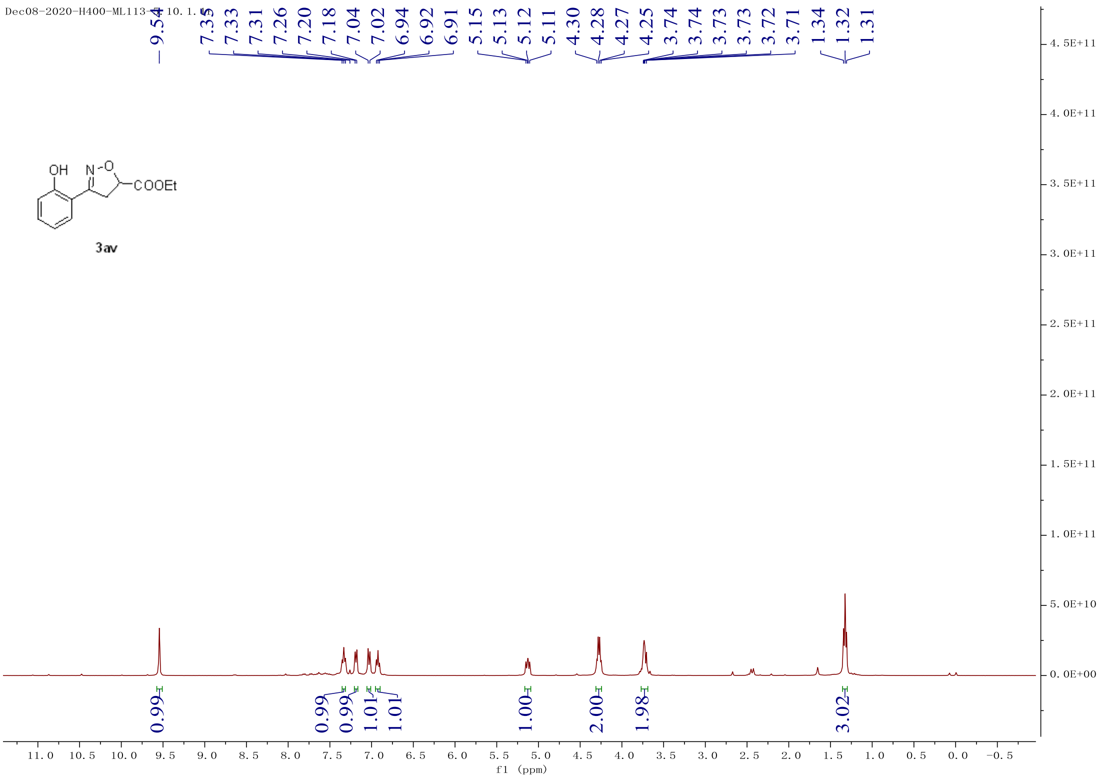
Dec08-2020-H400-ML113-5.10.1.1r
9.54
7.35 7.33 7.31 7.26 7.20 7.18 7.04 7.02 6.94 6.92 6.91
5.15 5.13 5.12 5.11
4.30 4.28 4.27 4.25
3.74 3.74 3.73 3.73 3.72 3.71
1.34 1.32 1.31
OH
N-O
COOEt
3av
0.99 0.99 0.99 1.01 1.01 1.00 2.00 1.98 3.02
f1 (ppm)

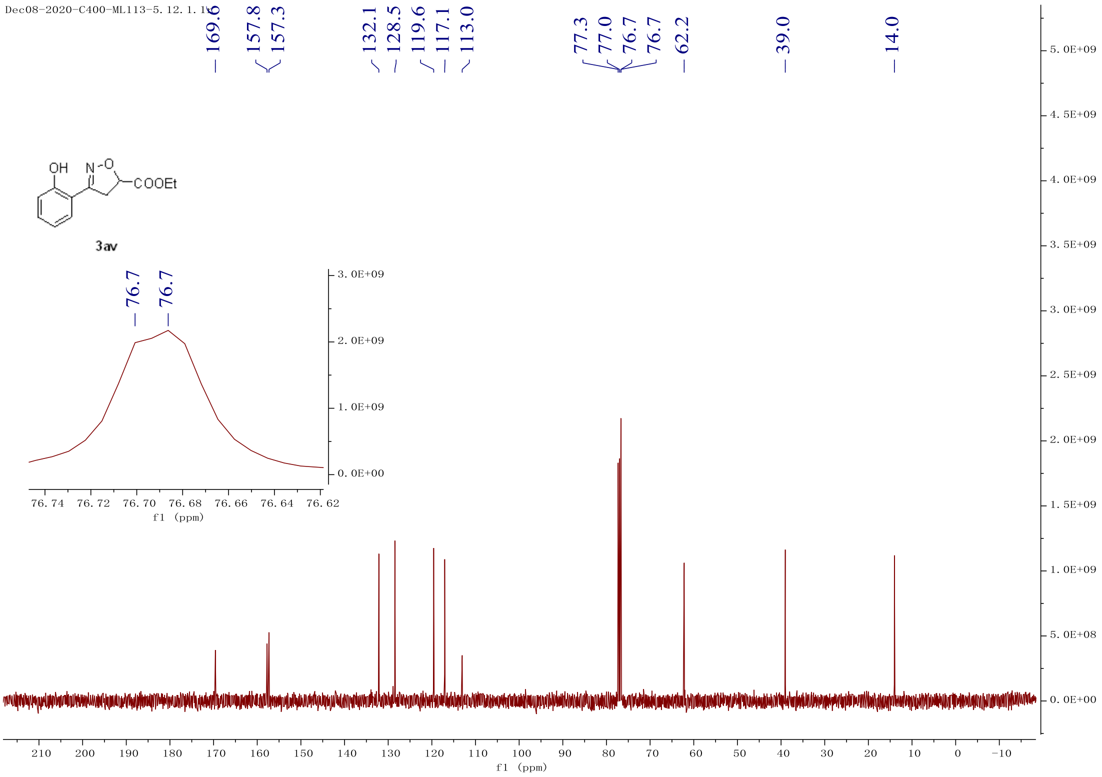
Dec08-2020-C400-ML113-5.12.1.1r
169.6
157.8 157.3
132.1 128.5 119.6 117.1 113.0
77.3 77.0 76.7 76.7
62.2
39.0
14.0
OH
N-O
COOEt
3av
76.7 76.7
f1 (ppm)

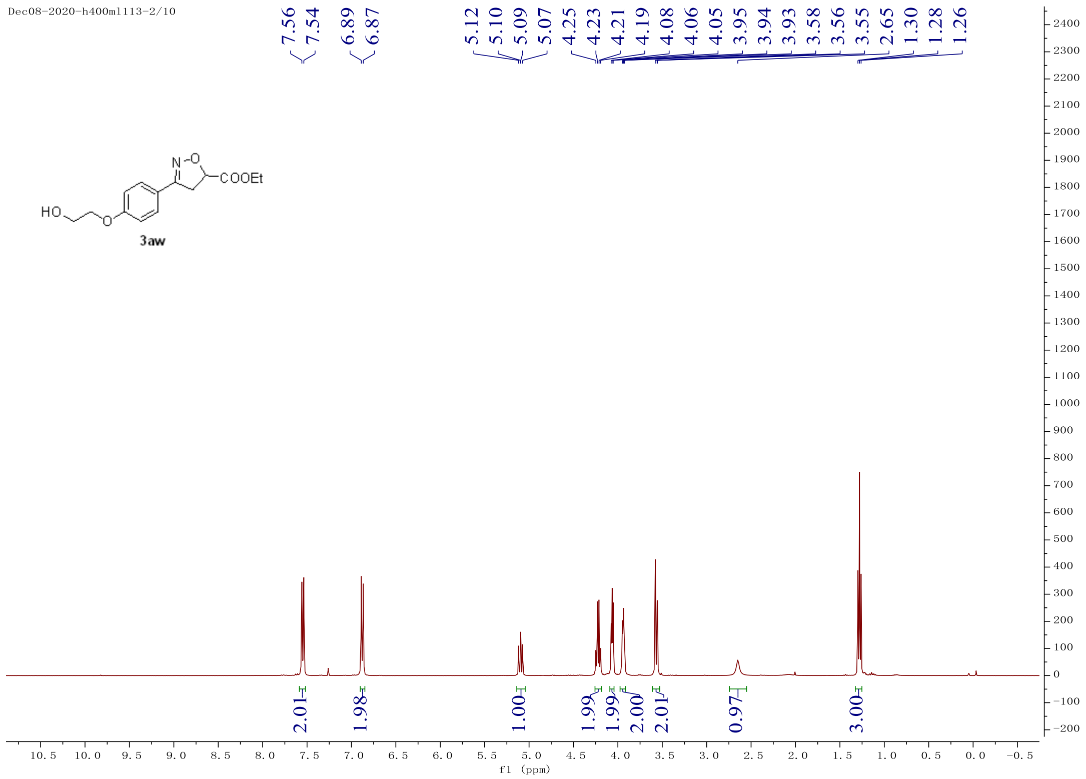
Dec08-2020-h400ml113-2/10
7.56 7.54 6.89 6.87 5.12 5.10 5.09 5.07 4.25 4.23 4.21 4.19 4.08 4.06 4.05 3.95 3.94 3.93 3.58 3.56 3.55 2.65 1.30 1.28 1.26
N-O
COOEt
HO
O
3aw
2.01 1.98 1.00 1.99 1.99 2.00 2.01 0.97 3.00
f1 (ppm)

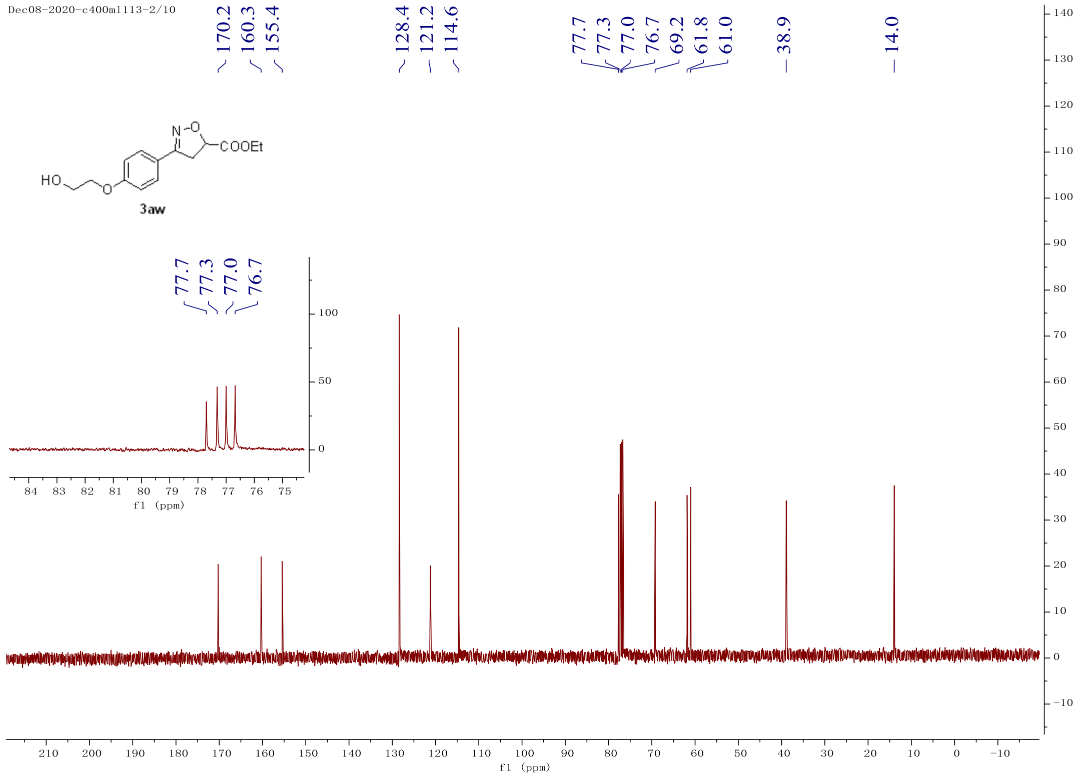
Dec08-2020-c400ml113-2/10
170.2 160.3 155.4 128.4 121.2 114.6 77.7 77.3 77.0 76.7 69.2 61.8 61.0 38.9 14.0
N-O
COOEt
HO
O
3aw
77.7 77.3 77.0 76.7
f1 (ppm)

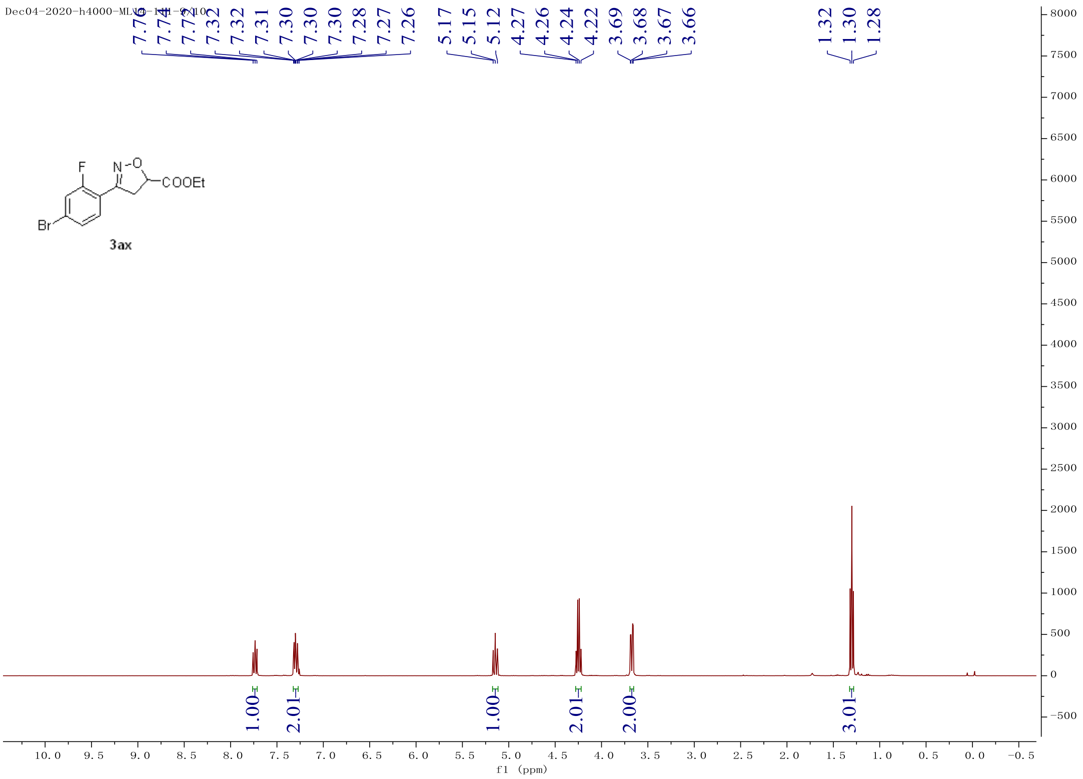
Dec04-2020-h4000-ML14-14-9-10
7.76
7.74
7.72
7.32
7.31
7.30
7.30
7.30
7.28
7.27
7.26
5.17
5.15
5.12
4.27
4.26
4.24
4.22
3.69
3.68
3.67
3.66
1.32
1.30
1.28
3ax
1.00
2.01
1.00
2.01
2.00
3.01
f1 (ppm)

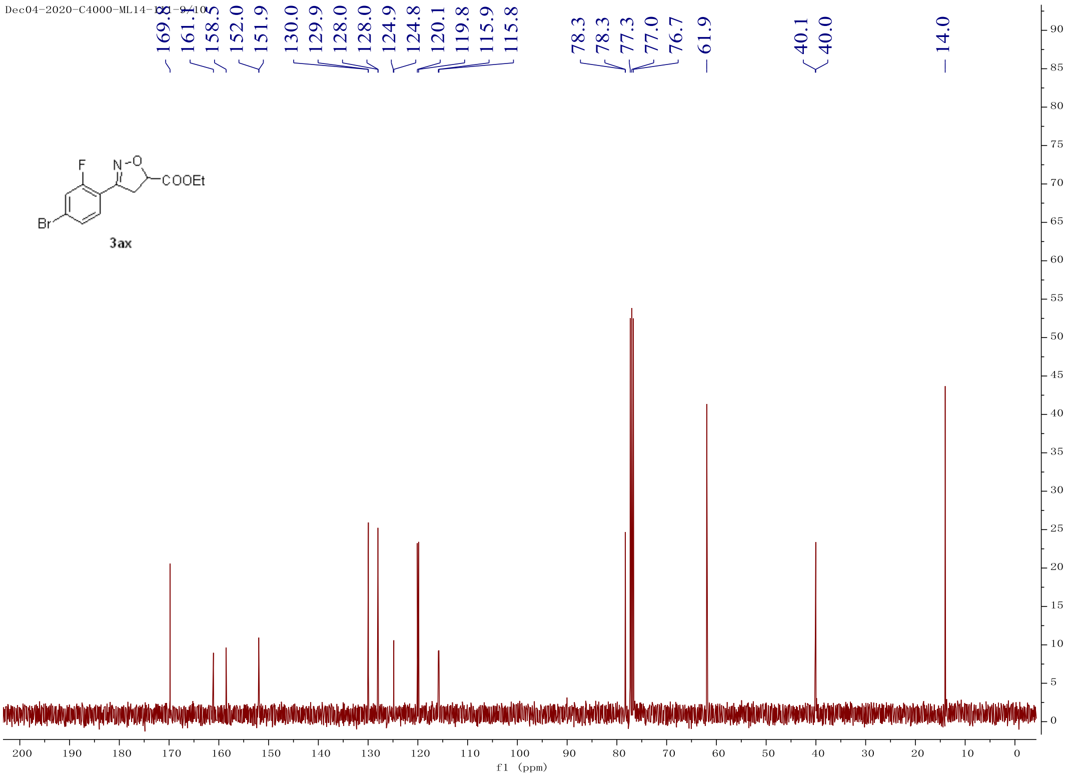
Dec04-2020-C4000-ML14-14-9-10
169.8
161.1
158.5
152.0
151.9
130.0
129.9
128.0
128.0
124.9
124.8
120.1
119.8
115.9
115.8
78.3
78.3
77.3
77.0
76.7
61.9
40.1
40.0
14.0
3ax
f1 (ppm)

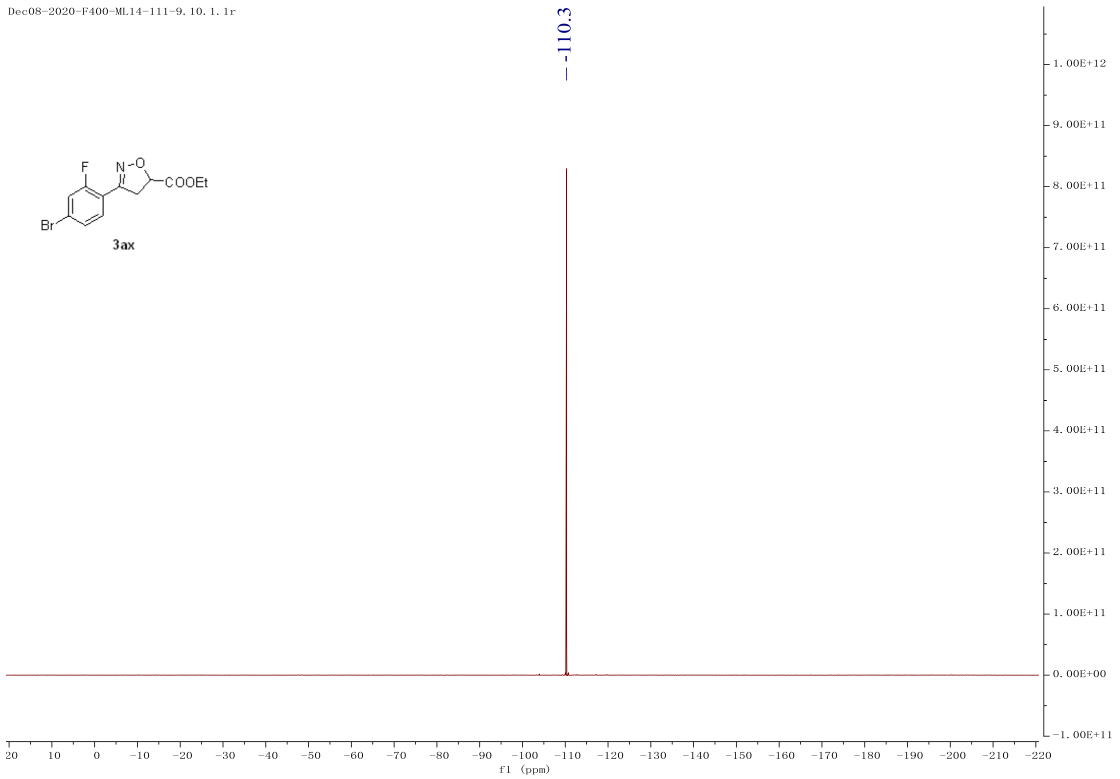
Dec08-2020-F400-ML14-111-9.10.1.1r
-110.3
F
Br
N-O
COOEt
3ax
f1 (ppm)

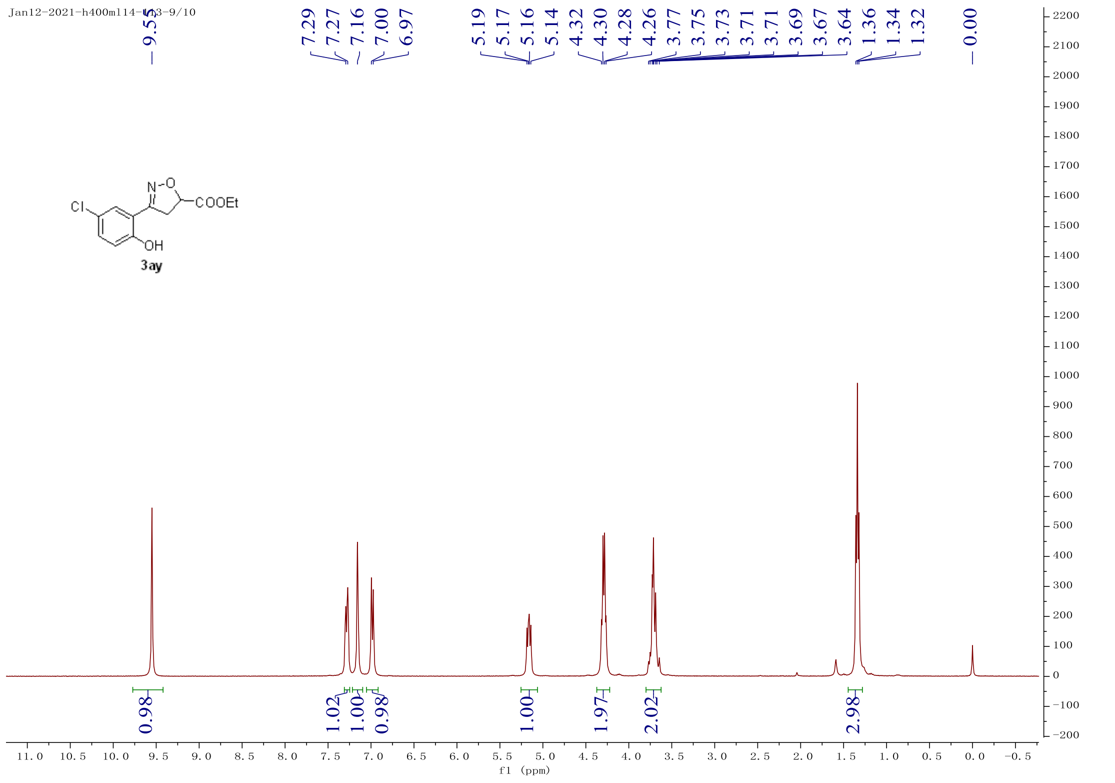
Jan12-2021-h400ml14-113-9/10
9.55
7.29
7.27
7.16
7.00
6.97
5.19
5.17
5.16
5.14
4.32
4.30
4.28
4.26
3.77
3.75
3.73
3.71
3.69
3.67
3.64
1.36
1.34
1.32
0.00
Cl
N-O
COOEt
OH
3ay
0.98
1.02
1.00
0.98
1.00
1.97
2.02
2.98
f1 (ppm)

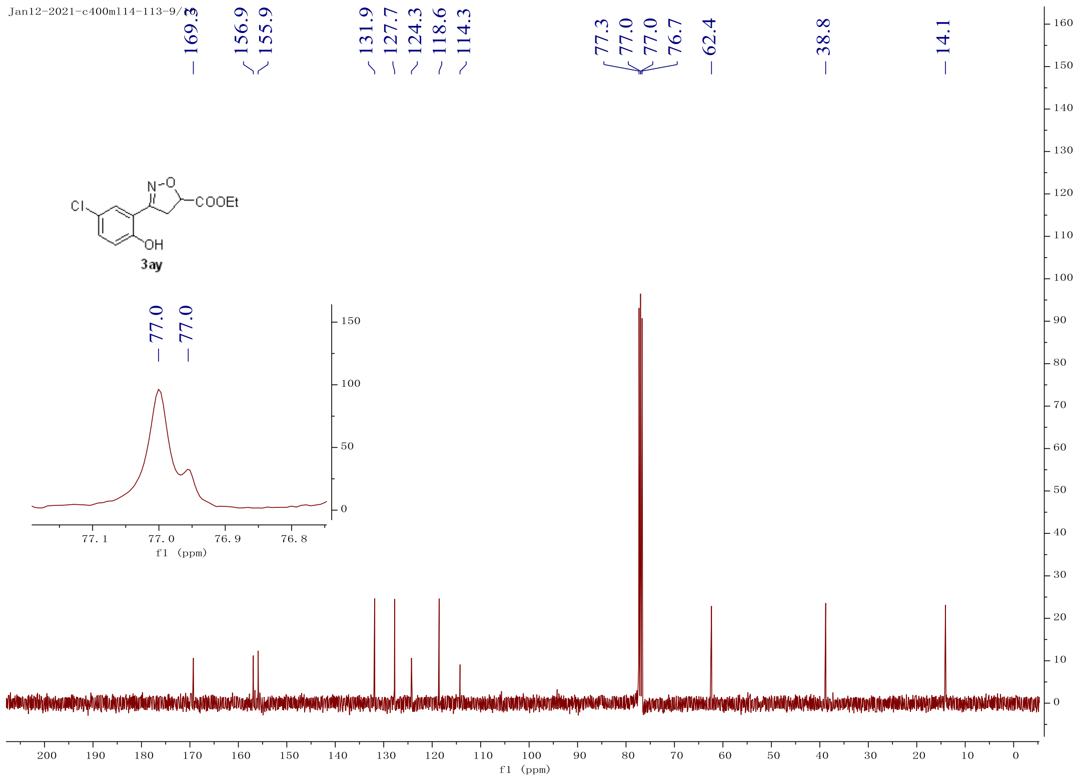
Jan12-2021-c400ml14-113-9/3
169.3
156.9
155.9
131.9
127.7
124.3
118.6
114.3
77.3
77.0
77.0
76.7
62.4
38.8
14.1
Cl
N-O
COOEt
OH
3ay
f1 (ppm)

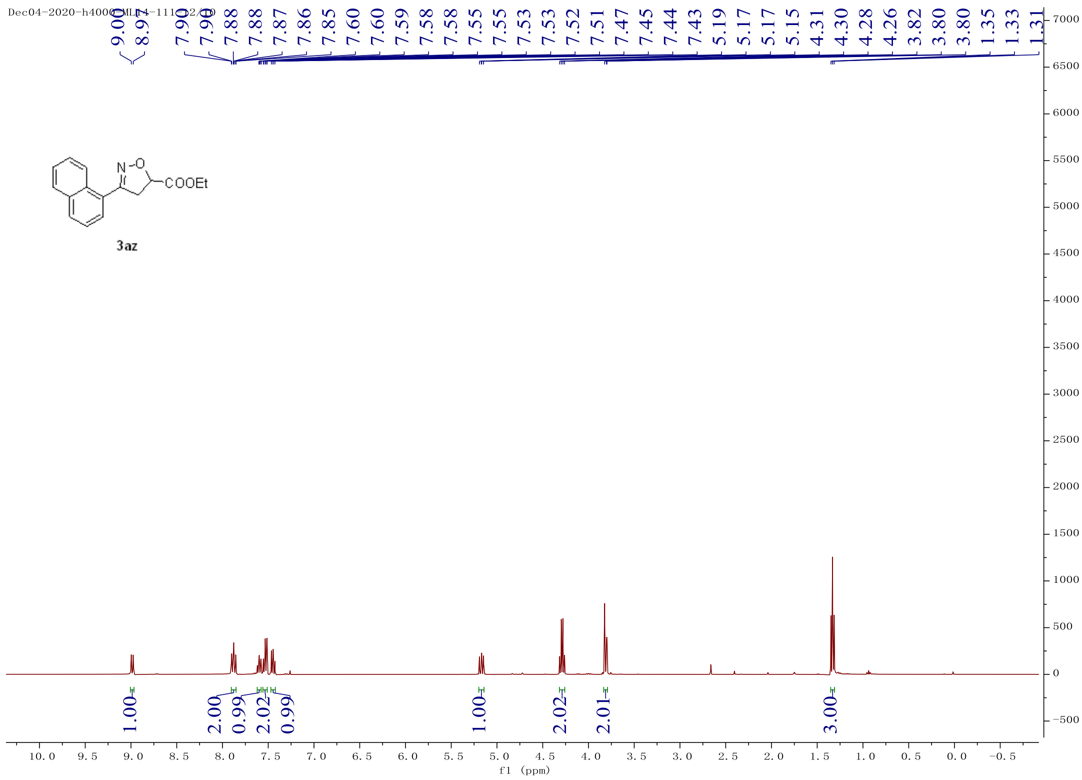
N-O
COOEt
3az
9.00
8.98
7.90
7.90
7.88
7.87
7.86
7.85
7.60
7.60
7.59
7.58
7.58
7.55
7.55
7.53
7.53
7.52
7.51
7.47
7.45
7.44
7.43
5.19
5.17
5.17
5.15
4.31
4.30
4.28
4.26
3.82
3.80
1.35
1.33
1.31
1.00
2.00
0.99
2.02
0.99
1.00
2.02
2.01
3.00
f1 (ppm)

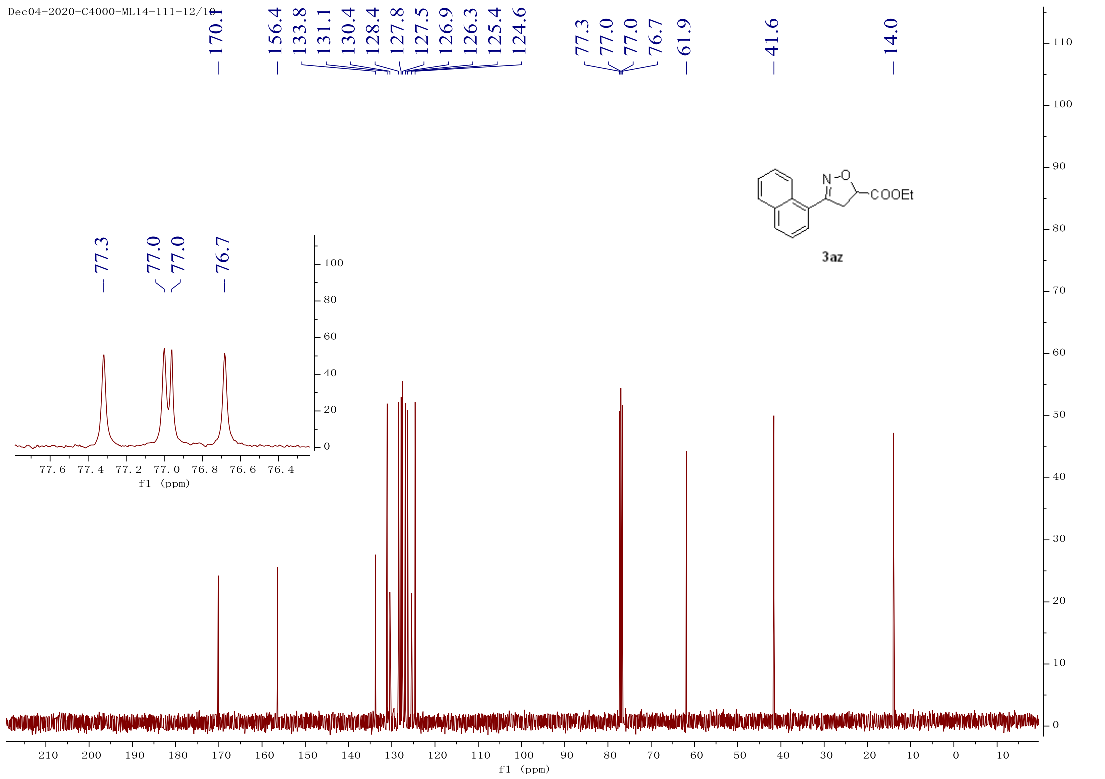
Dec04-2020-C4000-ML14-111-12/10
170.1
156.4
133.8
131.1
130.4
128.4
127.8
127.5
126.9
126.3
125.4
124.6
77.3
77.0
77.0
76.7
61.9
41.6
14.0
COOEt
3az
f1 (ppm)

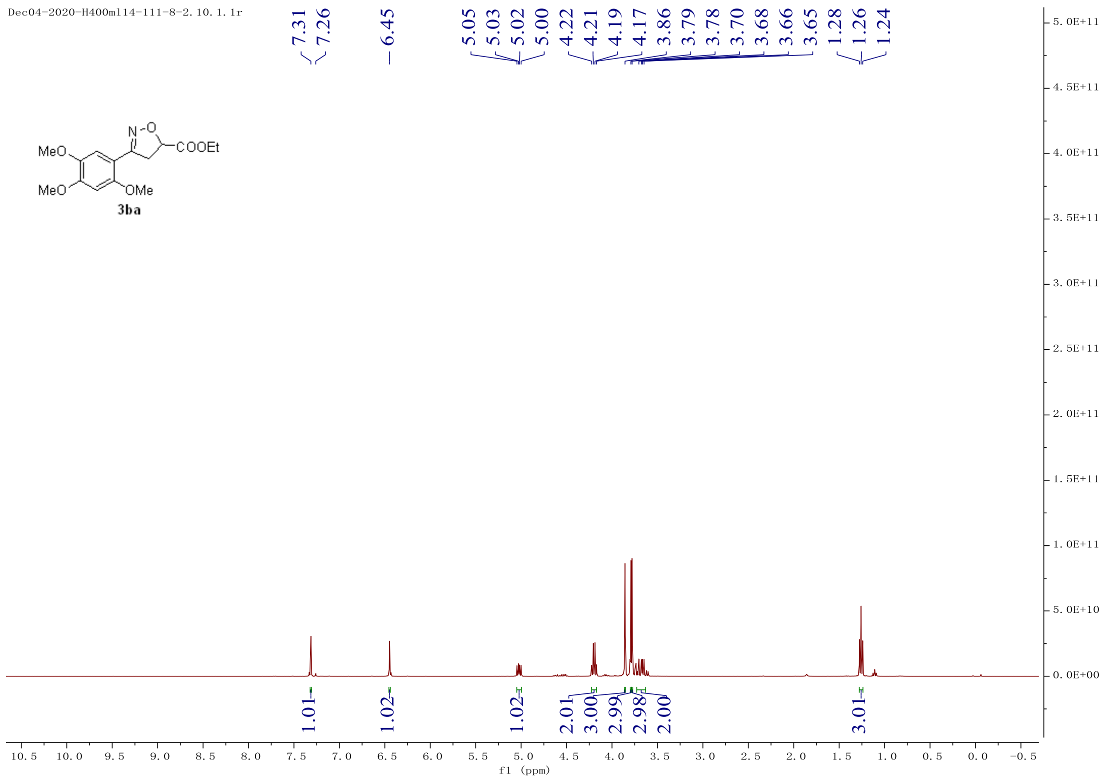
Dec04-2020-H400ml14-111-8-2.10.1.1r
7.31
7.26
6.45
5.05
5.03
5.02
5.00
4.22
4.21
4.19
4.17
3.86
3.79
3.78
3.70
3.68
3.66
3.65
1.28
1.26
1.24
MeO
MeO
OMe
COOEt
3ba
1.01
1.02
1.02
2.01
3.00
2.99
2.98
2.00
3.01
f1 (ppm)

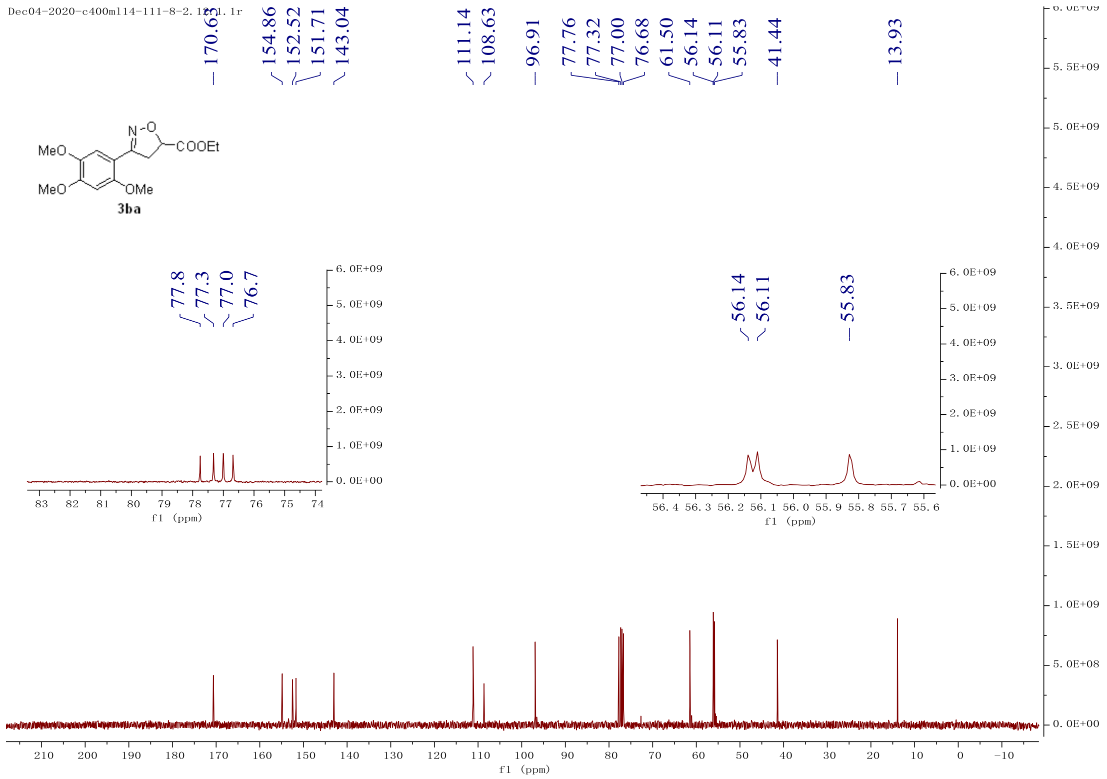

Dec04-2020-c400ml14-111-8-2.1[illegible]1.1r
170.63
154.86
152.52
151.71
143.04
111.14
108.63
96.91
77.76
77.32
77.00
76.68
61.50
56.14
56.11
55.83
41.44
13.93
MeO
MeO
OMe
N-O
COOEt
3ba
77.8
77.3
77.0
76.7
56.14
56.11
55.83
f1 (ppm)

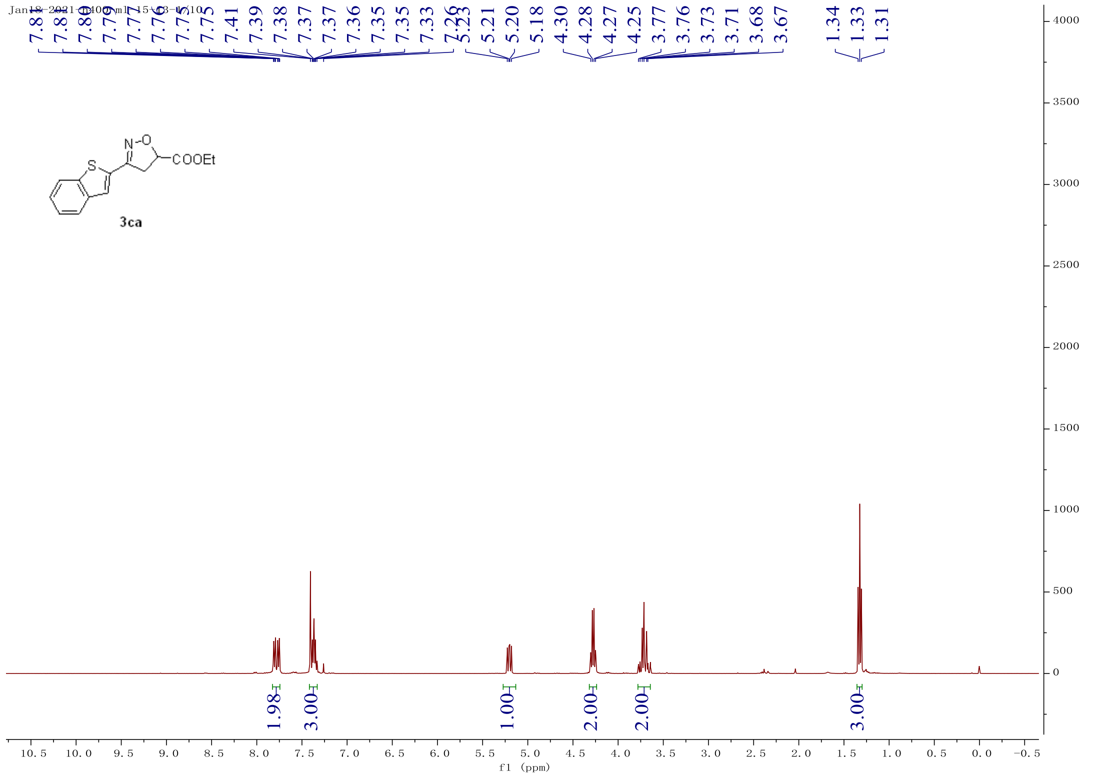

Jan18-2021-[illegible]
[illegible]
[illegible]
7.80
7.79
7.77
7.76
7.75
7.75
7.41
7.39
7.38
7.37
7.37
7.36
7.35
7.35
7.33
7.26
5.23
5.21
5.20
5.18
4.30
4.28
4.27
4.25
3.77
3.76
3.73
3.71
3.68
3.67
1.34
1.33
1.31
S
N-O
COOEt
3ca
1.98
3.00
1.00
2.00
2.00
3.00
f1 (ppm)

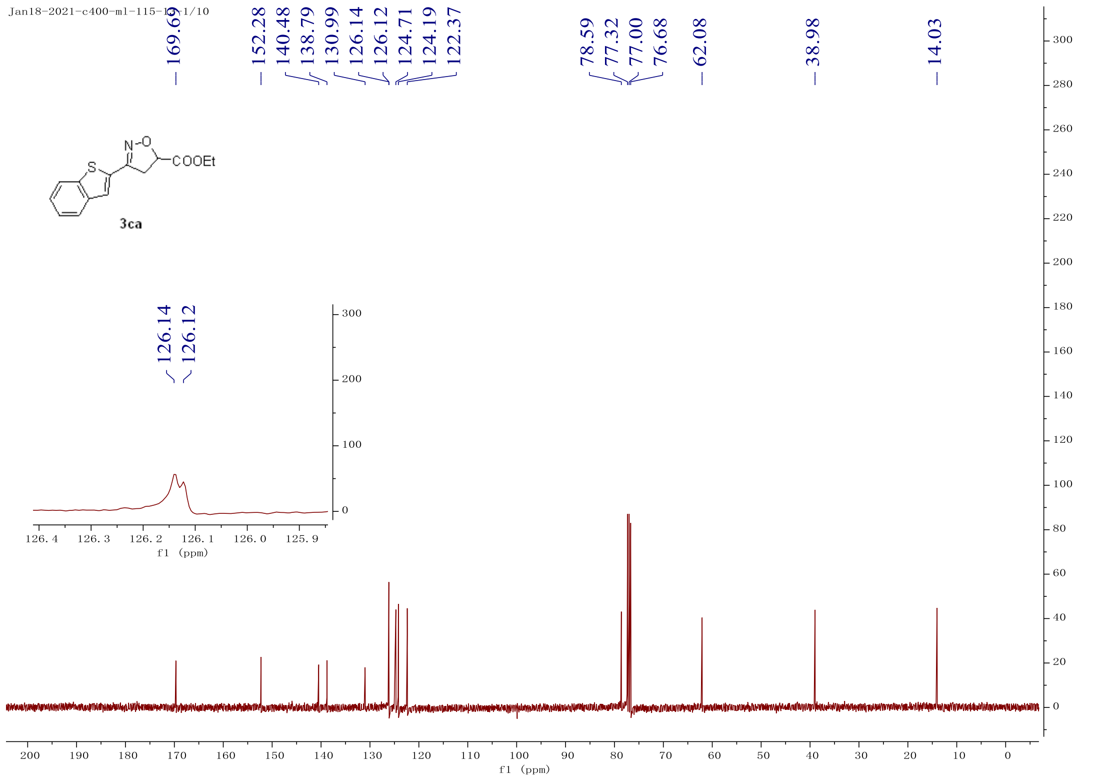

169.69
152.28
140.48
138.79
130.99
126.14
126.12
124.71
124.19
122.37
78.59
77.32
77.00
76.68
62.08
38.98
14.03
COOEt
3ca
f1 (ppm)

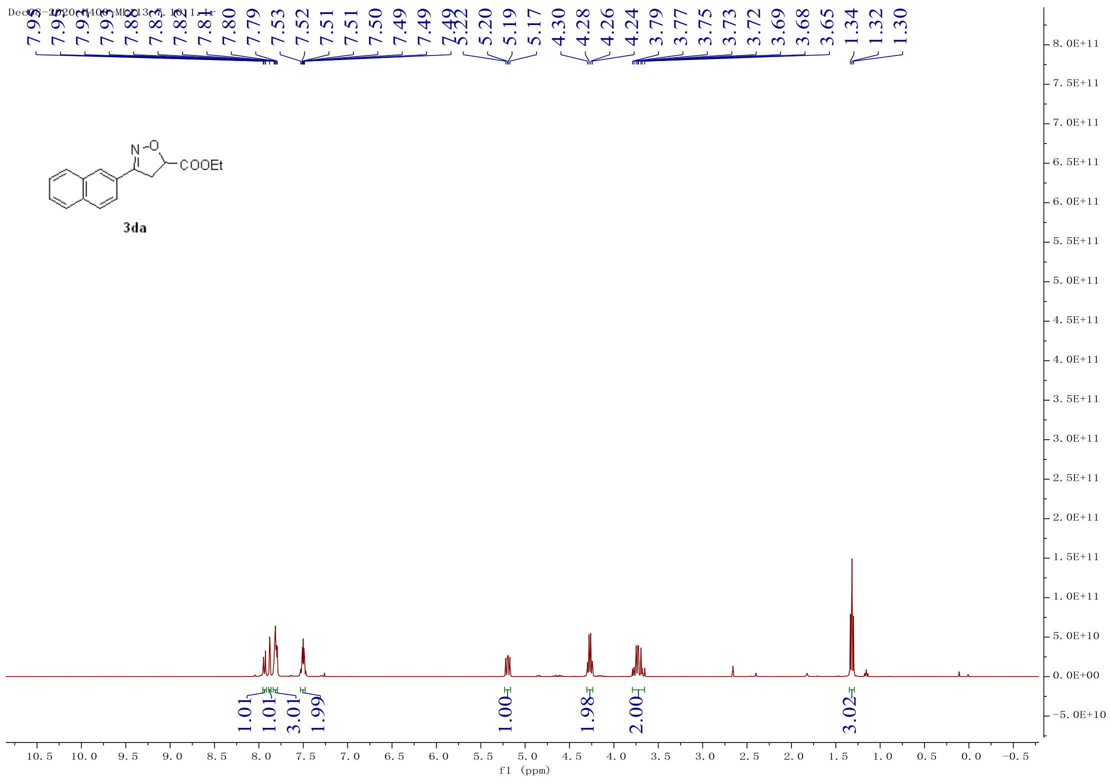

7.95
7.93
7.91
7.88
7.83
7.82
7.81
7.80
7.79
7.53
7.52
7.51
7.51
7.50
7.49
7.49
5.22
5.20
5.19
5.17
4.30
4.28
4.26
4.24
3.79
3.77
3.75
3.73
3.72
3.69
3.68
3.65
1.34
1.32
1.30
COOEt
3da
1.01
1.01
3.01
1.99
1.00
1.98
2.00
3.02
f1 (ppm)

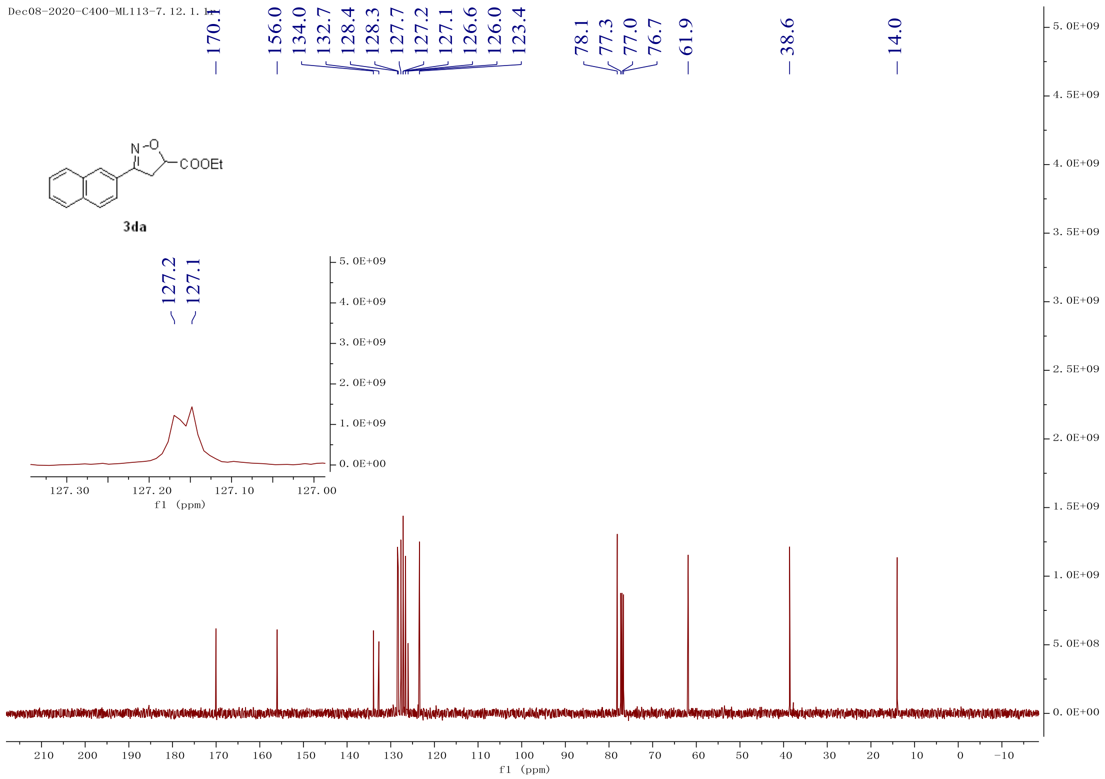
Dec08-2020-C400-ML113-7.12.1.1
170.1
156.0
134.0
132.7
128.4
128.3
127.7
127.2
127.1
126.6
126.0
123.4
78.1
77.3
77.0
76.7
61.9
38.6
14.0
COOEt
3da
127.2
127.1
f1 (ppm)
f1 (ppm)

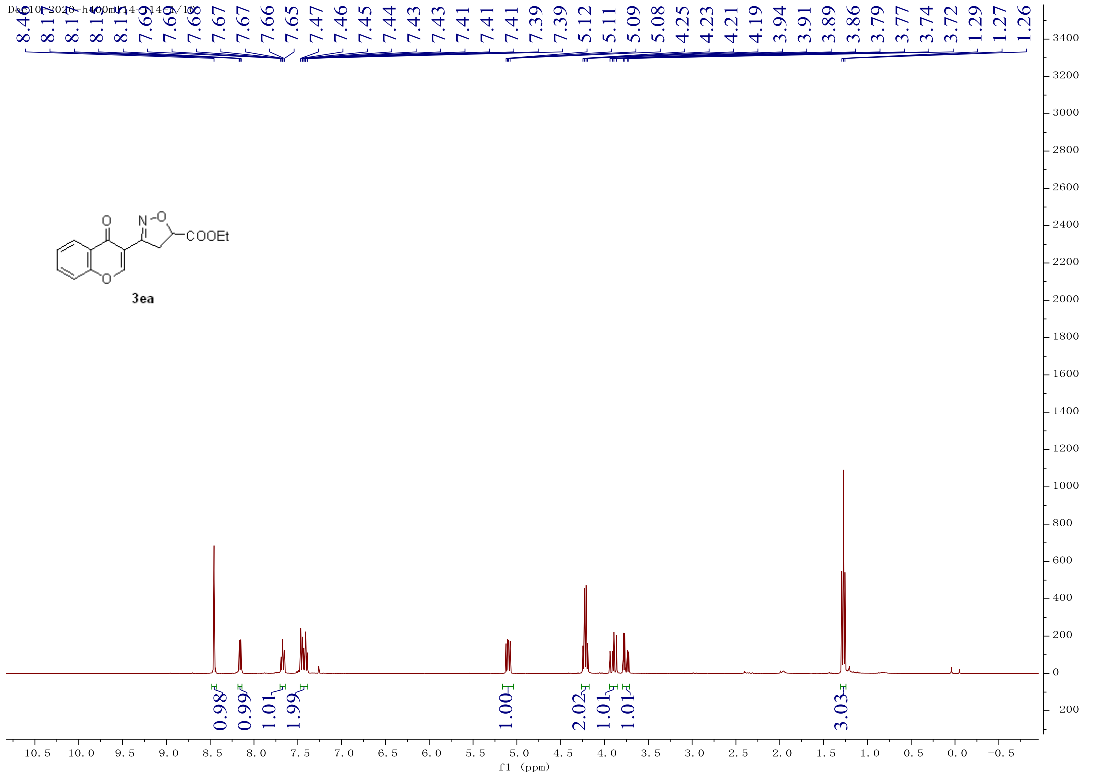
7.69
7.69
7.68
7.67
7.67
7.66
7.65
7.47
7.46
7.45
7.44
7.43
7.43
7.41
7.41
7.41
7.39
7.39
5.12
5.11
5.09
5.08
4.25
4.23
4.21
4.19
3.94
3.91
3.89
3.86
3.79
3.77
3.74
3.72
1.29
1.27
1.26
COOEt
3ea
0.98
0.99
1.01
1.99
1.00
2.02
1.01
1.01
3.03
f1 (ppm)

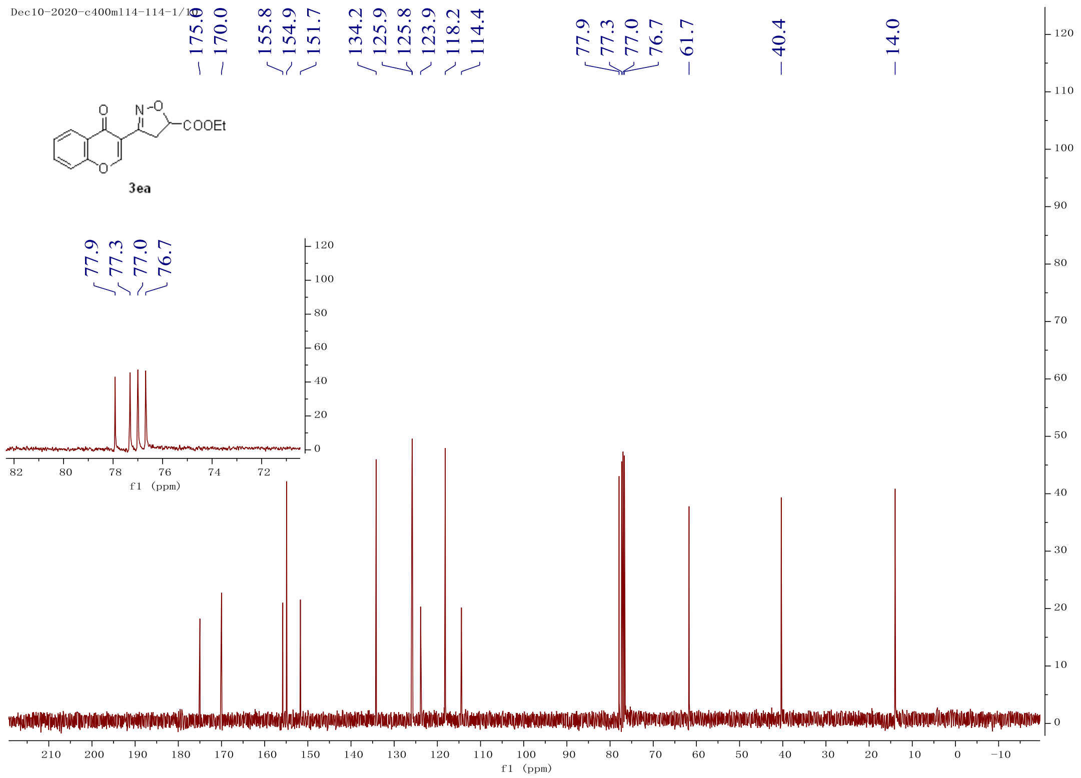
Dec10-2020-c400ml14-114-1/10
175.0
170.0
155.8
154.9
151.7
134.2
125.9
125.8
123.9
118.2
114.4
77.9
77.3
77.0
76.7
61.7
40.4
14.0
COOEt
3ea
f1 (ppm)

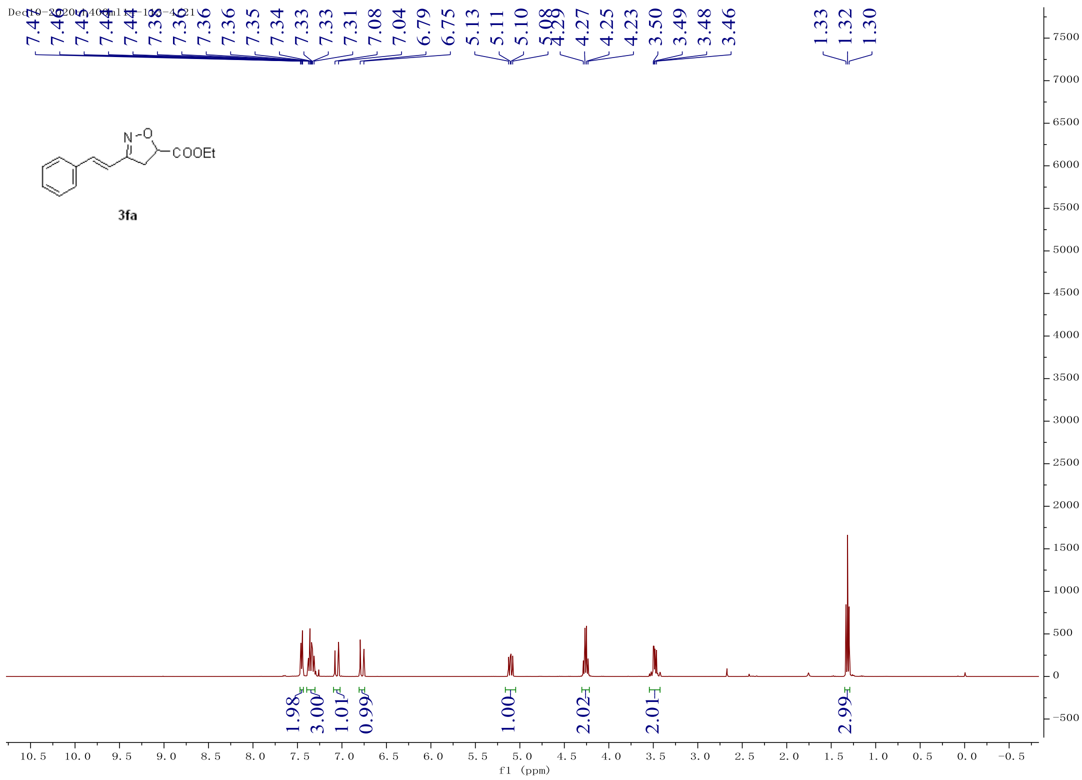
7.47
7.46
7.45
7.44
7.44
7.38
7.36
7.36
7.36
7.35
7.34
7.33
7.33
7.31
7.08
7.04
6.79
6.75
5.13
5.11
5.10
5.08
4.29
4.27
4.25
4.23
3.50
3.49
3.48
3.46
1.33
1.32
1.30
COOEt
3fa
1.98
3.00
1.01
0.99
1.00
2.02
2.01
2.99
f1 (ppm)

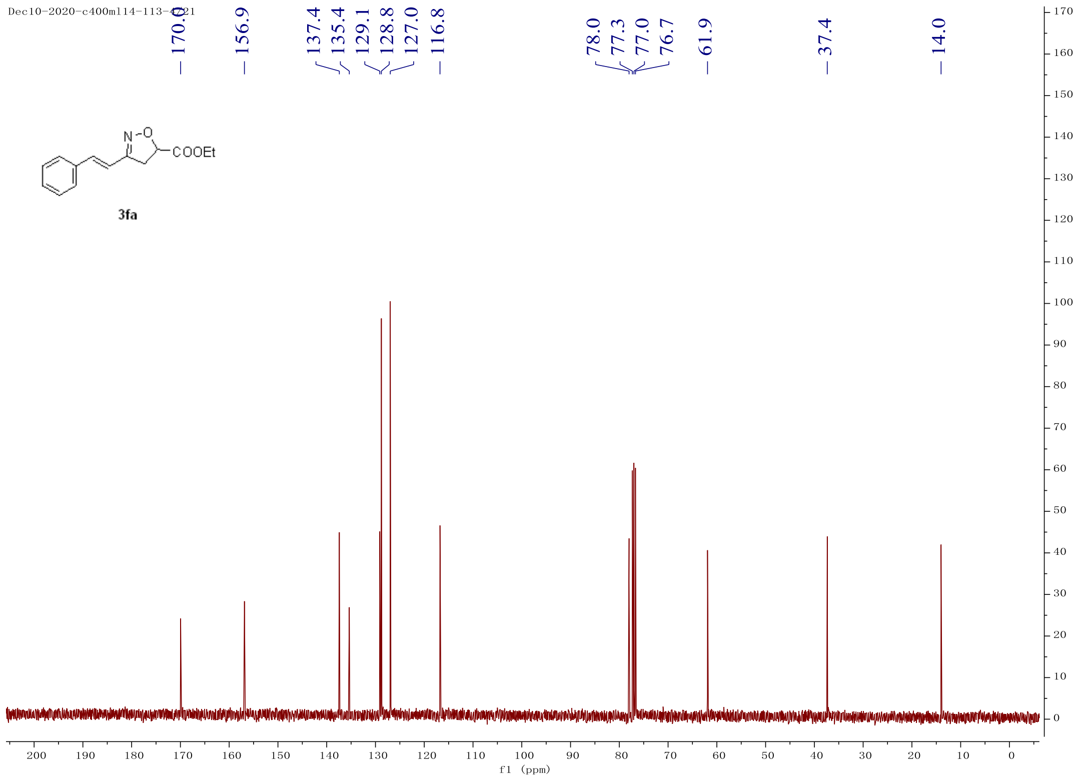
170.0
156.9
137.4
135.4
129.1
128.8
127.0
116.8
78.0
77.3
77.0
76.7
61.9
37.4
14.0
COOEt
3fa
f1 (ppm)

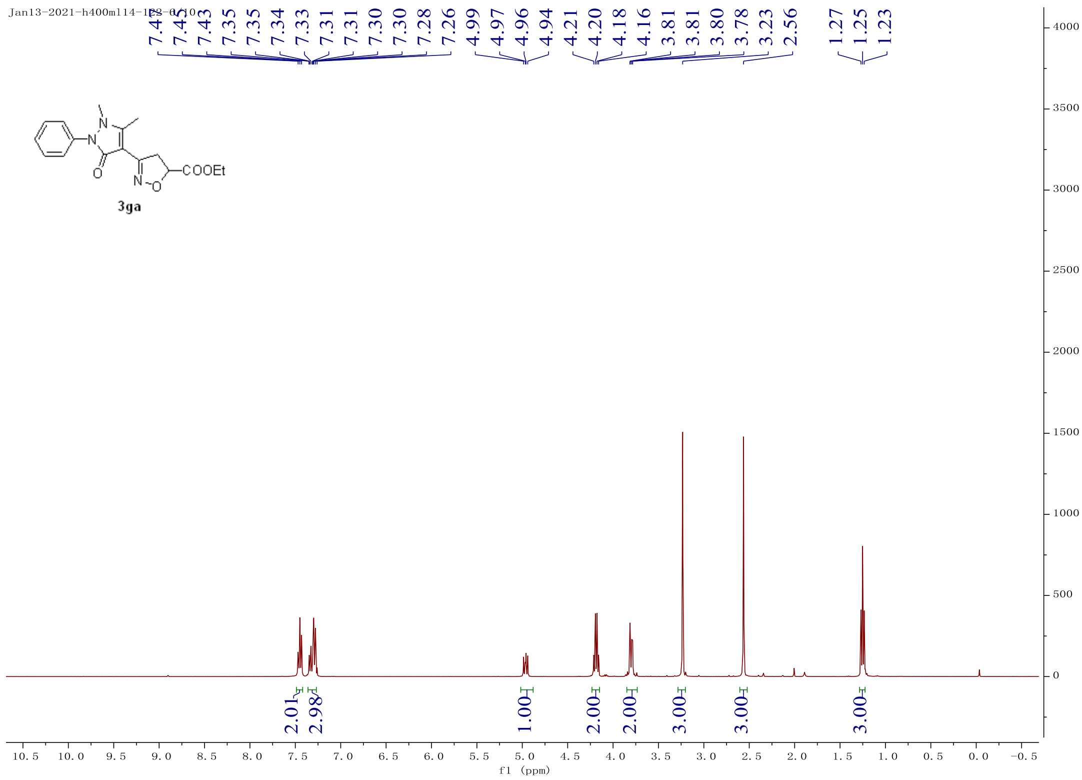
7.45
7.43
7.35
7.35
7.34
7.33
7.31
7.31
7.30
7.30
7.28
7.26
4.99
4.97
4.96
4.94
4.21
4.20
4.18
4.16
3.81
3.81
3.80
3.78
3.23
2.56
1.27
1.25
1.23
COOEt
3ga
2.01
2.98
1.00
2.00
2.00
3.00
3.00
3.00
f1 (ppm)

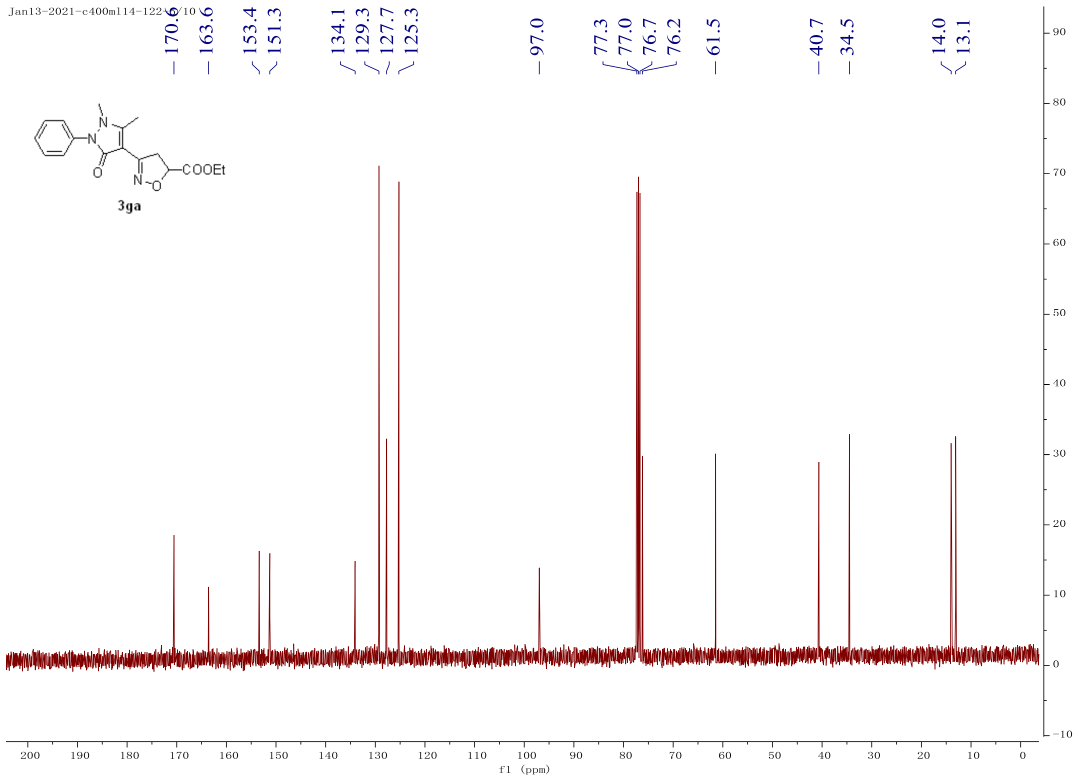
Jan13-2021-c400ml14-122-2/10
170.6
163.6
153.4
151.3
134.1
129.3
127.7
125.3
97.0
77.3
77.0
76.7
76.2
61.5
40.7
34.5
14.0
13.1
COOEt
3ga
f1 (ppm)

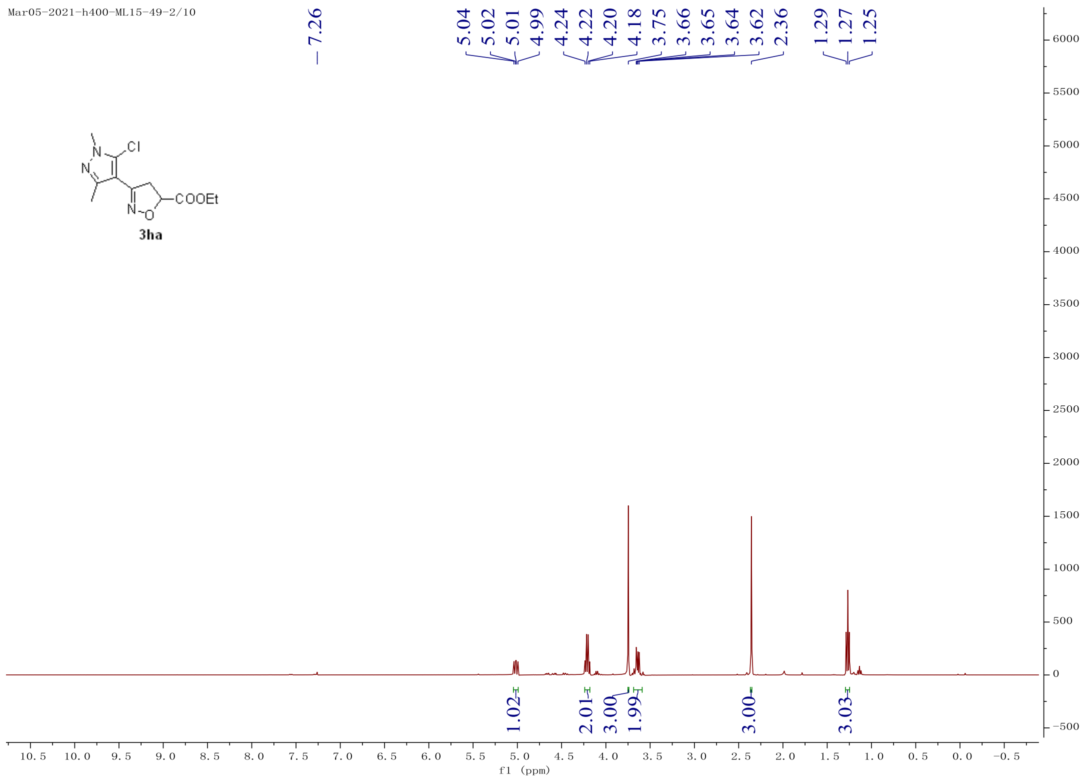
Mar05-2021-h400-ML15-49-2/10
7.26
5.04
5.02
5.01
4.99
4.24
4.22
4.20
4.18
3.75
3.66
3.65
3.64
3.62
2.36
1.29
1.27
1.25
Cl
COOEt
3ha
1.02
2.01
3.00
1.99
3.00
3.03
f1 (ppm)

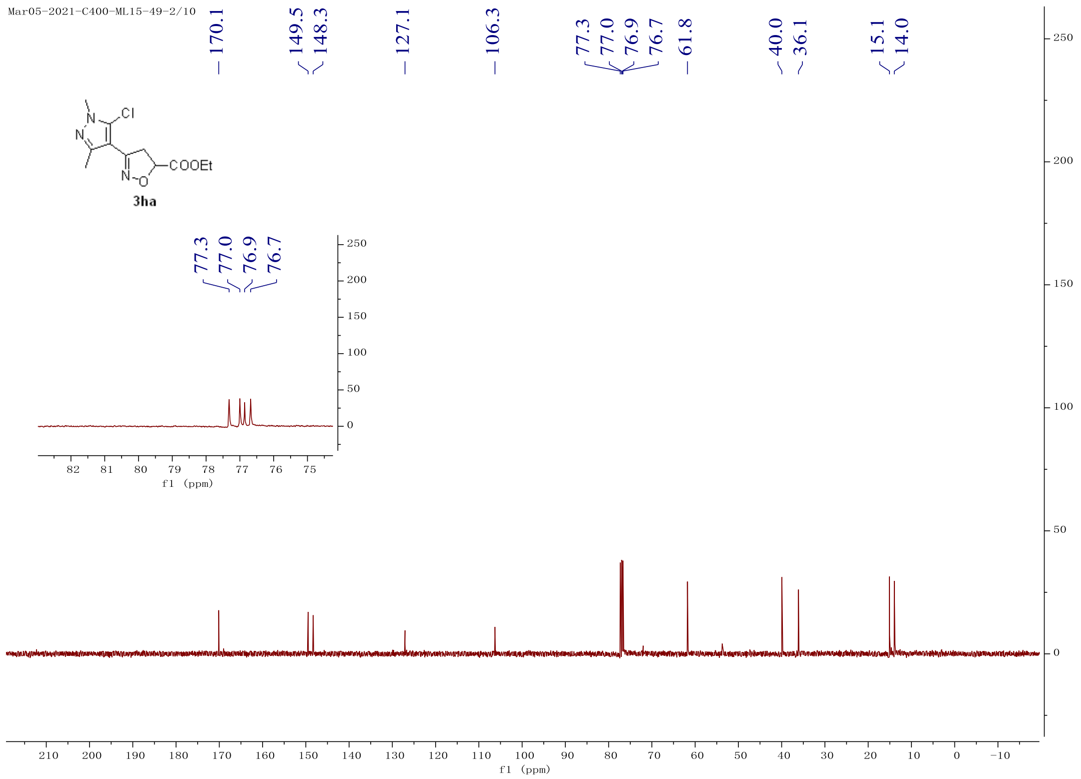

Mar05-2021-C400-ML15-49-2/10
170.1
149.5
148.3
127.1
106.3
77.3
77.0
76.9
76.7
61.8
40.0
36.1
15.1
14.0
Cl
N
N
COOEt
N-O
3ha
f1 (ppm)

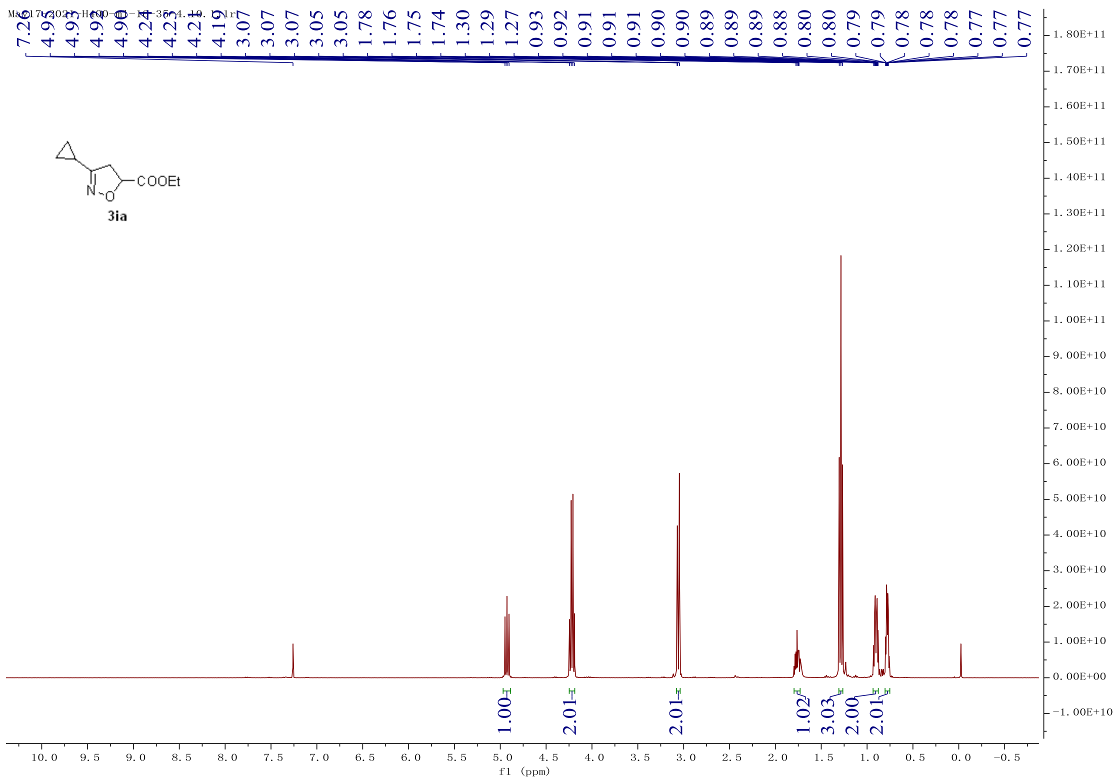

7.26
4.93
4.93
4.92
4.90
4.24
4.23
4.21
4.19
3.07
3.07
3.07
3.05
3.05
1.78
1.76
1.75
1.74
1.30
1.29
1.27
0.93
0.92
0.91
0.91
0.91
0.90
0.90
0.89
0.89
0.89
0.88
0.80
0.80
0.79
0.79
0.78
0.78
0.78
0.77
0.77
0.77
COOEt
N-O
3ia
1.00
2.01
2.01
1.02
3.03
2.00
2.01
f1 (ppm)

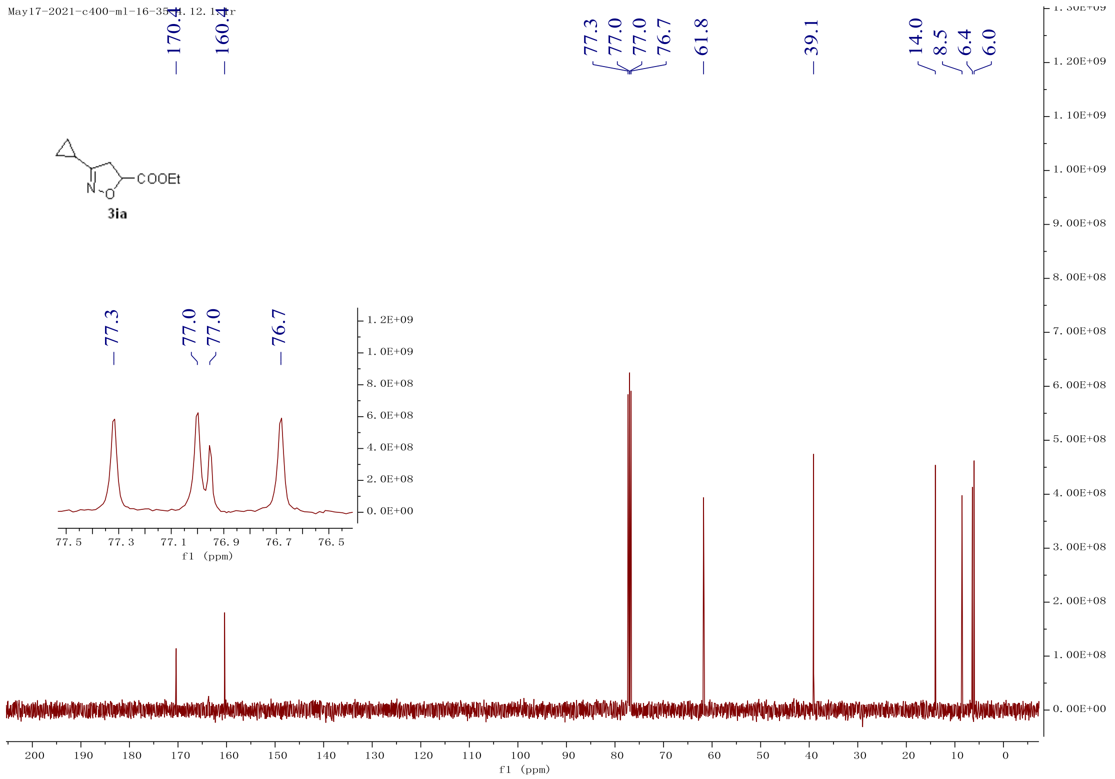
May17-2021-c400-ml-16-35-1.12.1.fr
170.4
160.4
77.3
77.0
77.0
76.7
61.8
39.1
14.0
8.5
6.4
6.0
COOEt
N
O
3ia
f1 (ppm)

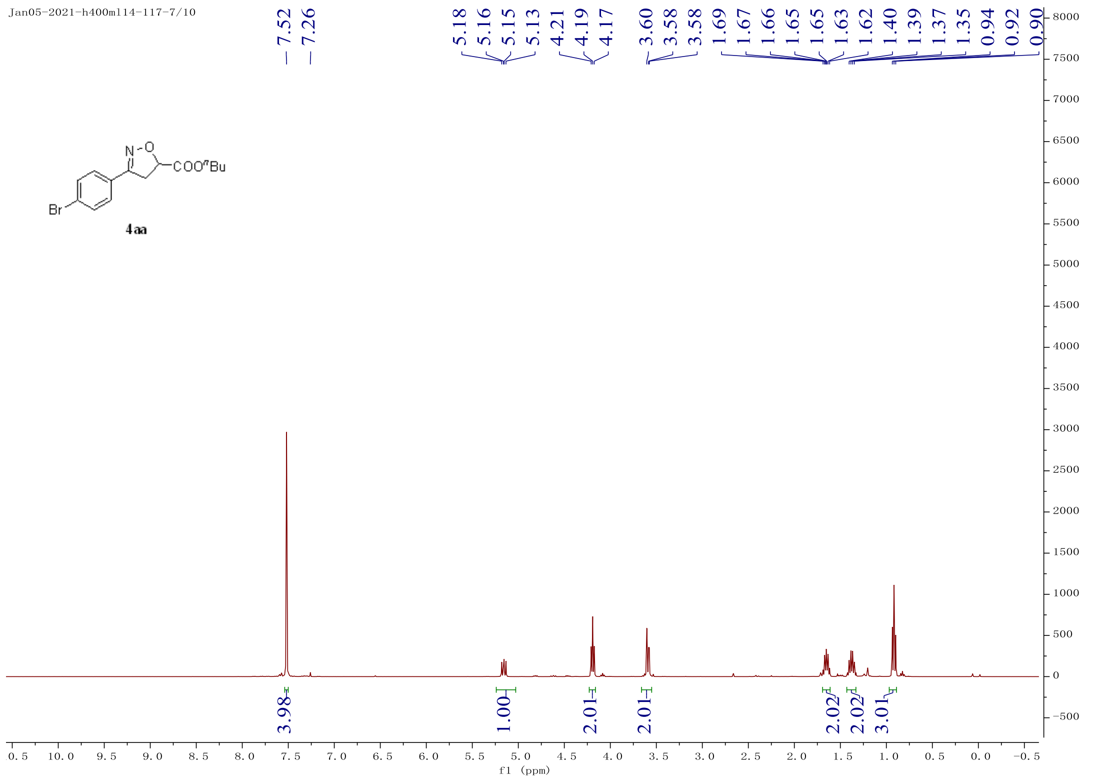
Jan05-2021-h400ml14-117-7/10
7.52
7.26
5.18
5.16
5.15
5.13
4.21
4.19
4.17
3.60
3.58
3.58
1.69
1.67
1.66
1.65
1.65
1.63
1.62
1.40
1.39
1.37
1.35
0.94
0.92
0.90
N-O
COO^{n}Bu
Br
4aa
3.98
1.00
2.01
2.01
2.02
2.02
3.01
f1 (ppm)

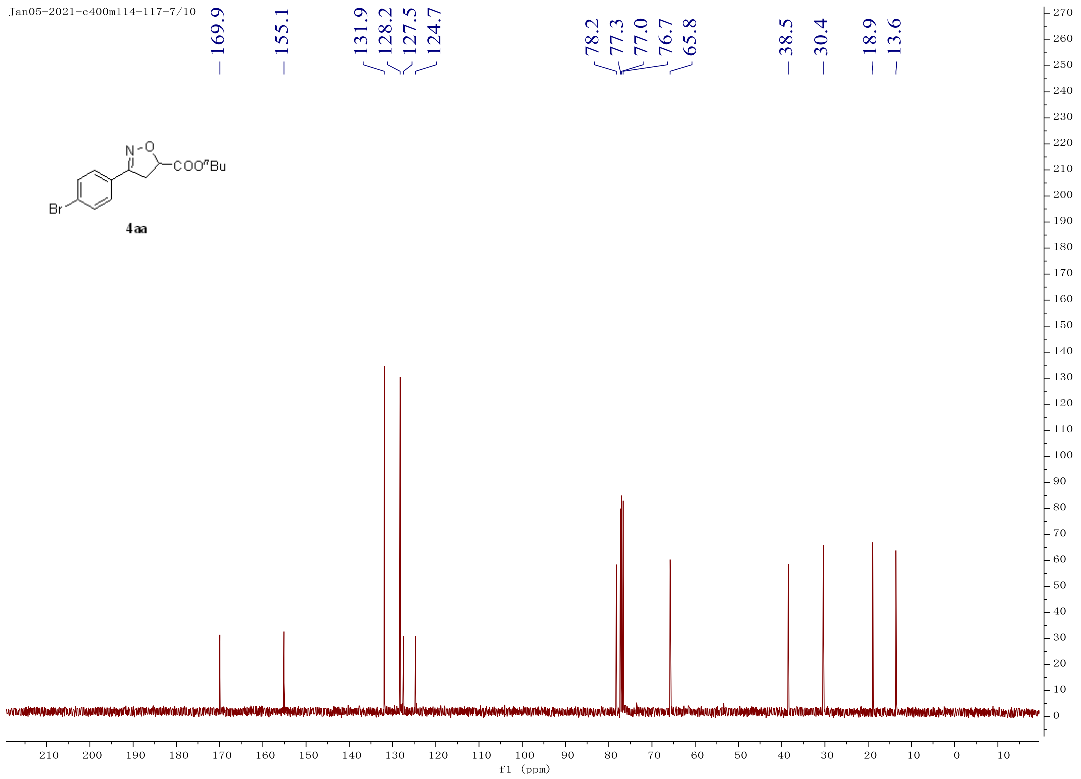
Jan05-2021-c400ml14-117-7/10
169.9
155.1
131.9
128.2
127.5
124.7
78.2
77.3
77.0
76.7
65.8
38.5
30.4
18.9
13.6
N-O
COO^{n}Bu
Br
4aa
f1 (ppm)

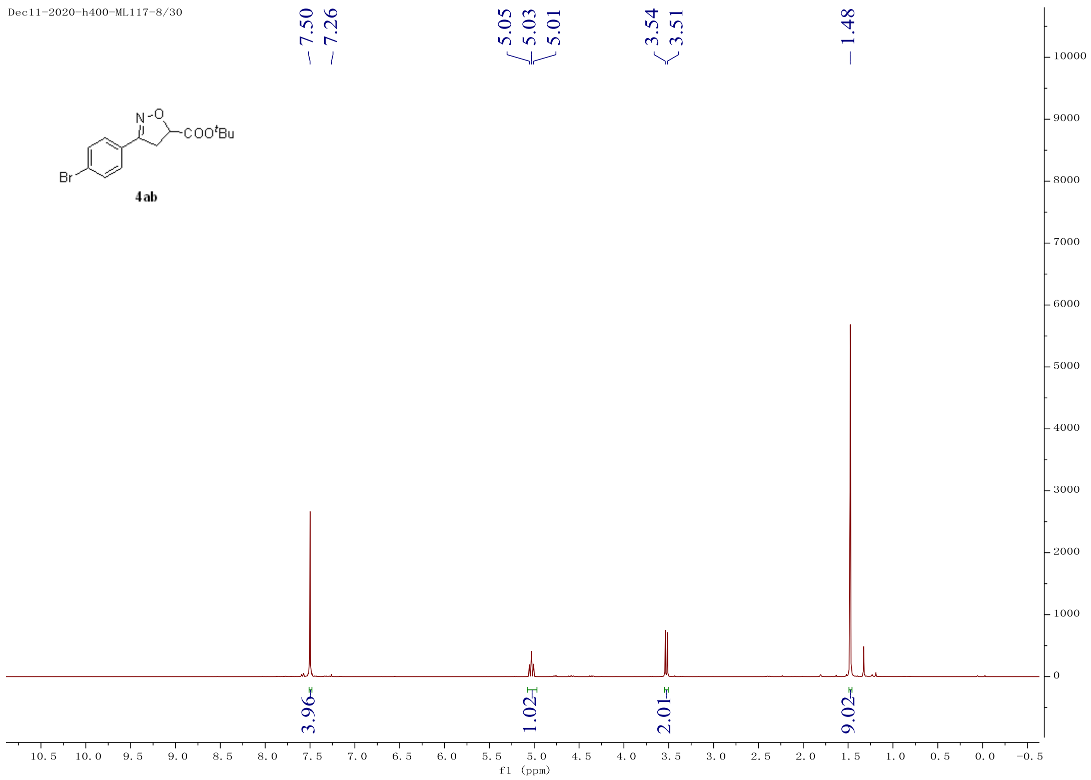
Dec11-2020-h400-ML117-8/30
7.50
7.26
5.05
5.03
5.01
3.54
3.51
1.48
N-O
COO^{t}Bu
Br
4ab
3.96
1.02
2.01
9.02
f1 (ppm)

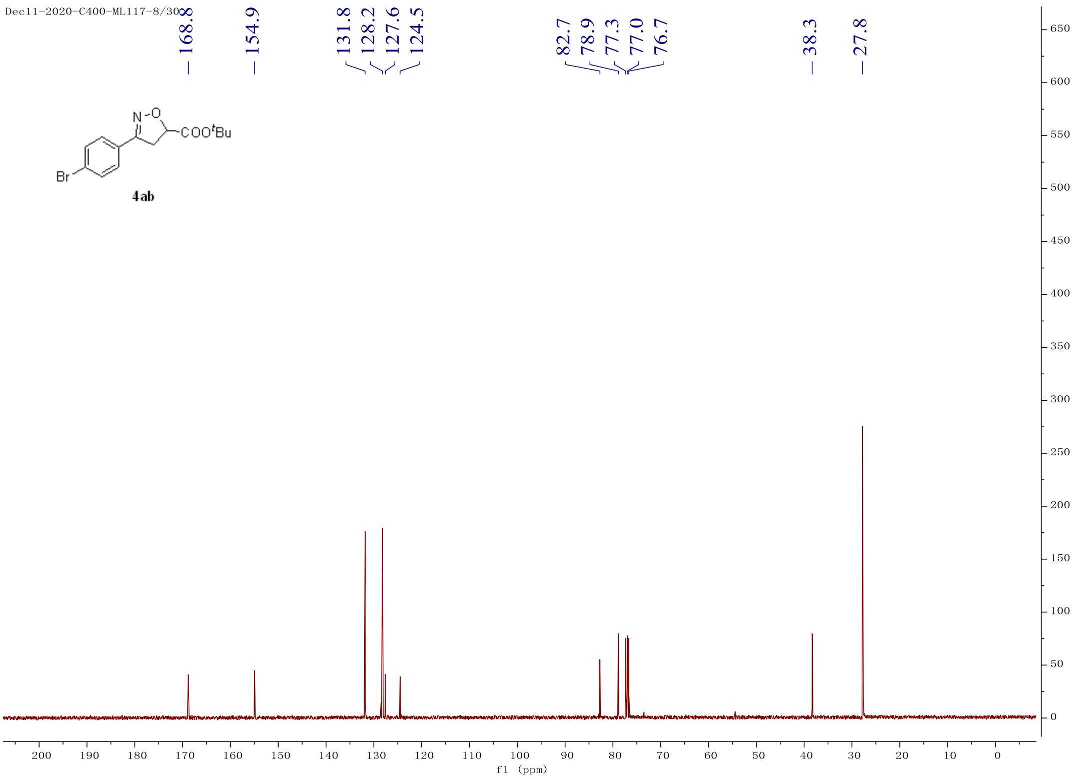

Dec11-2020-C400-ML117-8/30
168.8
154.9
131.8
128.2
127.6
124.5
82.7
78.9
77.3
77.0
76.7
38.3
27.8
4ab
f1 (ppm)

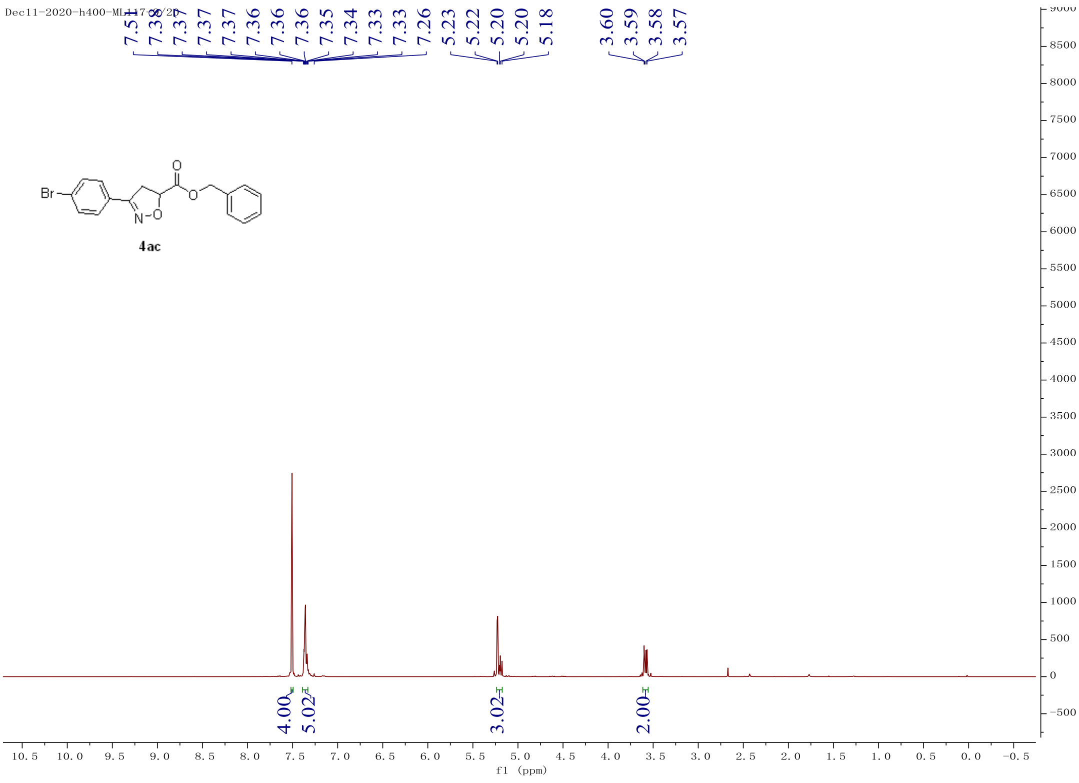

Dec11-2020-h400-ML117-8/27
7.51
7.38
7.37
7.37
7.37
7.36
7.36
7.35
7.34
7.33
7.33
7.26
5.23
5.22
5.20
5.20
5.18
3.60
3.59
3.58
3.57
4ac
4.00
5.02
3.02
2.00
f1 (ppm)

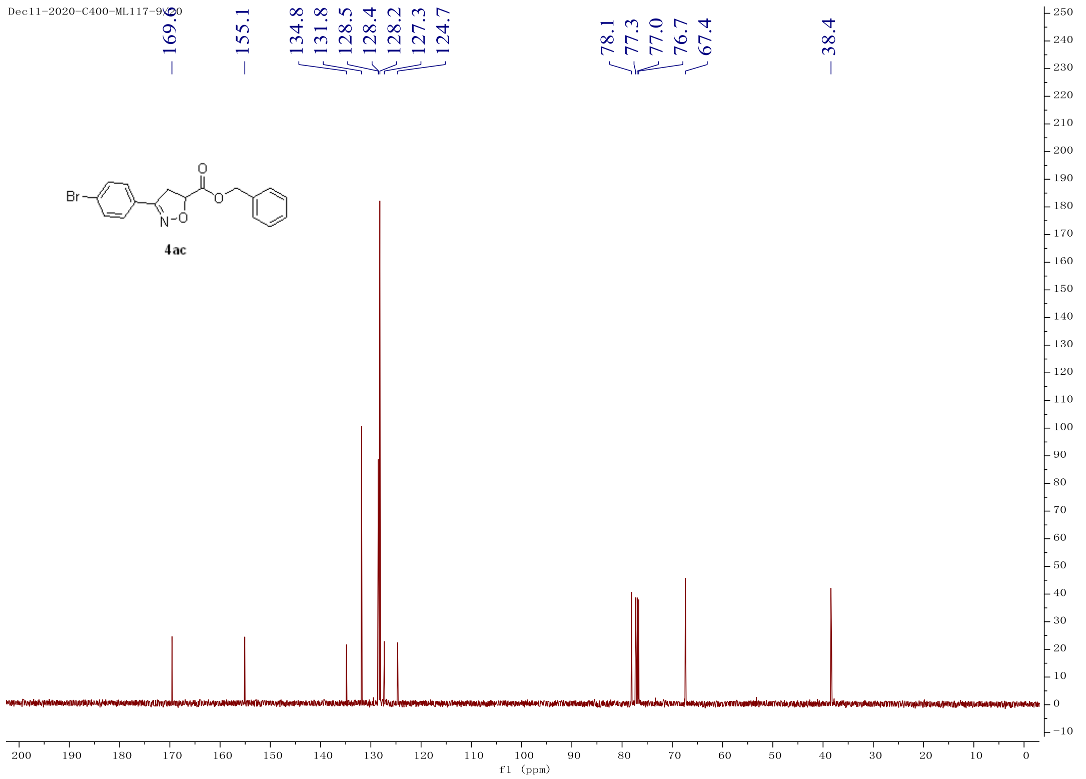

169.6
155.1
134.8
131.8
128.5
128.4
128.2
127.3
124.7
78.1
77.3
77.0
76.7
67.4
38.4
Br
O
O
N-O
4ac
f1 (ppm)

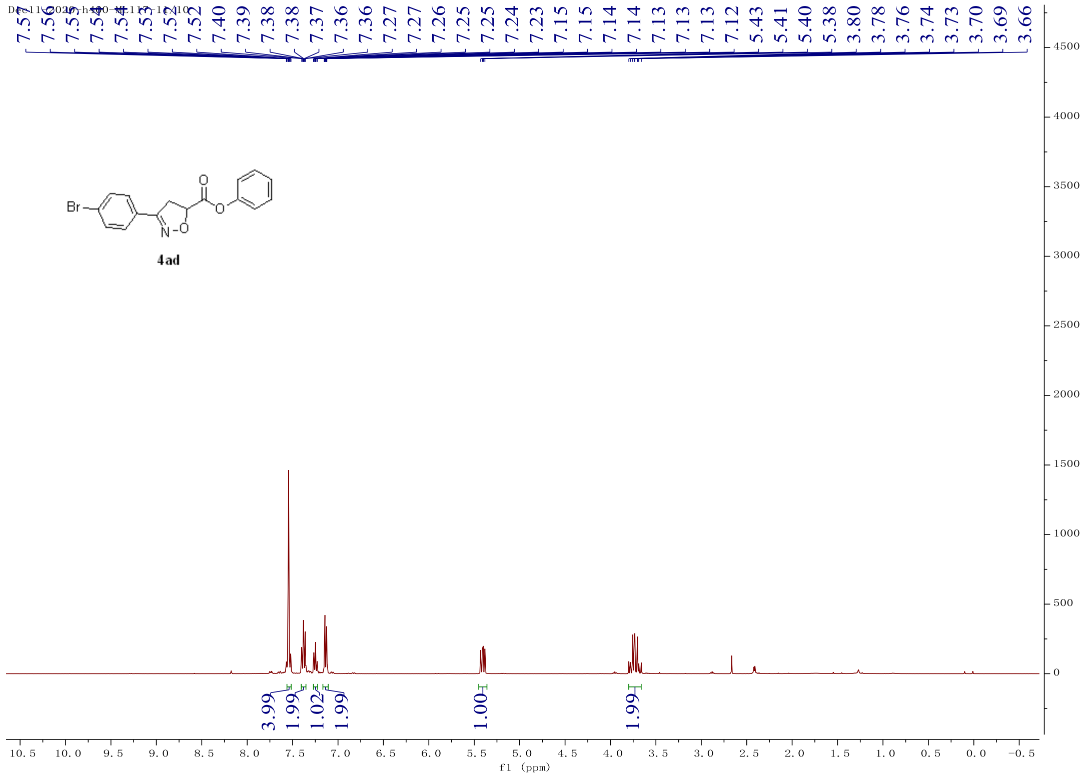

7.57
7.52
7.52
7.40
7.39
7.38
7.38
7.37
7.36
7.36
7.27
7.27
7.26
7.25
7.25
7.24
7.23
7.15
7.15
7.14
7.13
7.13
7.13
7.12
5.43
5.41
5.40
5.38
3.80
3.78
3.76
3.74
3.73
3.70
3.69
3.66
Br
O
O
N-O
4ad
3.99
1.99
1.02
1.99
1.00
1.99
f1 (ppm)

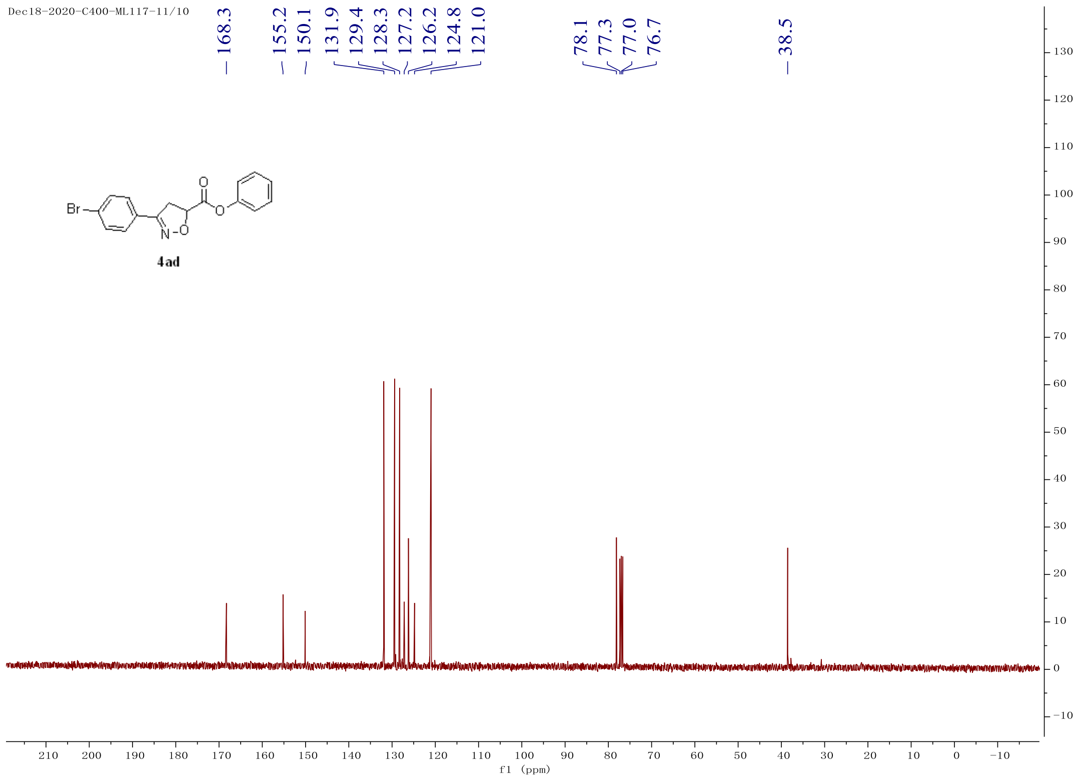
Dec18-2020-C400-ML117-11/10
168.3
155.2
150.1
131.9
129.4
128.3
127.2
126.2
124.8
121.0
78.1
77.3
77.0
76.7
38.5
Br
N-O
O
4ad
f1 (ppm)

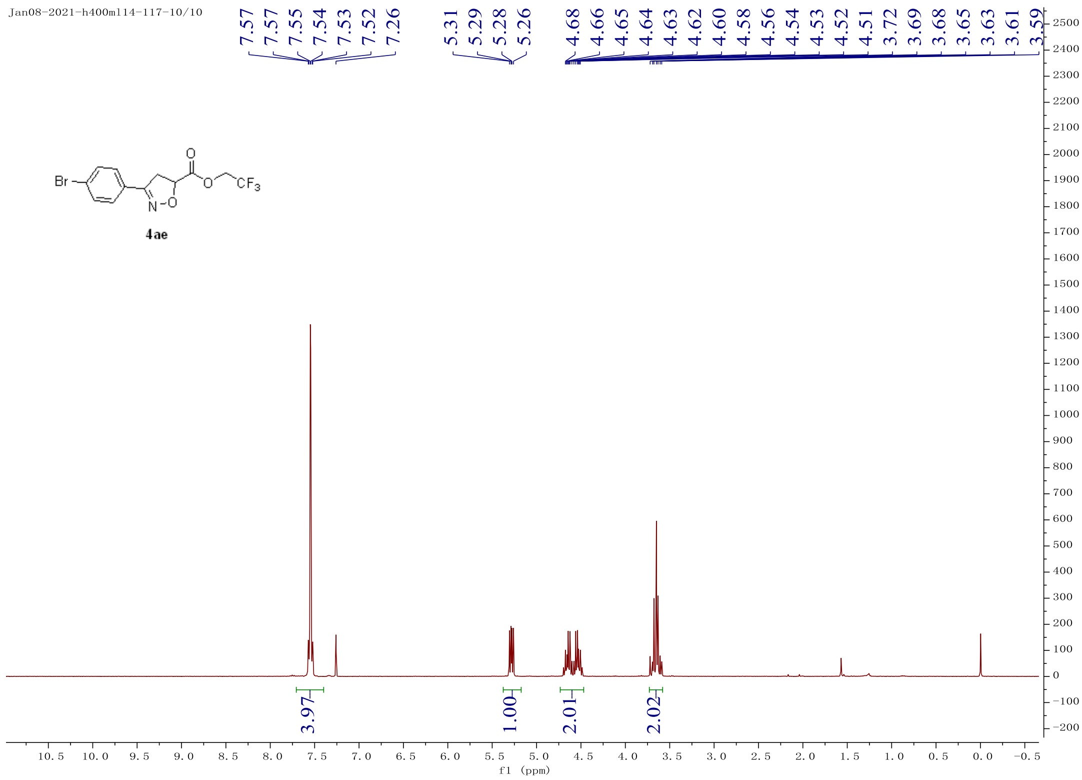
Jan08-2021-h400ml14-117-10/10
7.57
7.57
7.55
7.54
7.53
7.52
7.26
5.31
5.29
5.28
5.26
4.68
4.66
4.65
4.64
4.63
4.62
4.60
4.58
4.56
4.54
4.53
4.52
4.51
3.72
3.69
3.68
3.65
3.63
3.61
3.59
Br
N-O
O
CF_3
4ae
3.97
1.00
2.01
2.02
f1 (ppm)

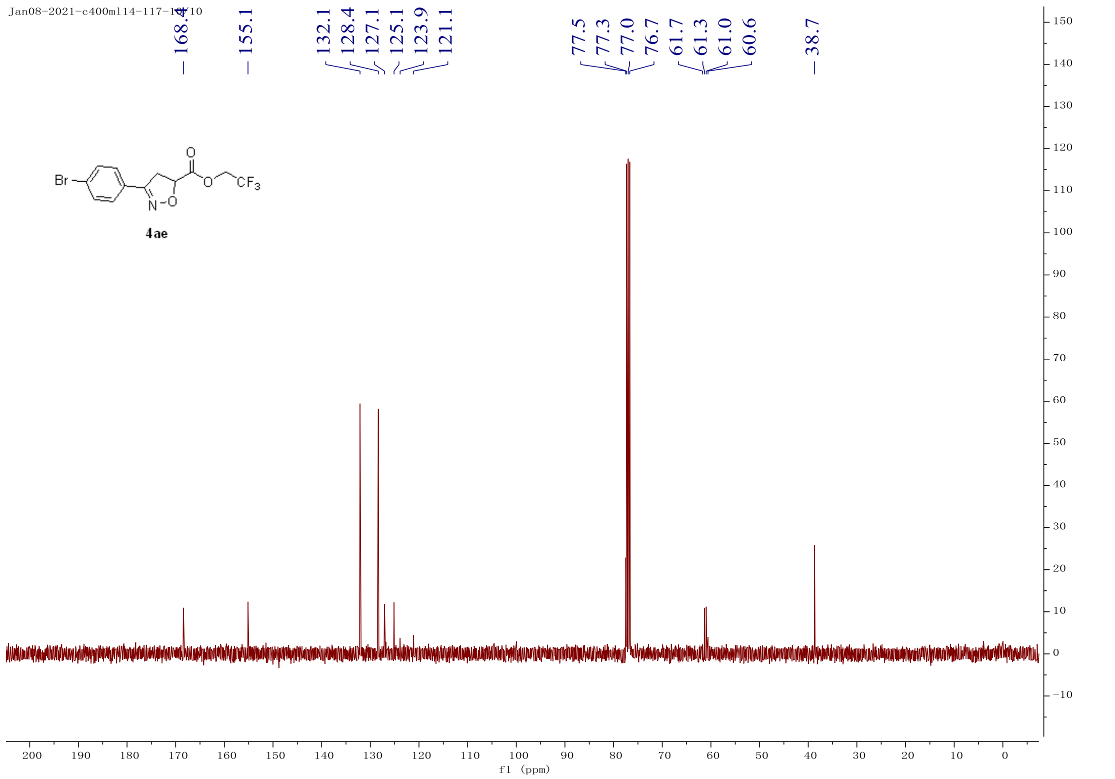
Jan08-2021-c400ml14-117-10.10
168.4
155.1
132.1
128.4
127.1
125.1
123.9
121.1
77.5
77.3
77.0
76.7
61.7
61.3
61.0
60.6
38.7
Br
O
O
N
O
CF3
4ae
f1 (ppm)

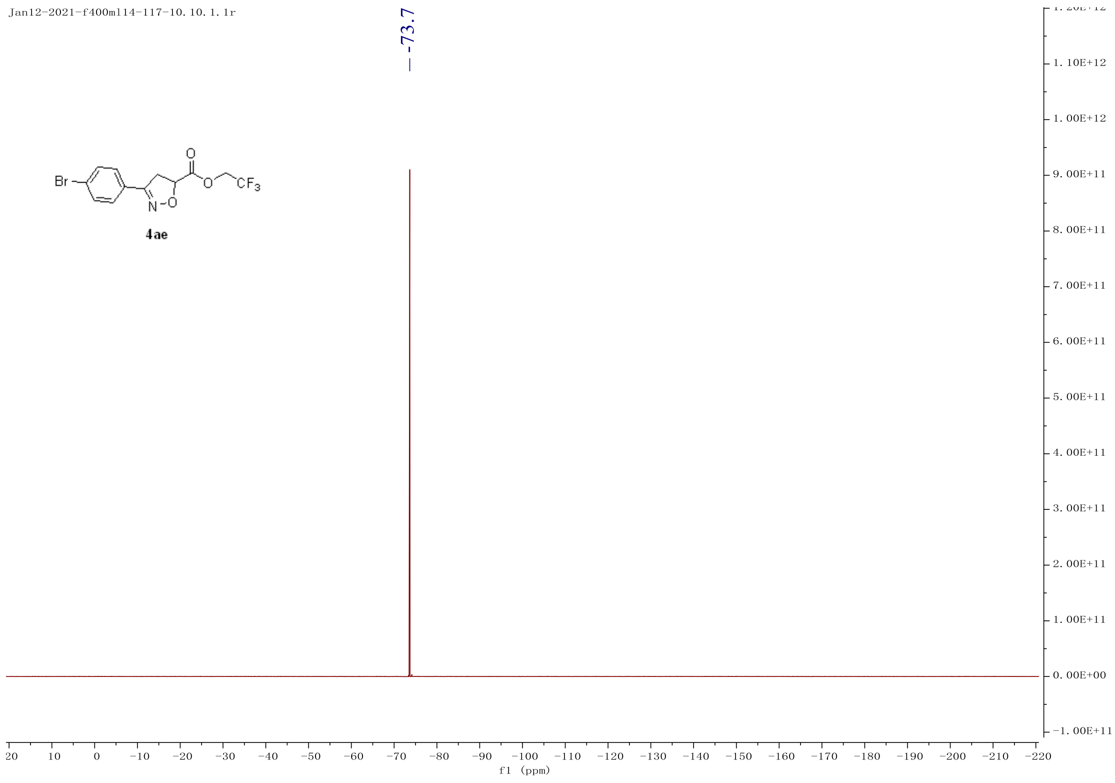
Jan12-2021-f400ml14-117-10.10.1.1r
-73.7
Br
O
O
N
O
CF3
4ae
f1 (ppm)

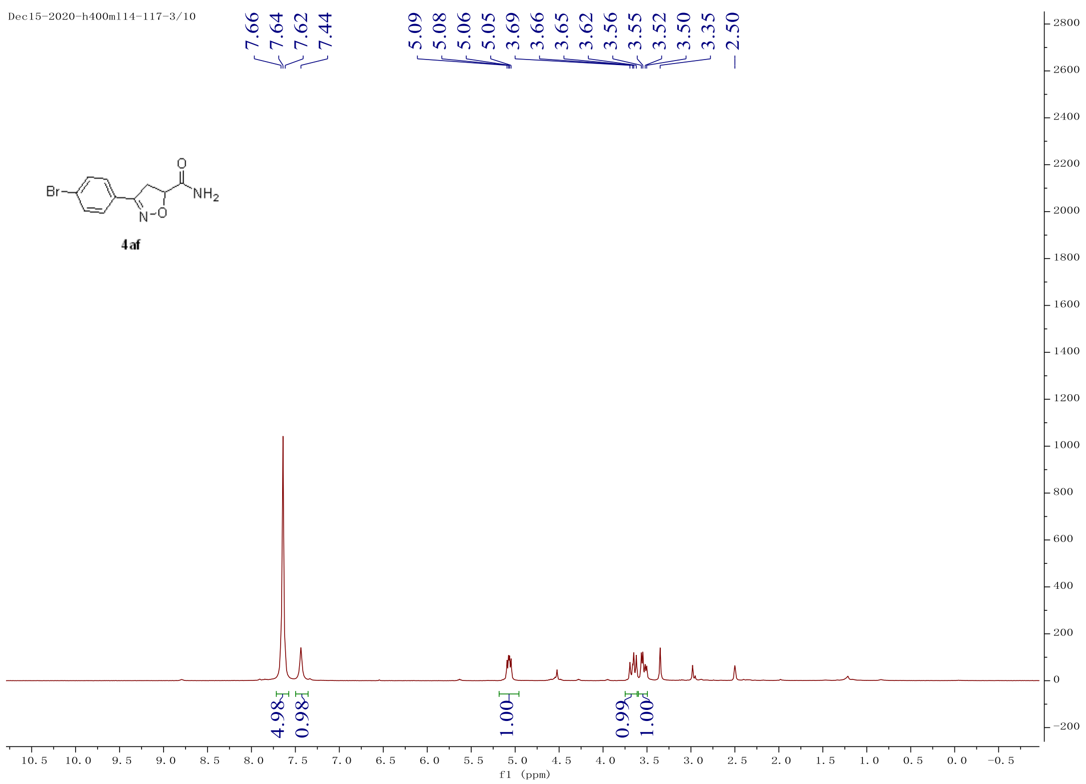
Dec15-2020-h400ml14-117-3/10
7.66
7.64
7.62
7.44
5.09
5.08
5.06
5.05
3.69
3.66
3.65
3.62
3.56
3.55
3.52
3.50
3.35
2.50
Br
O
NH2
N-O
4af
4.98
0.98
1.00
0.99
1.00
f1 (ppm)

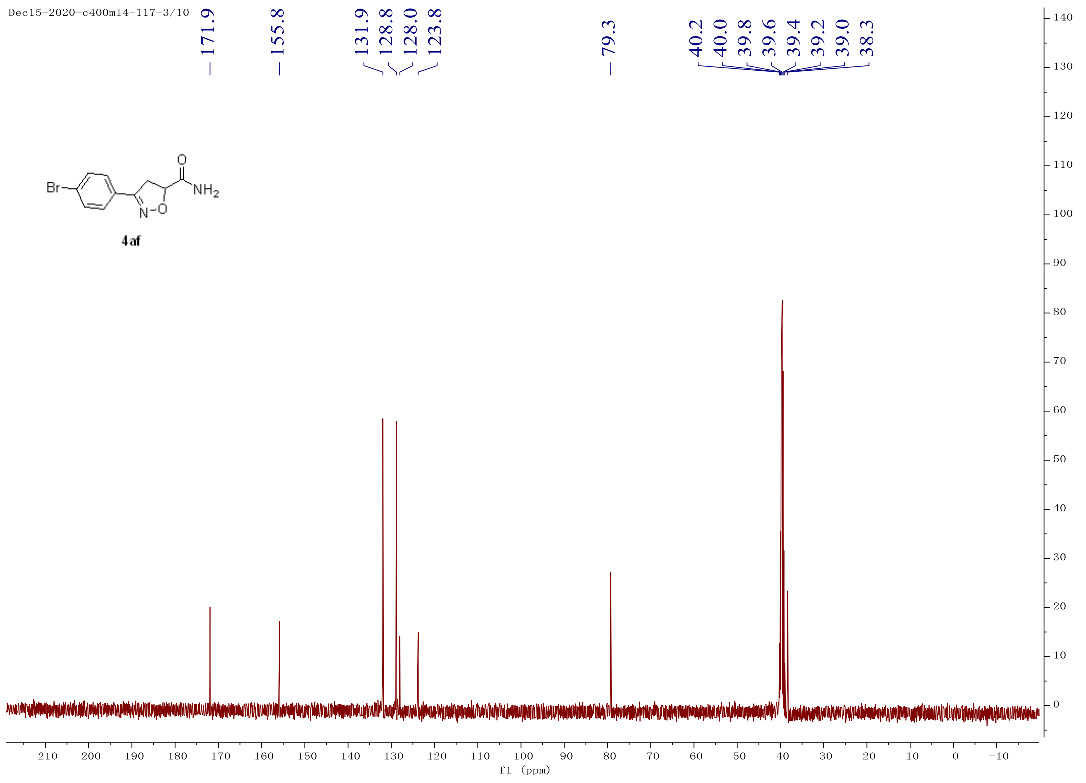
Dec15-2020-c400ml14-117-3/10
171.9
155.8
131.9
128.8
128.0
123.8
79.3
40.2
40.0
39.8
39.6
39.4
39.2
39.0
38.3
Br
O
NH2
N-O
4af
f1 (ppm)

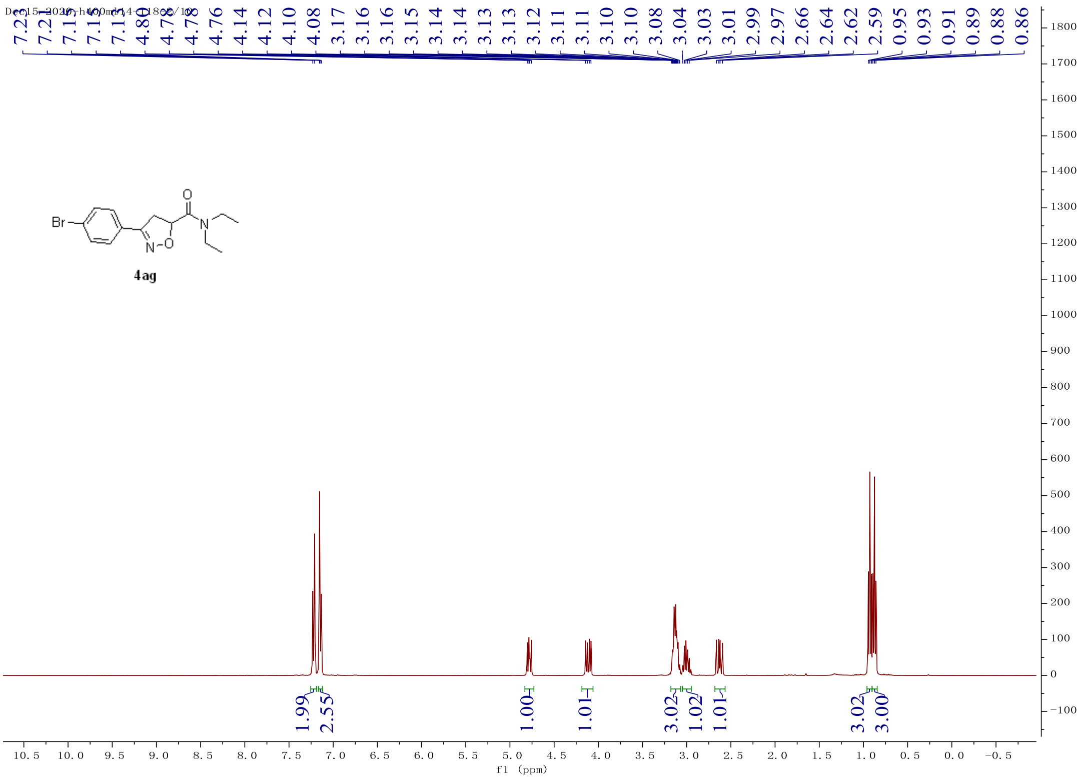
4.80
4.78
4.78
4.76
4.14
4.12
4.10
4.08
3.17
3.16
3.15
3.14
3.14
3.13
3.13
3.12
3.11
3.11
3.10
3.10
3.08
3.04
3.03
3.01
2.99
2.97
2.66
2.64
2.62
2.59
0.95
0.93
0.91
0.89
0.88
0.86
Br
O
N
N-O
4ag
1.99
2.55
1.00
1.01
3.02
1.02
1.01
3.02
3.00
f1 (ppm)

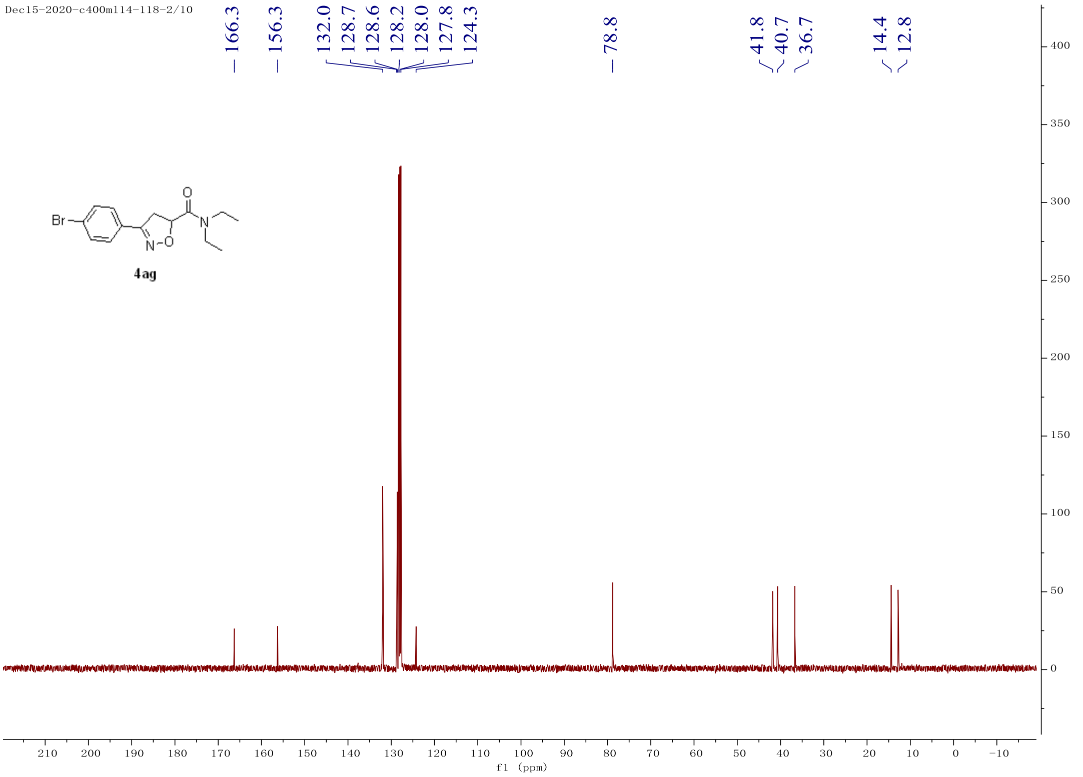
Dec15-2020-c400ml14-118-2/10
166.3
156.3
132.0
128.7
128.6
128.2
128.0
127.8
124.3
78.8
41.8
40.7
36.7
14.4
12.8
Br
O
N
N-O
4ag
f1 (ppm)

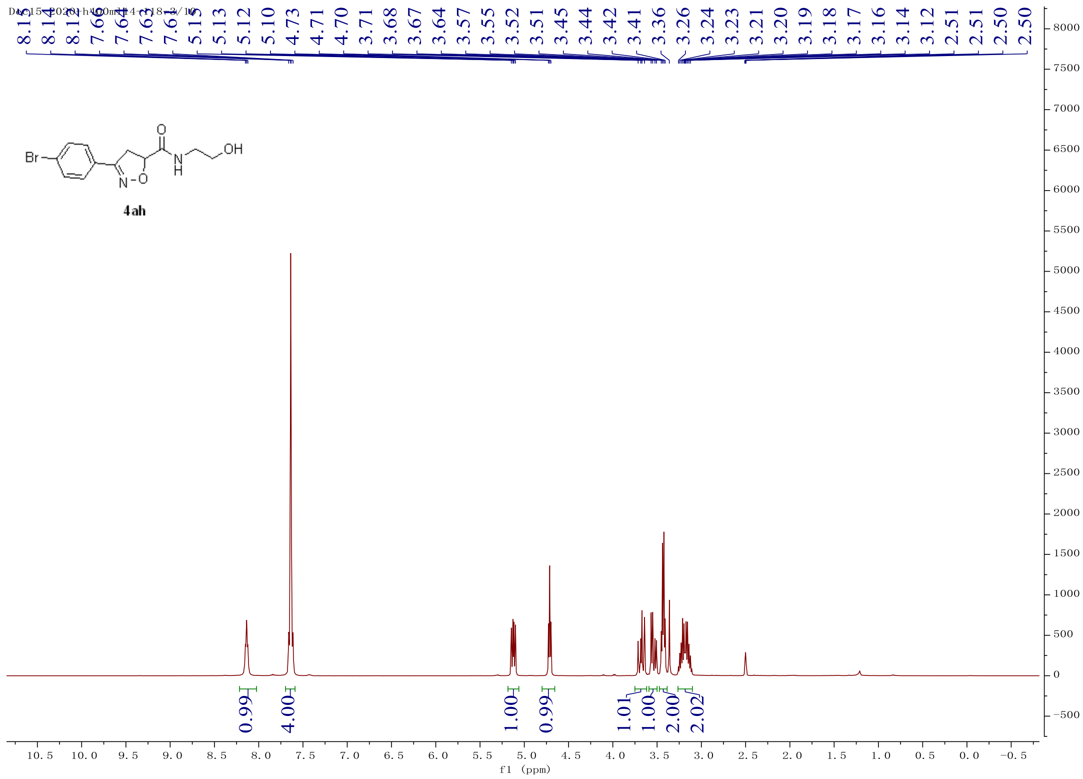
8.15
8.14
8.13
7.66
7.64
7.63
7.61
5.15
5.13
5.12
5.10
4.73
4.71
4.70
3.71
3.68
3.67
3.64
3.57
3.55
3.52
3.51
3.45
3.44
3.42
3.41
3.36
3.26
3.24
3.23
3.21
3.20
3.19
3.18
3.17
3.16
3.14
3.12
2.51
2.51
2.50
2.50
Br
O
N
H
OH
N
O
4ah
0.99
4.00
1.00
0.99
1.01
1.00
2.00
2.02
f1 (ppm)

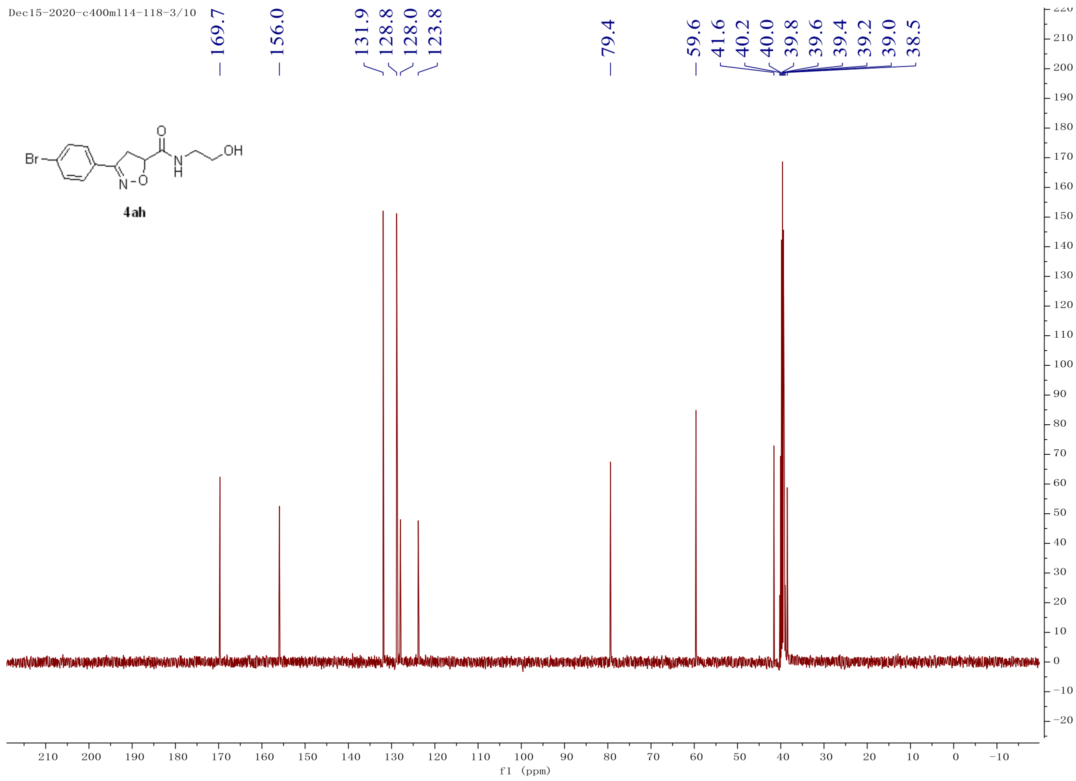
Dec15-2020-c400ml14-118-3/10
169.7
156.0
131.9
128.8
128.0
123.8
79.4
59.6
41.6
40.2
40.0
39.8
39.6
39.4
39.2
39.0
38.5
Br
O
N
H
OH
N
O
4ah
f1 (ppm)

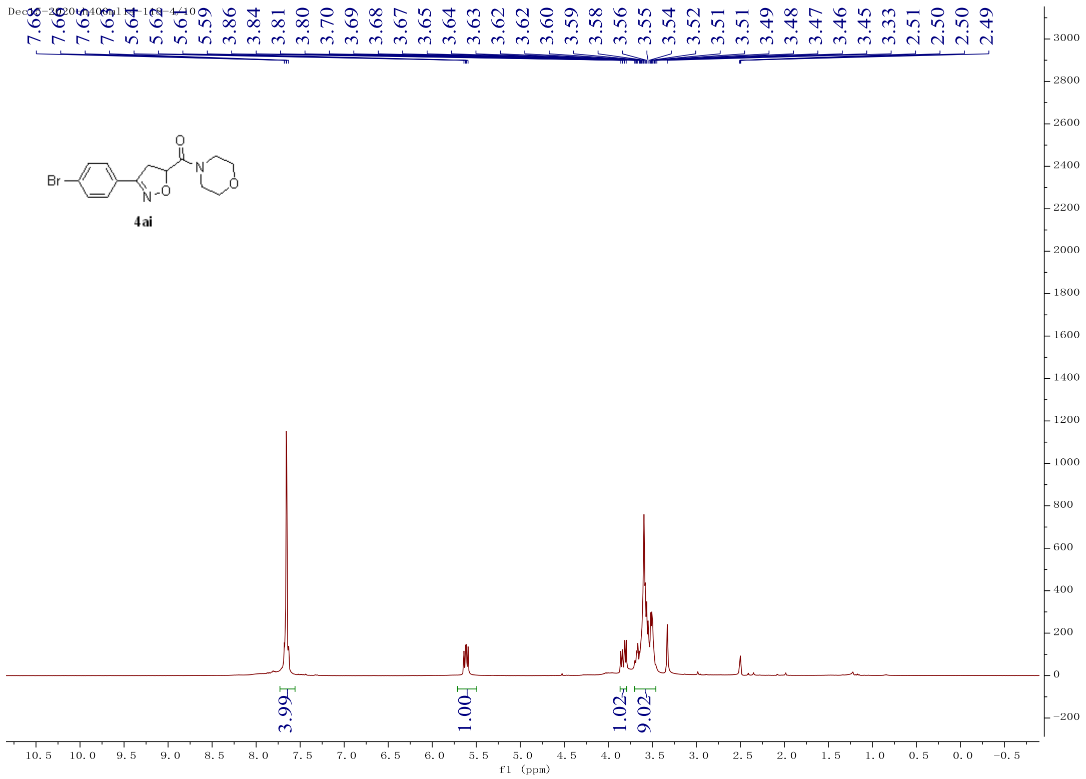

5.64
5.62
5.61
5.59
3.86
3.84
3.81
3.80
3.70
3.69
3.68
3.67
3.65
3.64
3.63
3.62
3.62
3.60
3.59
3.58
3.56
3.55
3.54
3.52
3.51
3.51
3.49
3.48
3.47
3.46
3.45
3.33
2.51
2.50
2.50
2.49
Br
N-O
O
N
4ai
3.99
1.00
1.02
9.02
f1 (ppm)

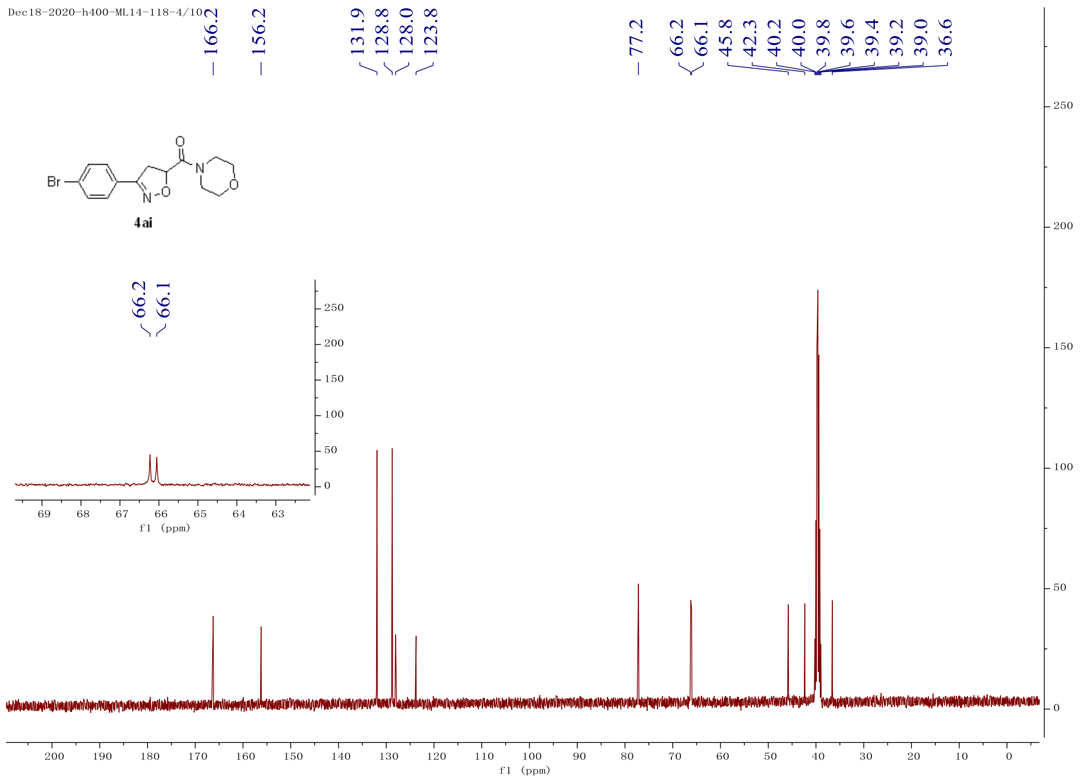

Dec18-2020-h400-ML14-118-4/10
166.2
156.2
131.9
128.8
128.0
123.8
77.2
66.2
66.1
45.8
42.3
40.2
40.0
39.8
39.6
39.4
39.2
39.0
36.6
Br
N-O
O
N
4ai
66.2
66.1
f1 (ppm)
f1 (ppm)

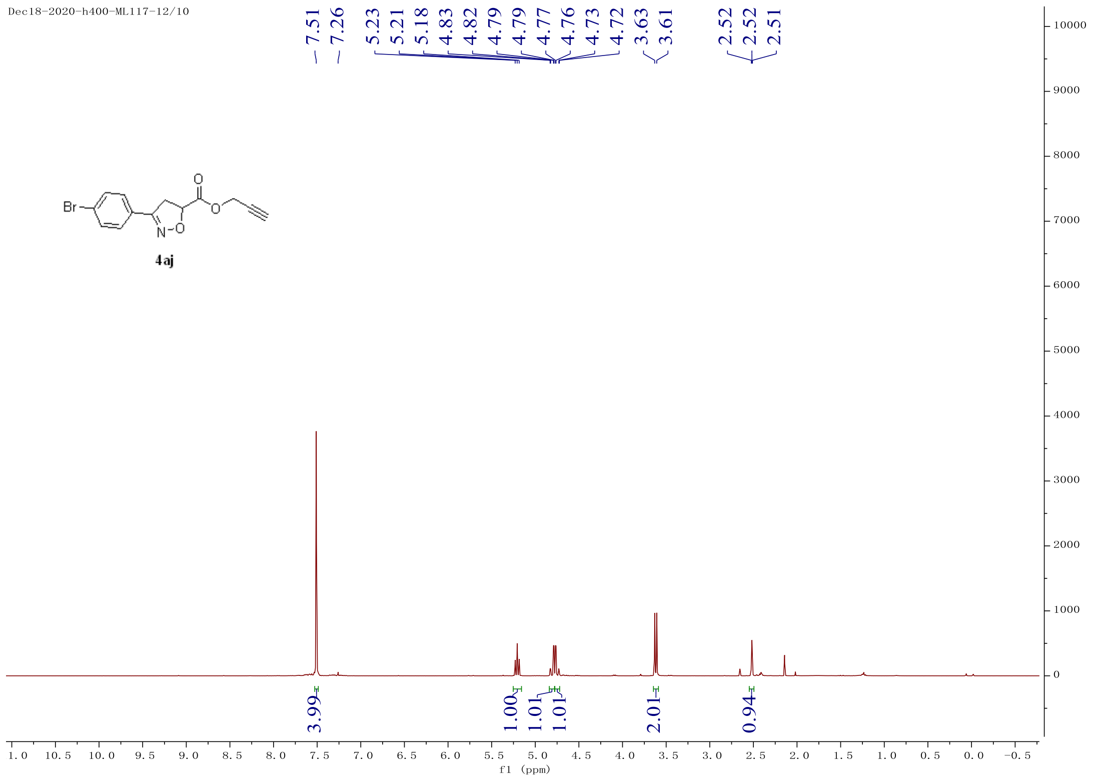
Dec18-2020-h400-ML117-12/10
7.51
7.26
5.23
5.21
5.18
4.83
4.82
4.79
4.79
4.77
4.76
4.73
4.72
3.63
3.61
2.52
2.52
2.51
Br
N
O
O
O
4aj
3.99
1.00
1.01
1.01
2.01
0.94
f1 (ppm)

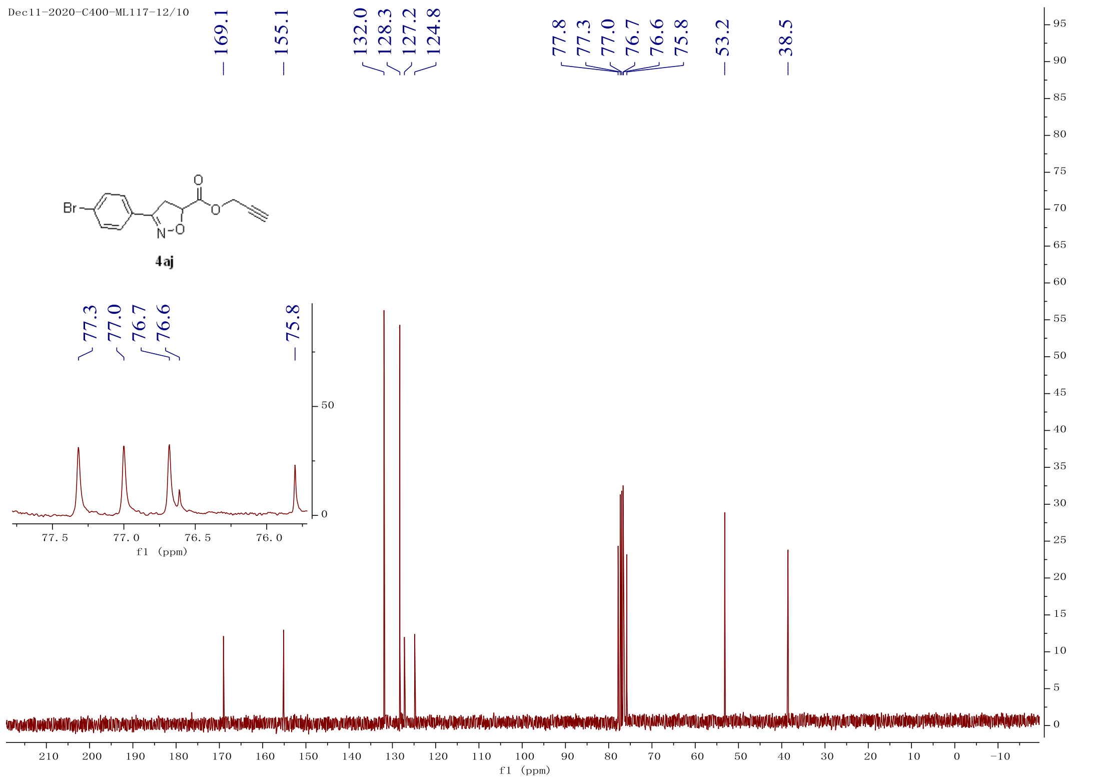
Dec11-2020-C400-ML117-12/10
169.1
155.1
132.0
128.3
127.2
124.8
77.8
77.3
77.0
76.7
76.6
75.8
53.2
38.5
Br
N
O
O
O
4aj
f1 (ppm)

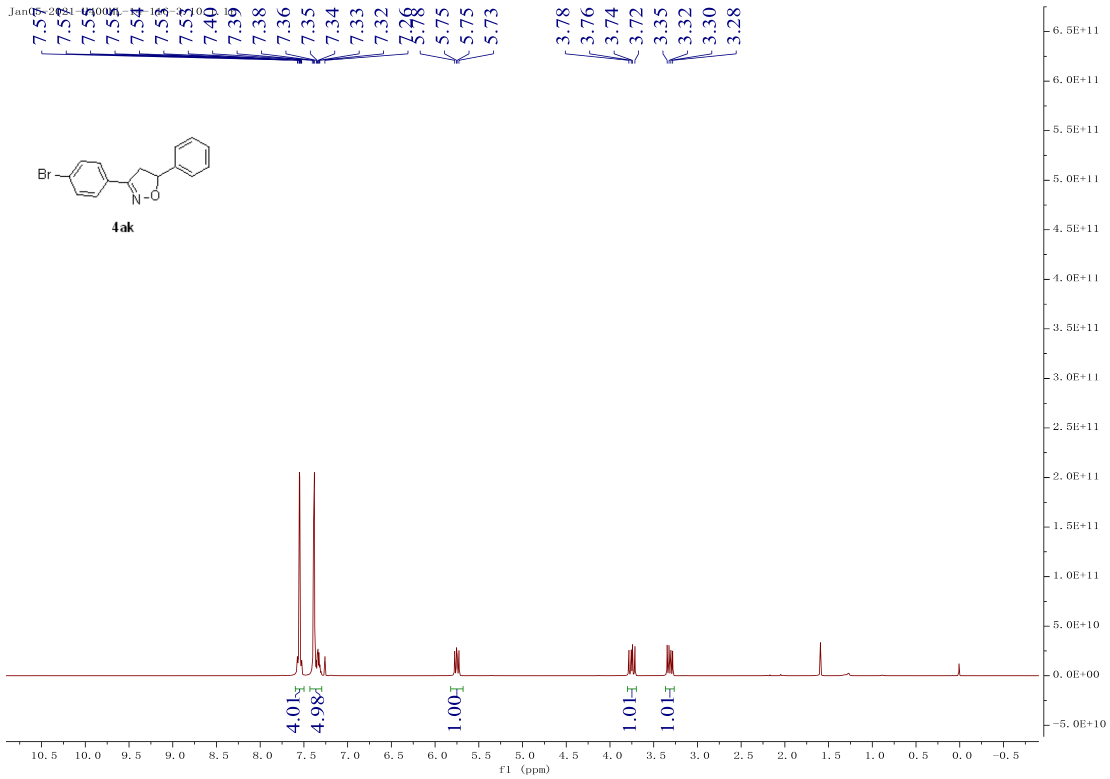

Br
N
O
4ak
7.57
7.40
7.39
7.38
7.36
7.35
7.34
7.33
7.32
7.26
5.78
5.75
5.75
5.73
3.78
3.76
3.74
3.72
3.35
3.32
3.30
3.28
4.01
4.98
1.00
1.01
1.01
f1 (ppm)

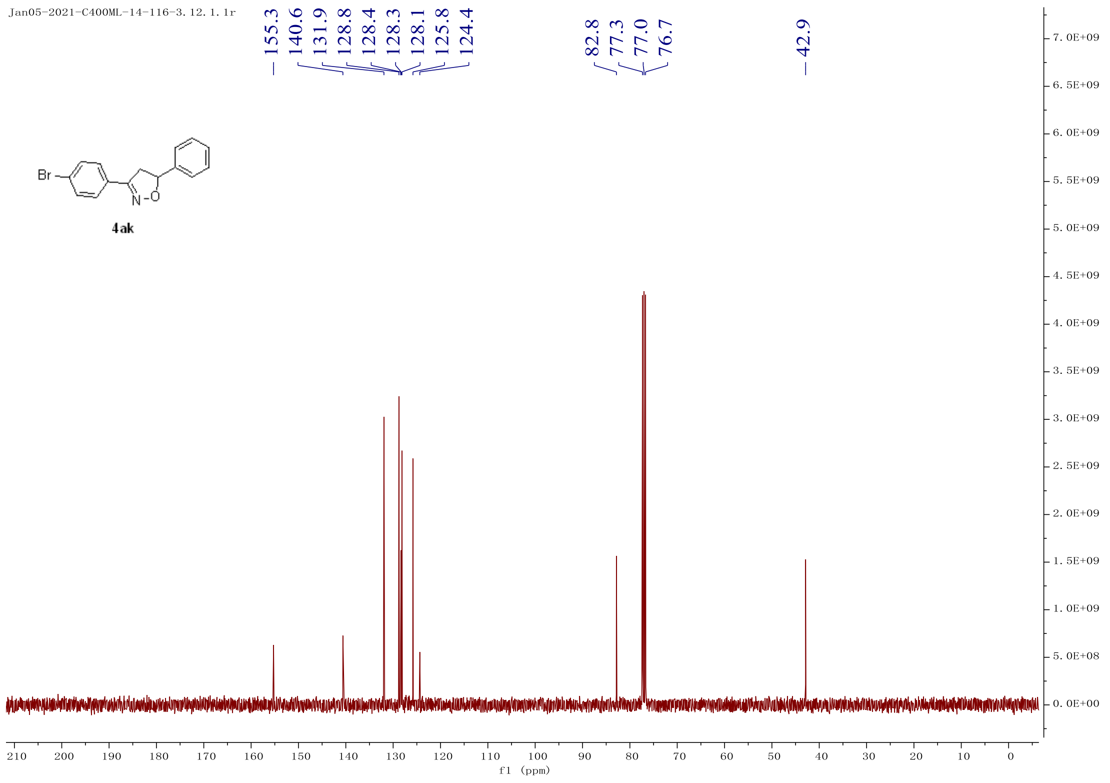

Jan05-2021-C400ML-14-116-3.12.1.1r
Br
N
O
4ak
155.3
140.6
131.9
128.8
128.4
128.3
128.1
125.8
124.4
82.8
77.3
77.0
76.7
42.9
f1 (ppm)

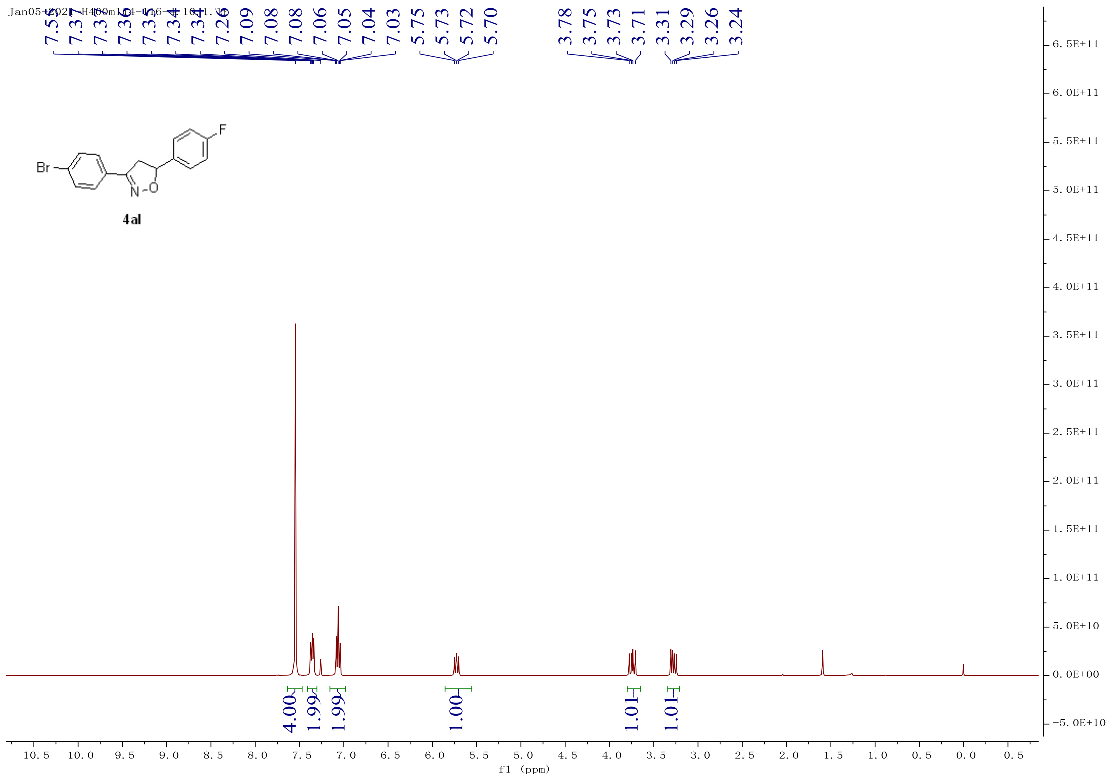

7.55 7.37 7.36 7.36 7.35 7.34 7.34 7.26 7.09 7.08 7.08 7.06 7.05 7.04 7.03 5.75 5.73 5.72 5.70 3.78 3.75 3.73 3.71 3.31 3.29 3.26 3.24
Br
N-O
F
4al
4.00 1.99 1.99 1.00 1.01 1.01
f1 (ppm)

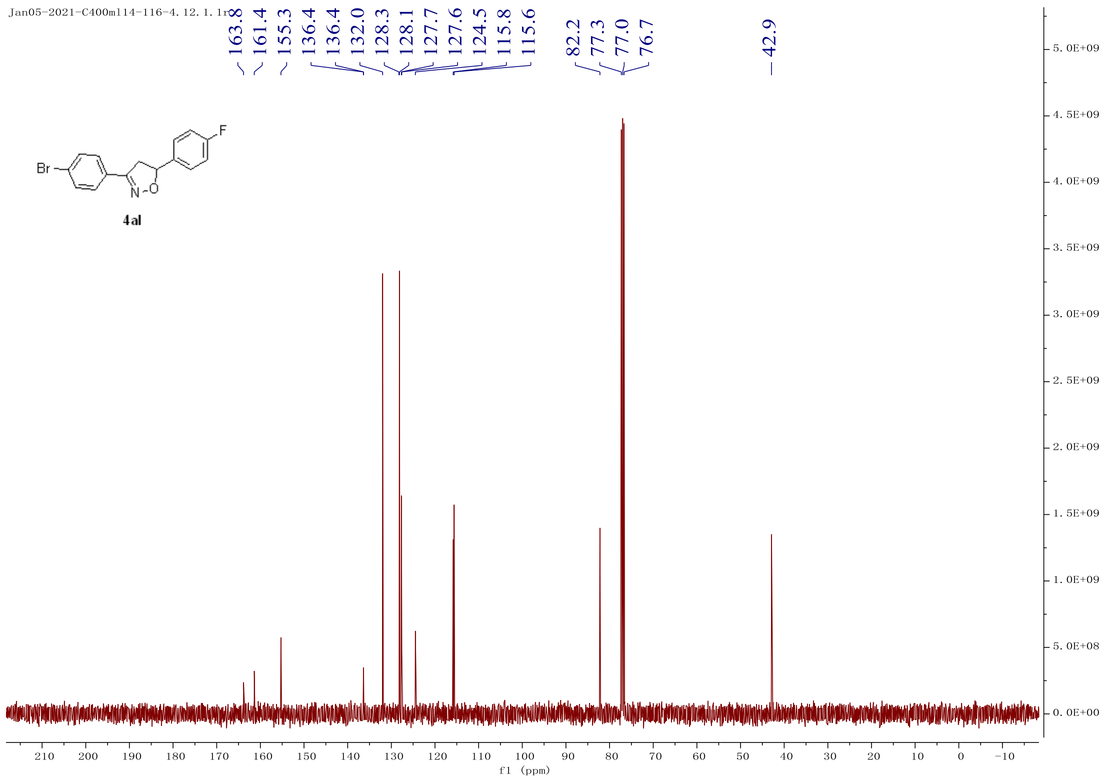

Jan05-2021-C400ml14-116-4.12.1.1r
163.8 161.4 155.3 136.4 136.4 132.0 128.3 128.1 127.7 127.6 124.5 115.8 115.6 82.2 77.3 77.0 76.7 42.9
Br
N-O
F
4al
f1 (ppm)

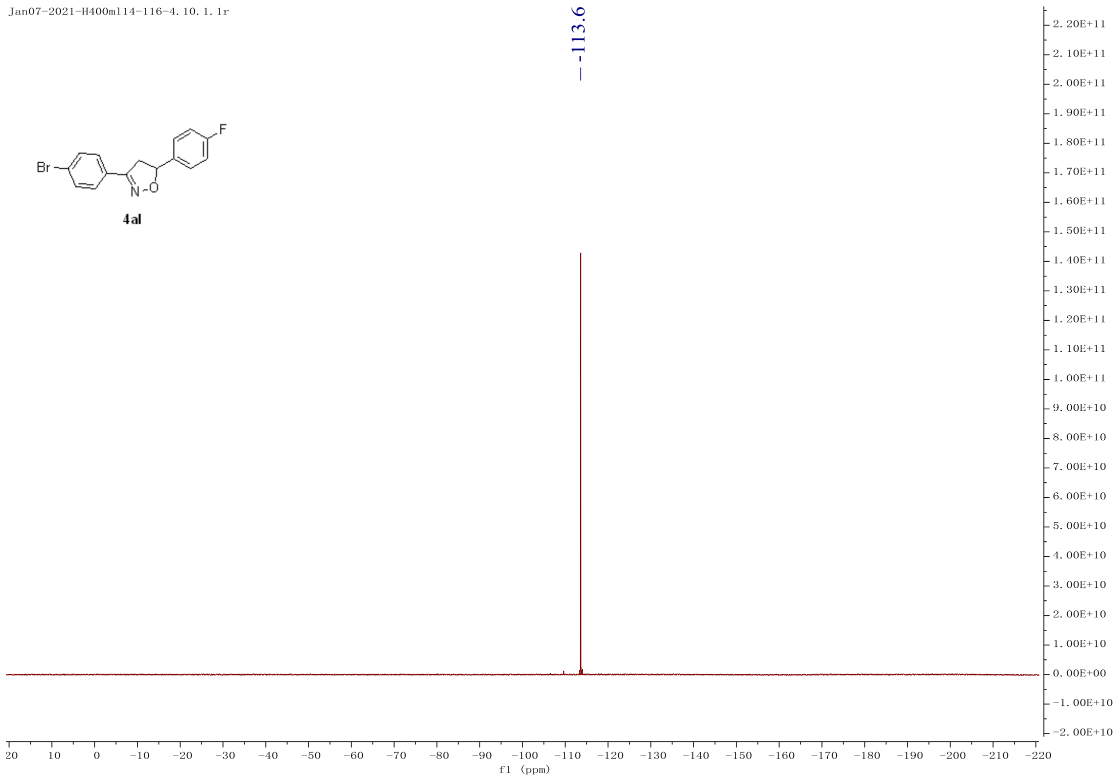
Jan07-2021-H400ml14-116-4.10.1.1r
-113.6
Br
F
N-O
4al
f1 (ppm)

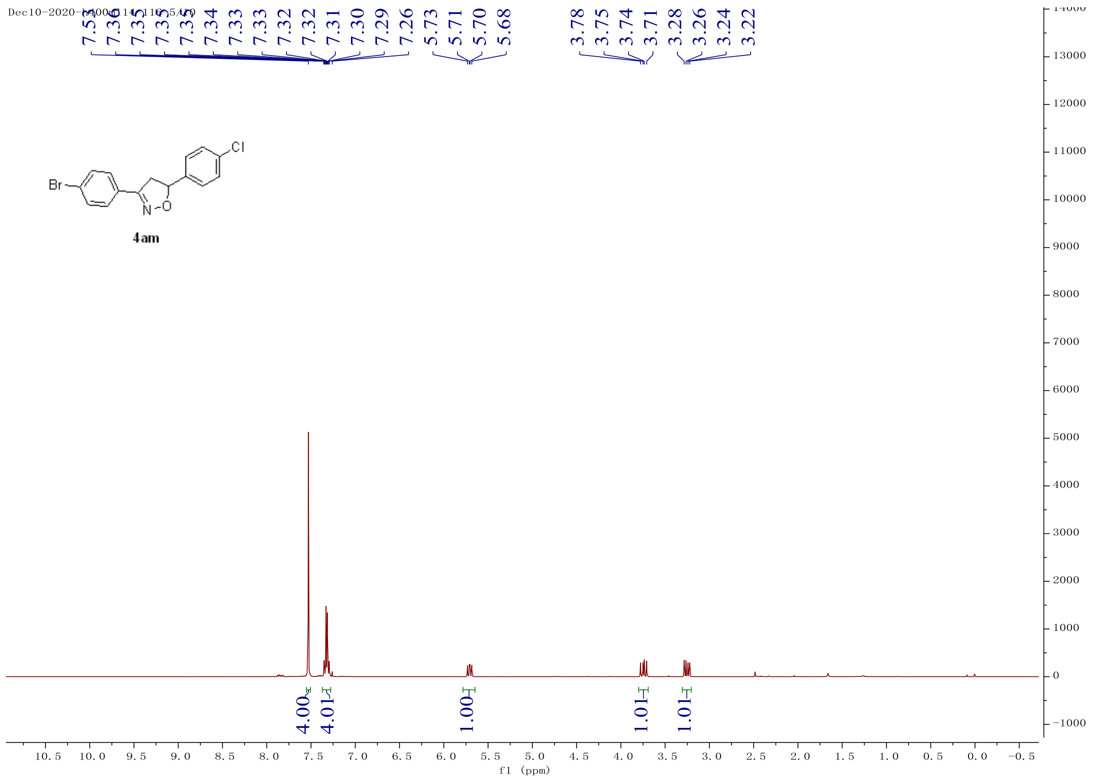
7.53
7.36
7.35
7.35
7.35
7.34
7.33
7.33
7.32
7.32
7.31
7.30
7.29
7.26
5.73
5.71
5.70
5.68
3.78
3.75
3.74
3.71
3.28
3.26
3.24
3.22
Br
Cl
N-O
4am
4.00
4.01
1.00
1.01
1.01
f1 (ppm)

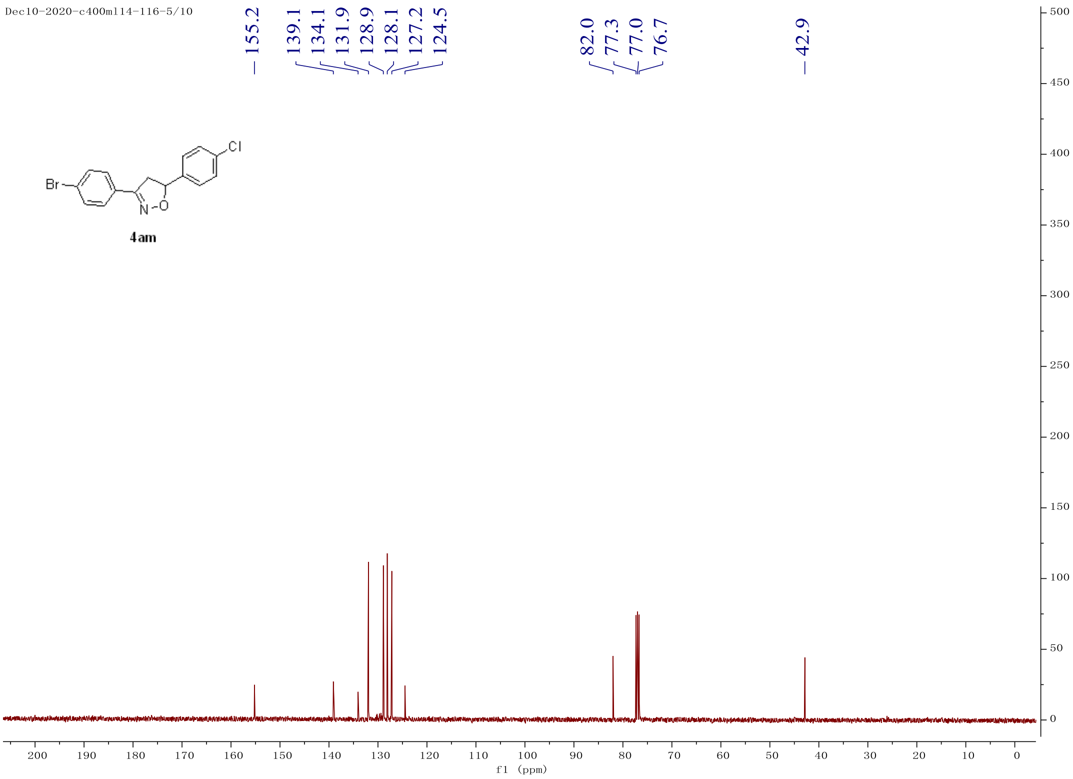
Dec10-2020-c400ml14-116-5/10
155.2
139.1
134.1
131.9
128.9
128.1
127.2
124.5
82.0
77.3
77.0
76.7
42.9
Br
Cl
N
O
4am
f1 (ppm)

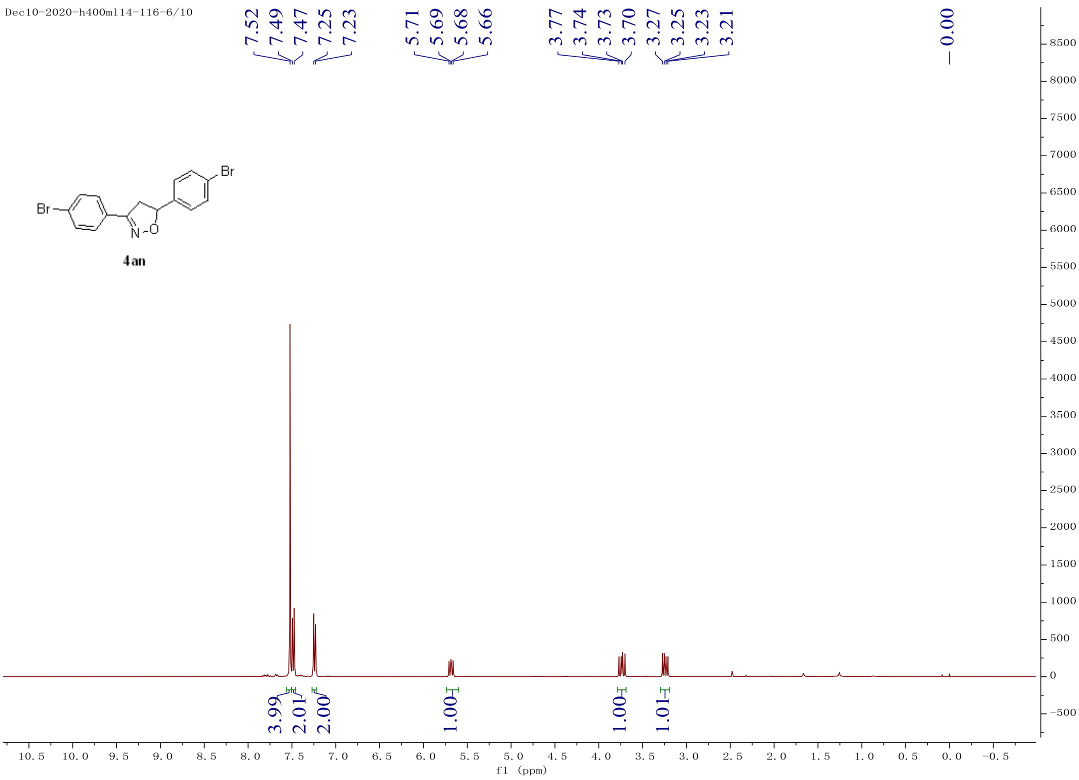
Dec10-2020-h400ml14-116-6/10
7.52
7.49
7.47
7.25
7.23
5.71
5.69
5.68
5.66
3.77
3.74
3.73
3.70
3.27
3.25
3.23
3.21
0.00
Br
Br
N
O
4an
3.99
2.01
2.00
1.00
1.00
1.01
f1 (ppm)

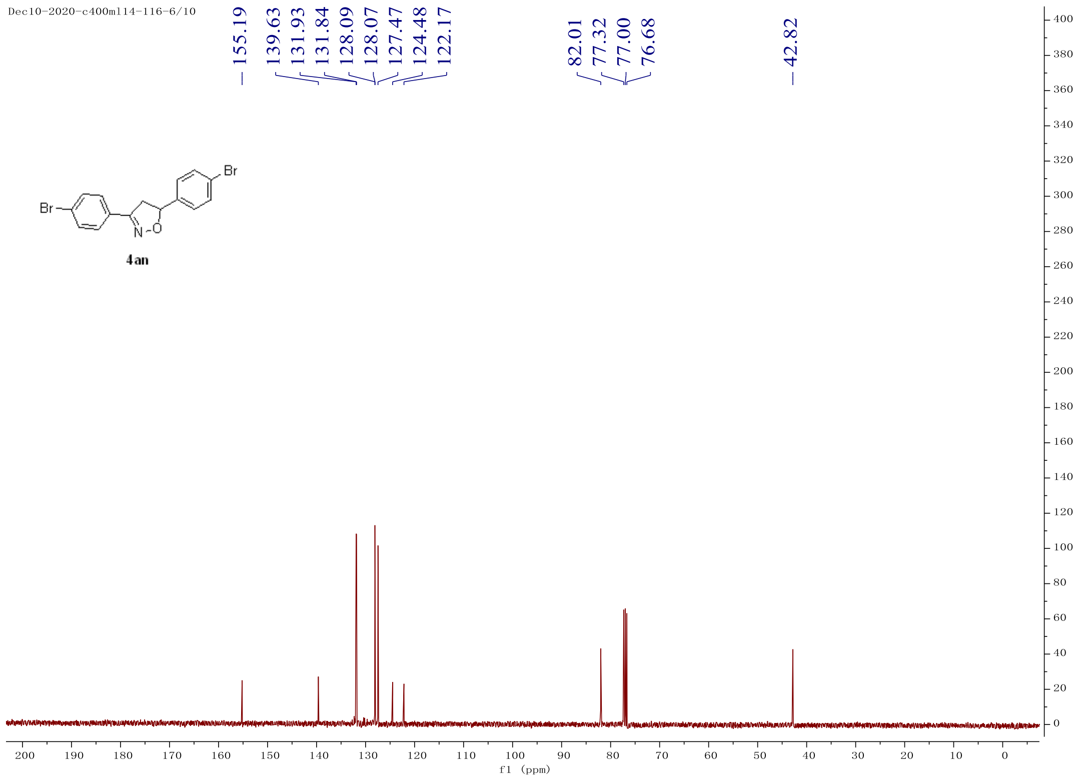
Dec10-2020-c400ml14-116-6/10
155.19
139.63
131.93
131.84
128.09
128.07
127.47
124.48
122.17
82.01
77.32
77.00
76.68
42.82
Br
Br
N
O
4an
f1 (ppm)

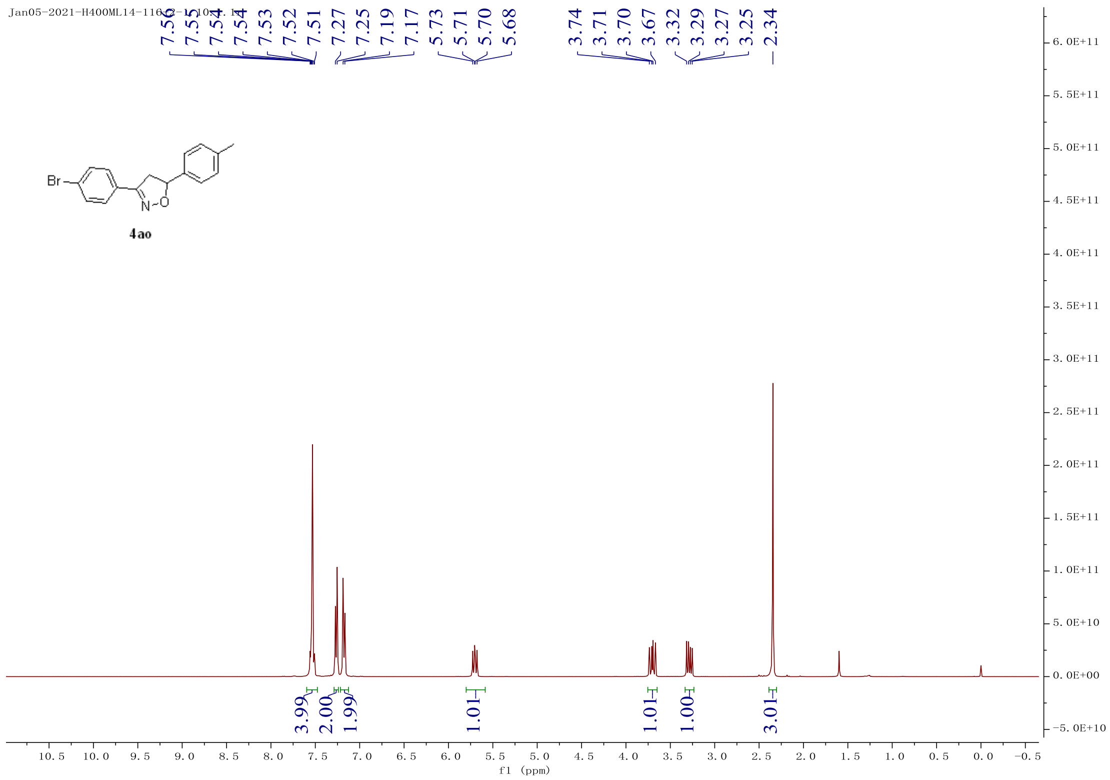
7.56
7.55
7.54
7.54
7.53
7.52
7.51
7.27
7.25
7.19
7.17
5.73
5.71
5.70
5.68
3.74
3.71
3.70
3.67
3.32
3.29
3.27
3.25
2.34
Br
N
O
4ao
3.99
2.00
1.99
1.01
1.01
1.00
3.01
f1 (ppm)

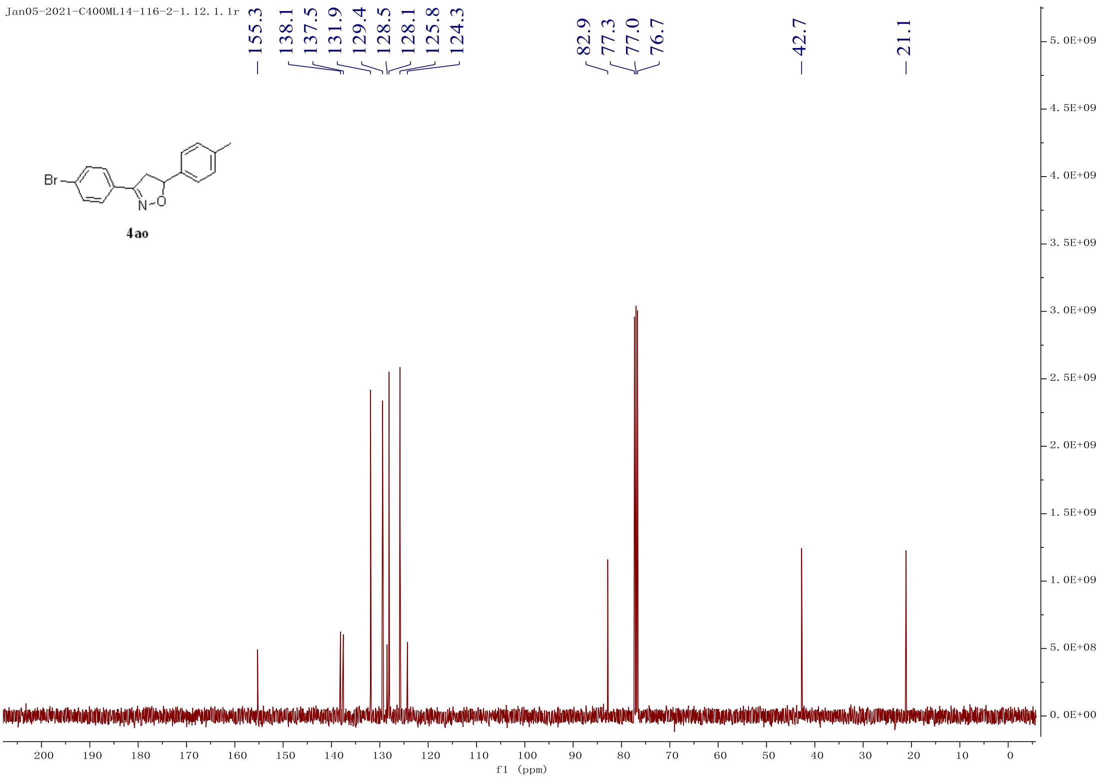

Jan05-2021-C400ML14-116-2-1.12.1.1r
155.3
138.1
137.5
131.9
129.4
128.5
128.1
125.8
124.3
82.9
77.3
77.0
76.7
42.7
21.1
Br
N
O
4ao
f1 (ppm)

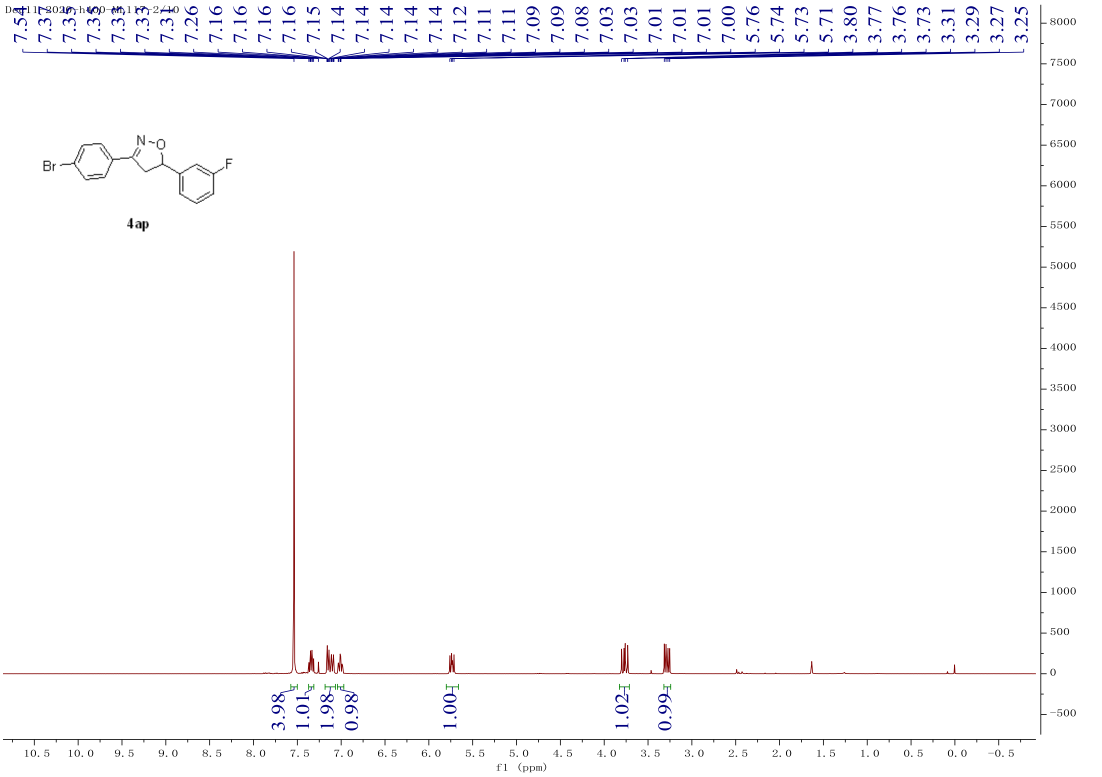

7.54
7.37
7.35
7.35
7.33
7.33
7.31
7.26
7.16
7.16
7.16
7.16
7.15
7.14
7.14
7.14
7.14
7.12
7.11
7.11
7.09
7.09
7.08
7.03
7.03
7.01
7.01
7.01
7.00
5.76
5.74
5.73
5.71
3.80
3.77
3.76
3.73
3.31
3.29
3.27
3.25
Br
N
O
F
4ap
3.98
1.01
1.98
0.98
1.00
1.02
0.99
f1 (ppm)

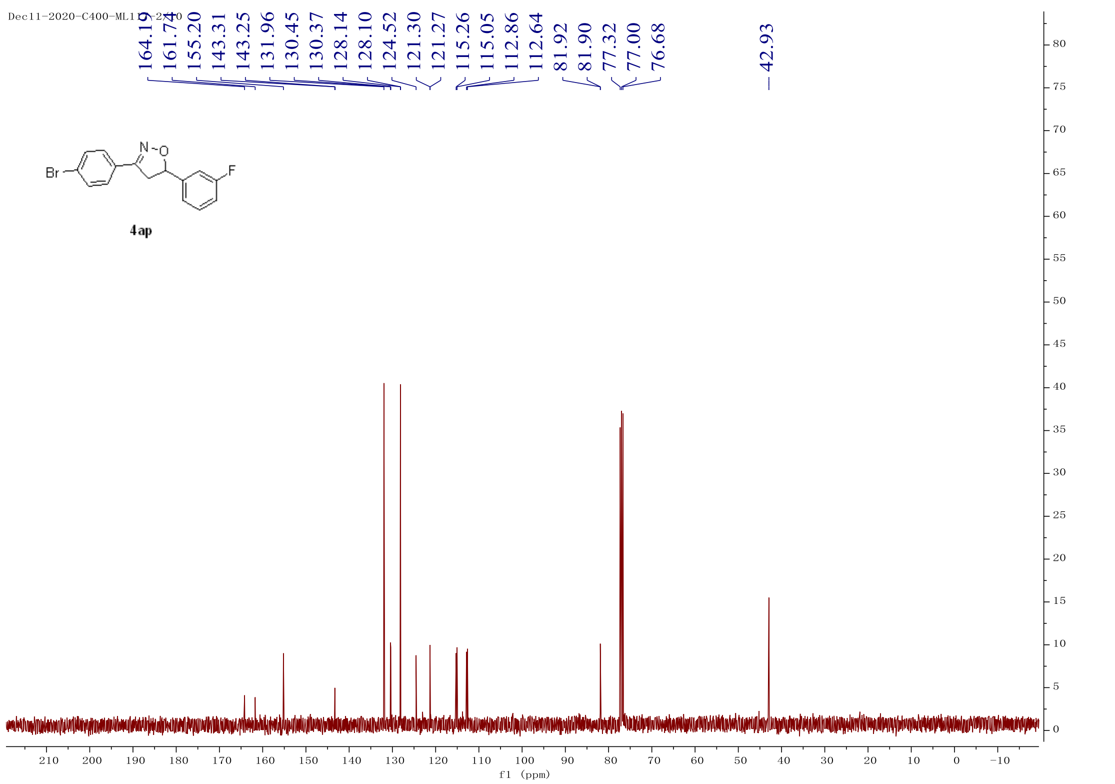
Dec11-2020-C400-ML11-7-2.10
164.19
161.74
155.20
143.31
143.25
131.96
130.45
130.37
128.14
128.10
124.52
121.30
121.27
115.26
115.05
112.86
112.64
81.92
81.90
77.32
77.00
76.68
42.93
Br
N
O
F
4ap
f1 (ppm)

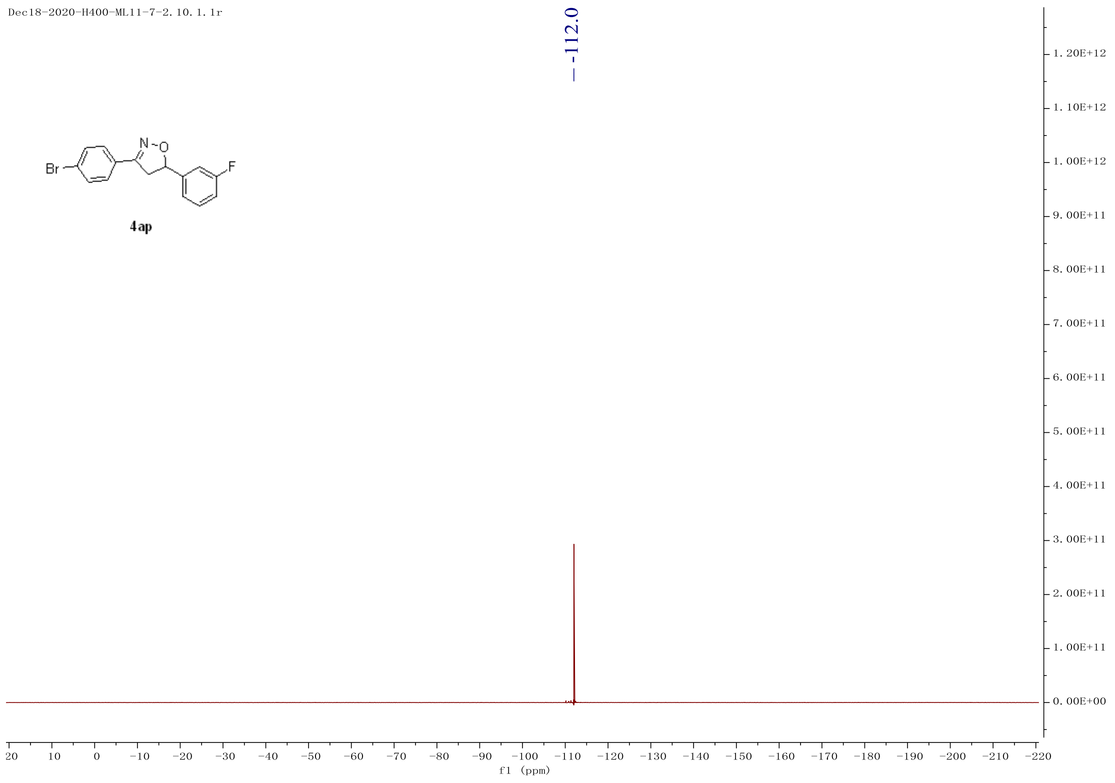
Dec18-2020-H400-ML11-7-2.10.1.1r
-112.0
Br
N
O
F
4ap
f1 (ppm)

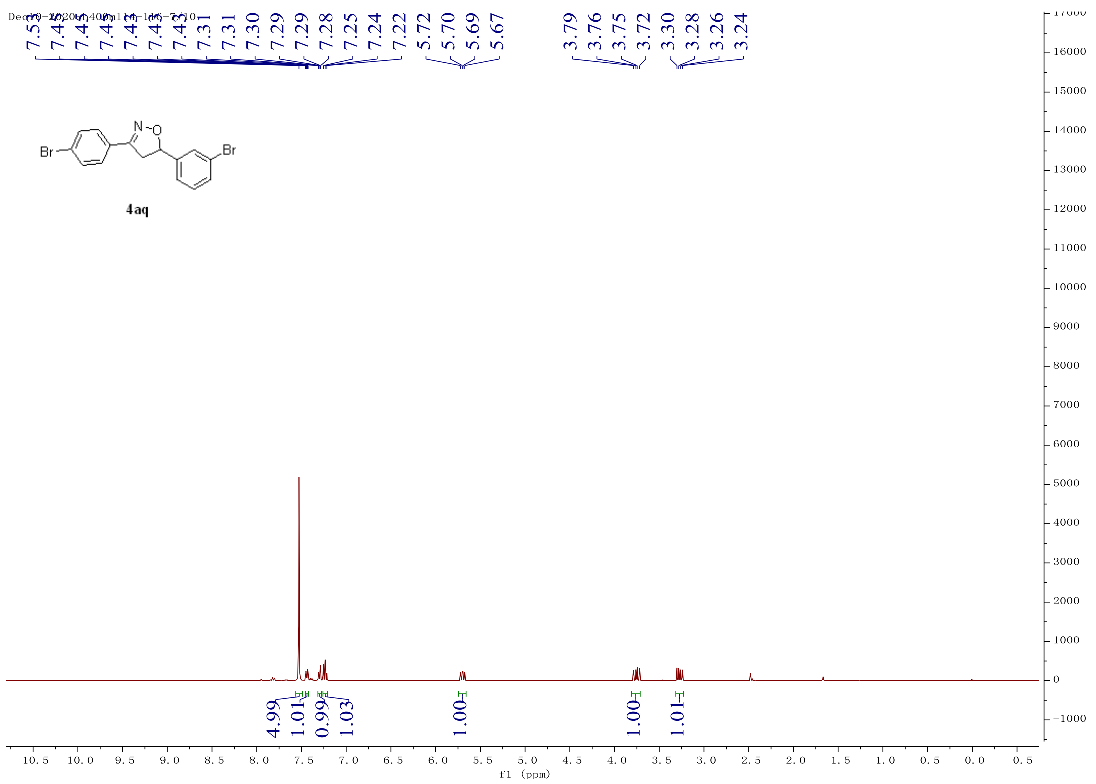

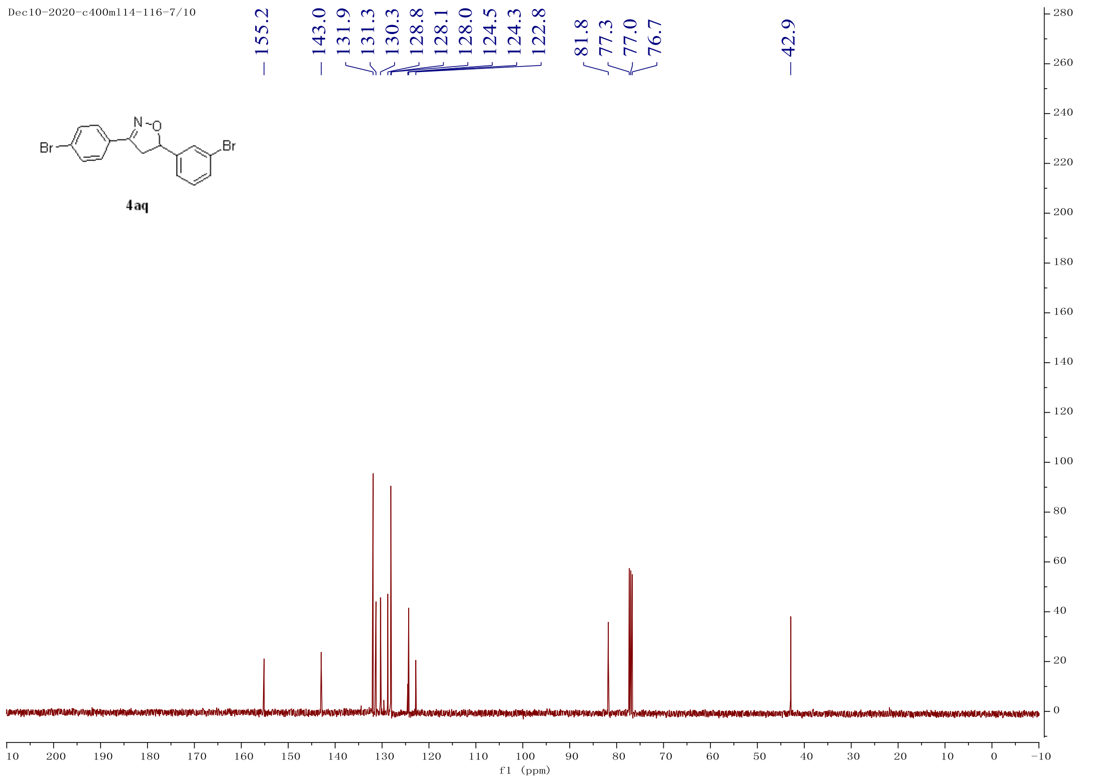

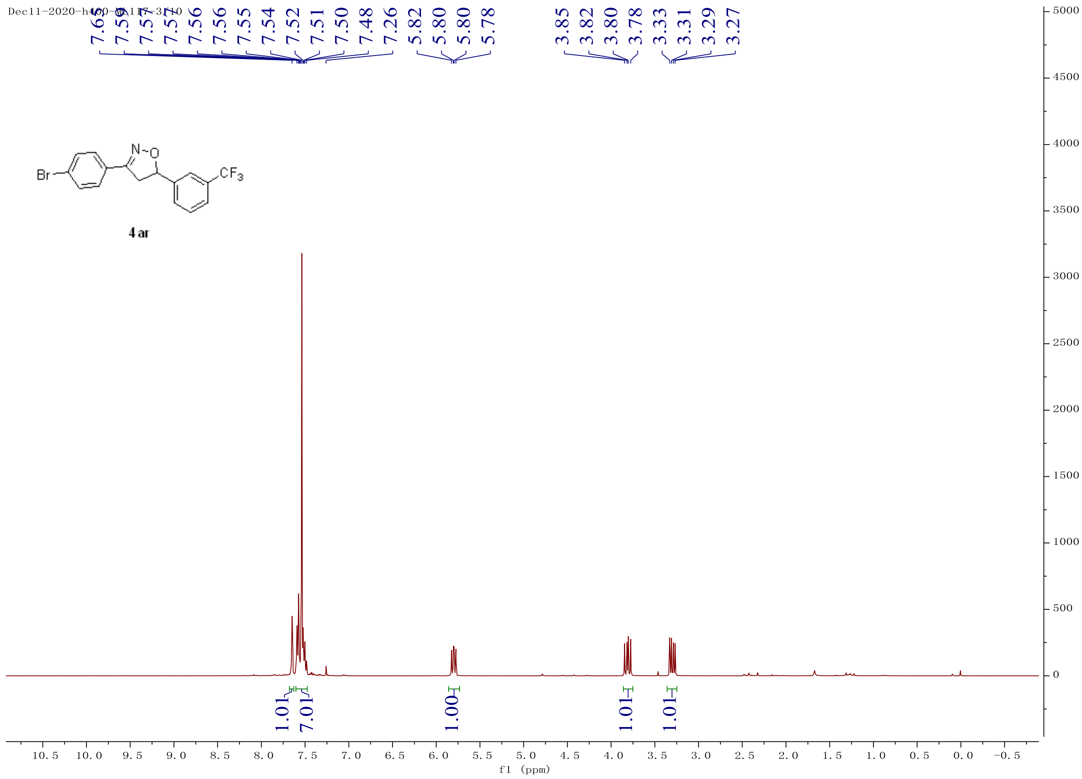

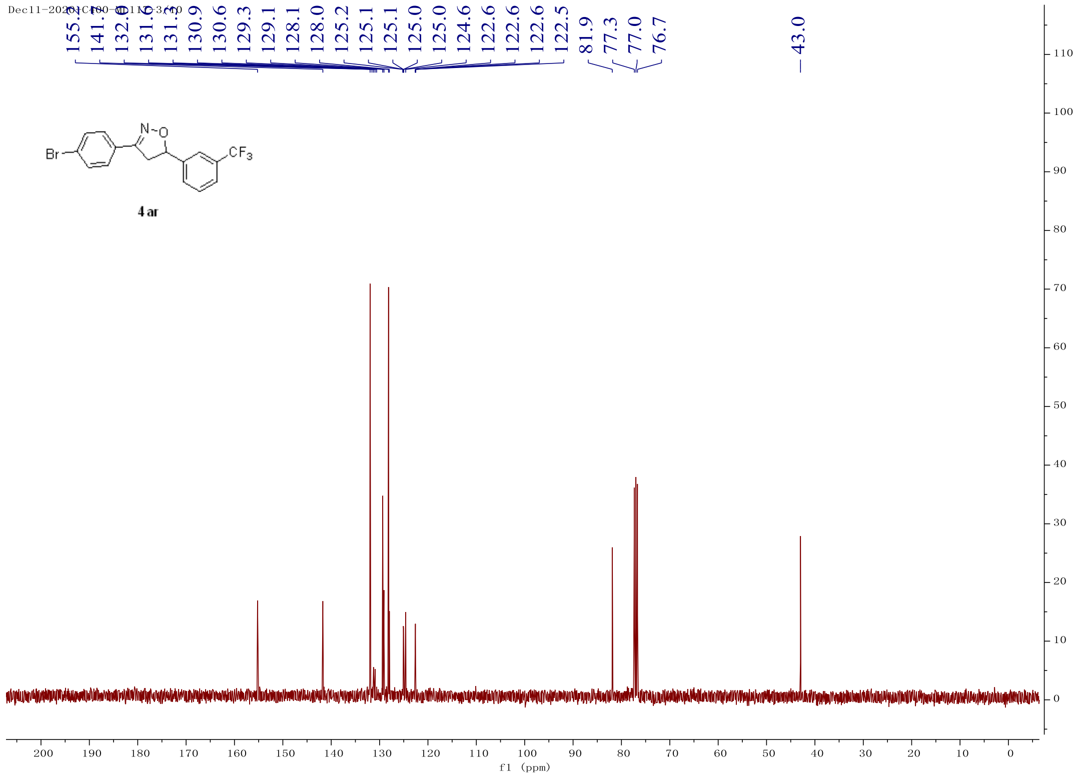

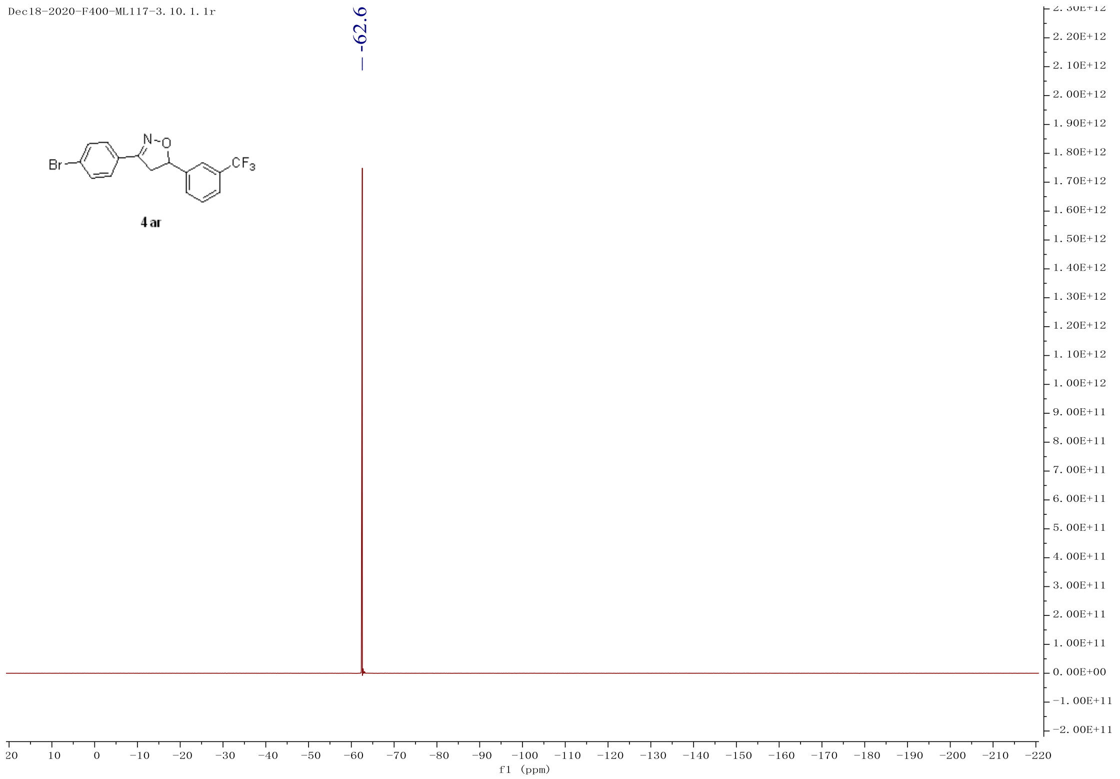
Dec18-2020-F400-ML117-3.10.1.1r
-62.6
Br
N-O
CF3
4 ar
f1 (ppm)

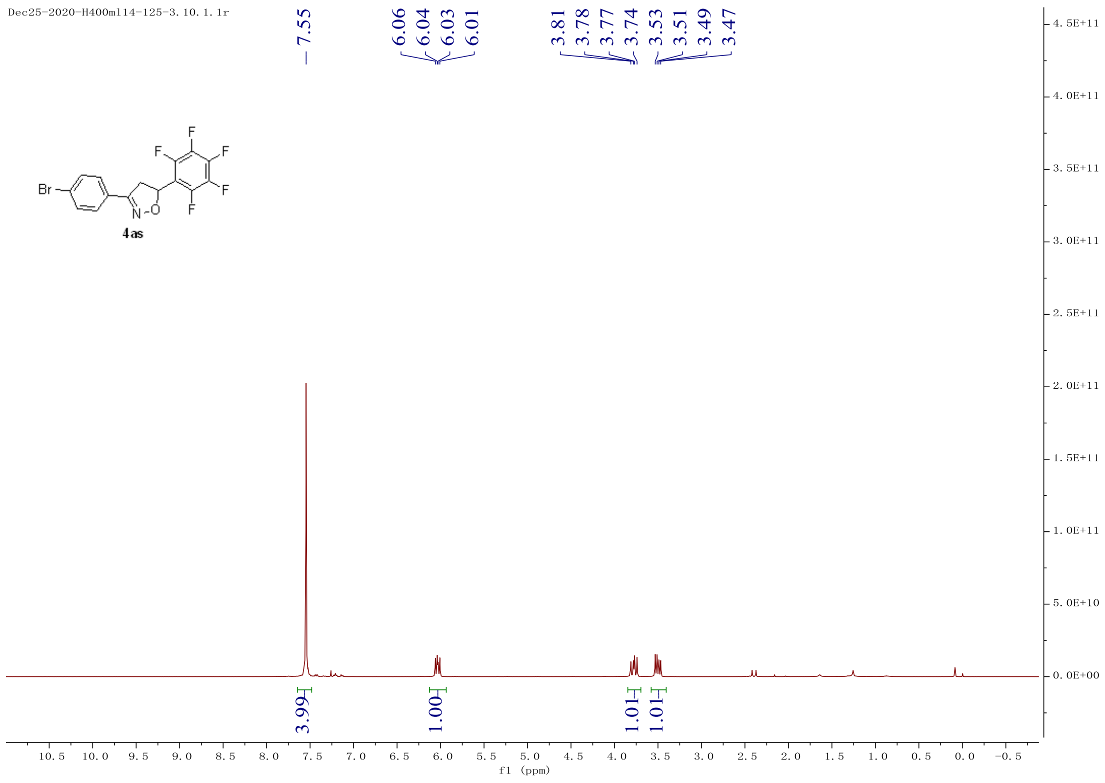
Dec25-2020-H400ml14-125-3.10.1.1r
7.55
6.06
6.04
6.03
6.01
3.81
3.78
3.77
3.74
3.53
3.51
3.49
3.47
Br
F
N-O
4as
3.99
1.00
1.01
1.01
f1 (ppm)

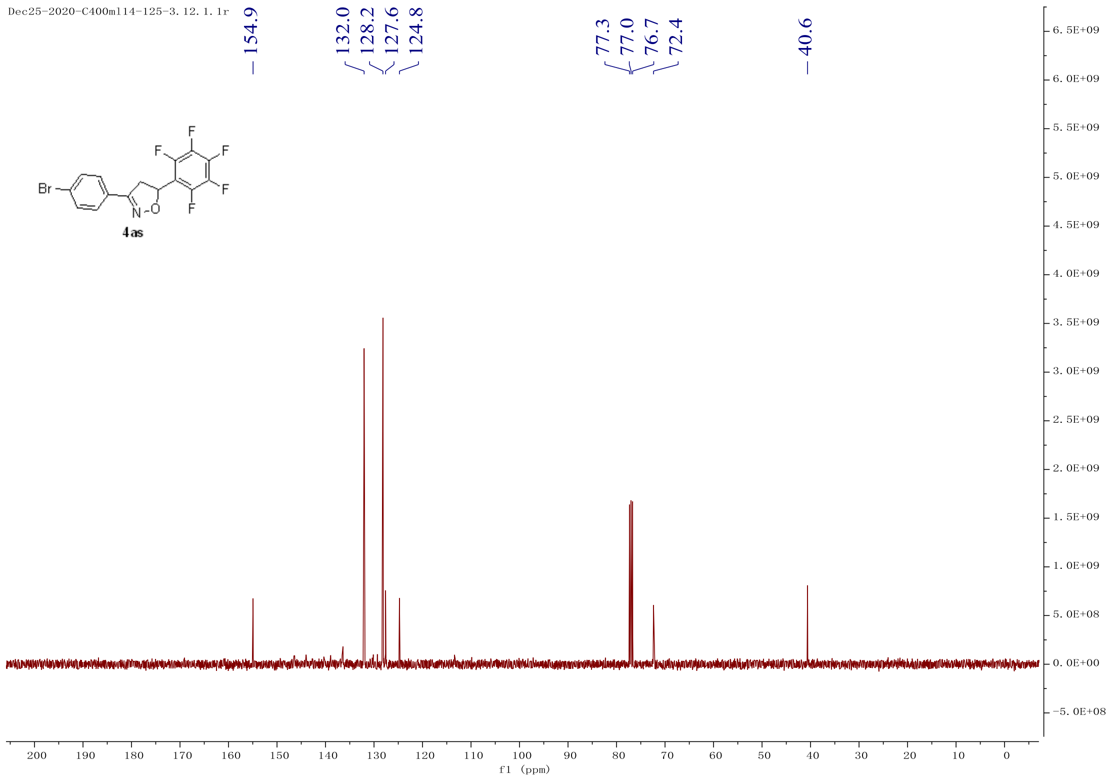
Dec25-2020-C400ml14-125-3.12.1.1r
154.9
132.0
128.2
127.6
124.8
77.3
77.0
76.7
72.4
40.6
Br
N-O
F
4as
f1 (ppm)

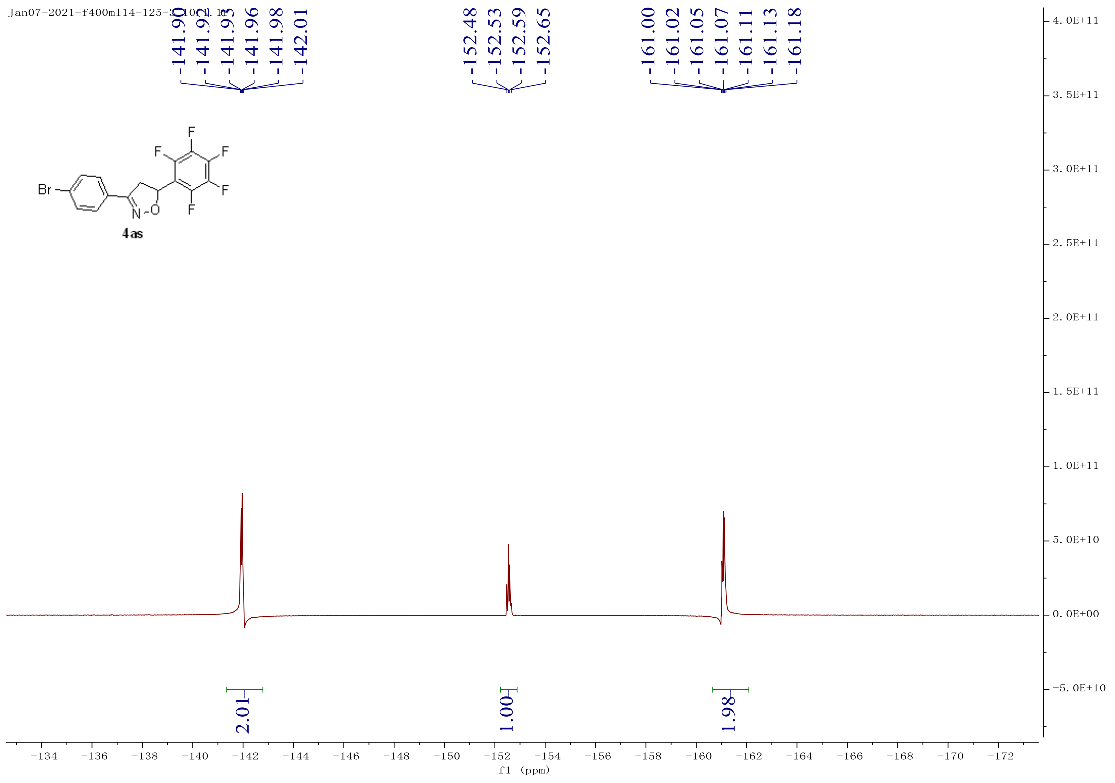
-141.90
-141.92
-141.93
-141.96
-141.98
-142.01
-152.48
-152.53
-152.59
-152.65
-161.00
-161.02
-161.05
-161.07
-161.11
-161.13
-161.18
Br
N-O
F
4as
2.01
1.00
1.98
f1 (ppm)

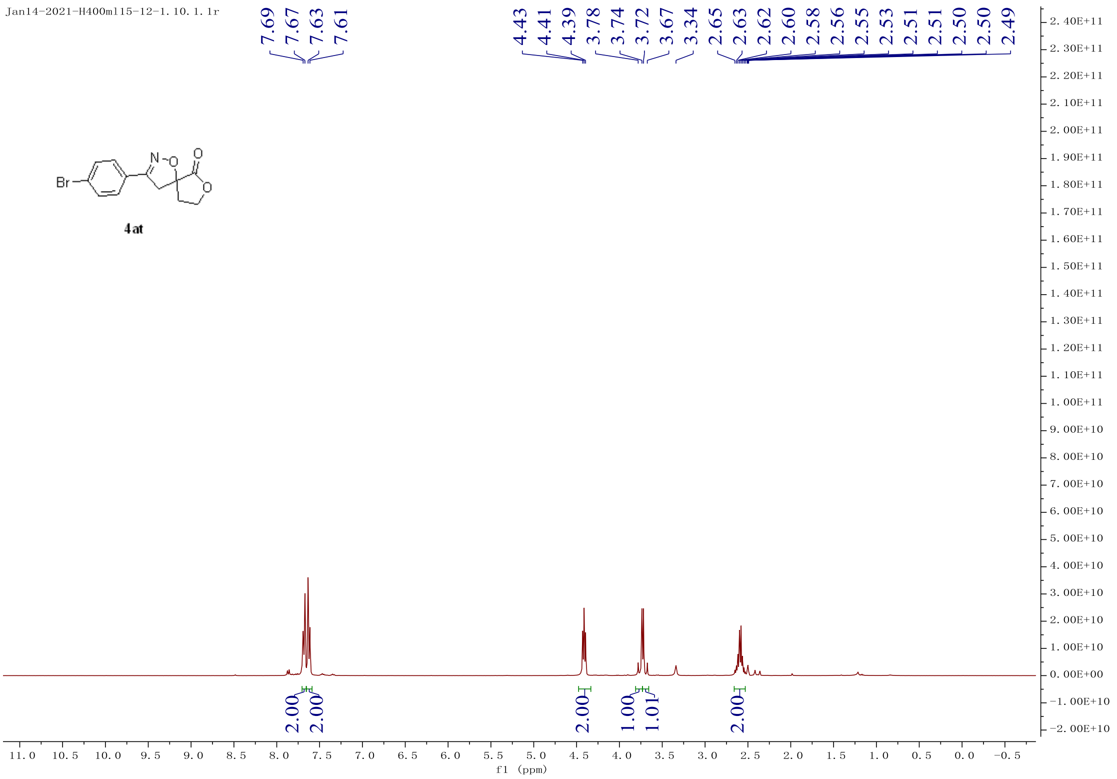
Jan14-2021-H400ml15-12-1.10.1.1r
7.69
7.67
7.63
7.61
4.43
4.41
4.39
3.78
3.74
3.72
3.67
3.34
2.65
2.63
2.62
2.60
2.58
2.56
2.55
2.53
2.51
2.51
2.50
2.50
2.49
4at
2.00
2.00
2.00
1.00
1.01
2.00
f1 (ppm)

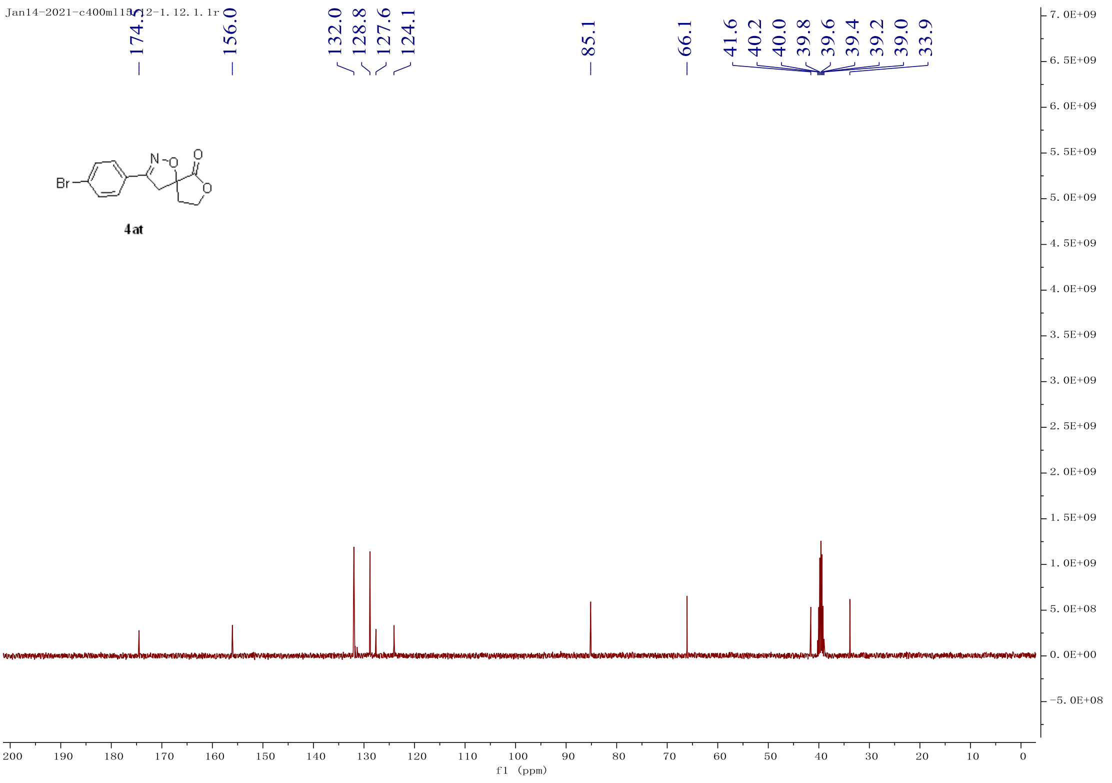
Jan14-2021-c400ml15-12-1.12.1.1r
174.5
156.0
132.0
128.8
127.6
124.1
85.1
66.1
41.6
40.2
40.0
39.8
39.6
39.4
39.2
39.0
33.9
4at
f1 (ppm)

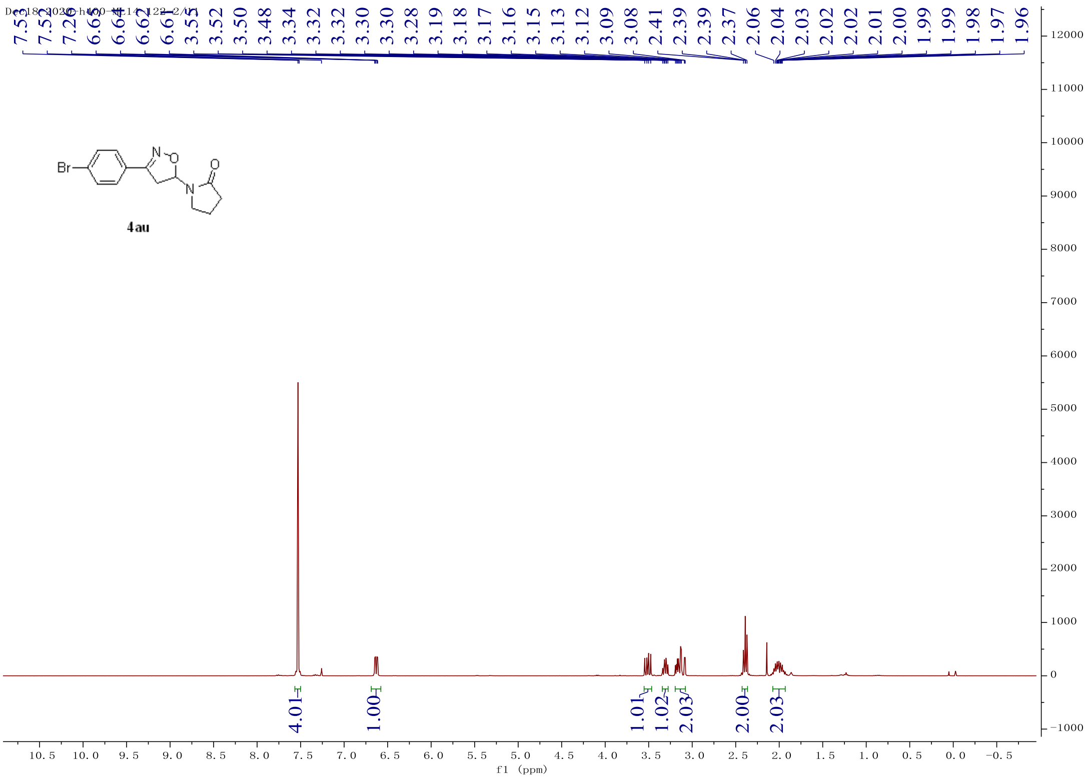
7.53
7.52
7.26
3.55
3.52
3.50
3.48
3.34
3.32
3.32
3.30
3.30
3.28
3.19
3.18
3.17
3.16
3.15
3.13
3.12
3.09
3.08
2.41
2.39
2.39
2.37
2.06
2.04
2.03
2.02
2.02
2.01
2.00
1.99
1.99
1.98
1.97
1.96
Br
N
O
N
O
4au
4.01
1.00
1.01
1.02
2.03
2.00
2.03
f1 (ppm)

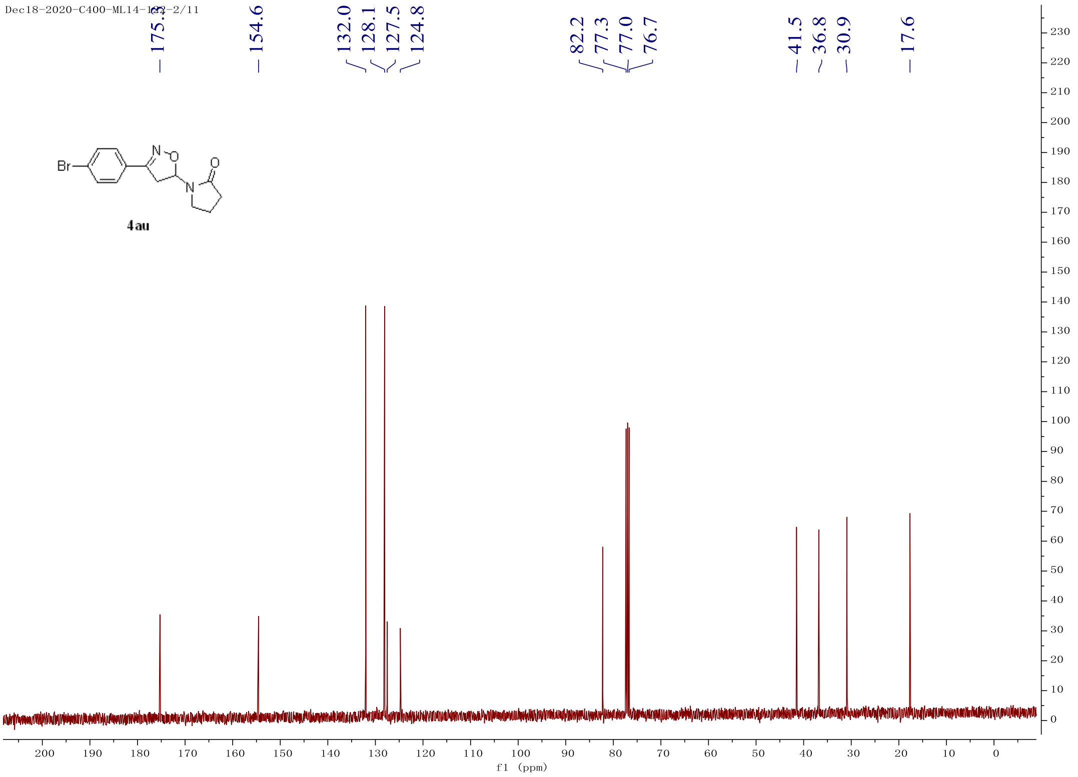
Dec18-2020-C400-ML14-122-2/11
154.6
132.0
128.1
127.5
124.8
82.2
77.3
77.0
76.7
41.5
36.8
30.9
17.6
Br
N
O
N
O
4au
f1 (ppm)

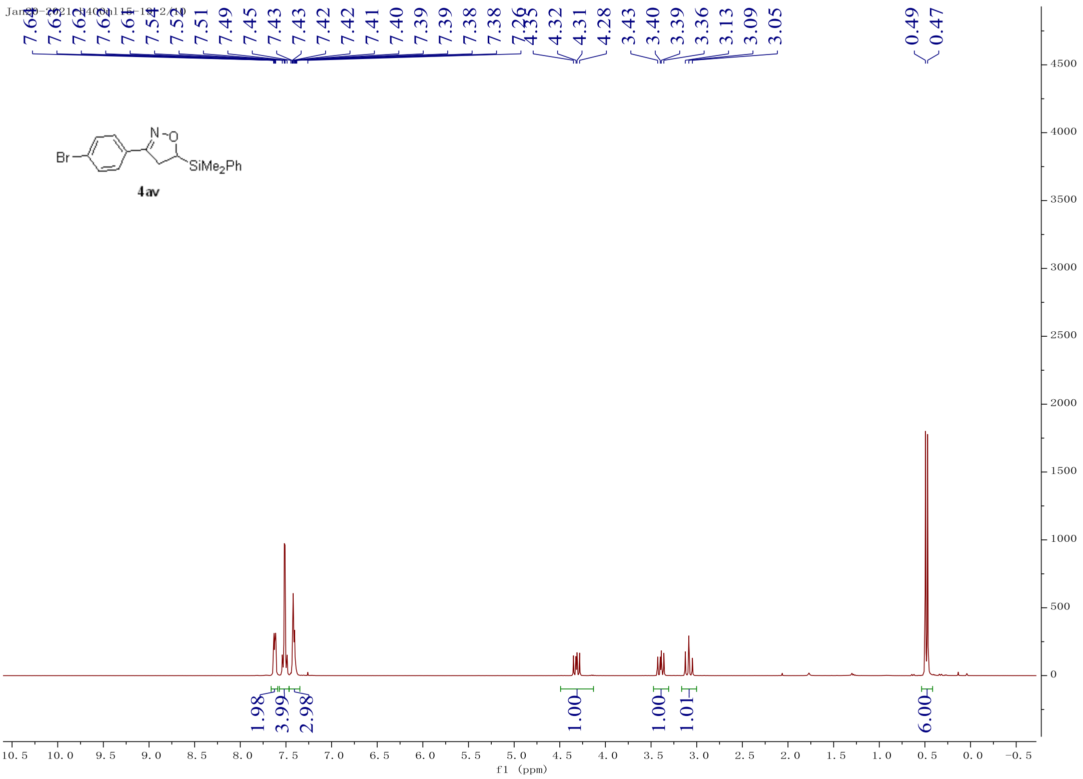

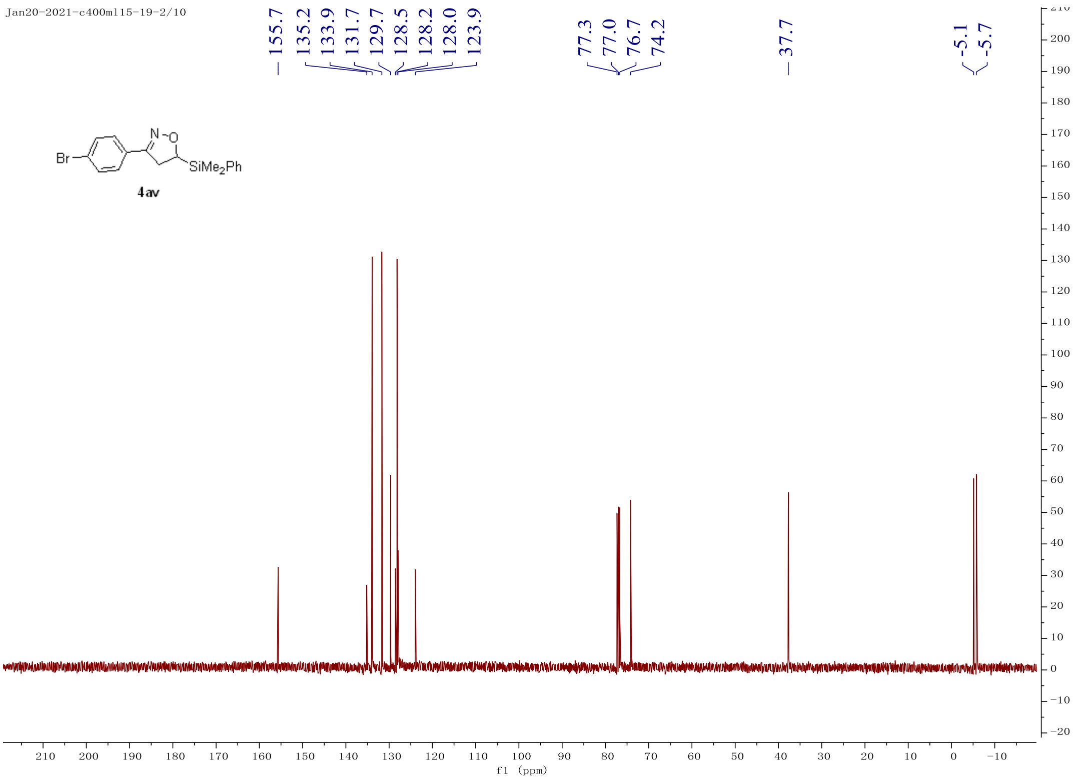

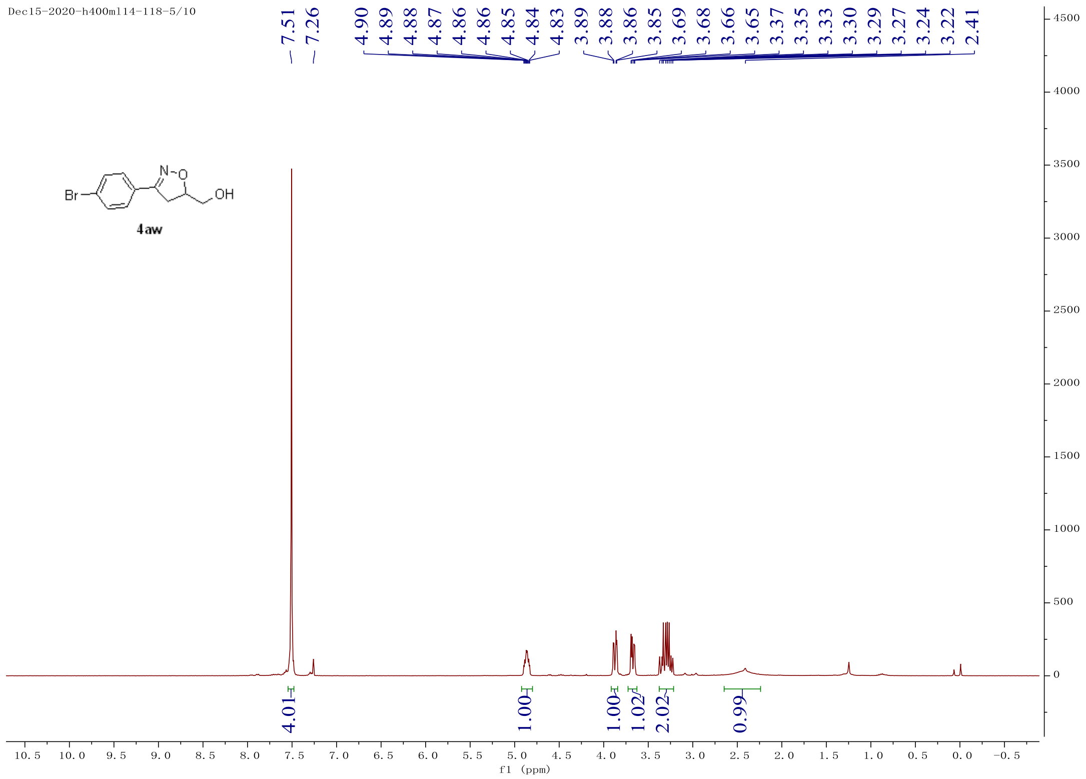
Dec15-2020-h400ml14-118-5/10
7.51
7.26
4.90
4.89
4.88
4.87
4.86
4.86
4.85
4.84
4.83
3.89
3.88
3.86
3.85
3.69
3.68
3.66
3.65
3.37
3.35
3.33
3.30
3.29
3.27
3.24
3.22
2.41
Br
N
O
OH
4aw
4.01
1.00
1.00
1.02
2.02
0.99
f1 (ppm)

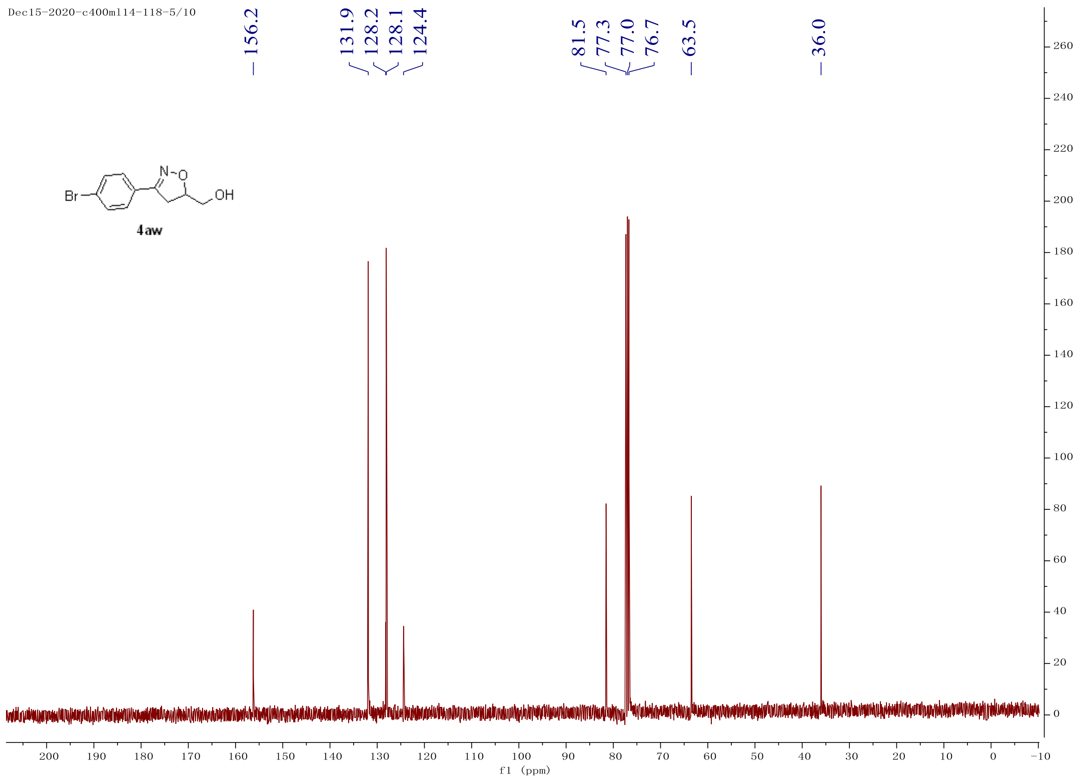
Dec15-2020-c400ml14-118-5/10
156.2
131.9
128.2
128.1
124.4
81.5
77.3
77.0
76.7
63.5
36.0
Br
N
O
OH
4aw
f1 (ppm)

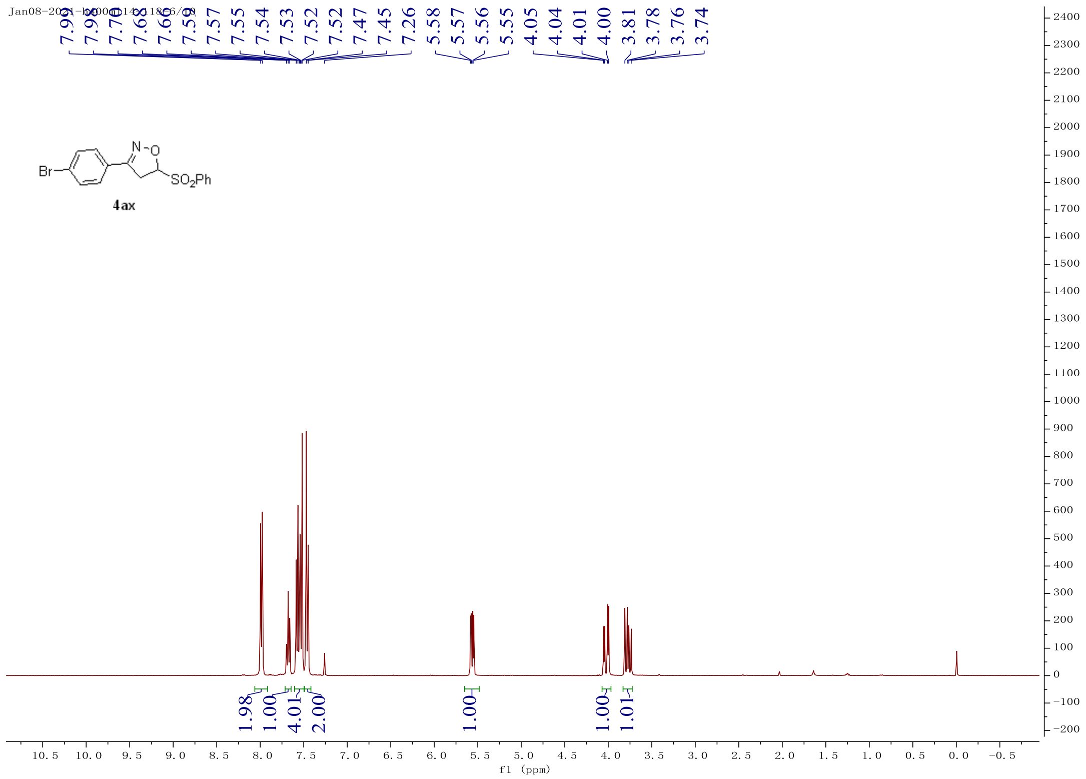
7.99 7.96 7.70 7.68 7.66 7.59 7.57 7.55 7.54 7.53 7.52 7.52 7.47 7.45 7.26 5.58 5.57 5.56 5.55 4.05 4.04 4.01 4.00 3.81 3.78 3.76 3.74
Br
N
O
SO2Ph
4ax
1.98
1.00
4.01
2.00
1.00
1.00
1.01
f1 (ppm)

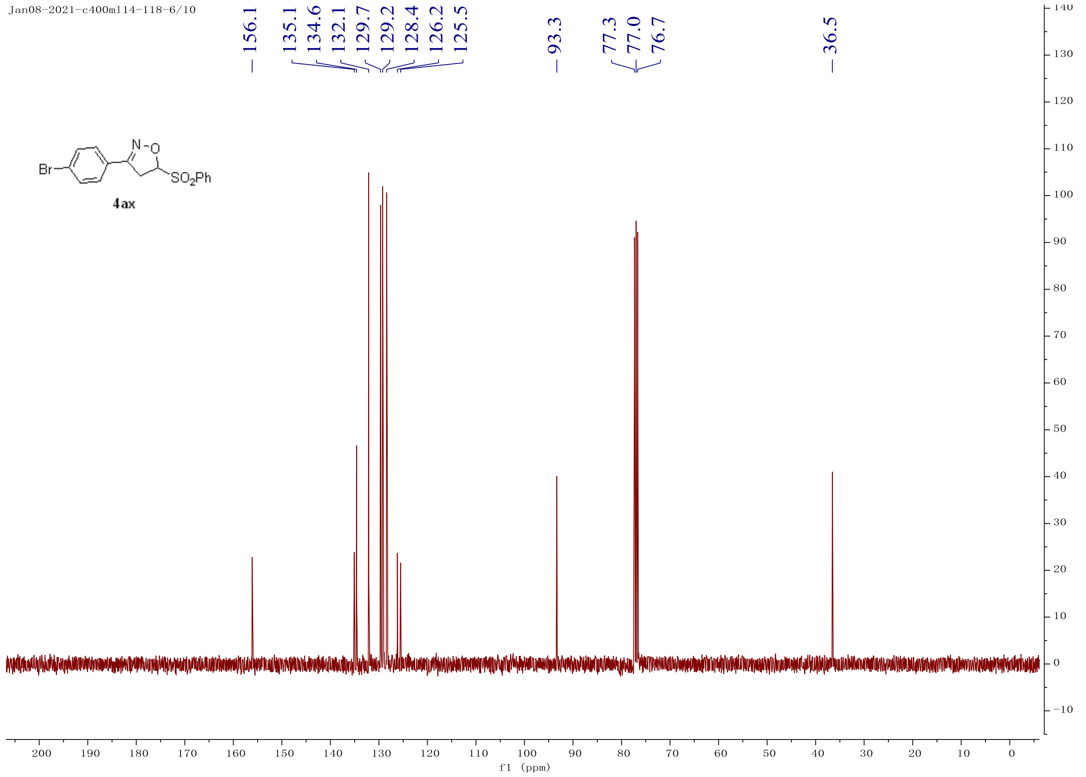
Jan08-2021-c400ml14-118-6/10
156.1
135.1 134.6 132.1 129.7 129.2 128.4 126.2 125.5
93.3
77.3 77.0 76.7
36.5
Br
N
O
SO2Ph
4ax
f1 (ppm)

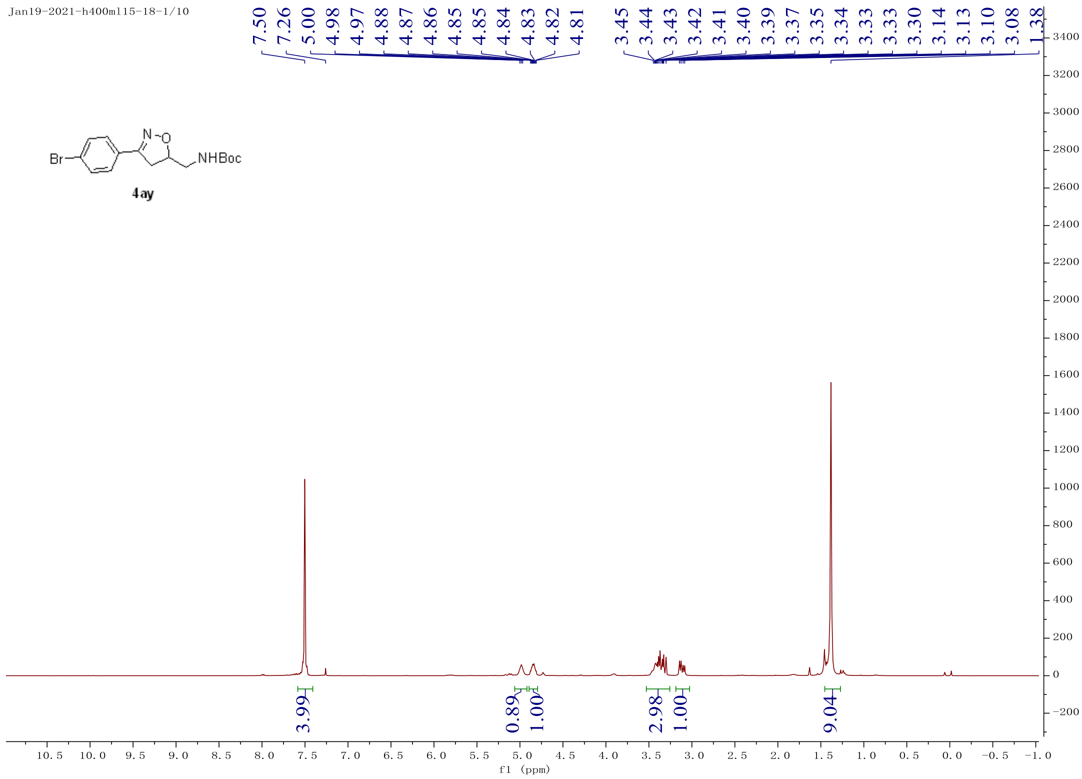

Jan19-2021-h400ml15-18-1/10
7.50 7.26 5.00 4.98 4.97 4.88 4.87 4.86 4.85 4.85 4.84 4.83 4.82 4.81
3.45 3.44 3.43 3.42 3.41 3.40 3.39 3.37 3.35 3.34 3.33 3.33 3.30 3.14 3.13 3.10 3.08 1.38
Br
N
O
NHBoc
4ay
3.99
0.89
1.00
2.98
1.00
9.04
f1 (ppm)

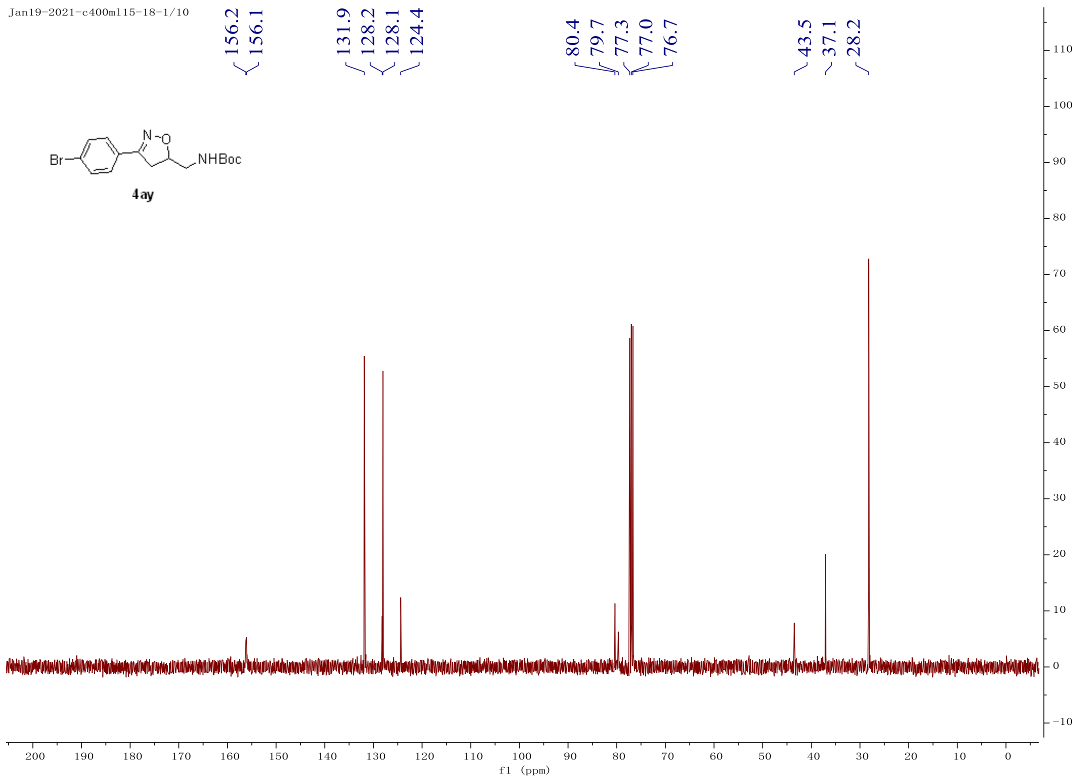

Jan19-2021-c400ml15-18-1/10
156.2 156.1
131.9 128.2 128.1 124.4
80.4 79.7 77.3 77.0 76.7
43.5 37.1 28.2
Br
N
O
NHBoc
4ay
f1 (ppm)

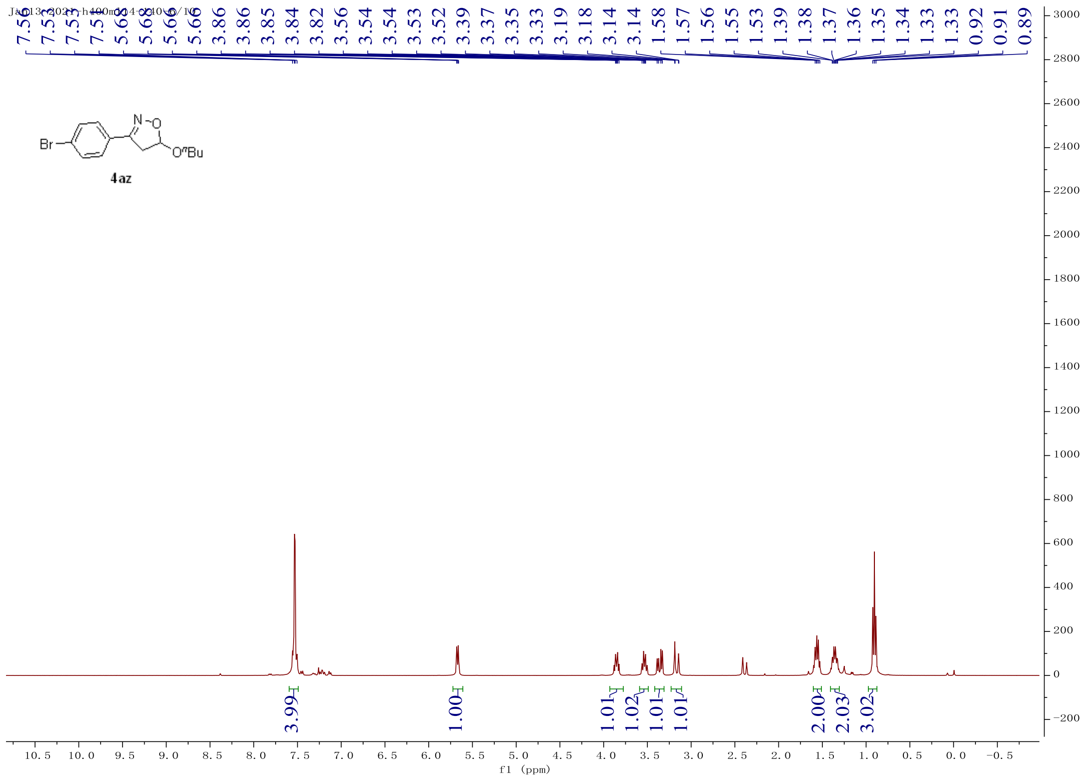
7.56 7.53 7.53 7.51 5.68 5.68 5.66 5.66 3.86 3.86 3.85 3.84 3.82 3.56 3.54 3.54 3.53 3.52 3.39 3.37 3.35 3.33 3.19 3.18 3.14 3.14 1.58 1.57 1.56 1.55 1.53 1.39 1.38 1.37 1.36 1.35 1.34 1.33 1.33 0.92 0.91 0.89
Br
N
O
O^{n}Bu
4az
3.99
1.00
1.01
1.02
1.01
1.01
2.00
2.03
3.02
f1 (ppm)

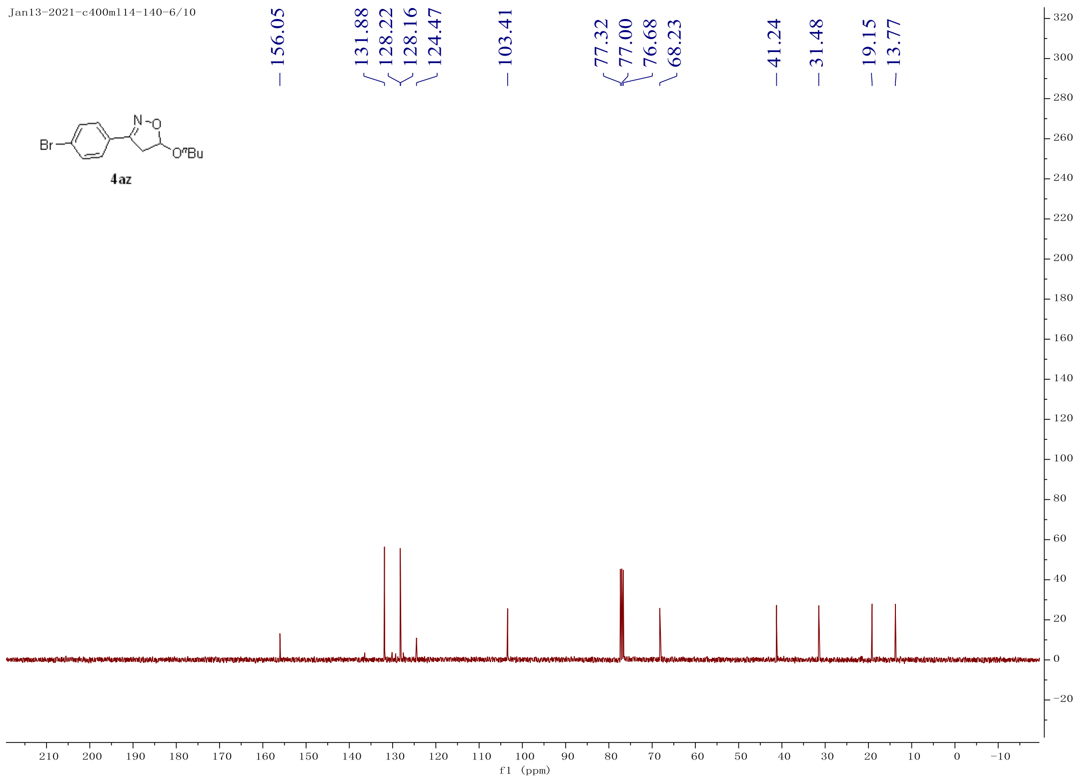
Jan13-2021-c400ml14-140-6/10
156.05
131.88
128.22
128.16
124.47
103.41
77.32
77.00
76.68
68.23
41.24
31.48
19.15
13.77
Br
N
O
O^{n}Bu
4az
f1 (ppm)

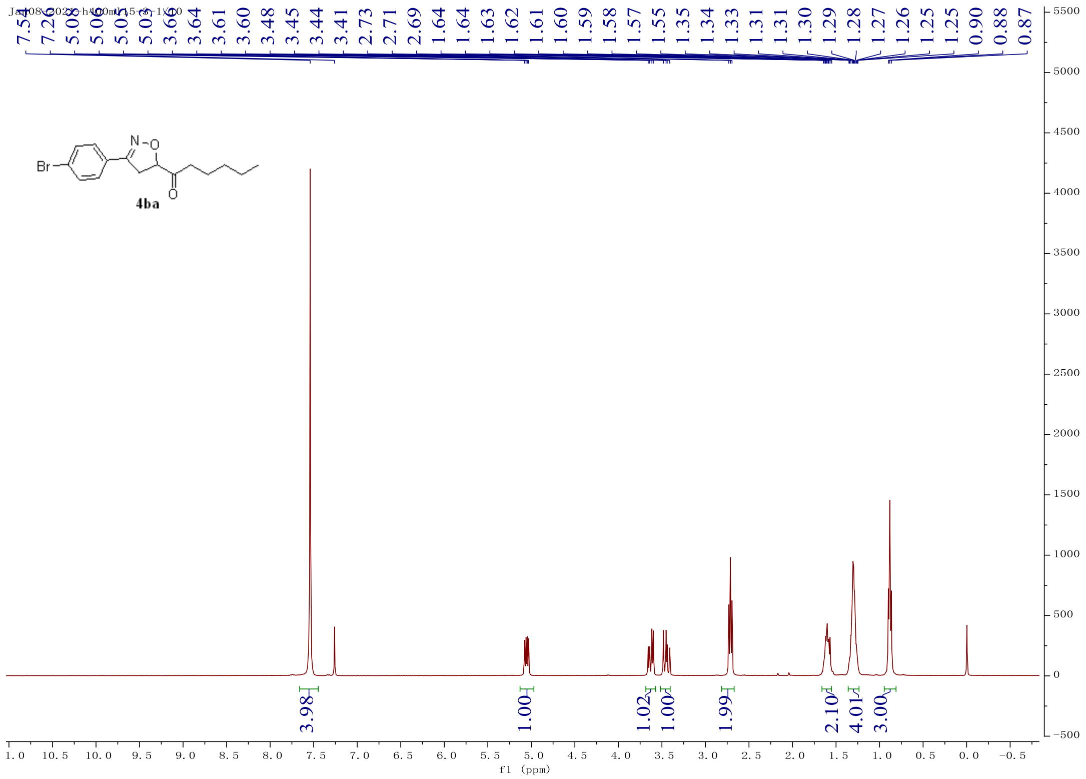
4ba
7.54
7.26
5.08
5.06
5.05
5.03
3.66
3.64
3.61
3.60
3.48
3.45
3.44
3.41
2.73
2.71
2.69
1.64
1.64
1.63
1.62
1.61
1.60
1.59
1.58
1.57
1.55
1.35
1.34
1.33
1.31
1.31
1.30
1.29
1.28
1.27
1.26
1.25
1.25
0.90
0.88
0.87
3.98
1.00
1.02
1.00
1.99
2.10
4.01
3.00
f1 (ppm)

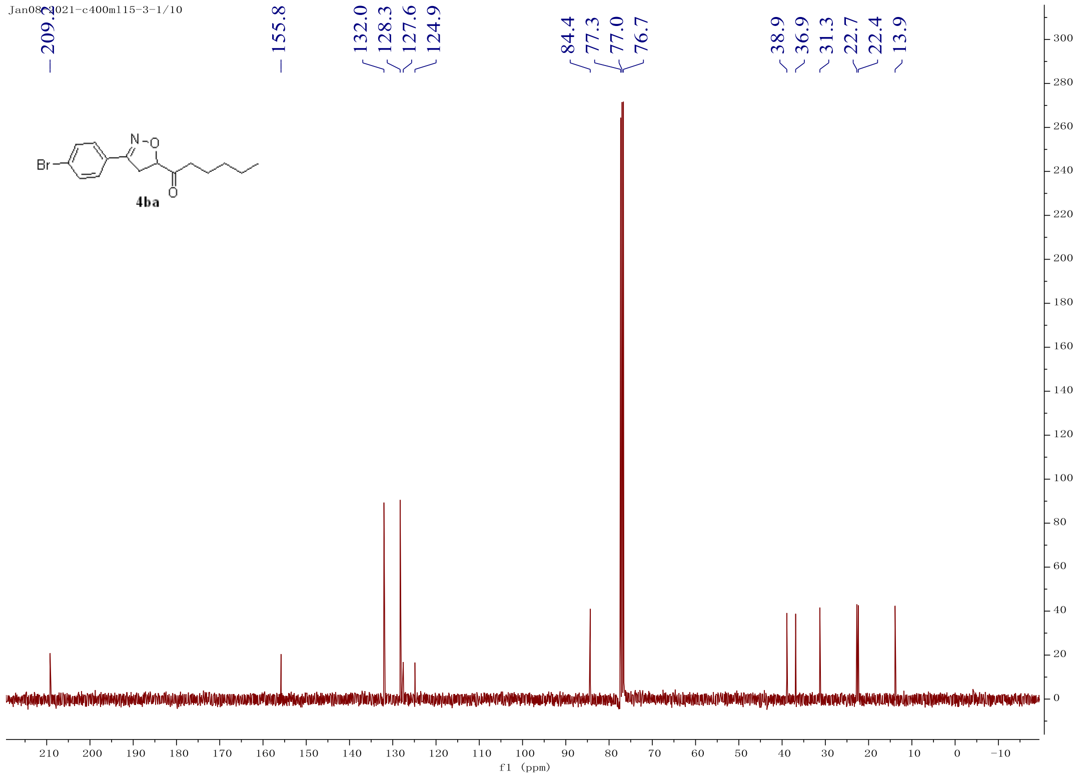
Jan08-2021-c400ml15-3-1/10
4ba
209.2
155.8
132.0
128.3
127.6
124.9
84.4
77.3
77.0
76.7
38.9
36.9
31.3
22.7
22.4
13.9
f1 (ppm)

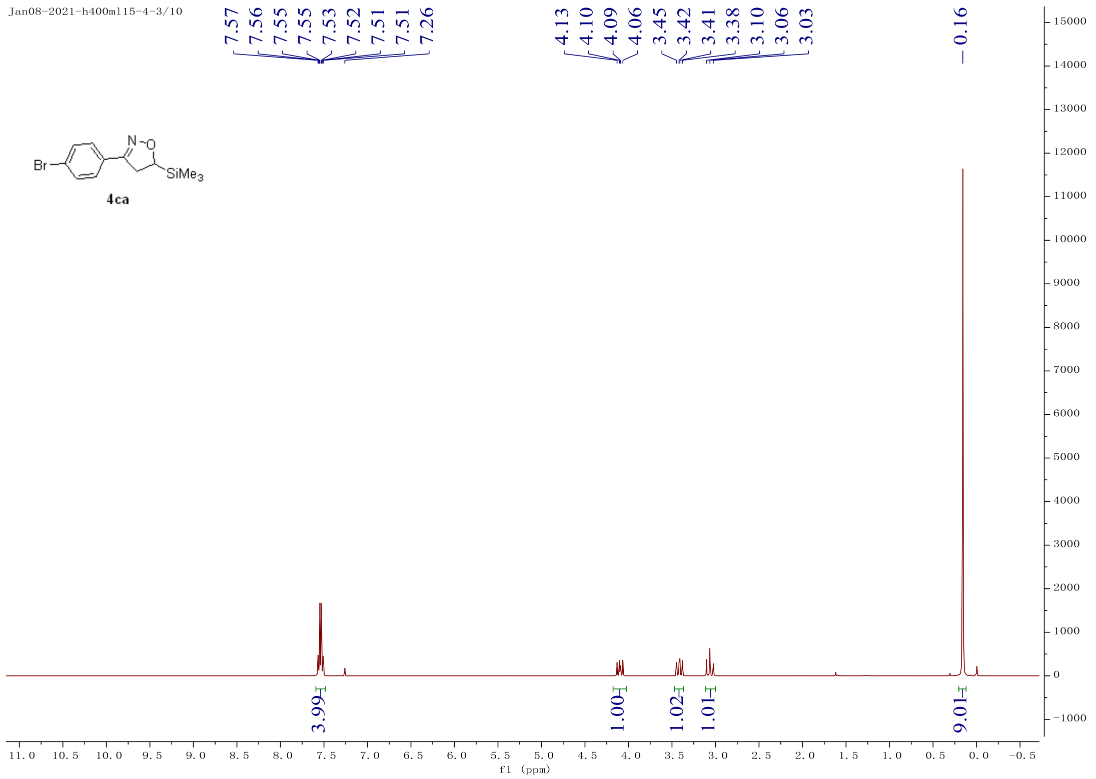
Jan08-2021-h400ml15-4-3/10
7.57 7.56 7.55 7.55 7.53 7.52 7.51 7.51 7.26
4.13 4.10 4.09 4.06 3.45 3.42 3.41 3.38 3.10 3.06 3.03
0.16
Br
N-O
SiMe3
4ca
3.99
1.00
1.02
1.01
9.01
f1 (ppm)

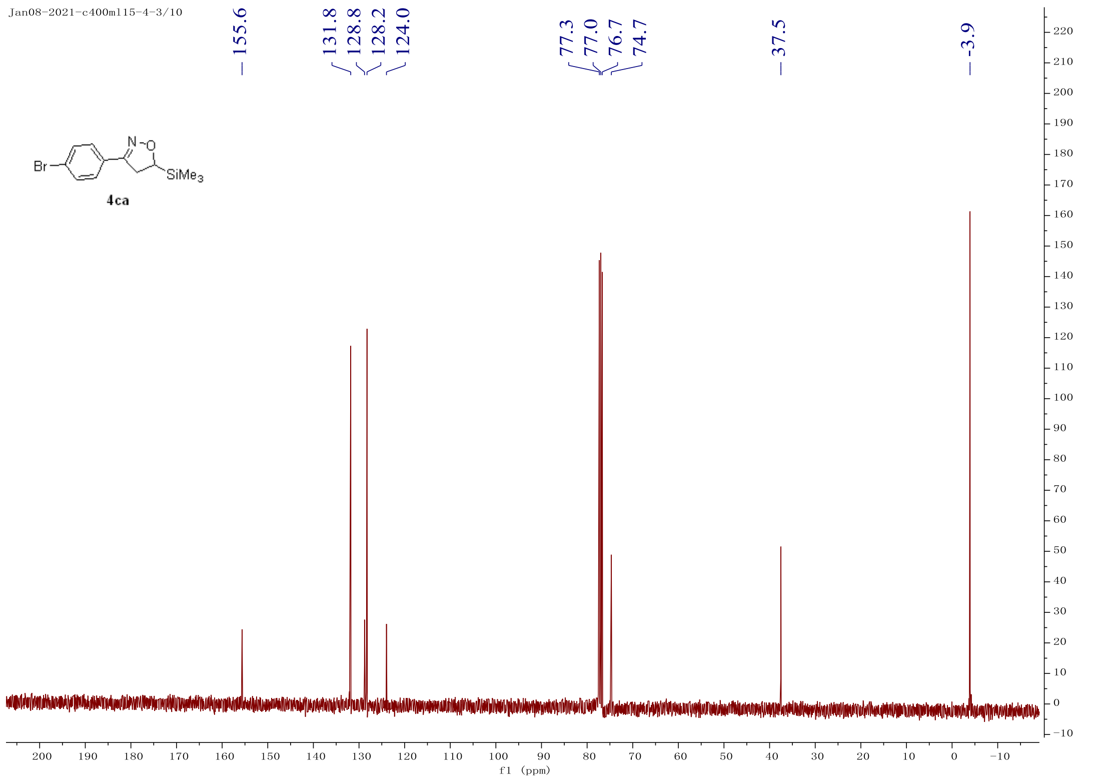
Jan08-2021-c400ml15-4-3/10
155.6
131.8 128.8 128.2 124.0
77.3 77.0 76.7 74.7
37.5
-3.9
Br
N-O
SiMe3
4ca
f1 (ppm)

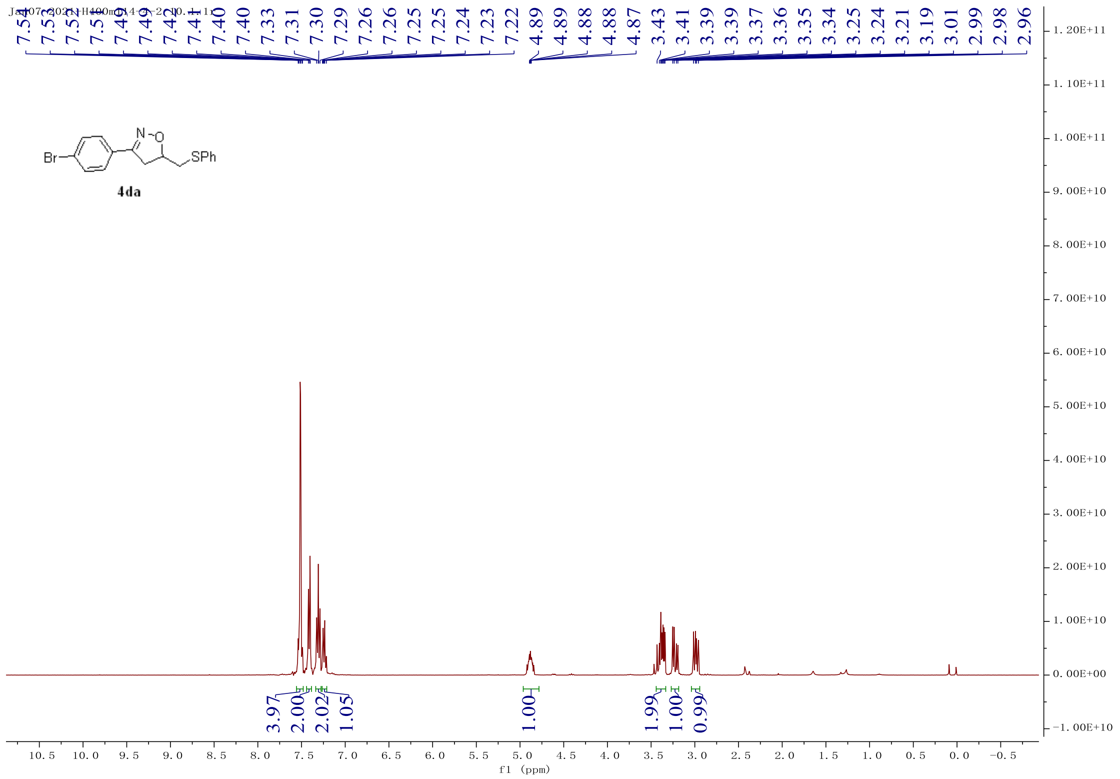

7.54
7.52
7.49
7.49
7.41
7.40
7.40
7.33
7.31
7.30
7.29
7.26
7.25
7.25
7.24
7.23
7.22
4.89
4.89
4.88
4.87
3.43
3.41
3.39
3.39
3.37
3.36
3.35
3.34
3.25
3.24
3.21
3.19
3.01
2.99
2.98
2.96
Br
N
O
SPh
4da
3.97
2.00
2.02
1.05
1.00
1.99
1.00
0.99
f1 (ppm)

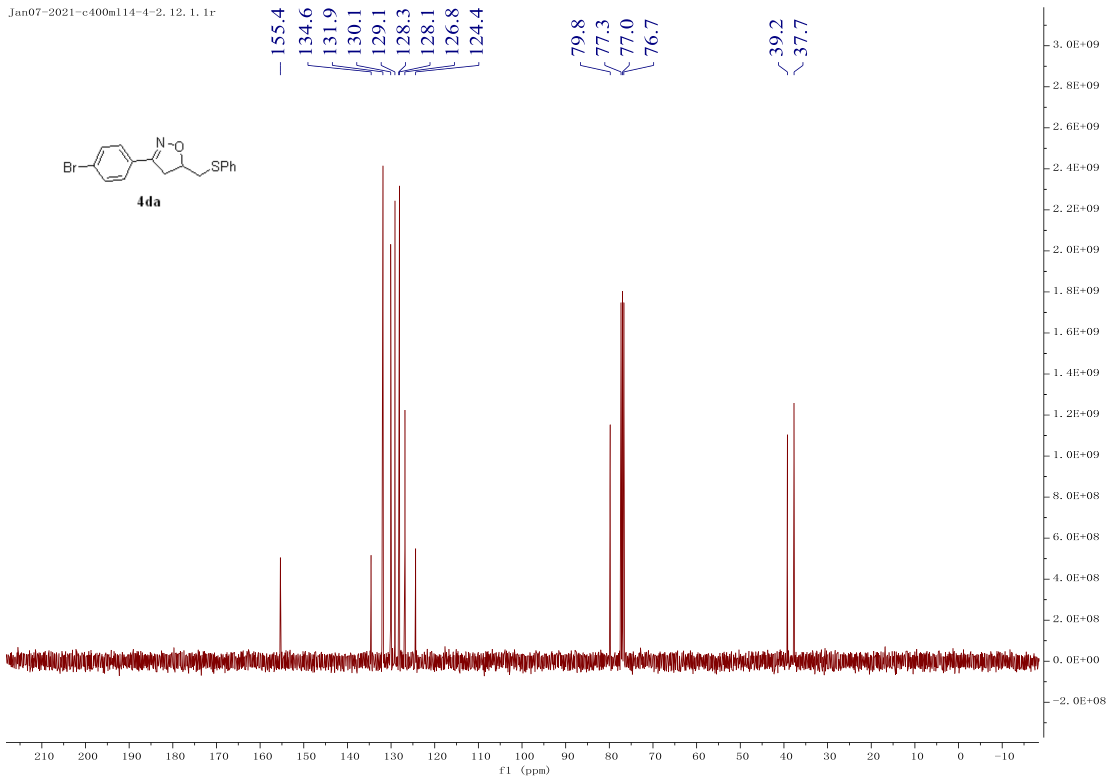

Jan07-2021-c400ml14-4-2.12.1.1r
155.4
134.6
131.9
130.1
129.1
128.3
128.1
126.8
124.4
79.8
77.3
77.0
76.7
39.2
37.7
Br
N
O
SPh
4da
f1 (ppm)

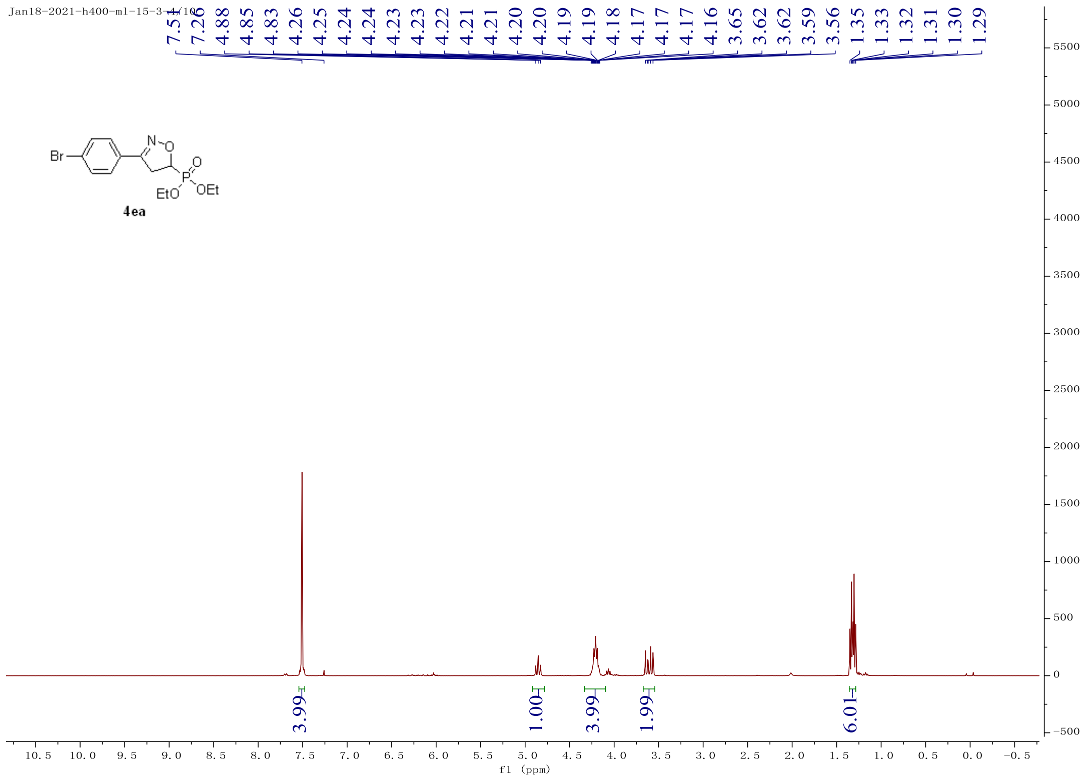
Jan18-2021-h400-ml-15-3-4/10
7.51
7.26
4.88
4.85
4.83
4.26
4.25
4.24
4.24
4.23
4.23
4.22
4.21
4.21
4.20
4.20
4.19
4.19
4.18
4.17
4.17
4.16
3.65
3.62
3.62
3.59
3.56
1.35
1.33
1.32
1.31
1.30
1.29
Br
N
O
P
O
EtO
OEt
4ea
3.99
1.00
3.99
1.99
6.01
f1 (ppm)

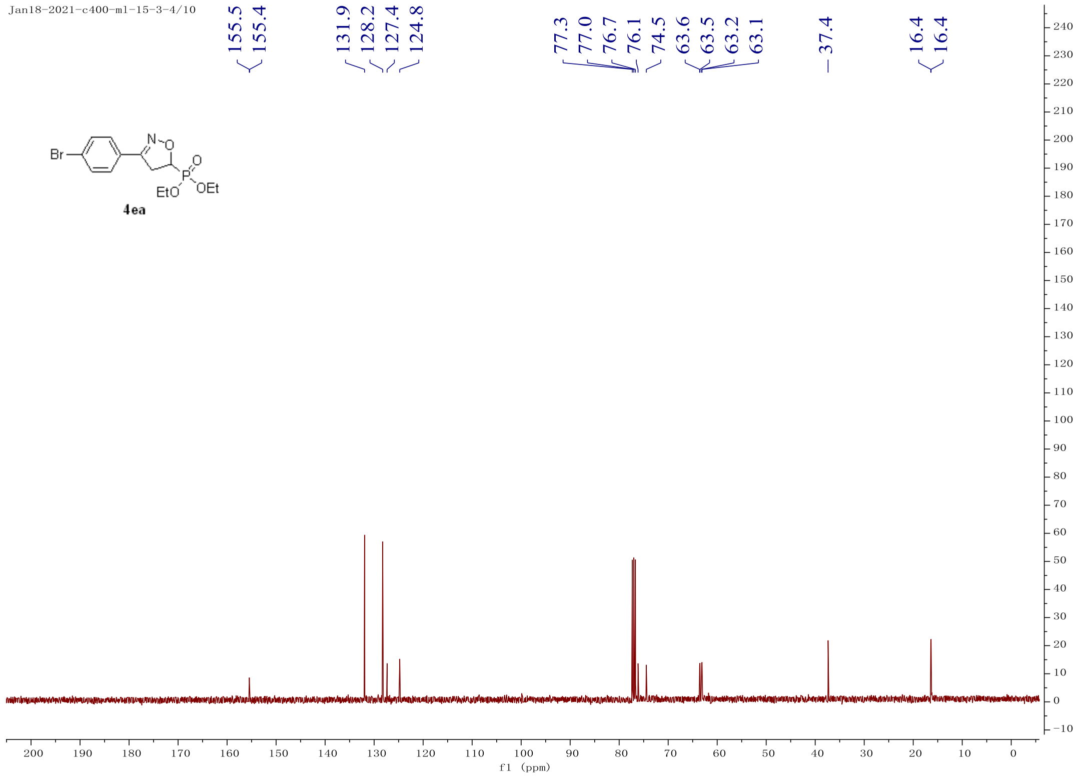
Jan18-2021-c400-ml-15-3-4/10
155.5
155.4
131.9
128.2
127.4
124.8
77.3
77.0
76.7
76.1
74.5
63.6
63.5
63.2
63.1
37.4
16.4
16.4
Br
N
O
P
O
EtO
OEt
4ea
f1 (ppm)

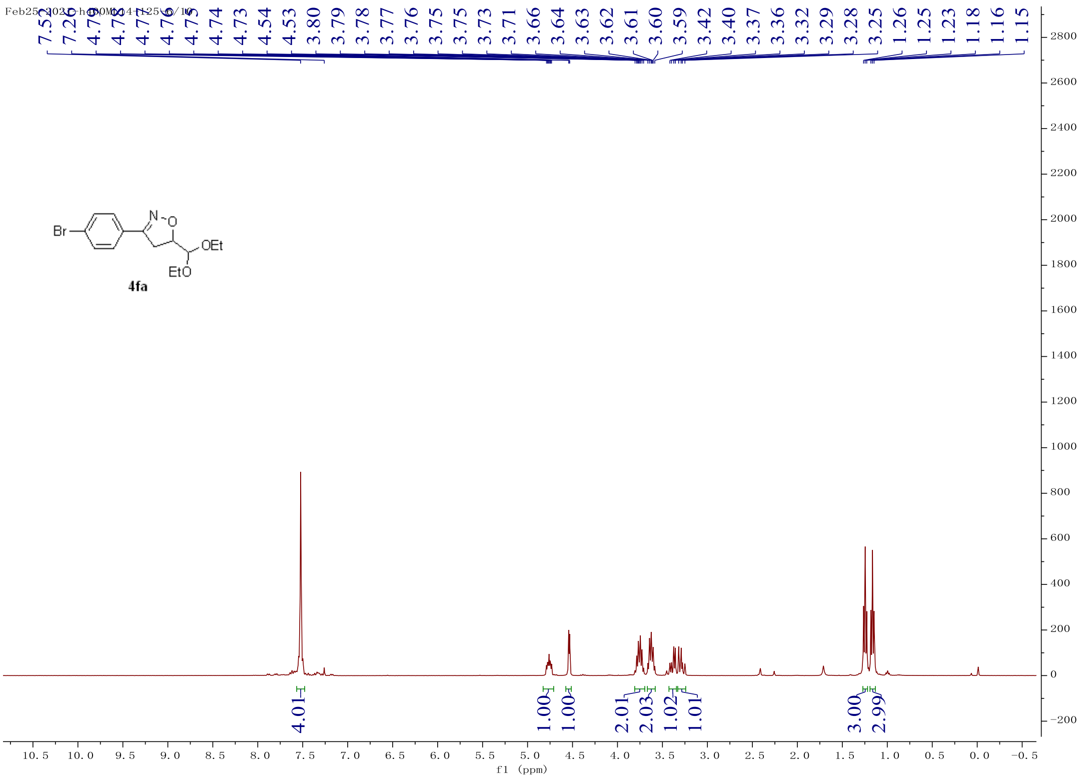
Feb25-2021-[illegible]-125-6/10
7.52 7.26 4.79 4.78 4.77 4.76 4.75 4.74 4.73 4.54 4.53 3.80 3.79 3.78 3.77 3.76 3.75 3.75 3.73 3.71 3.66 3.64 3.63 3.62 3.61 3.60 3.59 3.42 3.40 3.37 3.36 3.32 3.29 3.28 3.25 1.26 1.25 1.23 1.18 1.16 1.15
Br
N
O
OEt
EtO
4fa
4.01
1.00
1.00
2.01
2.03
1.02
1.01
3.00
2.99
f1 (ppm)

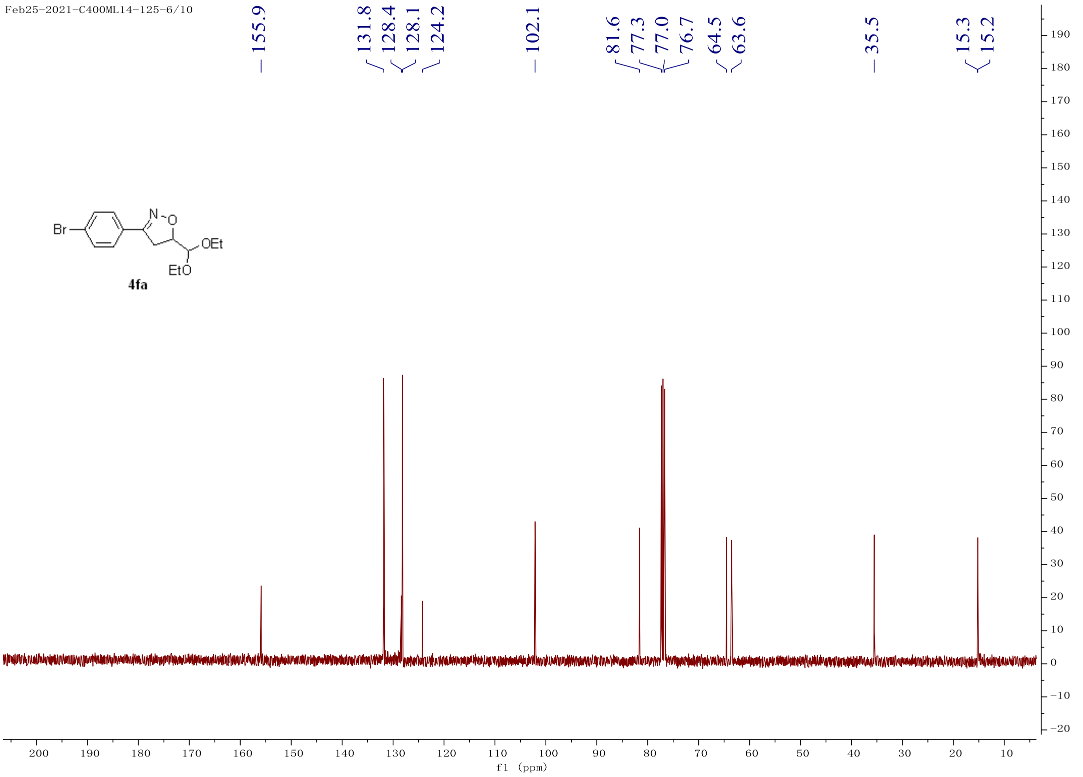
Feb25-2021-C400ML14-125-6/10
155.9
131.8
128.4
128.1
124.2
102.1
81.6
77.3
77.0
76.7
64.5
63.6
35.5
15.3
15.2
Br
N
O
OEt
EtO
4fa
f1 (ppm)

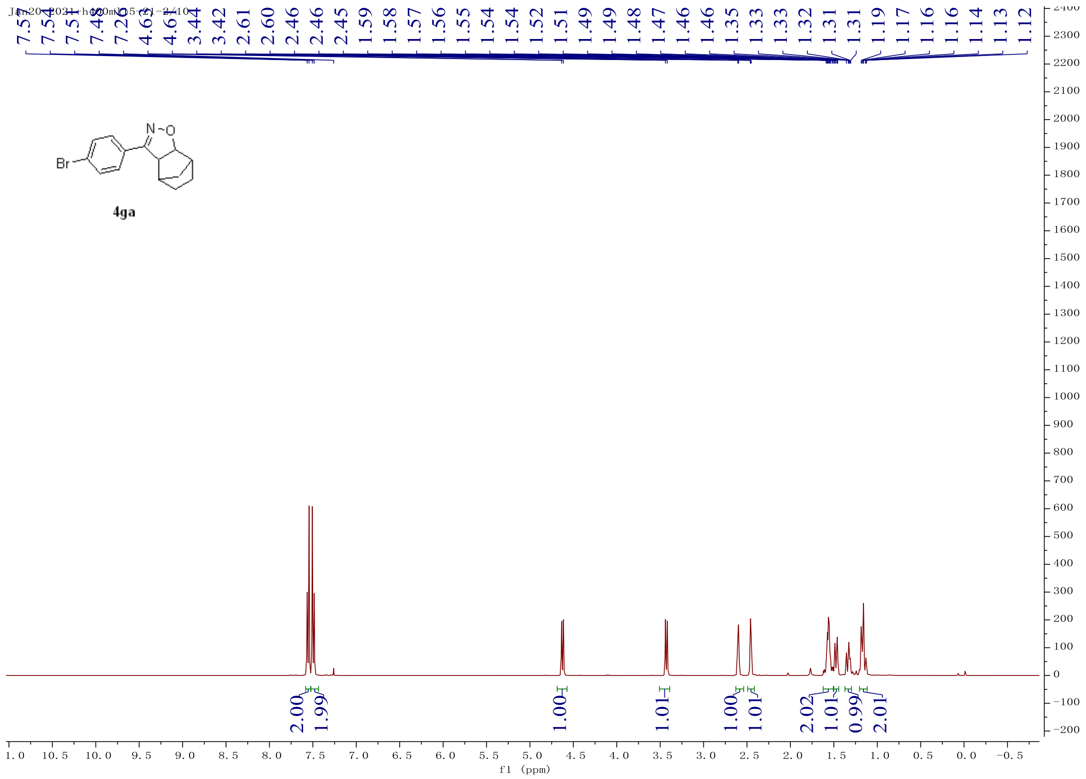
Jan20-2021-h400ml15-21-2/10
Br
N
O
4ga
2.00
1.99
1.00
1.01
1.00
1.01
2.02
1.01
0.99
2.01
f1 (ppm)

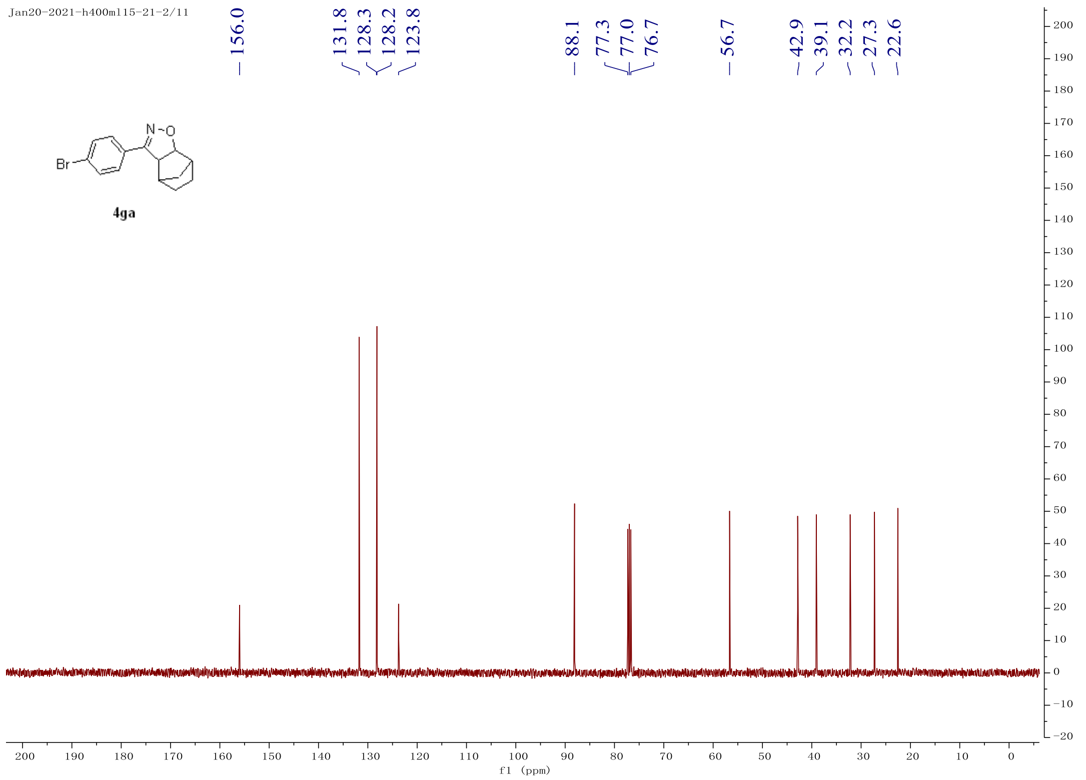
Jan20-2021-h400ml15-21-2/11
156.0
131.8
128.3
128.2
123.8
88.1
77.3
77.0
76.7
56.7
42.9
39.1
32.2
27.3
22.6
Br
N
O
4ga
f1 (ppm)

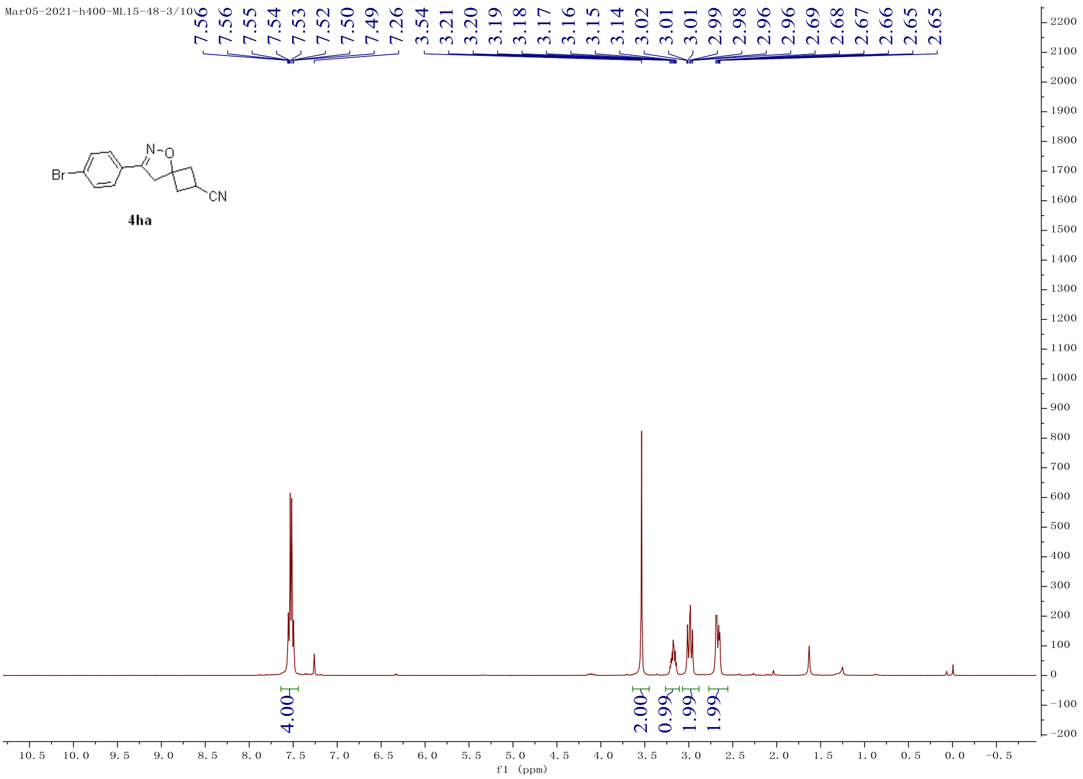

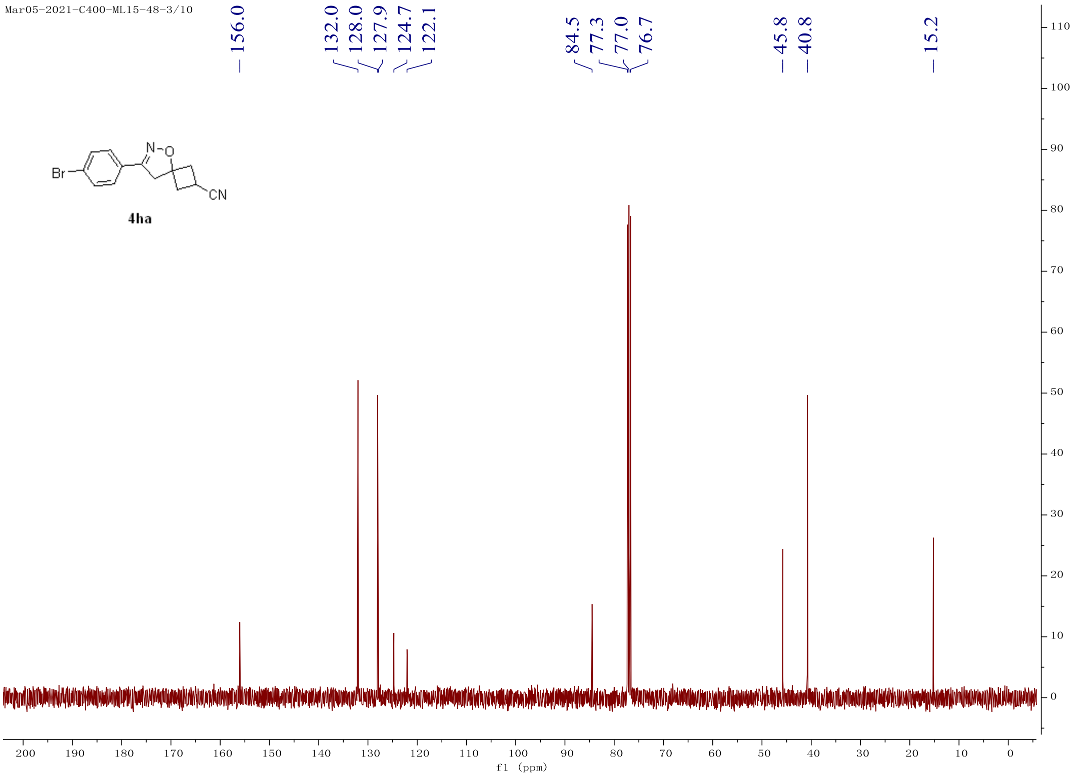

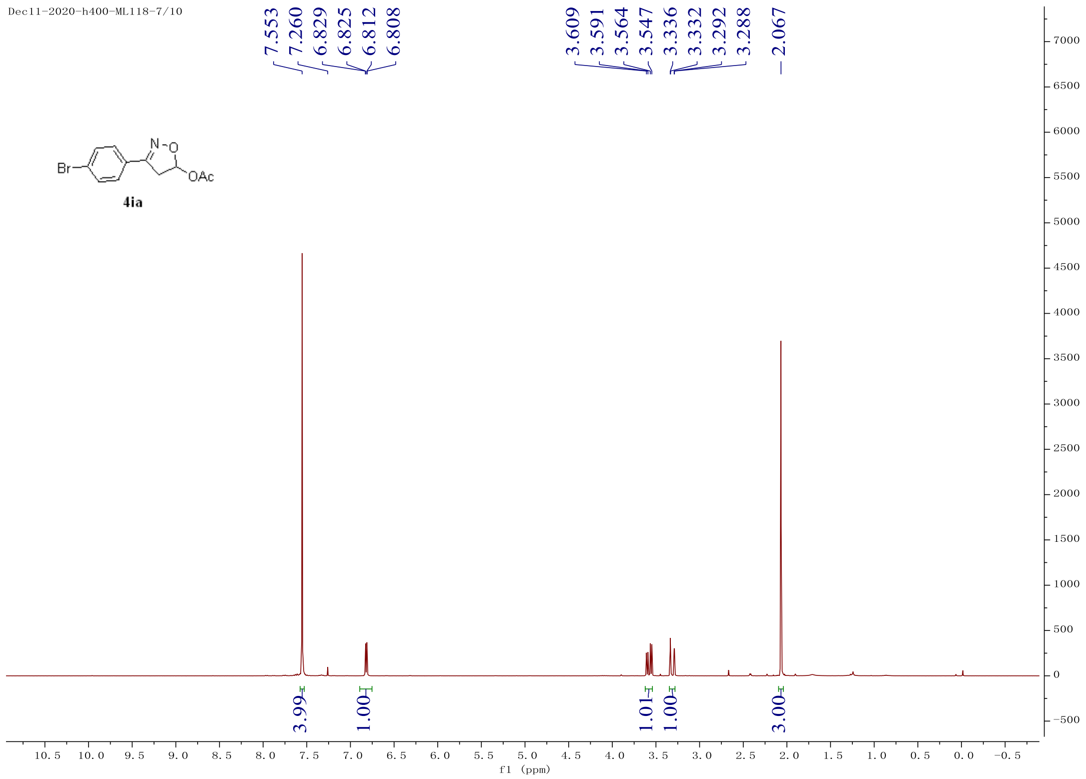
Dec11-2020-h400-ML118-7/10
7.553
7.260
6.829
6.825
6.812
6.808
3.609
3.591
3.564
3.547
3.336
3.332
3.292
3.288
2.067
Br
N
O
OAc
4ia
3.99
1.00
1.01
1.00
3.00
f1 (ppm)

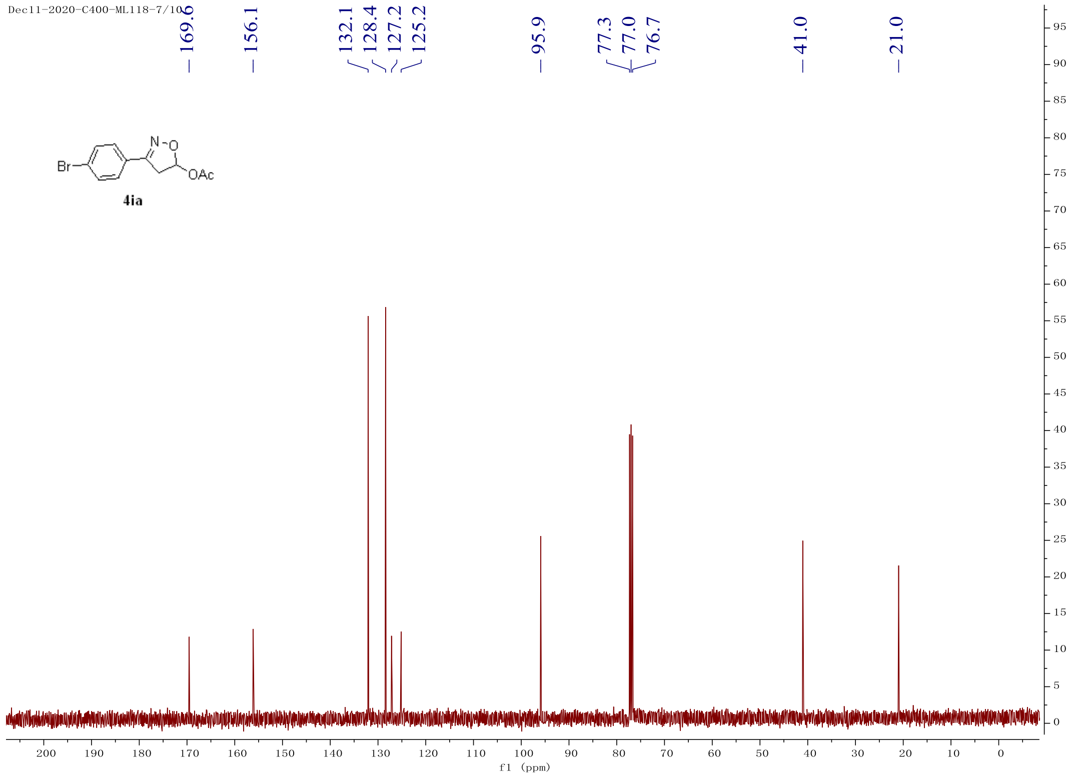
Dec11-2020-C400-ML118-7/10
169.6
156.1
132.1
128.4
127.2
125.2
95.9
77.3
77.0
76.7
41.0
21.0
Br
N
O
OAc
4ia
f1 (ppm)

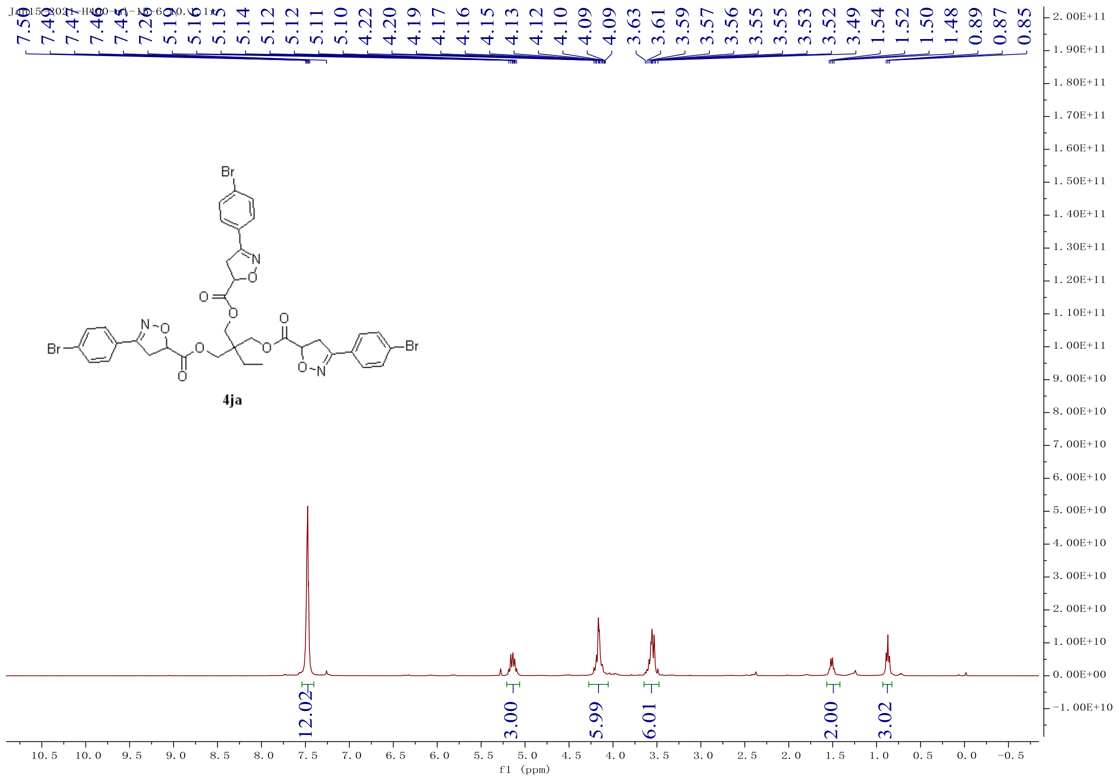
7.50 7.49 7.47 7.46 7.45 7.26 5.17 5.16 5.15 5.14 5.12 5.12 5.11 5.10 4.22 4.20 4.19 4.17 4.16 4.15 4.13 4.12 4.10 4.09 4.09 3.63 3.61 3.59 3.57 3.56 3.55 3.55 3.53 3.52 3.49 1.54 1.52 1.50 1.48 0.89 0.87 0.85
Br
4ja
12.02
3.00
5.99
6.01
2.00
3.02
f1 (ppm)

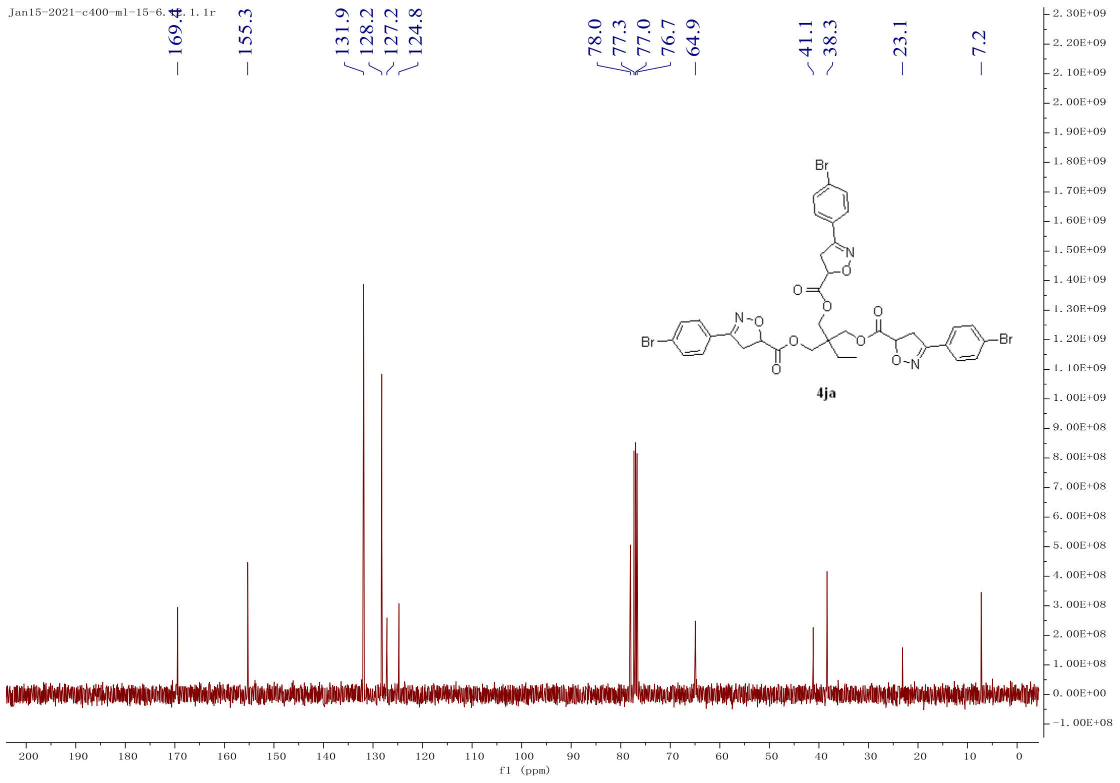
Jan15-2021-c400-ml-15-6.11.1.1r
169.4
155.3
131.9 128.2 127.2 124.8
78.0 77.3 77.0 76.7 64.9
41.1 38.3
23.1
7.2
Br
4ja
f1 (ppm)

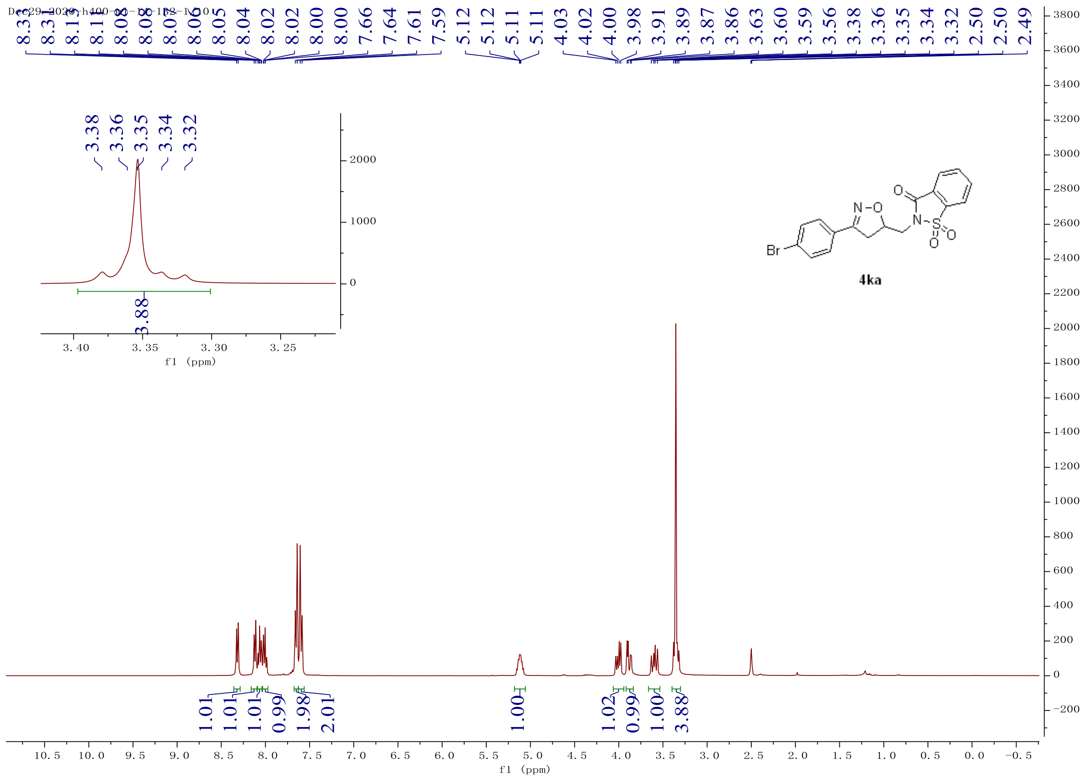
8.05
8.04
8.02
8.02
8.00
8.00
7.66
7.64
7.61
7.59
5.12
5.12
5.11
5.11
4.03
4.02
4.00
3.98
3.91
3.89
3.87
3.86
3.63
3.60
3.59
3.56
3.38
3.36
3.35
3.34
3.32
2.50
2.50
2.49
Br
N–O
O
N
S
O
O
4ka
1.01
1.01
1.01
0.99
1.98
2.01
1.00
1.02
0.99
1.00
3.88
f1 (ppm)

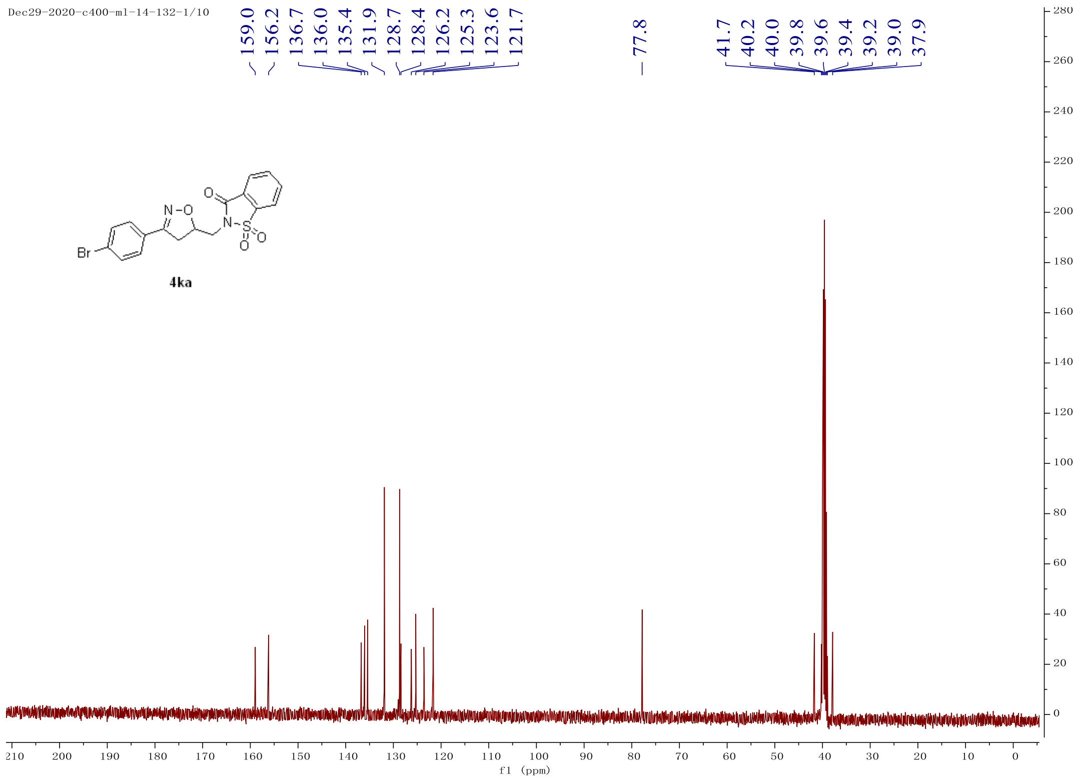
Dec29-2020-c400-ml-14-132-1/10
159.0
156.2
136.7
136.0
135.4
131.9
128.7
128.4
126.2
125.3
123.6
121.7
77.8
41.7
40.2
40.0
39.8
39.6
39.4
39.2
39.0
37.9
Br
N–O
O
N
S
O
O
4ka
f1 (ppm)

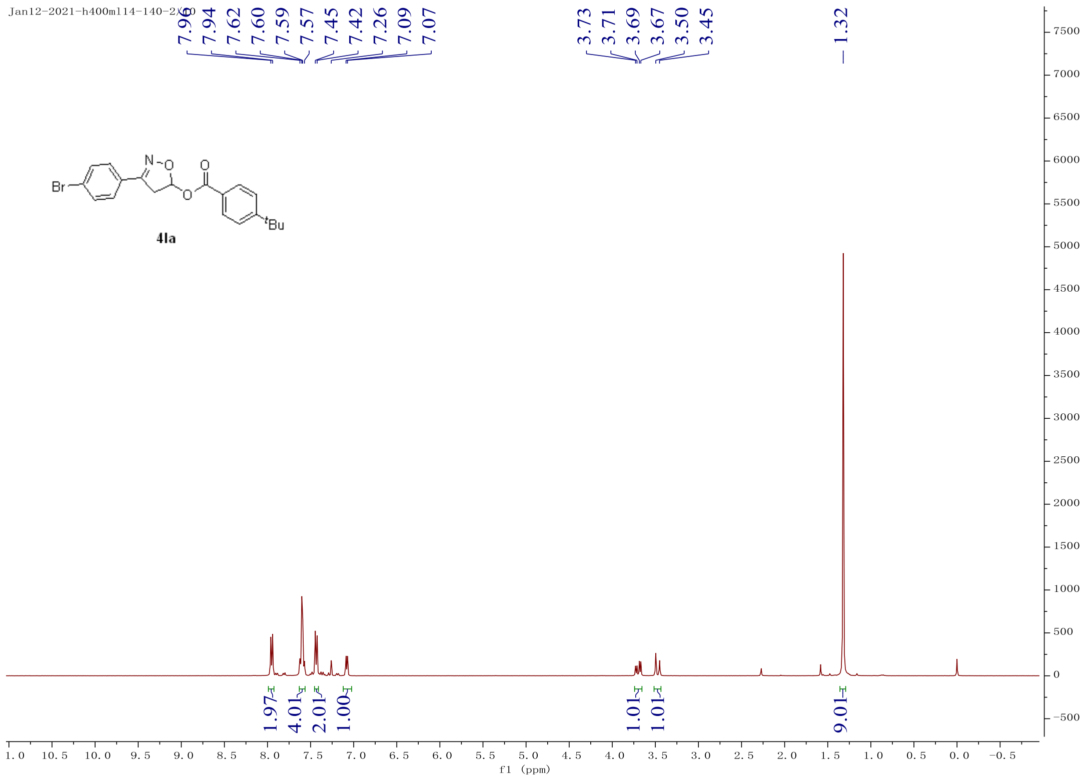
Jan12-2021-h400ml14-140-2/10
7.96
7.94
7.62
7.60
7.59
7.57
7.45
7.42
7.26
7.09
7.07
3.73
3.71
3.69
3.67
3.50
3.45
1.32
Br
N
O
O
O
tBu
4la
1.97
4.01
2.01
1.00
1.01
1.01
9.01
f1 (ppm)

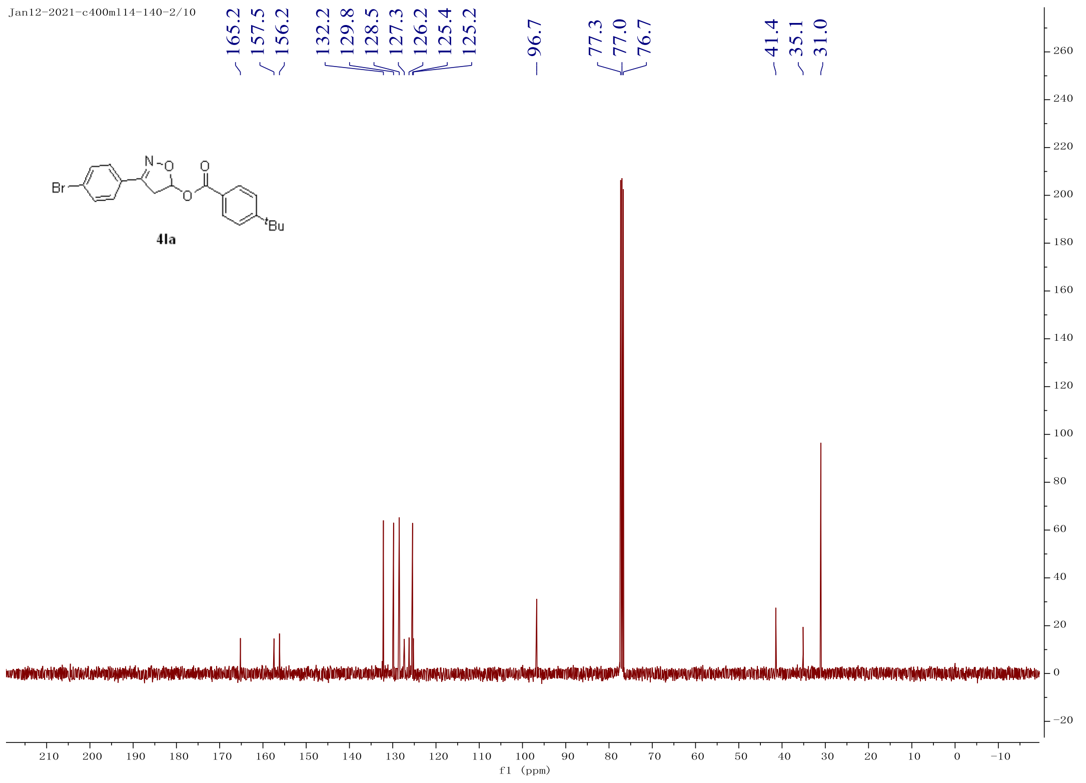
Jan12-2021-c400ml14-140-2/10
165.2
157.5
156.2
132.2
129.8
128.5
127.3
126.2
125.4
125.2
96.7
77.3
77.0
76.7
41.4
35.1
31.0
Br
N
O
O
O
tBu
4la
f1 (ppm)

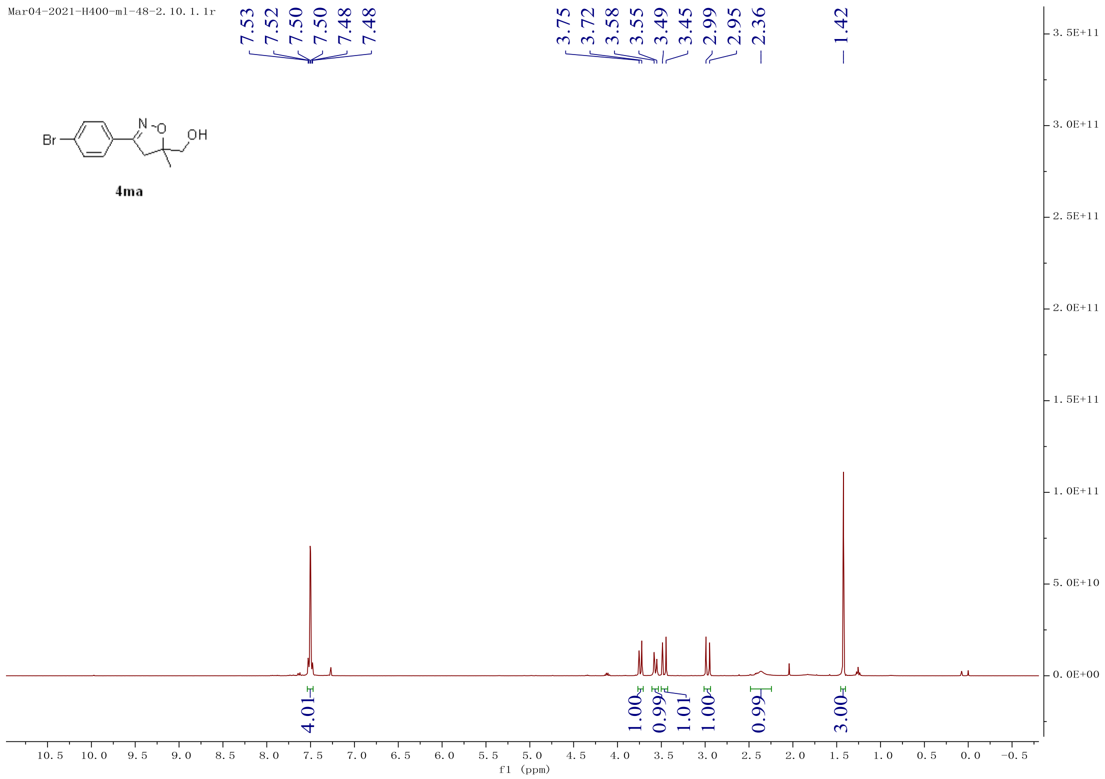
Mar04-2021-H400-ml-48-2.10.1.1r
7.53 7.52 7.50 7.50 7.48 7.48
3.75 3.72 3.58 3.55 3.49 3.45 2.99 2.95 2.36
1.42
Br
N
O
OH
4ma
4.01
1.00 0.99 1.01 1.00 0.99
3.00
f1 (ppm)

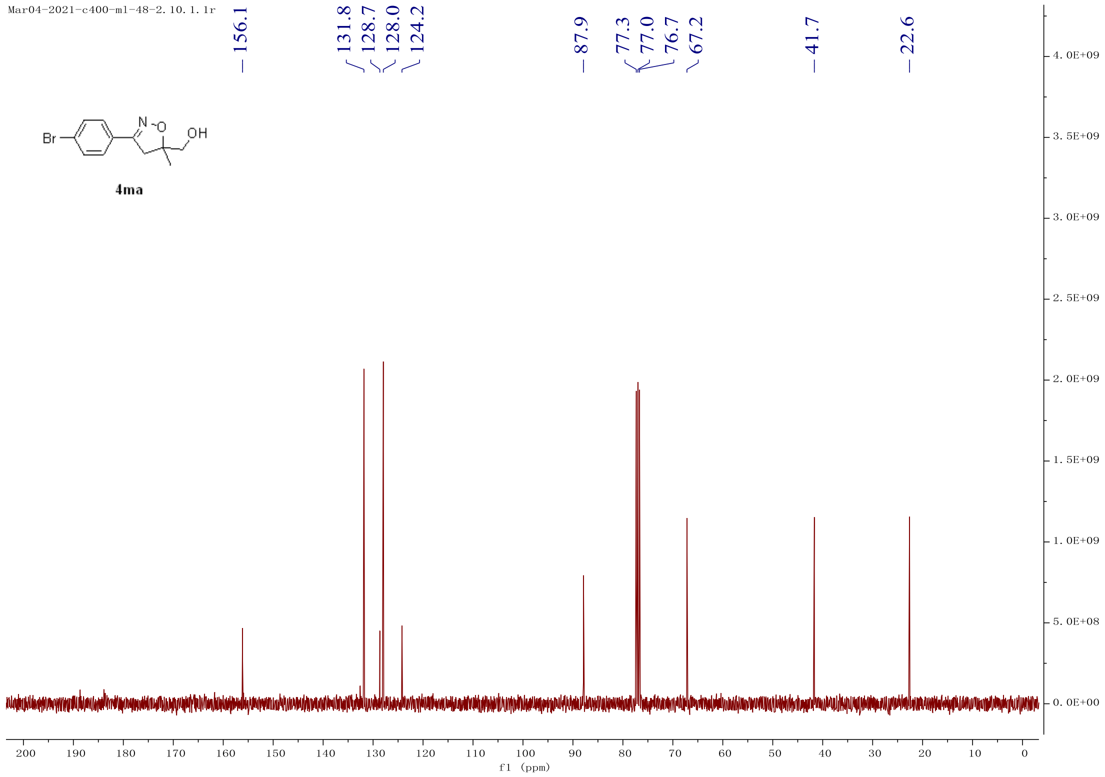
Mar04-2021-c400-ml-48-2.10.1.1r
156.1
131.8 128.7 128.0 124.2
87.9
77.3 77.0 76.7 67.2
41.7
22.6
Br
N
O
OH
4ma
f1 (ppm)

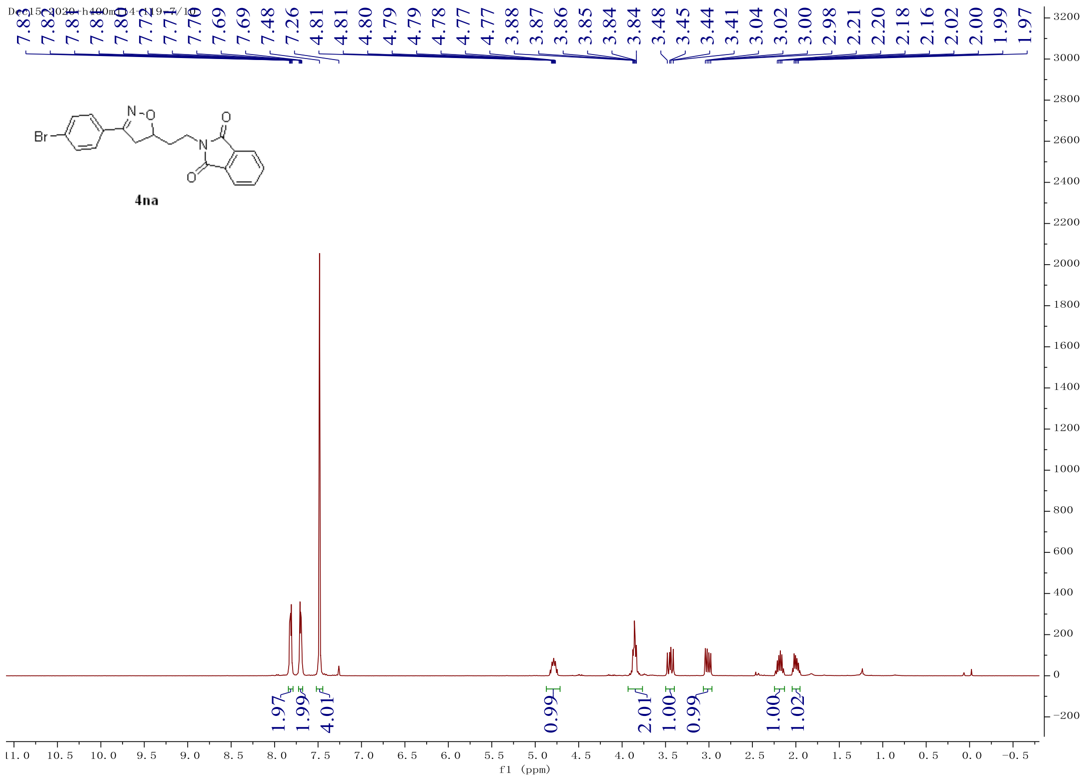

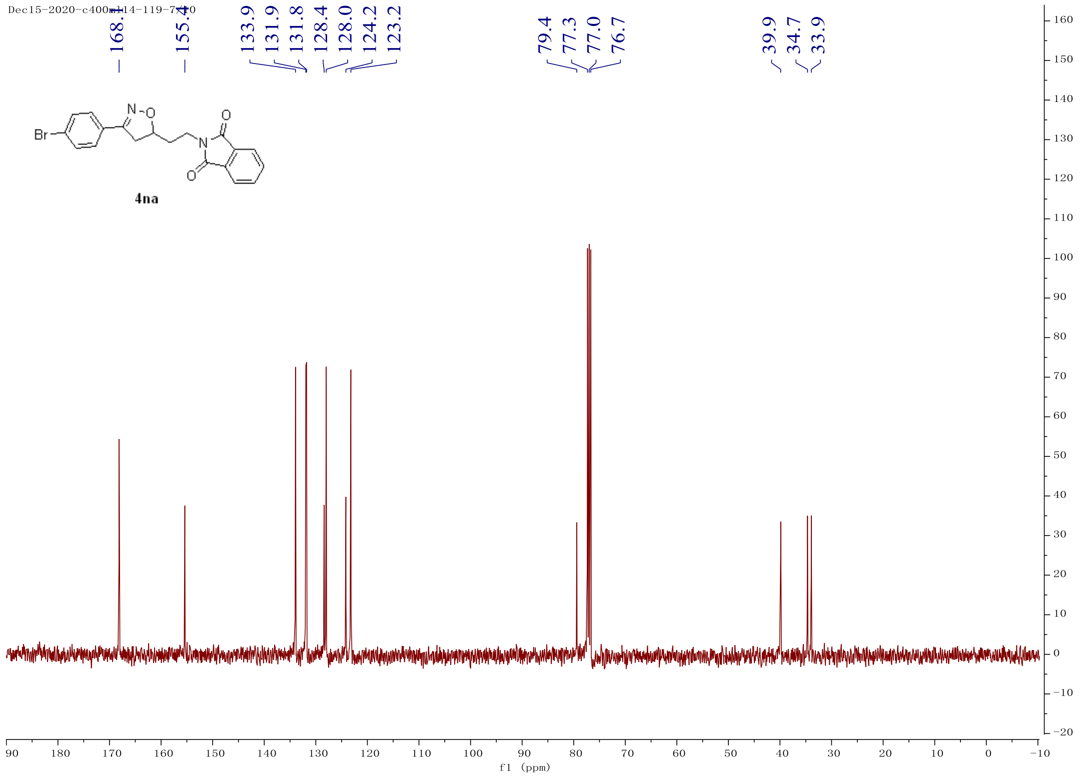

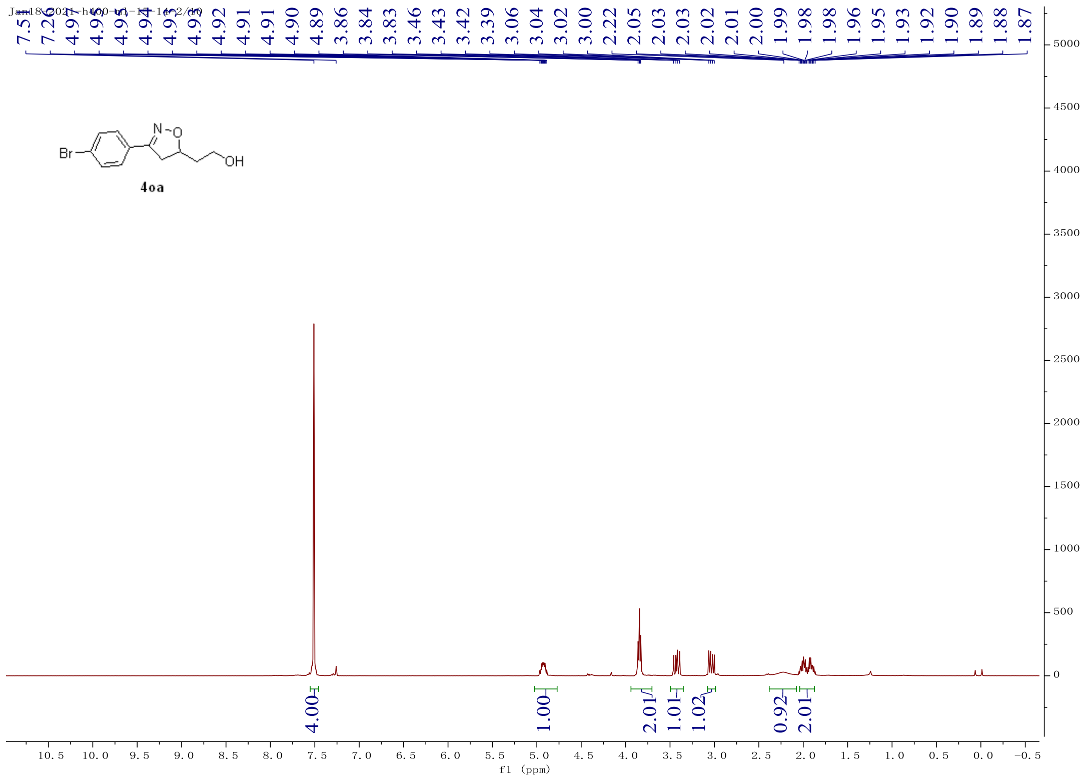
7.51
7.26
4.97
4.95
4.94
4.93
4.93
4.92
4.91
4.91
4.90
4.89
3.86
3.84
3.83
3.46
3.43
3.42
3.39
3.06
3.04
3.02
3.00
2.22
2.05
2.03
2.03
2.02
2.01
2.00
1.99
1.98
1.98
1.96
1.95
1.93
1.92
1.90
1.89
1.88
1.87
Br
N
O
OH
4oa
4.00
1.00
2.01
1.01
1.02
0.92
2.01
f1 (ppm)

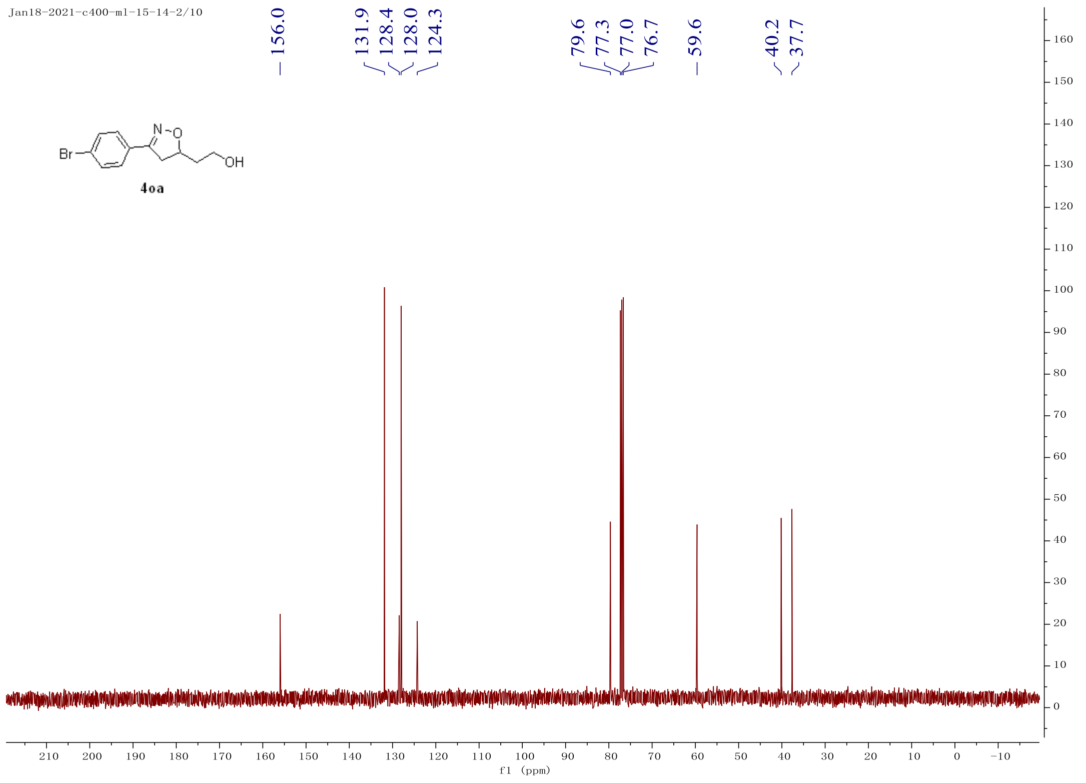
Jan18-2021-c400-ml-15-14-2/10
156.0
131.9
128.4
128.0
124.3
79.6
77.3
77.0
76.7
59.6
40.2
37.7
Br
N
O
OH
4oa
f1 (ppm)

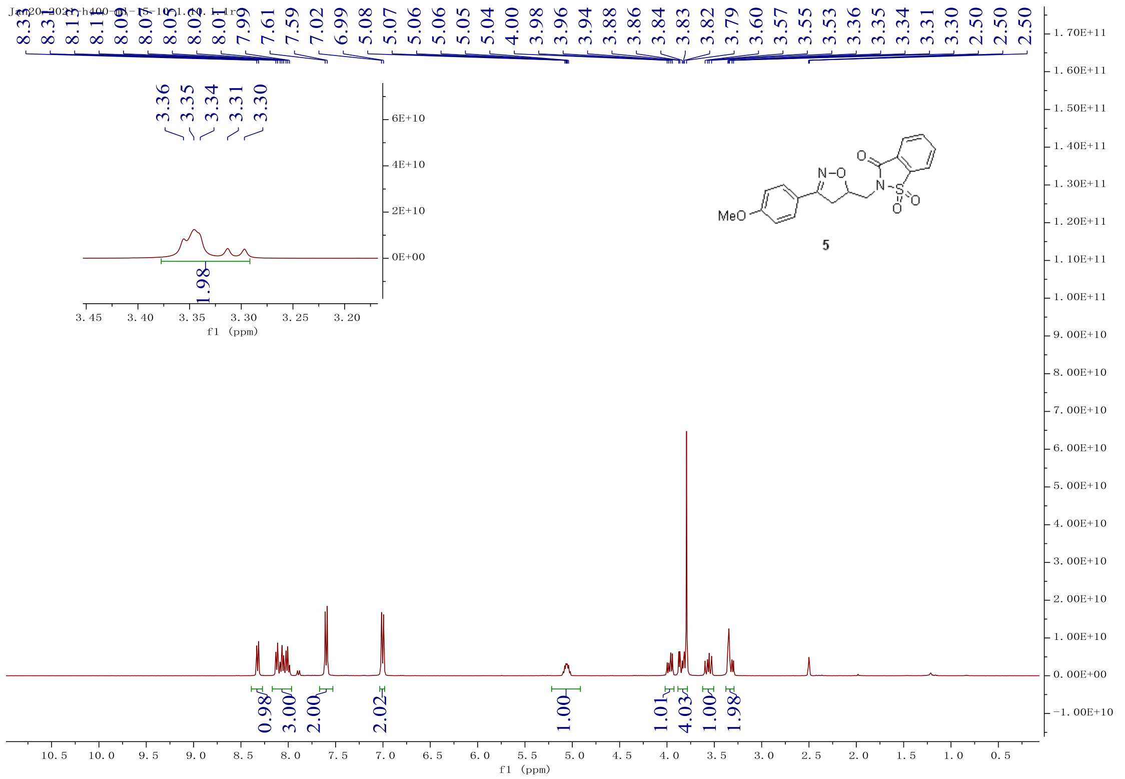
MeO
N–O
N
O
S
5

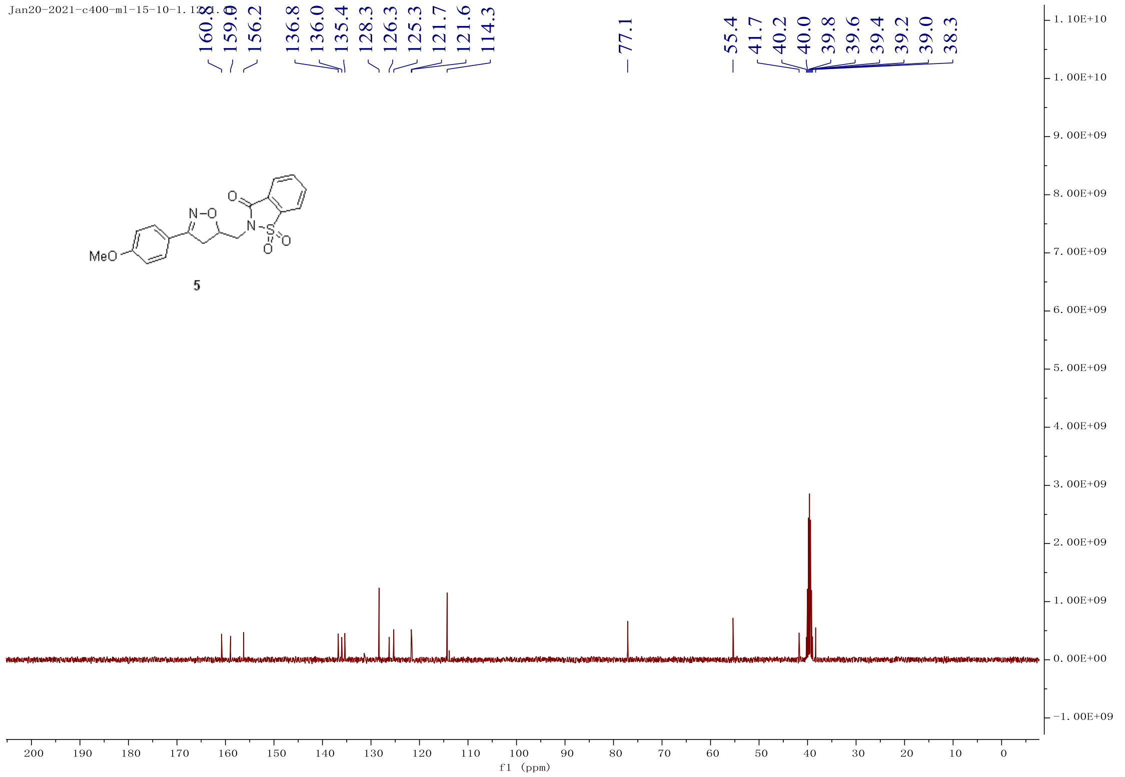
MeO
N–O
N
O
S
5

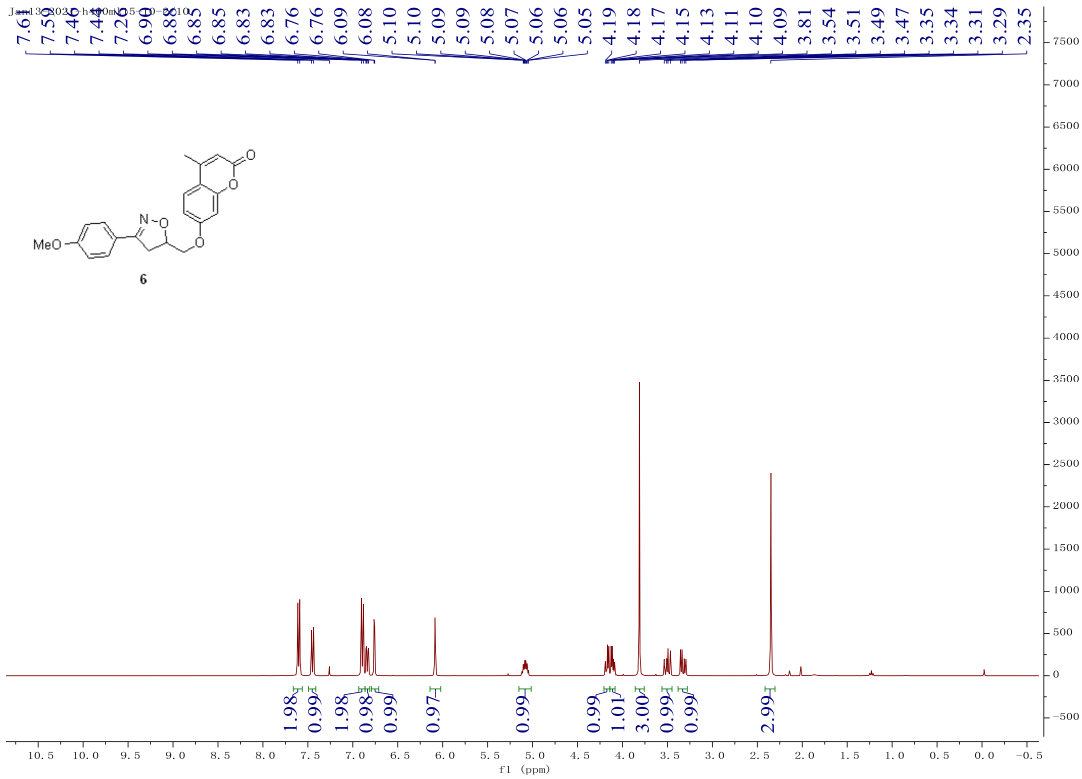
Jan13-2021-h400m115-10-2.10
7.61 7.59 7.46 7.44 7.26 6.90 6.88 6.85 6.85 6.83 6.83 6.76 6.76 6.09 6.08 5.10 5.10 5.09 5.09 5.08 5.07 5.06 5.06 5.05 4.19 4.18 4.17 4.15 4.13 4.11 4.10 4.09 3.81 3.54 3.51 3.49 3.47 3.35 3.34 3.31 3.29 2.35
MeO
N–O
O
O
O
6
1.98 0.99 1.98 0.98 0.99 0.97 0.99 0.99 1.01 3.00 0.99 0.99 2.99
f1 (ppm)

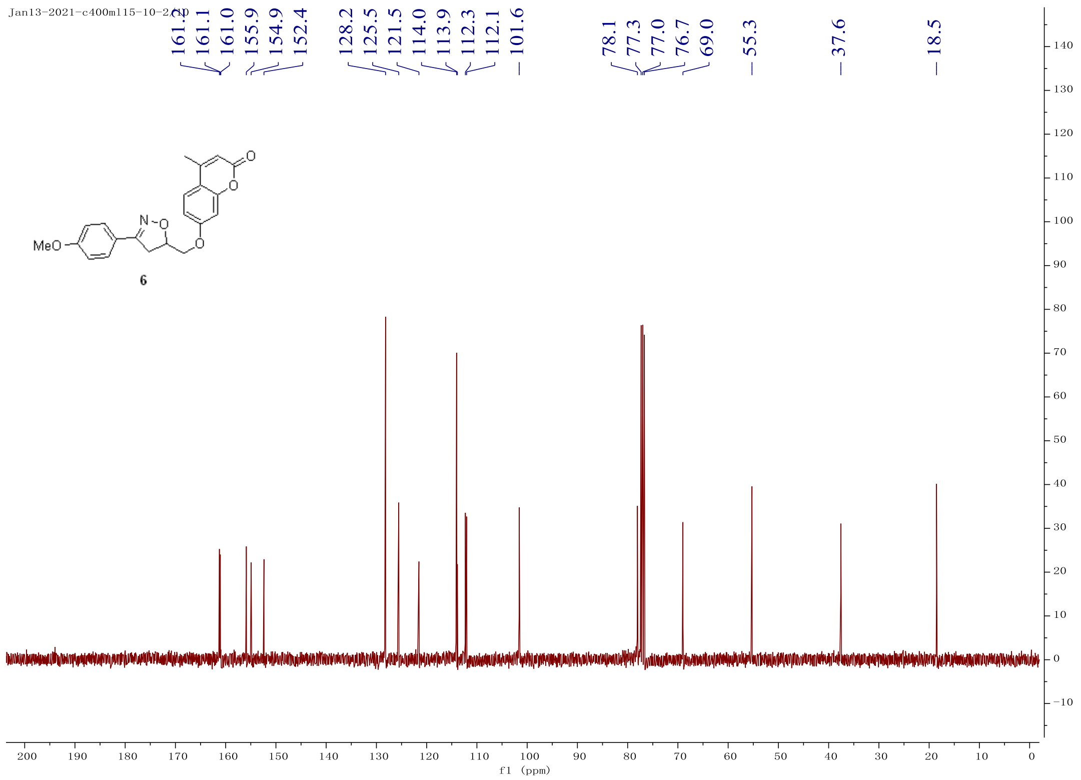
Jan13-2021-c400m115-10-2.10
161.2 161.1 161.0 155.9 154.9 152.4 128.2 125.5 121.5 114.0 113.9 112.3 112.1 101.6 78.1 77.3 77.0 76.7 69.0 55.3 37.6 18.5
MeO
N–O
O
O
O
6
f1 (ppm)

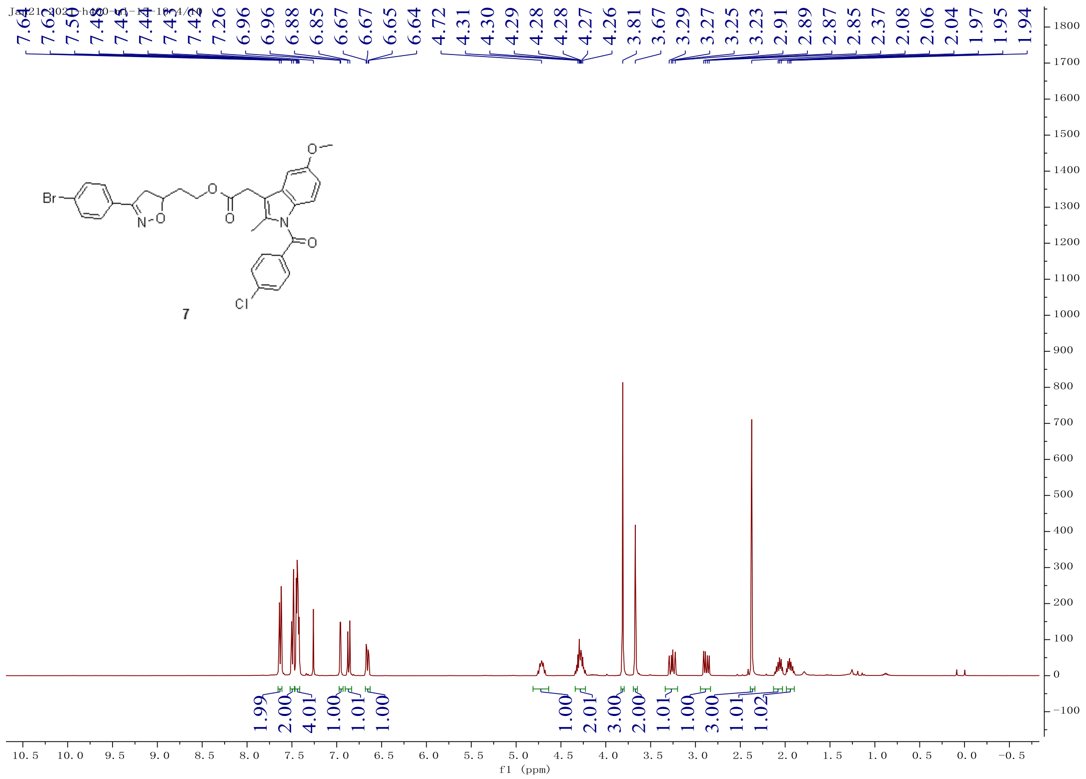
7.64 7.62 7.50 7.48 7.45 7.44 7.43 7.42 7.26 6.96 6.96 6.88 6.85 6.67 6.67 6.65 6.64 4.72 4.31 4.30 4.29 4.28 4.28 4.27 4.26 3.81 3.67 3.29 3.27 3.25 3.23 2.91 2.89 2.87 2.85 2.37 2.08 2.06 2.04 1.97 1.95 1.94
Br
N-O
O
O
O
N
O
Cl
7
1.99 2.00 4.01 1.00 1.01 1.00 1.00 2.01 3.00 2.00 1.01 1.00 3.00 1.01 1.02
f1 (ppm)

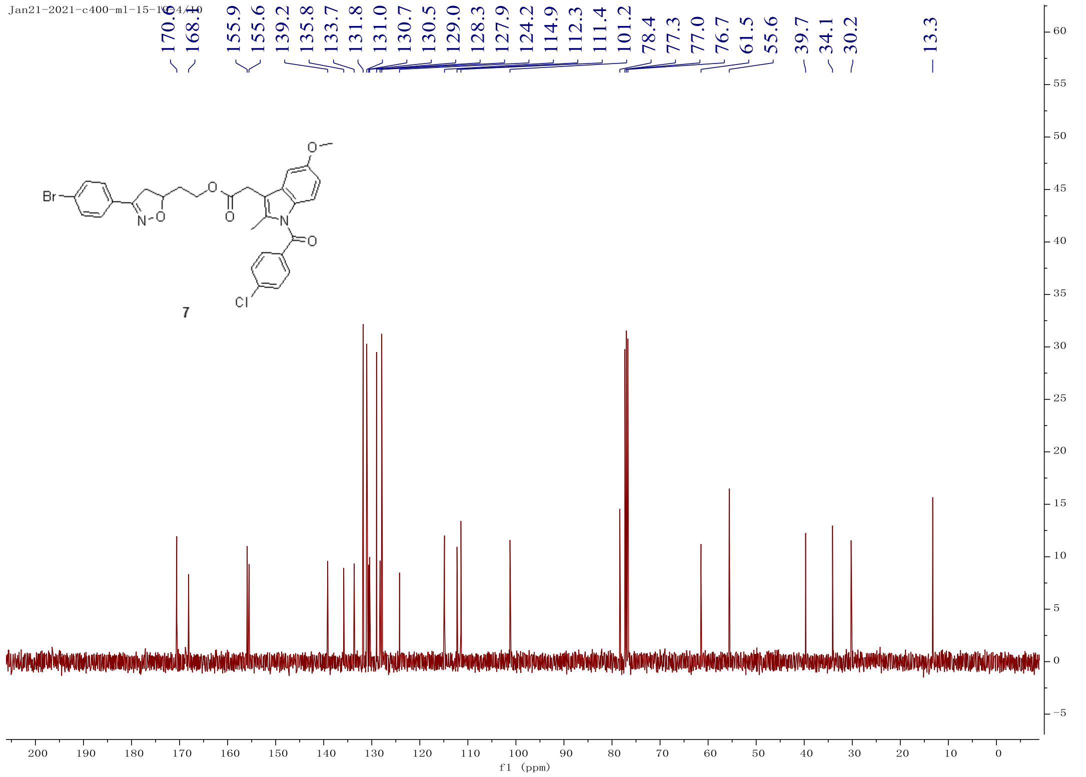
Jan21-2021-c400-ml-15-1064/10
170.6 168.1 155.9 155.6 139.2 135.8 133.7 131.8 131.0 130.7 130.5 129.0 128.3 127.9 124.2 114.9 112.3 111.4 101.2 78.4 77.3 77.0 76.7 61.5 55.6 39.7 34.1 30.2 13.3
Br
N-O
O
O
O
N
O
Cl
7
f1 (ppm)

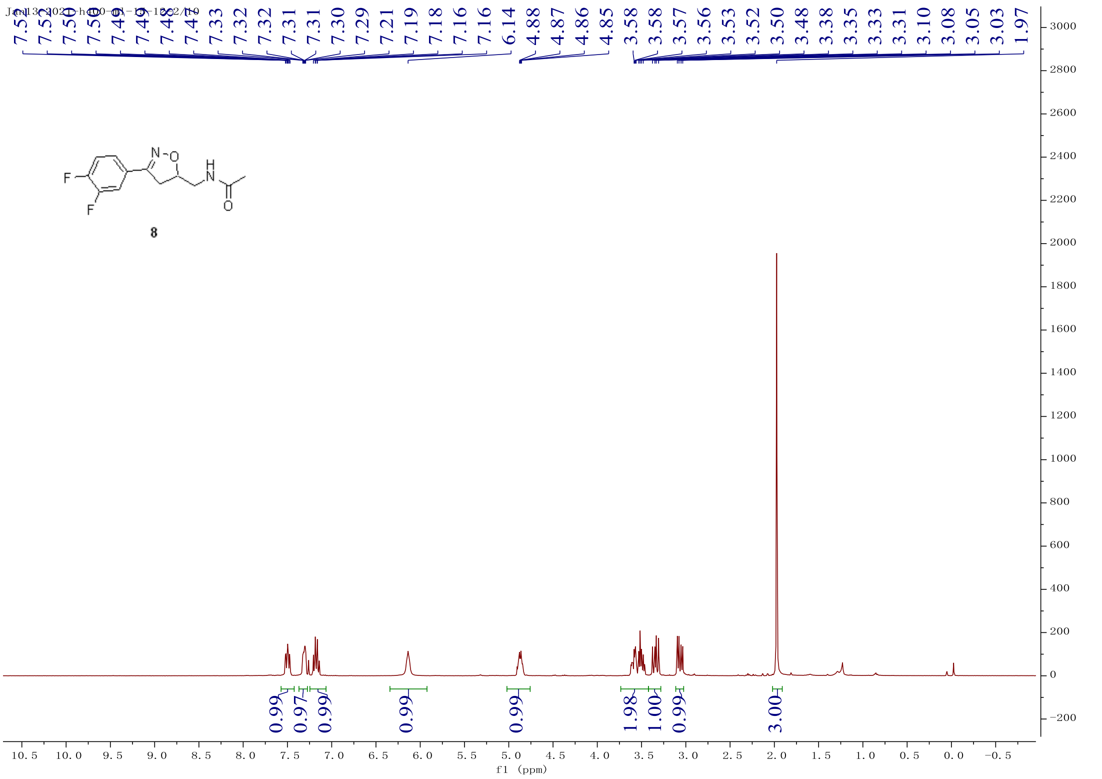
7.52
7.52
7.50
7.50
7.49
7.49
7.48
7.48
7.33
7.32
7.32
7.31
7.31
7.30
7.29
7.21
7.19
7.18
7.16
7.16
6.14
4.88
4.87
4.86
4.85
3.58
3.58
3.57
3.56
3.53
3.52
3.50
3.48
3.38
3.35
3.33
3.31
3.10
3.08
3.05
3.03
1.97
F
F
N-O
H
N
O
8
0.99
0.97
0.99
0.99
0.99
1.98
1.00
0.99
3.00
f1 (ppm)

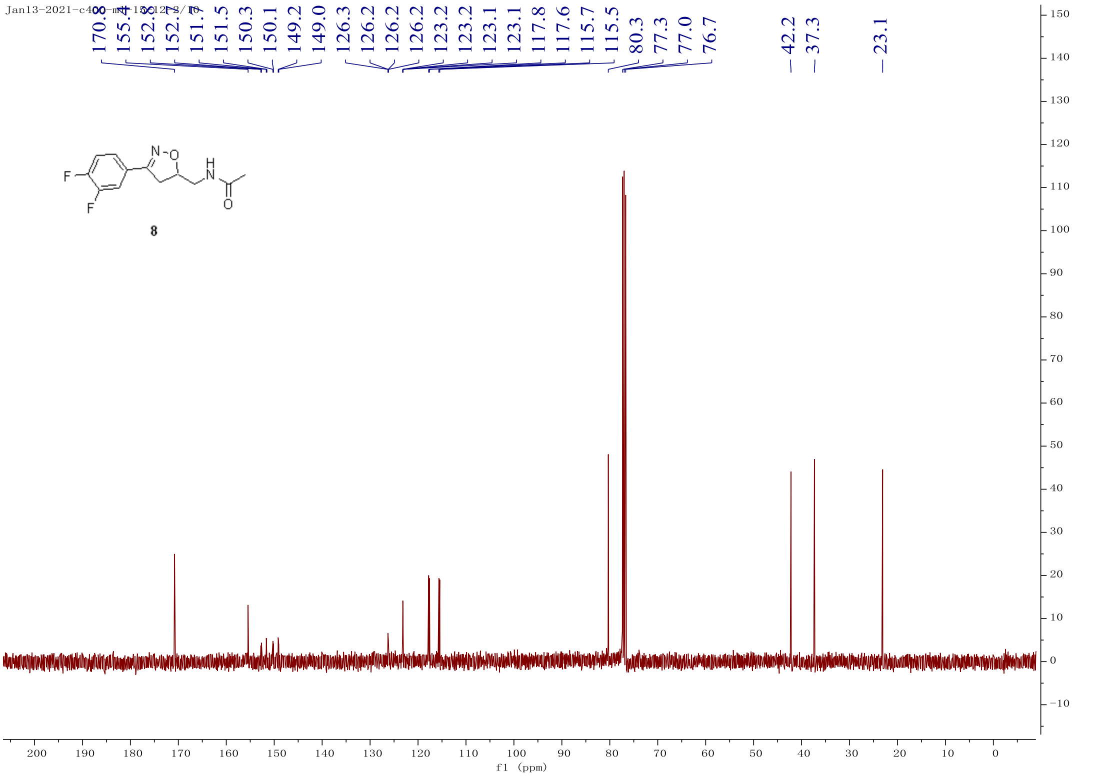
170.8
155.4
152.8
152.7
151.7
151.5
150.3
150.1
149.2
149.0
126.3
126.2
126.2
126.2
123.2
123.2
123.1
123.1
117.8
117.6
115.7
115.5
80.3
77.3
77.0
76.7
42.2
37.3
23.1
F
F
N-O
H
N
O
8
f1 (ppm)

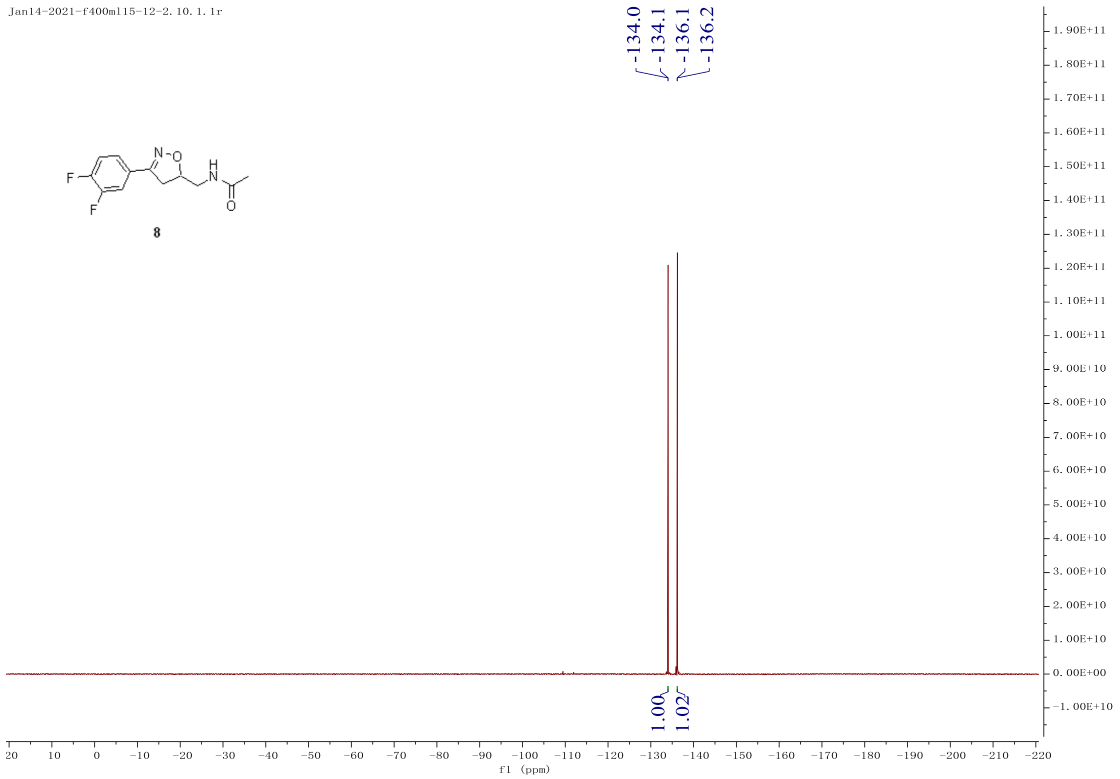
Jan14-2021-f400ml15-12-2.10.1.1r
-134.0
-134.1
-136.1
-136.2
F
F
N-O
H
N
O
8
1.00
1.02
f1 (ppm)

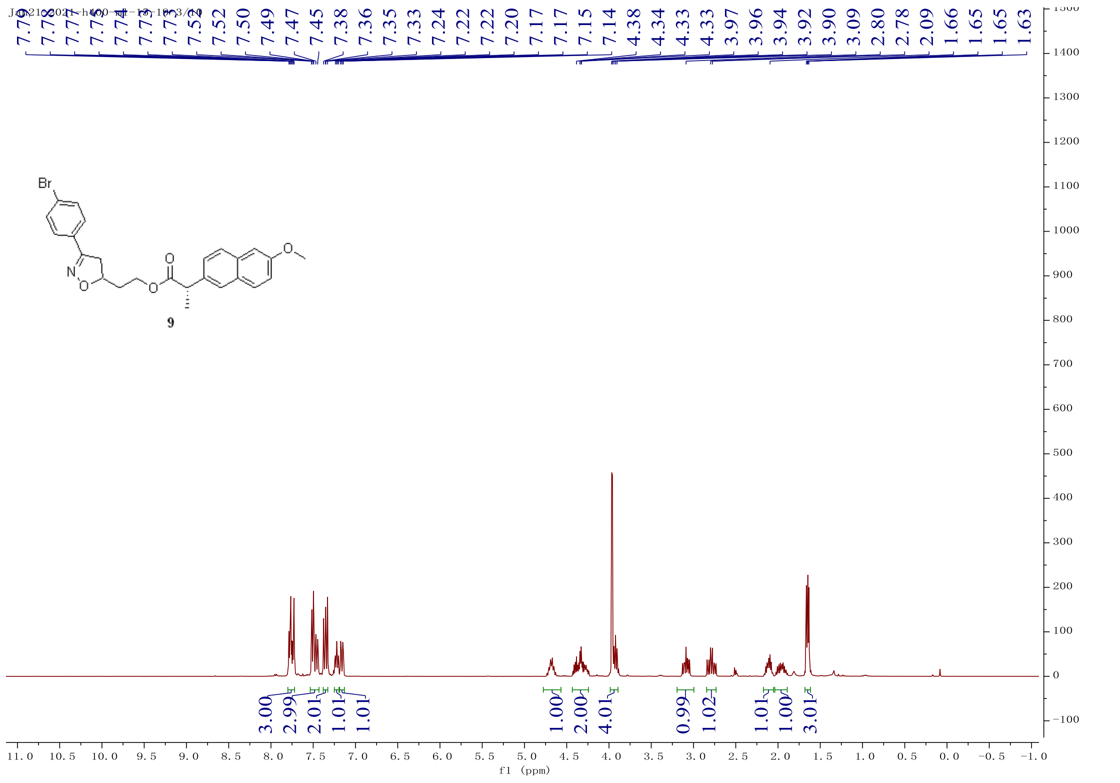
7.79
7.78
7.77
7.75
7.74
7.73
7.73
7.52
7.50
7.49
7.47
7.45
7.38
7.36
7.35
7.33
7.24
7.22
7.22
7.20
7.17
7.17
7.15
7.14
4.38
4.34
4.33
4.33
3.97
3.96
3.94
3.92
3.90
3.09
2.80
2.78
2.09
1.66
1.65
1.65
1.63
Br
N
O
O
O
O
9
3.00
2.99
2.01
1.01
1.01
1.00
2.00
4.01
0.99
1.02
1.01
1.00
3.01
f1 (ppm)

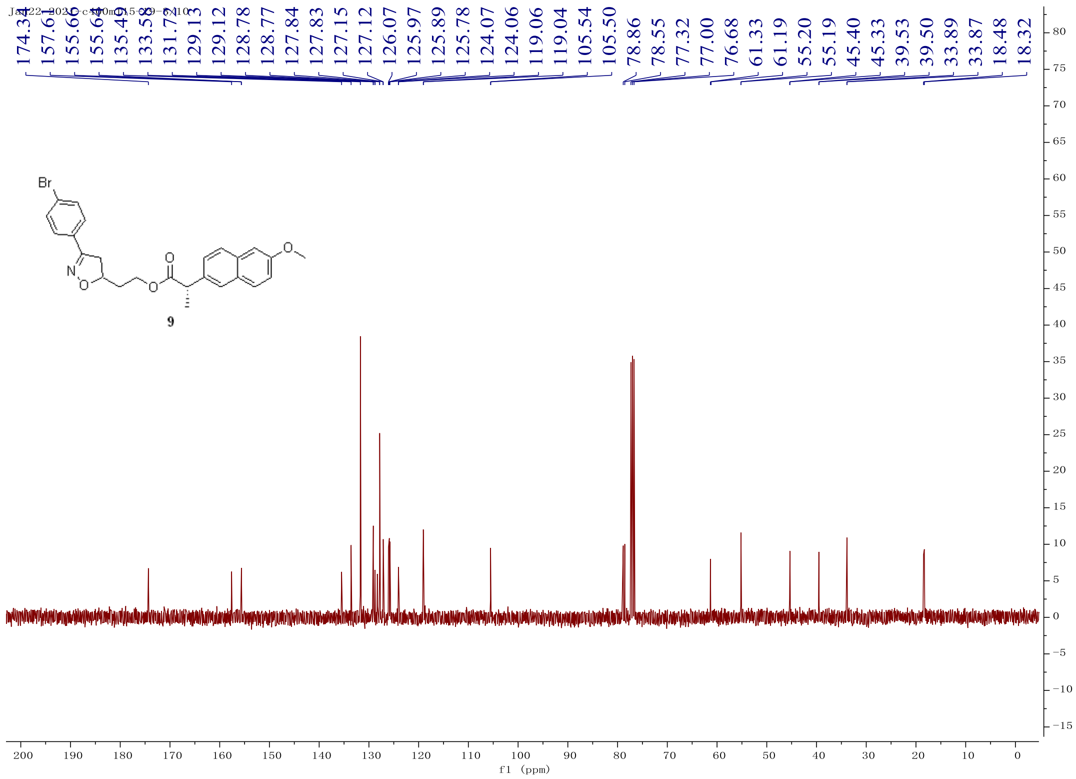
174.34 157.6 155.66 155.64 135.49 133.58 131.72 129.13 129.12 128.78 128.77 127.84 127.83 127.15 127.12 126.07 125.97 125.89 125.78 124.07 124.06 119.06 119.04 105.54 105.50 78.86 78.55 77.32 77.00 76.68 61.33 61.19 55.20 55.19 45.40 45.33 39.53 39.50 33.89 33.87 18.48 18.32
Br
N
O
9
f1 (ppm)

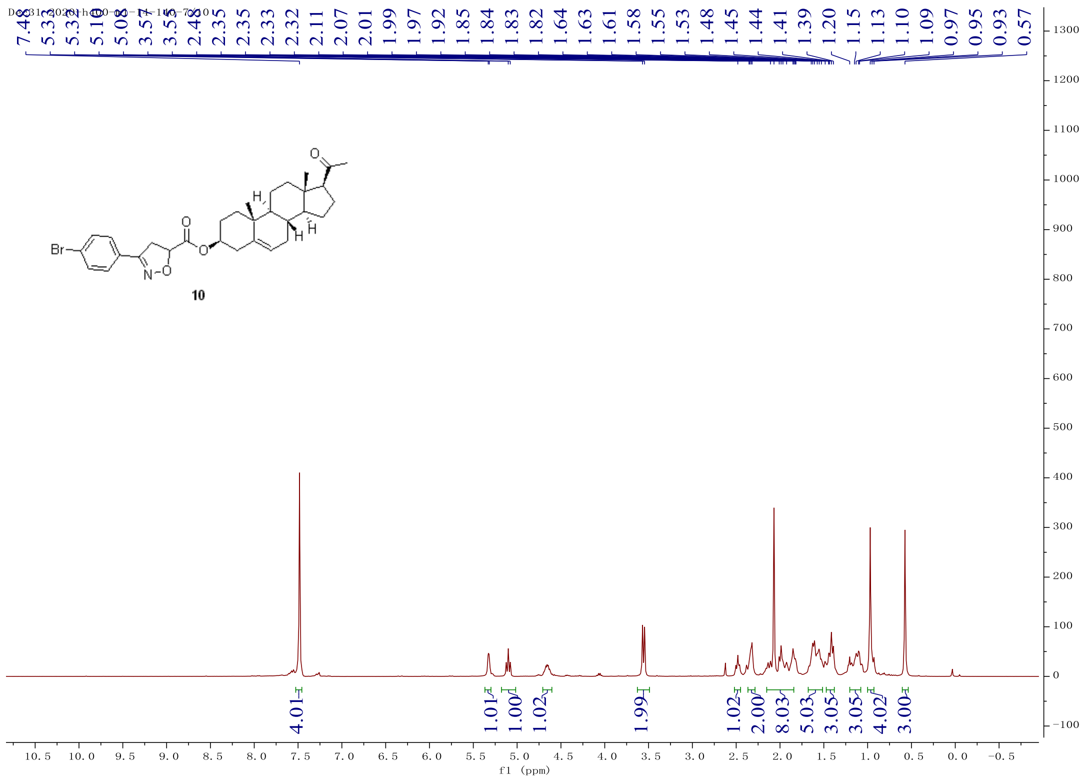
7.48 5.33 5.31 5.10 5.08 3.57 3.55 2.48 2.35 2.35 2.33 2.32 2.11 2.07 2.01 1.99 1.97 1.92 1.85 1.84 1.83 1.82 1.64 1.63 1.61 1.58 1.55 1.53 1.48 1.45 1.44 1.41 1.39 1.20 1.15 1.13 1.10 1.09 0.97 0.95 0.93 0.57
Br
N-O
O
H
10
4.01 1.01 1.00 1.02 1.99 1.02 2.00 8.03 5.03 3.05 3.05 4.02 3.00
f1 (ppm)

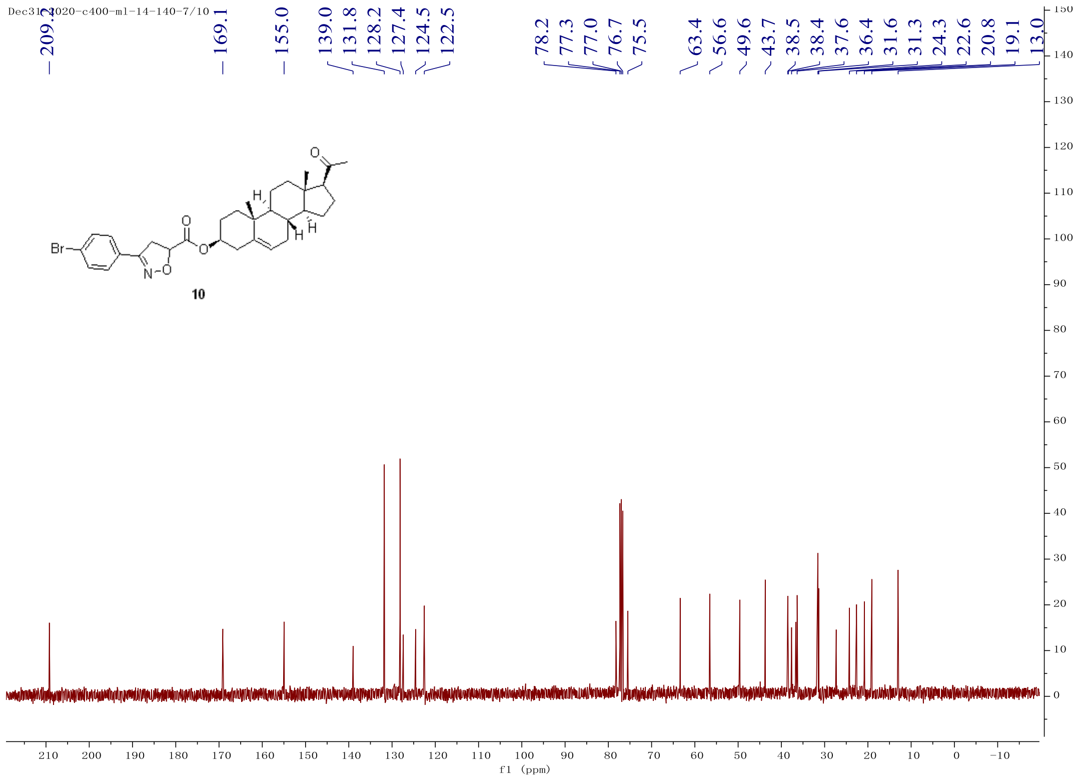
Dec31-2020-c400-ml-14-140-7/10
209.2
169.1
155.0
139.0
131.8
128.2
127.4
124.5
122.5
78.2
77.3
77.0
76.7
75.5
63.4
56.6
49.6
43.7
38.5
38.4
37.6
36.4
31.6
31.3
24.3
22.6
20.8
19.1
13.0
Br
N-O
O
H
10
f1 (ppm)

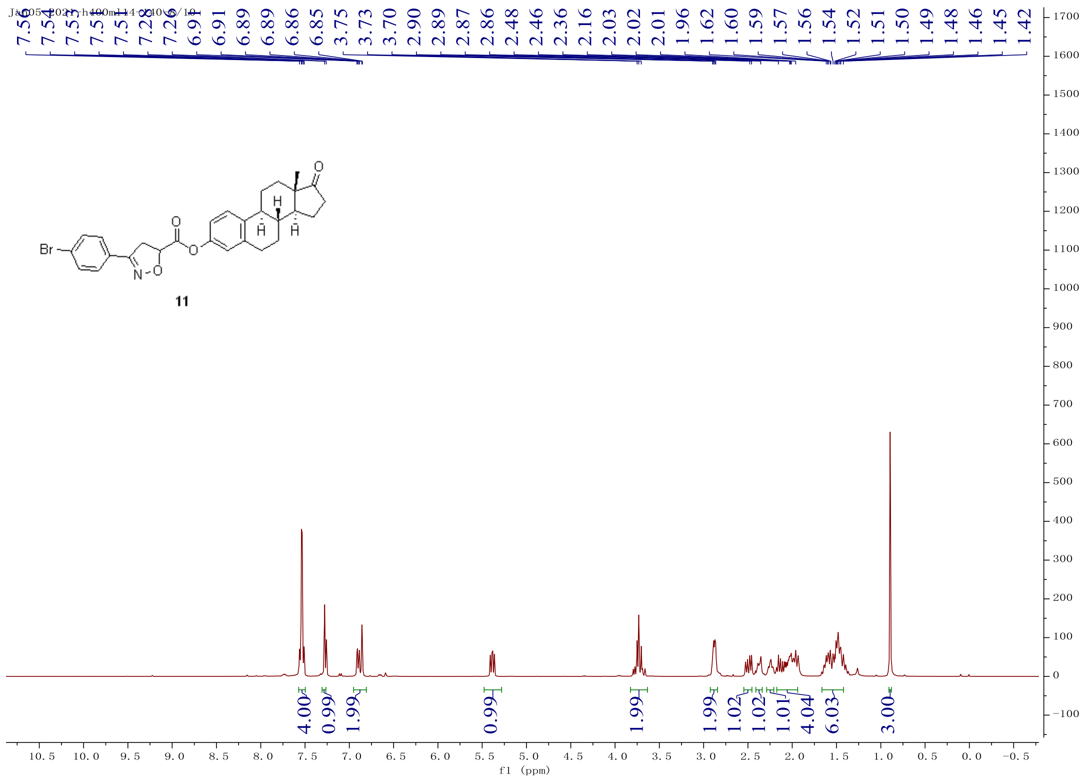
7.56
7.54
7.53
7.51
7.28
7.26
6.91
6.91
6.89
6.89
6.86
6.85
3.75
3.73
3.70
2.90
2.89
2.87
2.86
2.48
2.46
2.36
2.16
2.03
2.02
2.01
1.96
1.62
1.60
1.59
1.57
1.56
1.54
1.52
1.51
1.50
1.49
1.48
1.46
1.45
1.42
Br
N-O
O
H
11
4.00
0.99
1.99
0.99
1.99
1.99
1.02
1.02
1.01
4.04
6.03
3.00
f1 (ppm)

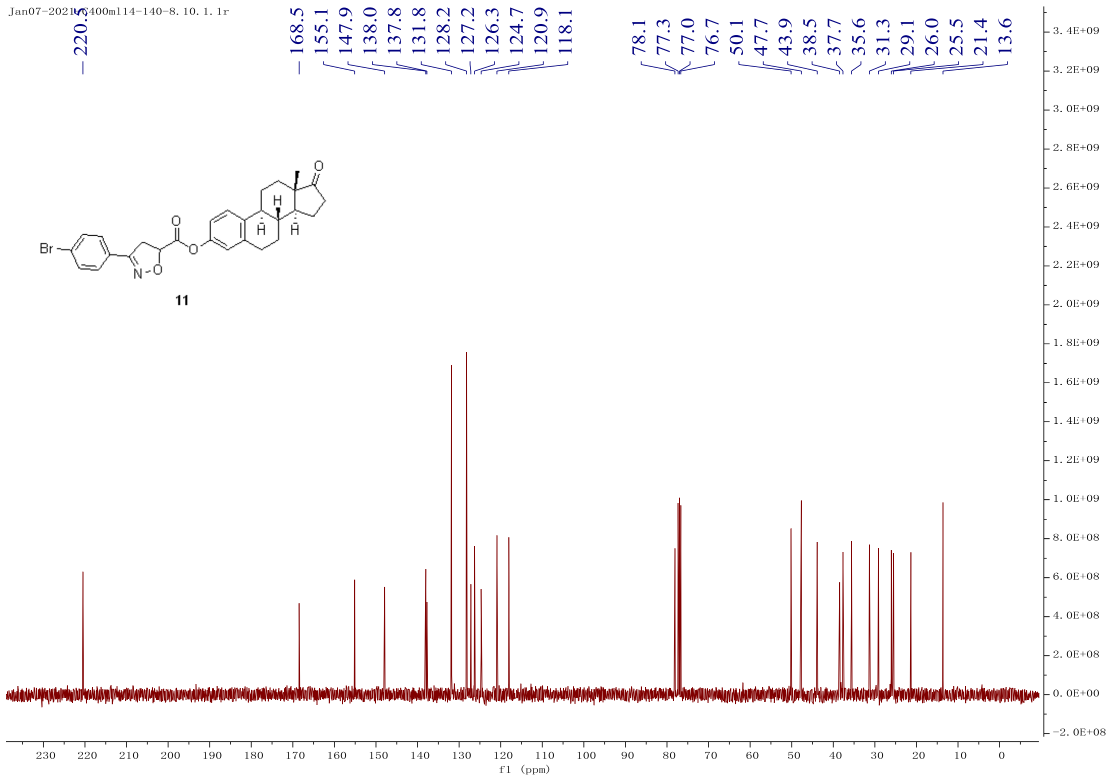
Jan07-2021-h400ml14-140-8.10.1.1r
220.5
168.5
155.1
147.9
138.0
137.8
131.8
128.2
127.2
126.3
124.7
120.9
118.1
78.1
77.3
77.0
76.7
50.1
47.7
43.9
38.5
37.7
35.6
31.3
29.1
26.0
25.5
21.4
13.6
Br
N-O
O
O
H
H
H
f1 (ppm)

11

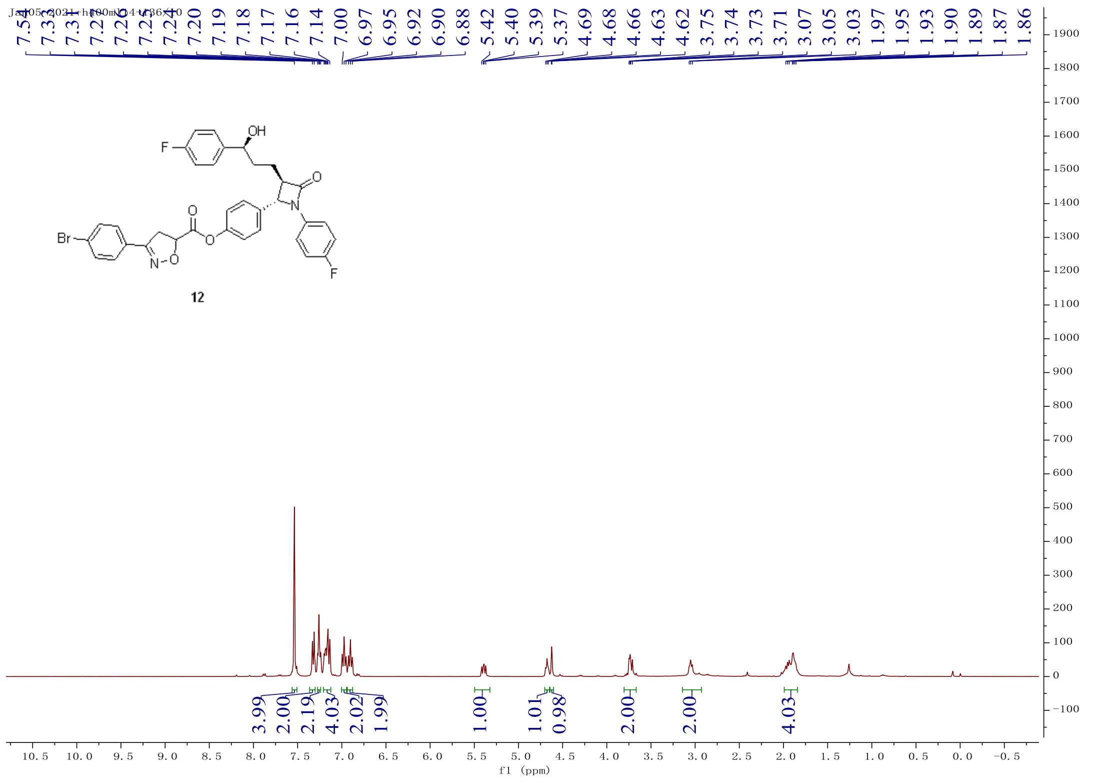
7.00
6.97
6.95
6.92
6.90
6.88
5.42
5.40
5.39
5.37
4.69
4.68
4.66
4.63
4.62
3.75
3.74
3.73
3.71
3.07
3.05
3.03
1.97
1.95
1.93
1.90
1.89
1.87
1.86
F
OH
O
N
Br
N-O
3.99
2.00
2.19
4.03
2.02
1.99
1.00
1.01
0.98
2.00
2.00
4.03
f1 (ppm)

12

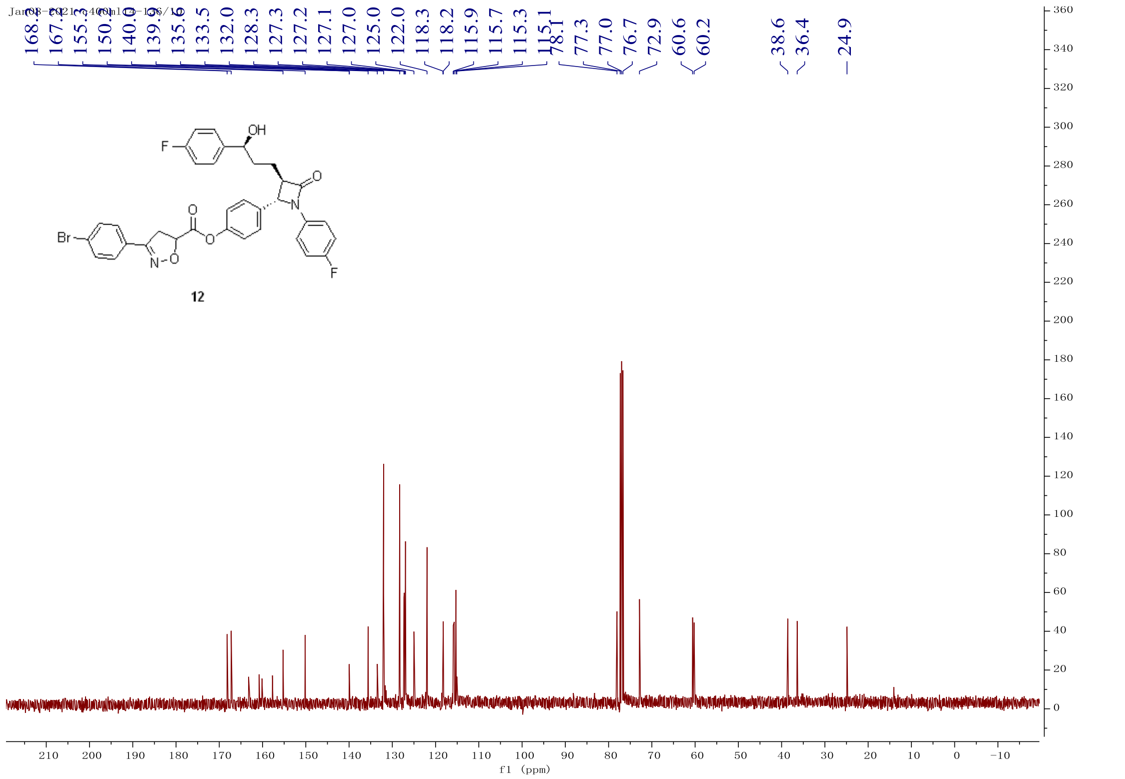
155.3
135.6
133.5
132.0
128.3
127.3
127.2
127.1
127.0
125.0
122.0
118.3
118.2
115.9
115.7
115.3
78.1
77.3
77.0
76.7
72.9
60.6
60.2
38.6
36.4
24.9
OH
F
O
N
Br
N-O
O
F
12
f1 (ppm)

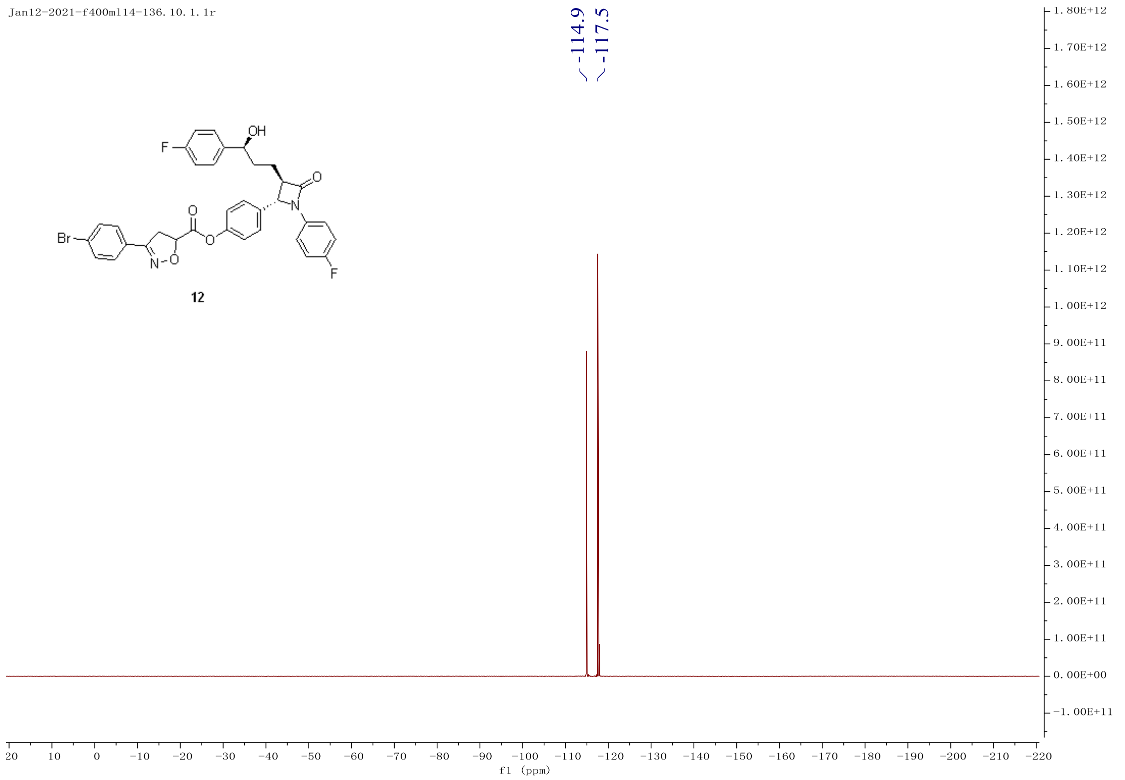
Jan12-2021-f400ml14-136.10.1.1r
-114.9
-117.5
OH
F
O
N
Br
N-O
O
F
12
f1 (ppm)

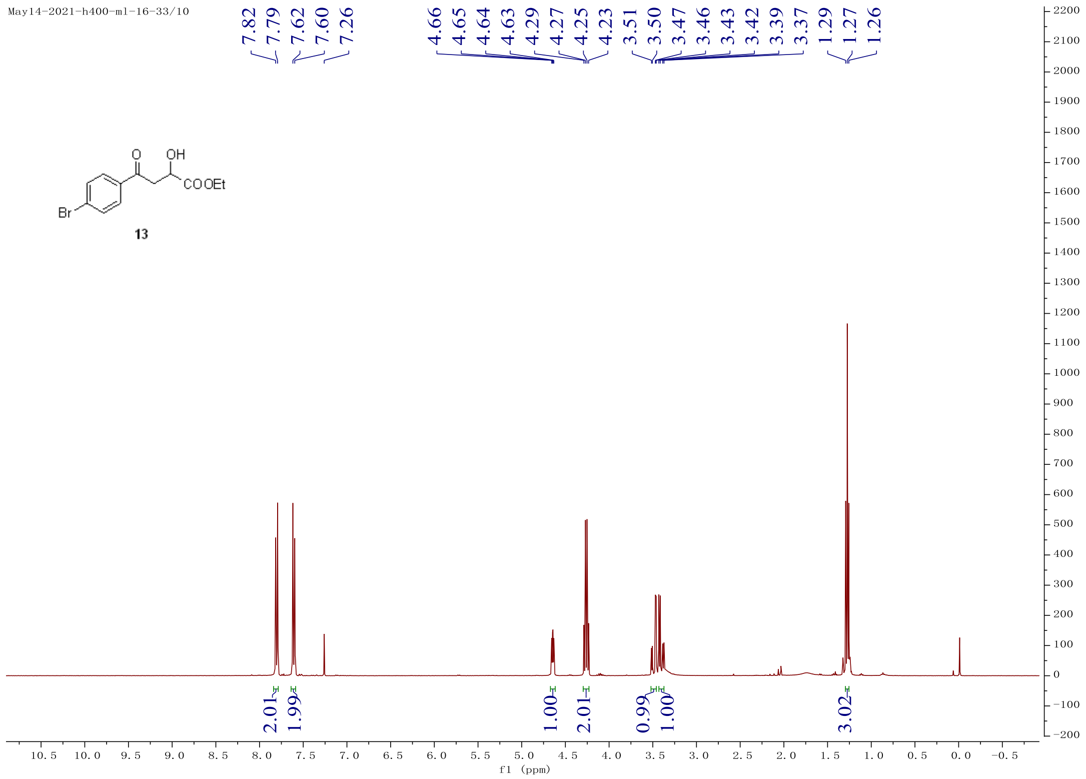
May14-2021-h400-ml-16-33/10
7.82 7.79 7.62 7.60 7.26 4.66 4.65 4.64 4.63 4.29 4.27 4.25 4.23 3.51 3.50 3.47 3.46 3.43 3.42 3.39 3.37 1.29 1.27 1.26
O OH
COOEt
Br
13
2.01 1.99 1.00 2.01 0.99 1.00 3.02
f1 (ppm)

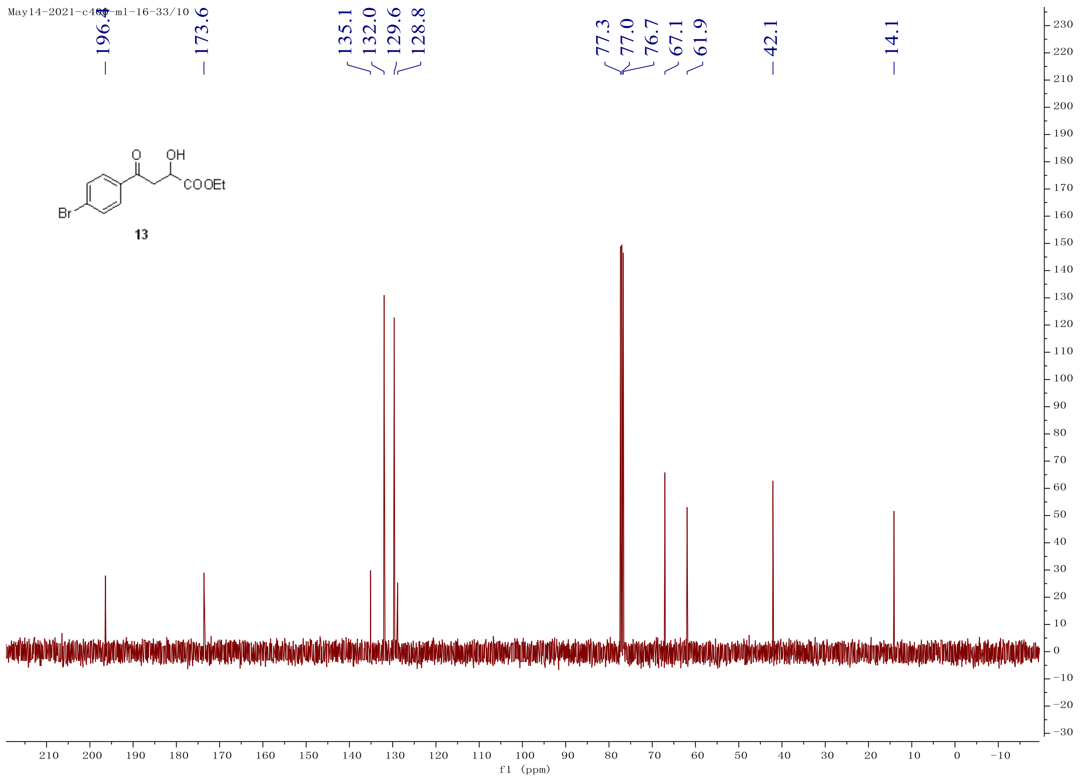
196.4 173.6 135.1 132.0 129.6 128.8 77.3 77.0 76.7 67.1 61.9 42.1 14.1
O OH
COOEt
Br
13
f1 (ppm)

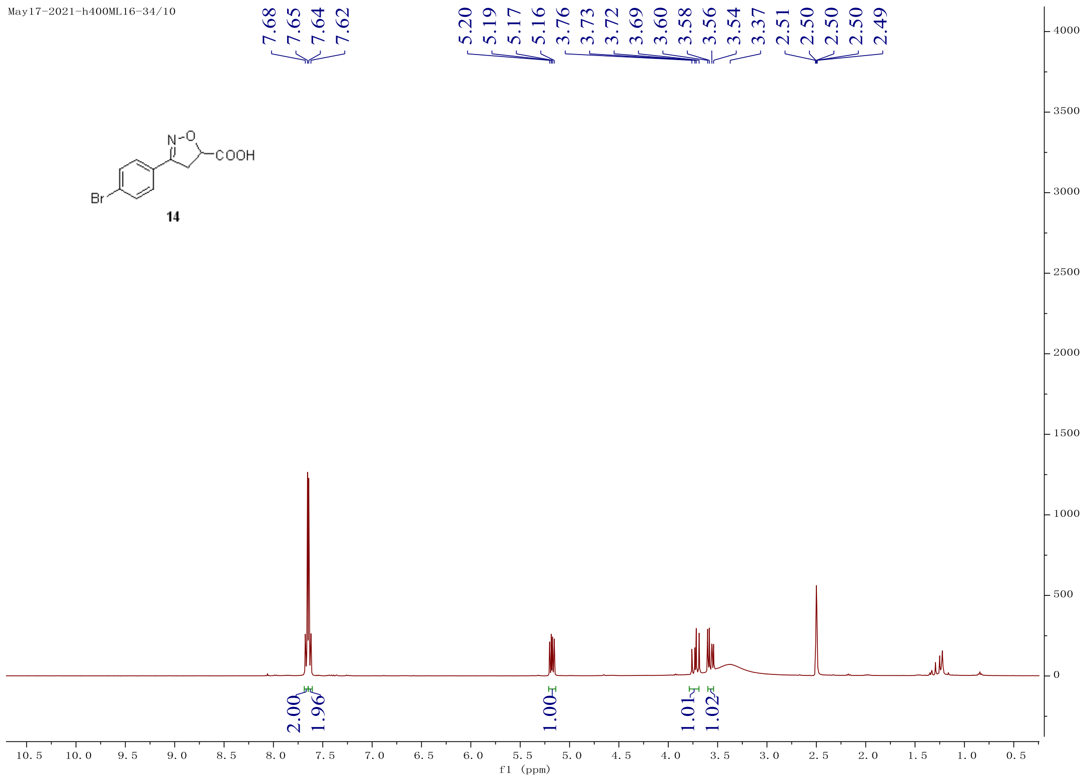
May17-2021-h400ML16-34/10
7.68 7.65 7.64 7.62 5.20 5.19 5.17 5.16 3.76 3.73 3.72 3.69 3.60 3.58 3.56 3.54 3.37 2.51 2.50 2.50 2.50 2.49
N-O
COOH
Br
14
2.00 1.96 1.00 1.01 1.02
f1 (ppm)

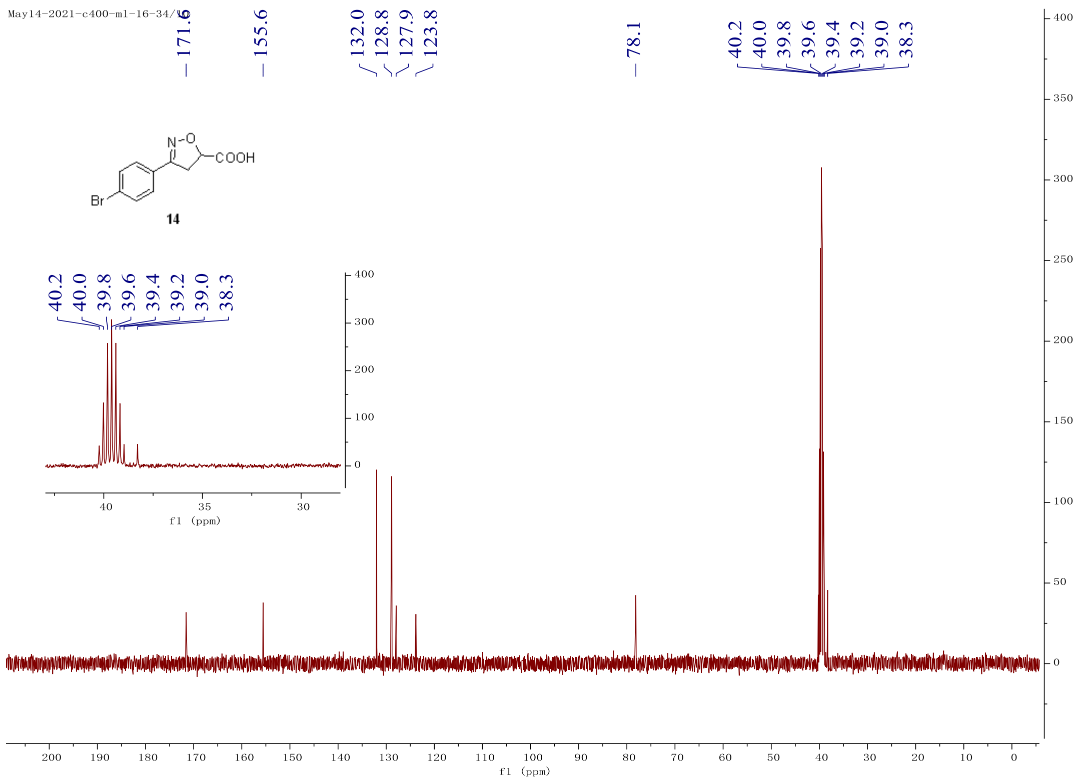
May14-2021-c400-ml-16-34/
171.6 155.6 132.0 128.8 127.9 123.8 78.1 40.2 40.0 39.8 39.6 39.4 39.2 39.0 38.3
N-O
COOH
Br
14
f1 (ppm)

Computational methods

The BP86 density functional method (DFT)[1] was employed to carry out all the calculations. The LANL2DZ[2] and 6-31G(d) basis sets[3] were used for Cu and the other atoms, respectively, in geometry optimizations. Vibrational frequency analyses at the same level of the theory were performed on all the optimized geometries to characterize them as local minima (no imaginary frequency) or transition states (one imaginary frequency). In addition, intrinsic reaction coordinate (IRC) calculations were used to verify that the transition state connect with appropriate reactant and product.[4] The Gibbs energies for all species were obtained at 298.15 K and 1 atm at their respective optimized structures. To consider solvation effects, the SMD solvation model[5] (solvent = tetrahydrofuran) was employed in single-point energy calculations at larger basis sets, SDD[6] for Cu and 6-311++G(d,p) for other atoms. The solution-phase Gibbs energy was determined by adding the solvation single-point energy and the gas-phase thermal correction to the Gibbs energy obtained from the vibrational frequencies. The translational entropy in solution was corrected using the method proposed by Whitesides *et al*.[7] Unless otherwise specified, the solution-phase Gibbs energy was used in the present discussions. All calculations were carried out with the Gaussian 09 suite of programs.[8]

References

[1] (a) Becke, A. D. *Phys. Rev. A* **1988**, *38*, 3098. (b) Perdew, J. P. *Phys. Rev. B* **1986**, *33*, 8822.

[2] Wadt, W. R.; Hay, P. J. *J. Chem. Phys.* **1985**, *82*, 284.

[3] Hariharan, P. C.; Pople, J. A. *Theor. Chim. Acta* **1973**, *28*, 213.

[4] Fukui, K. *Acc. Chem. Res.* **1981,** *14*, 363.

[5] Marenich, A.V.; Cramer, C. J.; Truhlar, D. G. *J Phys. Chem. B.* **2009**, *113*, 6378.

[6] Andrae, D.; Haeussermann, U.; Dolg, M.; Stoll, H.; Preuss, H. *Theor. Chim. Acta*. **19 90**, *77*, 123.

[7] Mammen, M.; Shakhnovich, E. I.; Deutch, J. M.; Whitesides, G. M. *J. Org. Chem.* **1998**, *63*, 3821.

[8] Frisch, M. J.; Trucks, G. W.; Schlegel, H. B.; Scuseria, G. E.; Robb, M. A.; Cheeseman, J. R.;

Scalmani, G.; Barone, V.; Mennucci, B.; Petersson, G. A.; Nakatsuji, H.; Caricato, M.; Li, X.; Hratchian, H. P.; Izmaylov, A. F.; Bloino, J.; Zheng, G.; Sonnenberg, J. L.; Hada, M.; Ehara, M.; Toyota, K.; Fukuda, R.; Hasegawa, J.; Ishida, M.; Nakajima, T.; Honda, Y.; Kitao, O.; Nakai, H.; Vreven, T.; Montgomery, J. A.; Peralta, J. E.; Ogliaro, F.; Bearpark, M.; Heyd, J. J.; Brothers, E.; Kudin, K. N.; Staroverov, V. N.; Kobayashi, R.; Normand, J.; Raghavachari, K.; Rendell, A.; Burant, J. C.; Iyengar, S. S.; Tomasi, J.; Cossi, M.; Rega, N.; Millam, N. J.; Klene, M.; Knox, J. E.; Cross, J. B.; Bakken, V.; Adamo, C.; Jaramillo, J.; Gomperts, R.; Stratmann, R. E.; Yazyev, O.; Austin, A. J.; Cammi, R.; Pomelli, C.; Ochterski, J. W.; Martin, R. L.; Morokuma, K.; Zakrzewski, V. G.; Voth, G. A.; Salvador, P.; Dannenberg, J. J.; Dapprich, S.; Daniels, A. D.; Farkas, O.; Foresman, J. B.; Ortiz, J. V.; Cioslowski, J.; Fox, D. J.; *Gaussian 09, Revision C.01*, Gaussian, Inc., Wallingford CT, **2010**.

Cartesian Coordinates and Energies

INT1

Center Number	Atomic Number	Atomic Type	Coordinates (Angstroms) X	Y	Z
1	6	0	-2.268066	-0.873651	0.000251
2	6	0	-0.937609	-1.308915	0.000064
3	6	0	0.127318	-0.374316	-0.000204
4	6	0	-0.185455	1.007637	-0.000278
5	6	0	-1.518936	1.432200	-0.000119
6	6	0	-2.569500	0.498308	0.000150
7	1	0	-3.075750	-1.614506	0.000460
8	1	0	-0.711749	-2.381954	0.000129
9	1	0	0.621097	1.750031	-0.000460
10	1	0	-1.739093	2.505857	-0.000182
11	1	0	-3.611055	0.836766	0.000287
12	6	0	1.497210	-0.878180	-0.000357
13	1	0	1.725340	-1.946688	-0.000926
14	7	0	2.539673	-0.086658	0.000062
15	7	0	3.449104	0.634087	0.000459

Zero-point correction= 0.111389 (Hartree/Particle)
Thermal correction to Energy= 0.118953
Thermal correction to Enthalpy= 0.119898
Thermal correction to Gibbs Free Energy= 0.079028
Sum of electronic and zero-point Energies= -379.694884
Sum of electronic and thermal Energies= -379.687319
Sum of electronic and thermal Enthalpies= -379.686375
Sum of electronic and thermal Free Energies= -379.727244
BP86/6-311++G(d,p)/SMD(THF)//BP86/6-31G(d) energy = -379.91348634

TBN

Center Number	Atomic Number	Atomic Type	Coordinates (Angstroms) X	Y	Z
1	6	0	0.768197	0.010437	0.000041
2	6	0	0.911224	0.853130	-1.274068
3	1	0	1.931300	1.272868	-1.329533
4	1	0	0.744059	0.233688	-2.173020
5	1	0	0.193589	1.691287	-1.284266
6	6	0	1.732651	-1.180413	-0.002590
7	1	0	2.773578	-0.812278	-0.001943
8	1	0	1.587220	-1.809227	0.893171
9	1	0	1.587000	-1.805560	-0.900877
10	6	0	0.911949	0.848181	1.277284
11	1	0	0.193672	1.685726	1.291347
12	1	0	0.746082	0.225146	2.173989
13	1	0	1.931753	1.268484	1.333412
14	8	0	-0.572338	-0.654439	-0.000771
15	7	0	-1.634959	0.304174	0.000332
16	8	0	-2.701120	-0.253981	-0.000305

Zero-point correction= 0.128308 (Hartree/Particle)
Thermal correction to Energy= 0.137021
Thermal correction to Enthalpy= 0.137965
Thermal correction to Gibbs Free Energy= 0.095841

Sum of electronic and zero-point Energies= -362.844215
Sum of electronic and thermal Energies= -362.835502
Sum of electronic and thermal Enthalpies= -362.834558
Sum of electronic and thermal Free Energies= -362.876682
BP86/6-311++G(d,p)/SMD(THF)//BP86/6-31G(d) energy = -363.08480634

TS1

Center Number	Atomic Number	Atomic Type	Coordinates (Angstroms) X	Y	Z
1	6	0	-0.425869	0.571053	-0.830956
2	1	0	0.166118	-0.003396	-1.551386
3	7	0	-0.395350	1.925531	-1.118581
4	7	0	-0.038559	2.942623	-0.668476
5	6	0	2.797543	-0.724233	0.087904
6	6	0	3.125755	-0.892793	1.585968
7	1	0	3.722700	-1.806329	1.762368
8	1	0	3.700869	-0.022310	1.945780
9	1	0	2.200616	-0.964666	2.183576
10	6	0	4.094012	-0.615706	-0.737780
11	1	0	4.691890	-1.540178	-0.641724
12	1	0	3.861618	-0.457541	-1.805522
13	1	0	4.702789	0.235161	-0.387379
14	6	0	1.940224	-1.904692	-0.418434
15	1	0	0.981086	-1.960400	0.125131
16	1	0	1.734734	-1.802327	-1.500685
17	1	0	2.469194	-2.863630	-0.268926
18	8	0	2.152583	0.550061	-0.140252
19	7	0	0.771555	0.647100	0.666998
20	8	0	0.694383	1.769426	1.142337
21	6	0	-1.735524	0.012121	-0.424809
22	6	0	-2.063194	-1.311772	-0.789919
23	6	0	-2.620535	0.745218	0.397384
24	6	0	-3.255772	-1.893742	-0.338731
25	1	0	-1.378052	-1.882816	-1.426882
26	6	0	-3.814161	0.161340	0.836813
27	1	0	-2.366185	1.767820	0.695639
28	6	0	-4.135461	-1.157913	0.471184
29	1	0	-3.501090	-2.921683	-0.627006
30	1	0	-4.496614	0.736822	1.471587
31	1	0	-5.071037	-1.610476	0.817421

Zero-point correction= 0.241527 (Hartree/Particle)
Thermal correction to Energy= 0.258063
Thermal correction to Enthalpy= 0.259007
Thermal correction to Gibbs Free Energy= 0.196594
Sum of electronic and zero-point Energies= -742.511007
Sum of electronic and thermal Energies= -742.494471
Sum of electronic and thermal Enthalpies= -742.493527
Sum of electronic and thermal Free Energies= -742.555940
BP86/6-311++G(d,p)/SMD(THF)//BP86/6-31G(d) energy = -742.97424823

N_2

Center Number	Atomic Number	Atomic Type	Coordinates (Angstroms) X	Y	Z
1	7	0	0.000000	0.000000	0.558483
2	7	0	0.000000	0.000000	-0.558483

Zero-point correction= 0.005364 (Hartree/Particle)
Thermal correction to Energy= 0.007724
Thermal correction to Enthalpy= 0.008668
Thermal correction to Gibbs Free Energy= -0.013106
Sum of electronic and zero-point Energies= -109.515585
Sum of electronic and thermal Energies= -109.513224
Sum of electronic and thermal Enthalpies= -109.512280
Sum of electronic and thermal Free Energies= -109.534054
BP86/6-311++G(d,p)/SMD(THF)//BP86/6-31G(d) energy = -109.55603207

INT2

Center Number	Atomic Number	Atomic Type	Coordinates (Angstroms) X	Y	Z
1	6	0	-4.135071	1.098889	0.064145
2	6	0	-2.769539	1.369755	-0.062007
3	6	0	-1.815883	0.317098	-0.101652
4	6	0	-2.282367	-1.020620	-0.006628
5	6	0	-3.652908	-1.279606	0.117038
6	6	0	-4.585668	-0.229565	0.154360
7	1	0	-4.851742	1.927456	0.091468
8	1	0	-2.422974	2.407890	-0.134664
9	1	0	-1.560314	-1.839130	-0.032682
10	1	0	-3.996349	-2.318081	0.187154
11	1	0	-5.655575	-0.443478	0.253897
12	6	0	-0.423085	0.700579	-0.235667
13	1	0	-0.142551	1.751121	-0.306809
14	6	0	3.048948	0.090938	0.101195
15	6	0	4.014342	1.270896	-0.110631
16	1	0	4.999777	1.025532	0.322827
17	1	0	4.150168	1.474681	-1.186665
18	1	0	3.633218	2.185566	0.374452
19	6	0	3.589249	-1.174017	-0.589722
20	1	0	4.579564	-1.433216	-0.172643
21	1	0	2.909065	-2.025993	-0.450869
22	1	0	3.707838	-0.991474	-1.671784
23	6	0	2.768211	-0.125102	1.595881
24	1	0	2.361599	0.793948	2.053040
25	1	0	2.055230	-0.950251	1.752729
26	1	0	3.708382	-0.380757	2.115794
27	8	0	1.844356	0.594634	-0.597657
28	7	0	0.602025	-0.139511	-0.308500
29	8	0	0.627284	-1.384973	-0.226545

Zero-point correction= 0.236062 (Hartree/Particle)
Thermal correction to Energy= 0.250442
Thermal correction to Enthalpy= 0.251386
Thermal correction to Gibbs Free Energy= 0.194477
Sum of electronic and zero-point Energies= -633.071250
Sum of electronic and thermal Energies= -633.056871
Sum of electronic and thermal Enthalpies= -633.055927
Sum of electronic and thermal Free Energies= -633.112836
BP86/6-311++G(d,p)/SMD(THF)//BP86/6-31G(d) energy = -633.49685259

H_2O

Center Number	Atomic Number	Atomic Type	Coordinates (Angstroms) X	Y	Z

1	8	0	0.000000	0.000000	0.121734
2	1	0	0.000000	0.764987	-0.486935
3	1	0	0.000000	-0.764987	-0.486935

Zero-point correction= 0.020584 (Hartree/Particle)
Thermal correction to Energy= 0.023418
Thermal correction to Enthalpy= 0.024363
Thermal correction to Gibbs Free Energy= 0.002888
Sum of electronic and zero-point Energies= -76.388614
Sum of electronic and thermal Energies= -76.385779
Sum of electronic and thermal Enthalpies= -76.384835
Sum of electronic and thermal Free Energies= -76.406309
BP86/6-311++G(d,p)/SMD(THF)//BP86/6-31G(d) energy = -76.46567358

TS2a

Center Number	Atomic Number	Atomic Type	Coordinates (Angstroms) X	Y	Z
1	6	0	-4.190167	1.195245	0.139087
2	6	0	-2.800580	1.260512	-0.026283
3	6	0	-2.040317	0.071102	-0.090968
4	6	0	-2.687949	-1.180627	0.011809
5	6	0	-4.077312	-1.234820	0.177354
6	6	0	-4.831723	-0.050083	0.242032
7	1	0	-4.773829	2.121240	0.185612
8	1	0	-2.290895	2.225990	-0.115401
9	1	0	-2.102396	-2.105054	-0.039617
10	1	0	-4.574636	-2.207920	0.255843
11	1	0	-5.918509	-0.098867	0.371110
12	6	0	-0.582865	0.197683	-0.266496
13	1	0	-0.036698	1.303823	-0.316283
14	6	0	3.152338	-0.091187	0.169344
15	6	0	4.259158	0.959157	-0.090562
16	1	0	5.136598	0.761722	0.552210
17	1	0	4.579463	0.920731	-1.145469
18	1	0	3.891780	1.978897	0.120833
19	6	0	3.678486	-1.504141	-0.142433
20	1	0	4.544291	-1.748232	0.499325
21	1	0	2.897126	-2.262843	0.024119
22	1	0	3.996743	-1.562679	-1.197440
23	6	0	2.654184	0.004594	1.626591
24	1	0	2.236921	1.005933	1.837087
25	1	0	1.869578	-0.746816	1.822405
26	1	0	3.481107	-0.172507	2.337333
27	8	0	2.090009	0.200888	-0.765321
28	7	0	0.201014	-0.781071	-0.378454
29	8	0	0.448210	-1.966547	-0.434308
30	8	0	0.925449	2.318142	-0.419499
31	1	0	1.052087	2.607835	0.511092
32	1	0	1.695314	1.261769	-0.597416

Zero-point correction= 0.252444 (Hartree/Particle)
Thermal correction to Energy= 0.269216
Thermal correction to Enthalpy= 0.270161
Thermal correction to Gibbs Free Energy= 0.207573
Sum of electronic and zero-point Energies= -709.430833
Sum of electronic and thermal Energies= -709.414061
Sum of electronic and thermal Enthalpies= -709.413116

Sum of electronic and thermal Free Energies= -709.475704
BP86/6-311++G(d,p)/SMD(THF)//BP86/6-31G(d) energy = -709.92148583

2a

Center Number	Atomic Number	Atomic Type	Coordinates (Angstroms) X	Y	Z
1	6	0	-2.213722	-1.392917	0.000142
2	1	0	-3.222581	-1.819656	0.000056
3	1	0	-1.371485	-2.091765	0.000650
4	6	0	-2.019498	-0.061369	-0.000265
5	1	0	-2.858470	0.643068	-0.000703
6	6	0	-0.690623	0.608582	-0.000058
7	8	0	-0.547041	1.827921	-0.000083
8	8	0	0.349142	-0.278163	0.000180
9	6	0	1.670454	0.331749	0.000464
10	1	0	1.762053	0.980433	-0.888978
11	1	0	1.762231	0.979217	0.890776
12	6	0	2.692572	-0.795052	-0.000397
13	1	0	2.581617	-1.430350	-0.895722
14	1	0	3.712958	-0.373293	-0.000174
15	1	0	2.581769	-1.431672	0.894009

Zero-point correction= 0.120355 (Hartree/Particle)
Thermal correction to Energy= 0.128508
Thermal correction to Enthalpy= 0.129452
Thermal correction to Gibbs Free Energy= 0.086801
Sum of electronic and zero-point Energies= -345.659255
Sum of electronic and thermal Energies= -345.651102
Sum of electronic and thermal Enthalpies= -345.650158
Sum of electronic and thermal Free Energies= -345.692809
BP86/6-311++G(d,p)/SMD(THF)//BP86/6-31G(d) energy = -345.89413613

TS2b

Center Number	Atomic Number	Atomic Type	Coordinates (Angstroms) X	Y	Z
1	6	0	-4.270743	-0.771768	1.169824
2	6	0	-2.922938	-0.483100	0.915947
3	6	0	-2.521041	-0.044147	-0.367083
4	6	0	-3.501300	0.085310	-1.379383
5	6	0	-4.844307	-0.218567	-1.122326
6	6	0	-5.235496	-0.647645	0.156231
7	1	0	-4.568364	-1.102785	2.171209
8	1	0	-2.173152	-0.581651	1.704818
9	1	0	-3.202847	0.431144	-2.376887
10	1	0	-5.587217	-0.111748	-1.920639
11	1	0	-6.285654	-0.882925	0.361552
12	6	0	-1.123248	0.285451	-0.735298
13	6	0	1.263616	2.604493	0.206825
14	6	0	2.479872	3.043121	-0.623170
15	1	0	2.829855	4.033317	-0.283014
16	1	0	3.307032	2.322245	-0.508084
17	1	0	2.220515	3.111356	-1.693621
18	6	0	1.632337	2.465267	1.693361
19	1	0	1.981181	3.438944	2.082608
20	1	0	0.770052	2.139531	2.296335
21	1	0	2.438069	1.724155	1.824487

22	6	0	0.077856	3.560822	0.002533
23	1	0	-0.177013	3.645686	-1.068656
24	1	0	-0.814619	3.211311	0.549597
25	1	0	0.331367	4.568302	0.377587
26	8	0	0.961916	1.287468	-0.391296
27	7	0	-0.211799	0.656561	0.216000
28	8	0	-0.014815	-0.051018	1.241914
29	6	0	-0.267243	-1.609004	-1.139734
30	1	0	-1.216389	-1.992545	-1.528367
31	6	0	0.190797	-2.075382	0.092867
32	1	0	-0.484738	-2.592074	0.779940
33	6	0	1.612760	-2.177875	0.489028
34	8	0	2.004814	-2.718053	1.520751
35	8	0	2.461241	-1.648417	-0.452182
36	6	0	3.866844	-1.725893	-0.107302
37	1	0	4.044224	-1.135882	0.810688
38	1	0	4.124051	-2.774824	0.125296
39	6	0	4.658041	-1.189224	-1.291847
40	1	0	4.378136	-0.144973	-1.513164
41	1	0	5.739095	-1.219975	-1.068205
42	1	0	4.474698	-1.794358	-2.196413
43	1	0	-0.998051	0.847730	-1.666147
44	1	0	0.472269	-1.306908	-1.889248

Zero-point correction= 0.357967 (Hartree/Particle)
Thermal correction to Energy= 0.380804
Thermal correction to Enthalpy= 0.381748
Thermal correction to Gibbs Free Energy= 0.304061
Sum of electronic and zero-point Energies= -978.707408
Sum of electronic and thermal Energies= -978.684572
Sum of electronic and thermal Enthalpies= -978.683627
Sum of electronic and thermal Free Energies= -978.761314
BP86/6-311++G(d,p)/SMD(THF)//BP86/6-31G(d) energy = -979.36530871

INT3

Center Number	Atomic Number	Atomic Type	Coordinates (Angstroms) X	Y	Z
1	6	0	-4.254683	-0.732443	1.302711
2	6	0	-2.950061	-0.331767	0.972522
3	6	0	-2.512753	-0.366746	-0.366323
4	6	0	-3.409254	-0.807645	-1.361605
5	6	0	-4.709557	-1.215017	-1.030069
6	6	0	-5.137283	-1.178416	0.306867
7	1	0	-4.581565	-0.694162	2.348271
8	1	0	-2.264101	0.014730	1.750626
9	1	0	-3.085036	-0.828026	-2.410148
10	1	0	-5.391822	-1.552377	-1.818727
11	1	0	-6.154396	-1.490889	0.569060
12	6	0	-1.104162	0.025633	-0.793668
13	6	0	1.031171	2.854913	-0.137161
14	6	0	-0.100772	3.839066	-0.470732
15	1	0	0.171225	4.863669	-0.159249
16	1	0	-0.303751	3.849692	-1.556610
17	1	0	-1.024229	3.548827	0.057590
18	6	0	2.285609	3.125902	-0.981115
19	1	0	2.670828	4.138754	-0.770325
20	1	0	3.073668	2.391800	-0.742523
21	1	0	2.054405	3.059315	-2.058836

22	6	0	1.367136	2.854110	1.362267
23	1	0	0.463210	2.684364	1.970075
24	1	0	2.091095	2.055375	1.589766
25	1	0	1.797456	3.831498	1.647681
26	8	0	0.649896	1.504261	-0.591080
27	7	0	-0.480120	0.996766	0.155728
28	8	0	0.049627	0.123779	1.196898
29	6	0	-0.099025	-1.158653	-0.828027
30	1	0	-0.581990	-2.119432	-1.061372
31	6	0	0.479954	-1.127726	0.608650
32	1	0	0.047714	-1.933797	1.230248
33	6	0	2.008416	-1.268874	0.648735
34	8	0	2.813344	-0.432085	1.020924
35	8	0	2.332970	-2.525848	0.216856
36	6	0	3.758791	-2.843186	0.198100
37	1	0	4.238663	-2.345309	1.056810
38	1	0	3.792939	-3.936010	0.336602
39	6	0	4.400872	-2.419426	-1.118688
40	1	0	4.371099	-1.323013	-1.229398
41	1	0	5.458184	-2.739336	-1.140274
42	1	0	3.883066	-2.880472	-1.977193
43	1	0	-1.145772	0.531635	-1.771157
44	1	0	0.686880	-0.963380	-1.574578

Zero-point correction= 0.362120 (Hartree/Particle)
Thermal correction to Energy= 0.384050
Thermal correction to Enthalpy= 0.384995
Thermal correction to Gibbs Free Energy= 0.309240
Sum of electronic and zero-point Energies= -978.751446
Sum of electronic and thermal Energies= -978.729515
Sum of electronic and thermal Enthalpies= -978.728571
Sum of electronic and thermal Free Energies= -978.804326
BP86/6-311++G(d,p)/SMD(THF)//BP86/6-31G(d) energy = -979.40578388

INT4

Center Number	Atomic Number	Atomic Type	Coordinates (Angstroms) X	Y	Z
1	6	0	2.019175	1.247957	-0.000003
2	6	0	0.620554	1.203238	0.000001
3	6	0	-0.048922	-0.048277	0.000001
4	6	0	0.714199	-1.243804	-0.000002
5	6	0	2.112413	-1.182170	0.000000
6	6	0	2.769738	0.059729	0.000000
7	1	0	2.526799	2.218758	-0.000002
8	1	0	0.032436	2.126248	0.000002
9	1	0	0.197653	-2.208646	-0.000003
10	1	0	2.692797	-2.111248	0.000001
11	1	0	3.864238	0.101841	0.000001
12	6	0	-1.474728	-0.103625	0.000004
13	7	0	-2.655552	-0.010775	0.000005
14	8	0	-3.874955	0.043772	-0.000005

Zero-point correction= 0.100127 (Hartree/Particle)
Thermal correction to Energy= 0.107749
Thermal correction to Enthalpy= 0.108693
Thermal correction to Gibbs Free Energy= 0.066435
Sum of electronic and zero-point Energies= -399.550099
Sum of electronic and thermal Energies= -399.542477

Sum of electronic and thermal Enthalpies= -399.541533
Sum of electronic and thermal Free Energies= -399.583791
BP86/6-311++G(d,p)/SMD(THF)//BP86/6-31G(d) energy = -399.76869019

^{t}BuOH…OH$_2$

Center Number	Atomic Number	Atomic Type	Coordinates (Angstroms) X	Y	Z
1	6	0	-0.717310	0.001088	0.000396
2	6	0	-0.919893	1.425268	0.559033
3	1	0	-1.779486	1.474524	1.252720
4	1	0	-0.021842	1.758438	1.112603
5	1	0	-1.096548	2.135142	-0.267941
6	6	0	-0.417985	-0.992931	1.143068
7	1	0	-1.250768	-1.044812	1.868425
8	1	0	-0.252000	-2.005186	0.733030
9	1	0	0.490882	-0.689152	1.695730
10	6	0	-1.948676	-0.447710	-0.801202
11	1	0	-2.135878	0.252053	-1.634294
12	1	0	-1.780884	-1.451910	-1.228481
13	1	0	-2.848538	-0.483303	-0.162192
14	8	0	0.371597	0.000065	-0.946447
15	1	0	3.456004	0.456596	-0.741522
16	8	0	3.027726	0.035844	0.032533
17	1	0	2.863367	-0.880844	-0.273318
18	1	0	1.184290	0.276897	-0.451222

Zero-point correction= 0.155669 (Hartree/Particle)
Thermal correction to Energy= 0.166192
Thermal correction to Enthalpy= 0.167136
Thermal correction to Gibbs Free Energy= 0.119716
Sum of electronic and zero-point Energies= -309.923981
Sum of electronic and thermal Energies= -309.913458
Sum of electronic and thermal Enthalpies= -309.912514
Sum of electronic and thermal Free Energies= -309.959934
BP86/6-311++G(d,p)/SMD(THF)//BP86/6-31G(d) energy = -310.21590971

TS3a

Center Number	Atomic Number	Atomic Type	Coordinates (Angstroms) X	Y	Z
1	6	0	4.364488	0.323488	-1.055889
2	6	0	3.088808	-0.233102	-1.186951
3	6	0	2.196186	-0.235096	-0.082268
4	6	0	2.617740	0.331694	1.143597
5	6	0	3.898600	0.886574	1.260542
6	6	0	4.776701	0.885530	0.165486
7	1	0	5.043175	0.318415	-1.915989
8	1	0	2.765058	-0.670958	-2.136444
9	1	0	1.937185	0.335575	1.999903
10	1	0	4.210935	1.322256	2.215842
11	1	0	5.777533	1.319925	0.261161
12	6	0	0.878511	-0.807719	-0.208746
13	7	0	0.173225	-1.372711	-1.036225
14	8	0	-0.908989	-1.886313	-1.294977
15	6	0	-0.607825	-0.773109	1.520706
16	1	0	0.076257	-1.352351	2.150716
17	6	0	-1.749764	-1.360726	1.030746

18	1	0	-1.941616	-2.429530	1.158178
19	6	0	-2.887096	-0.614279	0.440615
20	8	0	-3.994795	-1.099907	0.231866
21	8	0	-2.569611	0.693043	0.174960
22	6	0	-3.656380	1.477140	-0.387181
23	1	0	-3.993978	0.997018	-1.322999
24	1	0	-4.508926	1.462229	0.315537
25	6	0	-3.129077	2.885162	-0.621906
26	1	0	-2.278106	2.876988	-1.324318
27	1	0	-3.926363	3.516833	-1.051568
28	1	0	-2.794838	3.346623	0.323272
29	1	0	-0.537085	0.318035	1.572996

Zero-point correction= 0.221726 (Hartree/Particle)
Thermal correction to Energy= 0.237842
Thermal correction to Enthalpy= 0.238786
Thermal correction to Gibbs Free Energy= 0.174854
Sum of electronic and zero-point Energies= -745.197661
Sum of electronic and thermal Energies= -745.181545
Sum of electronic and thermal Enthalpies= -745.180601
Sum of electronic and thermal Free Energies= -745.244534
BP86/6-311++G(d,p)/SMD(THF)//BP86/6-31G(d) energy = -745.64800408

TS3b

Center Number	Atomic Number	Atomic Type	Coordinates (Angstroms) X	Y	Z
1	6	0	4.712971	-0.482031	-0.766285
2	6	0	3.330200	-0.588430	-0.972503
3	6	0	2.438986	-0.516956	0.119026
4	6	0	2.951150	-0.322429	1.416642
5	6	0	4.335984	-0.223380	1.621667
6	6	0	5.218664	-0.304072	0.532518
7	1	0	5.397183	-0.535074	-1.620813
8	1	0	2.933487	-0.723157	-1.985902
9	1	0	2.251780	-0.231345	2.255555
10	1	0	4.725980	-0.073101	2.634719
11	1	0	6.299572	-0.222170	0.693550
12	6	0	0.942780	-0.682937	-0.090467
13	6	0	-1.009663	2.661084	-0.256198
14	6	0	0.339677	3.143034	0.317108
15	1	0	0.444567	4.238609	0.213325
16	1	0	0.422847	2.886420	1.388033
17	1	0	1.173882	2.661123	-0.220720
18	6	0	-2.182255	3.316003	0.515656
19	1	0	-2.161223	4.415199	0.396541
20	1	0	-3.148287	2.941572	0.133949
21	1	0	-2.114919	3.081294	1.592723
22	6	0	-1.119080	2.977901	-1.758301
23	1	0	-0.290574	2.504101	-2.310232
24	1	0	-2.070552	2.589722	-2.162112
25	1	0	-1.080946	4.068228	-1.935279
26	8	0	-1.157032	1.243185	-0.085718
27	7	0	0.450483	0.177885	-1.108477
28	8	0	-0.342086	-0.497193	-1.952774
29	6	0	0.415731	-2.113851	-0.393249
30	1	0	1.211620	-2.734764	-0.840653
31	6	0	-0.698786	-1.848407	-1.400497
32	1	0	-0.706931	-2.507255	-2.281613

33	6	0	-2.152129	-1.734884	-0.911326
34	8	0	-3.096752	-1.740130	-1.684376
35	8	0	-2.256985	-1.615084	0.437972
36	6	0	-3.623620	-1.365759	0.908175
37	1	0	-3.954578	-0.409712	0.468577
38	1	0	-4.273884	-2.166769	0.517684
39	6	0	-3.600598	-1.325775	2.427585
40	1	0	-2.986483	-0.487342	2.799419
41	1	0	-4.628151	-1.180670	2.804525
42	1	0	-3.213214	-2.269481	2.848933
43	1	0	0.432952	-0.294981	0.932721
44	1	0	0.046680	-2.616644	0.513667
45	8	0	-0.375488	0.335720	2.019726
46	1	0	-0.980588	-0.424655	2.163478
47	1	0	-0.856929	0.899001	1.023305

Zero-point correction= 0.378766 (Hartree/Particle)
Thermal correction to Energy= 0.403051
Thermal correction to Enthalpy= 0.403996
Thermal correction to Gibbs Free Energy= 0.323057
Sum of electronic and zero-point Energies= -1055.114724
Sum of electronic and thermal Energies= -1055.090439
Sum of electronic and thermal Enthalpies= -1055.089495
Sum of electronic and thermal Free Energies= -1055.170433
BP86/6-311++G(d,p)/SMD(THF)//BP86/6-31G(d) energy = -1055.83391432

TS3c

Center Number	Atomic Number	Atomic Type	Coordinates (Angstroms) X	Y	Z
1	6	0	-4.507516	0.604028	-0.258669
2	6	0	-3.163949	0.573828	0.135950
3	6	0	-2.416019	-0.617512	0.023704
4	6	0	-3.029362	-1.763502	-0.517144
5	6	0	-4.375452	-1.726905	-0.917947
6	6	0	-5.120008	-0.545997	-0.786283
7	1	0	-5.079233	1.533721	-0.158859
8	1	0	-2.676940	1.469006	0.534457
9	1	0	-2.458862	-2.692080	-0.632735
10	1	0	-4.839525	-2.628039	-1.334881
11	1	0	-6.169910	-0.518341	-1.098604
12	6	0	-0.961901	-0.628376	0.429912
13	6	0	0.848288	2.622739	-0.389461
14	6	0	-0.192801	3.466726	-1.160422
15	1	0	0.217429	4.443993	-1.477503
16	1	0	-0.528677	2.923875	-2.062421
17	1	0	-1.076618	3.650752	-0.524395
18	6	0	2.100701	2.364498	-1.264983
19	1	0	2.565909	3.312489	-1.593939
20	1	0	2.844882	1.788712	-0.688238
21	1	0	1.821856	1.787723	-2.165776
22	6	0	1.272940	3.343223	0.917731
23	1	0	0.389129	3.517874	1.554780
24	1	0	1.986292	2.715107	1.476874
25	1	0	1.746642	4.315735	0.689267
26	8	0	0.289815	1.390778	0.003018
27	7	0	-0.682808	0.154114	1.590354
28	8	0	0.351827	-0.375618	2.253752
29	6	0	-0.192855	-1.950941	0.579478

30	1	0	-0.821575	-2.718389	1.067318
31	6	0	0.945418	-1.516018	1.498323
32	1	0	1.215399	-2.252613	2.273378
33	6	0	2.252215	-1.020187	0.847607
34	8	0	3.025727	-0.245175	1.382271
35	8	0	2.465927	-1.659060	-0.331875
36	6	0	3.740326	-1.354574	-0.983166
37	1	0	3.704807	-0.306637	-1.326790
38	1	0	4.543953	-1.438148	-0.231468
39	6	0	3.908924	-2.333125	-2.134486
40	1	0	3.086864	-2.235343	-2.863791
41	1	0	4.859140	-2.128001	-2.657803
42	1	0	3.933215	-3.374813	-1.771504
43	1	0	-0.385880	0.104745	-0.342302
44	1	0	0.181294	-2.344939	-0.375728

Zero-point correction= 0.355531 (Hartree/Particle)
Thermal correction to Energy= 0.377905
Thermal correction to Enthalpy= 0.378849
Thermal correction to Gibbs Free Energy= 0.301827
Sum of electronic and zero-point Energies= -978.700313
Sum of electronic and thermal Energies= -978.677939
Sum of electronic and thermal Enthalpies= -978.676995
Sum of electronic and thermal Free Energies= -978.754018
BP86/6-311++G(d,p)/SMD(THF)//BP86/6-31G(d) energy = -979.35504853

Product

Center Number	Atomic Number	Atomic Type	Coordinates (Angstroms) X	Y	Z
1	6	0	-1.732371	1.725870	-0.131055
2	8	0	-3.125197	1.329264	-0.449297
3	6	0	-0.905324	0.438507	-0.294415
4	1	0	-1.469464	2.531028	-0.830807
5	1	0	-1.717401	2.101453	0.906835
6	6	0	-3.310681	-0.028862	-0.296596
7	8	0	-4.401500	-0.564467	-0.395548
8	6	0	-1.966023	-0.578522	0.004891
9	7	0	-1.599161	-1.593040	0.720399
10	8	0	-0.246165	-1.432663	1.026332
11	6	0	0.166681	0.007066	0.715825
12	1	0	0.054053	0.549584	1.673242
13	6	0	1.603987	0.012026	0.264423
14	6	0	2.556842	0.758090	0.985473
15	6	0	2.015181	-0.715568	-0.873306
16	6	0	3.898046	0.792947	0.568678
17	1	0	2.243210	1.317508	1.874976
18	6	0	3.355996	-0.687732	-1.282885
19	1	0	1.286656	-1.311969	-1.434483
20	6	0	4.300340	0.069195	-0.565245
21	1	0	4.628336	1.381944	1.134889
22	1	0	3.665905	-1.259672	-2.164803
23	1	0	5.347090	0.090424	-0.888818
24	1	0	-0.557407	0.335817	-1.340453

Zero-point correction= 0.226406 (Hartree/Particle)
Thermal correction to Energy= 0.241359
Thermal correction to Enthalpy= 0.242303
Thermal correction to Gibbs Free Energy= 0.181301

Sum of electronic and zero-point Energies= -745.260795
Sum of electronic and thermal Energies= -745.245842
Sum of electronic and thermal Enthalpies= -745.244898
Sum of electronic and thermal Free Energies= -745.305900
BP86/6-311++G(d,p)/SMD(THF)//BP86/6-31G(d) energy = -745.71256103

$CuCl_2$

Center Number	Atomic Number	Atomic Type	Coordinates (Angstroms) X	Y	Z
1	29	0	0.000000	0.000000	0.000000
2	17	0	0.000000	0.000000	2.122888
3	17	0	0.000000	0.000000	-2.122888

Zero-point correction= 0.002034 (Hartree/Particle)
Thermal correction to Energy= 0.006637
Thermal correction to Enthalpy= 0.007581
Thermal correction to Gibbs Free Energy= -0.025199
Sum of electronic and zero-point Energies= -1116.741985
Sum of electronic and thermal Energies= -1116.737382
Sum of electronic and thermal Enthalpies= -1116.736438
Sum of electronic and thermal Free Energies= -1116.769218
BP86/6-311++G(d,p)-SDD/SMD(THF)//BP86/6-31G(d)-LANL2DZ energy = -1118.04648592

INT2’

Center Number	Atomic Number	Atomic Type	Coordinates (Angstroms) X	Y	Z
1	6	0	0.028641	-0.103443	1.187675
2	1	0	0.372801	0.732857	1.815200
3	7	0	0.361745	-1.291288	1.758928
4	7	0	0.707976	-2.298467	2.173337
5	6	0	-1.298184	0.008633	0.535247
6	6	0	-1.878780	1.297142	0.458028
7	6	0	-1.965718	-1.105494	-0.024438
8	6	0	-3.127341	1.458473	-0.152878
9	1	0	-1.334511	2.161405	0.853636
10	6	0	-3.217289	-0.930773	-0.623606
11	1	0	-1.494251	-2.093886	-0.014044
12	6	0	-3.802526	0.347408	-0.688485
13	1	0	-3.572271	2.456980	-0.214520
14	1	0	-3.733124	-1.794601	-1.055357
15	1	0	-4.778350	0.478184	-1.167926
16	29	0	1.442567	0.241852	-0.401581
17	17	0	1.523461	-1.723156	-1.351341
18	17	0	1.816797	2.331507	0.160385

Zero-point correction= 0.114507 (Hartree/Particle)
Thermal correction to Energy= 0.128011
Thermal correction to Enthalpy= 0.128955
Thermal correction to Gibbs Free Energy= 0.070341
Sum of electronic and zero-point Energies= -1496.460391
Sum of electronic and thermal Energies= -1496.446886
Sum of electronic and thermal Enthalpies= -1496.445942
Sum of electronic and thermal Free Energies= -1496.504556
BP86/6-311++G(d,p)-SDD/SMD(THF)//BP86/6-31G(d)-LANL2DZ energy = -1497.99007349

TS1’

Center Number	Atomic Number	Atomic Type	Coordinates (Angstroms) X	Y	Z
1	6	0	-0.044894	0.166779	1.016540
2	1	0	0.027722	0.838276	1.882524
3	7	0	0.299411	-1.393921	1.944721
4	7	0	0.826953	-2.388077	1.849914
5	6	0	-1.359708	0.032740	0.405466
6	6	0	-2.450494	0.792137	0.912668
7	6	0	-1.559368	-0.806576	-0.725468
8	6	0	-3.701266	0.724667	0.292077
9	1	0	-2.296142	1.441553	1.781377
10	6	0	-2.818687	-0.883274	-1.325433
11	1	0	-0.709778	-1.381080	-1.115178
12	6	0	-3.886496	-0.115666	-0.821872
13	1	0	-4.534969	1.322165	0.675273
14	1	0	-2.970029	-1.531142	-2.194845
15	1	0	-4.869131	-0.169288	-1.303467
16	29	0	1.475123	0.364712	-0.240285
17	17	0	2.049095	-1.616177	-0.987991
18	17	0	1.457652	2.552179	-0.061641

Zero-point correction= 0.112333 (Hartree/Particle)
Thermal correction to Energy= 0.125946
Thermal correction to Enthalpy= 0.126890
Thermal correction to Gibbs Free Energy= 0.067992
Sum of electronic and zero-point Energies= -1496.442187
Sum of electronic and thermal Energies= -1496.428574
Sum of electronic and thermal Enthalpies= -1496.427630
Sum of electronic and thermal Free Energies= -1496.486528
BP86/6-311++G(d,p)-SDD/SMD(THF)//BP86/6-31G(d)-LANL2DZ energy = -1497.96866462

INT3’

Center Number	Atomic Number	Atomic Type	Coordinates (Angstroms) X	Y	Z
1	6	0	0.152998	1.345615	0.781605
2	1	0	0.364139	1.460293	1.851220
3	6	0	-0.974488	0.531502	0.361098
4	6	0	-1.525674	-0.414864	1.299347
5	6	0	-1.522081	0.563818	-0.967027
6	6	0	-2.560671	-1.267889	0.919051
7	1	0	-1.130508	-0.441885	2.321459
8	6	0	-2.558834	-0.299139	-1.321530
9	1	0	-1.122862	1.285206	-1.686543
10	6	0	-3.084370	-1.217538	-0.389307
11	1	0	-2.969445	-1.976639	1.646812
12	1	0	-2.966774	-0.259047	-2.337068
13	1	0	-3.896269	-1.891329	-0.680986
14	29	0	1.020698	-0.363494	0.107815
15	17	0	2.559743	-1.761766	-0.348913
16	17	0	0.649679	2.756807	-0.141731

Zero-point correction= 0.105828 (Hartree/Particle)
Thermal correction to Energy= 0.116639
Thermal correction to Enthalpy= 0.117583
Thermal correction to Gibbs Free Energy= 0.065878
Sum of electronic and zero-point Energies= -1386.969643

Sum of electronic and thermal Energies= -1386.958832
Sum of electronic and thermal Enthalpies= -1386.957888
Sum of electronic and thermal Free Energies= -1387.009593
BP86/6-311++G(d,p)-SDD/SMD(THF)//BP86/6-31G(d)-LANL2DZ energy = -1388.45257080

TS2'

Center Number	Atomic Number	Atomic Type	Coordinates (Angstroms) X	Y	Z
1	6	0	-0.353292	-0.340189	0.235959
2	1	0	-0.551553	-0.985233	-0.622815
3	6	0	0.750678	0.610982	0.156438
4	6	0	1.261768	0.967010	-1.148618
5	6	0	1.224574	1.351445	1.289009
6	6	0	2.183491	2.034693	-1.282970
7	1	0	0.828586	0.505229	-2.044757
8	6	0	2.132344	2.397999	1.124661
9	1	0	0.852172	1.087945	2.283018
10	6	0	2.612264	2.748547	-0.157447
11	1	0	2.550770	2.296488	-2.280498
12	1	0	2.475315	2.952190	2.004429
13	1	0	3.324962	3.571621	-0.269570
14	29	0	2.420779	-0.624465	-0.319289
15	17	0	3.904433	-2.149513	-0.304523
16	17	0	-0.689277	-1.134973	1.752349
17	6	0	-3.988610	-0.396254	-0.454458
18	6	0	-4.610759	0.564334	-1.483730
19	1	0	-5.295166	0.021739	-2.160263
20	1	0	-5.180530	1.359396	-0.973329
21	1	0	-3.825093	1.040156	-2.096783
22	6	0	-5.065639	-1.015597	0.452642
23	1	0	-5.779308	-1.600488	-0.153923
24	1	0	-4.607749	-1.684980	1.200506
25	1	0	-5.623548	-0.227199	0.985672
26	6	0	-3.165067	-1.495783	-1.145166
27	1	0	-2.411868	-1.069175	-1.829788
28	1	0	-2.664618	-2.141800	-0.403447
29	1	0	-3.835698	-2.131610	-1.750058
30	8	0	-3.201375	0.400358	0.498136
31	7	0	-1.959305	0.981781	-0.068756
32	8	0	-1.710163	2.039127	0.467031

Zero-point correction= 0.235989 (Hartree/Particle)
Thermal correction to Energy= 0.256236
Thermal correction to Enthalpy= 0.257180
Thermal correction to Gibbs Free Energy= 0.183506
Sum of electronic and zero-point Energies= -1749.782408
Sum of electronic and thermal Energies= -1749.762161
Sum of electronic and thermal Enthalpies= -1749.761216
Sum of electronic and thermal Free Energies= -1749.834891
BP86/6-311++G(d,p)-SDD/SMD(THF)//BP86/6-31G(d)-LANL2DZ energy = -1751.51710849

INT4'

Center Number	Atomic Number	Atomic Type	Coordinates (Angstroms) X	Y	Z
1	6	0	-0.473253	-0.334642	-0.134841
2	1	0	-0.502563	-0.826894	-1.115255

3	6	0	0.681759	0.636502	-0.005904
4	6	0	1.338303	1.100646	-1.195229
5	6	0	0.988238	1.264251	1.238444
6	6	0	2.251122	2.187416	-1.118662
7	1	0	1.040013	0.707016	-2.174728
8	6	0	1.883633	2.337011	1.285381
9	1	0	0.498291	0.901453	2.145486
10	6	0	2.507450	2.807502	0.108475
11	1	0	2.742009	2.535041	-2.033052
12	1	0	2.102252	2.812411	2.246896
13	1	0	3.206547	3.648370	0.159228
14	29	0	2.481843	-0.469658	-0.294022
15	17	0	4.136814	-1.809574	-0.240723
16	17	0	-0.308196	-1.730705	1.104634
17	6	0	-4.169013	-0.143956	-0.335788
18	6	0	-4.490257	-0.551975	1.111912
19	1	0	-5.581986	-0.494613	1.272277
20	1	0	-4.168440	-1.591259	1.294988
21	1	0	-3.995013	0.106440	1.840125
22	6	0	-4.872942	-1.082712	-1.332162
23	1	0	-5.966062	-0.985602	-1.214415
24	1	0	-4.608382	-0.825208	-2.371823
25	1	0	-4.594255	-2.133909	-1.146948
26	6	0	-4.508410	1.325273	-0.638992
27	1	0	-3.993904	2.009368	0.052182
28	1	0	-4.224648	1.580149	-1.674939
29	1	0	-5.597464	1.476746	-0.534113
30	8	0	-2.750346	-0.431634	-0.654757
31	7	0	-1.745165	0.356638	0.029754
32	8	0	-2.002151	1.071253	1.028527

Zero-point correction= 0.238857 (Hartree/Particle)
Thermal correction to Energy= 0.259159
Thermal correction to Enthalpy= 0.260103
Thermal correction to Gibbs Free Energy= 0.186309
Sum of electronic and zero-point Energies= -1749.803407
Sum of electronic and thermal Energies= -1749.783105
Sum of electronic and thermal Enthalpies= -1749.782161
Sum of electronic and thermal Free Energies= -1749.855955
BP86/6-311++G(d,p)-SDD/SMD(THF)//BP86/6-31G(d)-LANL2DZ energy = -1751.53694330

TS3'

Center Number	Atomic Number	Atomic Type	Coordinates (Angstroms) X	Y	Z
1	6	0	-0.689207	0.422073	-0.758269
2	1	0	-1.008787	0.567958	-1.794374
3	6	0	0.531896	1.178777	-0.353863
4	6	0	1.136612	2.044656	-1.285380
5	6	0	1.140044	0.967990	0.923841
6	6	0	2.337946	2.705249	-0.967550
7	1	0	0.672209	2.193172	-2.266608
8	6	0	2.382956	1.594160	1.202069
9	1	0	0.591910	0.433489	1.707672
10	6	0	2.972688	2.465638	0.258127
11	1	0	2.792418	3.380839	-1.699321
12	1	0	2.849058	1.448882	2.181732
13	1	0	3.928459	2.946188	0.488957
14	29	0	2.285897	-0.642561	0.207090

15	17	0	3.973008	-1.981658	0.045570
16	17	0	-0.081465	-1.530068	-0.798969
17	6	0	-4.199029	-0.205516	0.096807
18	6	0	-3.875097	-1.493002	0.868698
19	1	0	-4.816870	-1.927080	1.249156
20	1	0	-3.397334	-2.232394	0.203918
21	1	0	-3.212311	-1.305995	1.726751
22	6	0	-5.150330	-0.494733	-1.077058
23	1	0	-6.119149	-0.842407	-0.679052
24	1	0	-5.326591	0.414765	-1.676266
25	1	0	-4.740777	-1.279158	-1.735436
26	6	0	-4.753780	0.918358	0.986272
27	1	0	-4.069761	1.154688	1.814124
28	1	0	-4.926170	1.831121	0.390146
29	1	0	-5.721791	0.598813	1.411587
30	8	0	-2.998879	0.273642	-0.655116
31	7	0	-1.811958	0.528121	0.091118
32	8	0	-1.784403	0.555357	1.340014

Zero-point correction= 0.238163 (Hartree/Particle)
Thermal correction to Energy= 0.258100
Thermal correction to Enthalpy= 0.259044
Thermal correction to Gibbs Free Energy= 0.185214
Sum of electronic and zero-point Energies= -1749.803525
Sum of electronic and thermal Energies= -1749.783588
Sum of electronic and thermal Enthalpies= -1749.782644
Sum of electronic and thermal Free Energies= -1749.856474
BP86/6-311++G(d,p)-SDD/SMD(THF)//BP86/6-31G(d)-LANL2DZ energy = -1751.53421180

INT2’’

Center Number	Atomic Number	Atomic Type	Coordinates (Angstroms) X	Y	Z
1	6	0	-1.200459	-0.902128	1.091917
2	1	0	-0.796759	-0.665776	2.089403
3	7	0	-1.734515	-2.152328	1.096338
4	7	0	-2.176119	-3.211082	1.055828
5	6	0	-2.010492	0.165271	0.415947
6	6	0	-1.909336	1.489161	0.899637
7	6	0	-2.838518	-0.103287	-0.696914
8	6	0	-2.629451	2.520975	0.281820
9	1	0	-1.254328	1.704823	1.750805
10	6	0	-3.561027	0.934330	-1.301396
11	1	0	-2.912904	-1.121690	-1.095275
12	6	0	-3.461145	2.249684	-0.817231
13	1	0	-2.542579	3.542785	0.667812
14	1	0	-4.201170	0.711590	-2.161968
15	1	0	-4.025015	3.057571	-1.295384
16	29	0	0.468384	-0.979815	-0.050174
17	17	0	1.820177	-1.812424	-1.552721
18	6	0	2.837831	0.481274	1.293617

19	6	0	3.831566	1.428650	0.603908
20	6	0	3.103239	1.763015	-0.713702
21	6	0	1.652787	1.869096	-0.242463
22	1	0	3.024400	-0.572761	1.018601
23	1	0	2.821501	0.581104	2.393694
24	1	0	3.986376	2.342987	1.206067
25	1	0	4.812532	0.950652	0.444534
26	1	0	3.465391	2.691168	-1.187808
27	1	0	3.197677	0.926694	-1.428524
28	1	0	1.433033	2.856758	0.211443
29	1	0	0.907658	1.658729	-1.027113
30	8	0	1.505314	0.841106	0.788626

Zero-point correction= 0.228550 (Hartree/Particle)
Thermal correction to Energy= 0.246827
Thermal correction to Enthalpy= 0.247771
Thermal correction to Gibbs Free Energy= 0.176585
Sum of electronic and zero-point Energies= -1268.564841
Sum of electronic and thermal Energies= -1268.546564
Sum of electronic and thermal Enthalpies= -1268.545620
Sum of electronic and thermal Free Energies= -1268.616806
BP86/6-311++G(d,p)/SDD/SMD// BP86/6-31G(d)/LANL2DZ energy in THF= -1270.23377483

TS1''

Center Number	Atomic Number	Atomic Type	Coordinates (Angstroms) X	Y	Z
1	6	0	-1.169989	0.604962	-0.992442
2	1	0	-1.182117	0.553933	-2.096223
3	7	0	-1.751239	2.293728	-0.923353
4	7	0	-1.901832	3.316573	-0.466230
5	6	0	-2.264086	-0.187208	-0.383601
6	6	0	-3.138666	-0.960699	-1.187180
7	6	0	-2.396469	-0.254093	1.027697
8	6	0	-4.094020	-1.799586	-0.598705
9	1	0	-3.051227	-0.907314	-2.279086
10	6	0	-3.371121	-1.069611	1.612548
11	1	0	-1.716842	0.341007	1.650547
12	6	0	-4.217289	-1.848460	0.800910
13	1	0	-4.752663	-2.406067	-1.230037
14	1	0	-3.466322	-1.110063	2.703010
15	1	0	-4.972960	-2.494955	1.260858
16	29	0	0.518660	0.723628	-0.109113

17	17	0	1.890180	1.735613	1.325509
18	6	0	3.048990	-0.534403	-1.311234
19	6	0	4.179111	-1.426425	-0.790860
20	6	0	3.762965	-1.617304	0.681542
21	6	0	2.239189	-1.751760	0.577663
22	1	0	3.226479	0.527194	-1.054217
23	1	0	2.852366	-0.631947	-2.392742
24	1	0	4.193662	-2.394186	-1.325616
25	1	0	5.168800	-0.951863	-0.902226
26	1	0	4.231055	-2.495109	1.158658
27	1	0	4.012228	-0.715191	1.266676
28	1	0	1.914299	-2.797706	0.417613
29	1	0	1.713801	-1.325517	1.448436
30	8	0	1.844382	-0.985932	-0.610747

Zero-point correction= 0.226767 (Hartree/Particle)
Thermal correction to Energy= 0.244965
Thermal correction to Enthalpy= 0.245909
Thermal correction to Gibbs Free Energy= 0.175239
Sum of electronic and zero-point Energies= -1268.548222
Sum of electronic and thermal Energies= -1268.530024
Sum of electronic and thermal Enthalpies= -1268.529080
Sum of electronic and thermal Free Energies= -1268.599750
BP86/6-311++G(d,p)/SDD/SMD// BP86/6-31G(d)/LANL2DZ energy in THF= -1270.21563018

INT3''

Center Number	Atomic Number	Atomic Type	Coordinates (Angstroms) X	Y	Z
1	6	0	-1.306584	-0.477338	1.030387
2	1	0	-1.454917	-0.923044	2.036456
3	6	0	-2.562646	-0.067363	0.466504
4	6	0	-3.784347	-0.159595	1.205097
5	6	0	-2.623795	0.464457	-0.859688
6	6	0	-4.993930	0.269601	0.655202
7	1	0	-3.753259	-0.571752	2.220782
8	6	0	-3.834962	0.878921	-1.413528
9	1	0	-1.690926	0.522195	-1.432361
10	6	0	-5.020149	0.785799	-0.655340
11	1	0	-5.921084	0.198681	1.234229
12	1	0	-3.868452	1.275326	-2.433987
13	1	0	-5.970775	1.114004	-1.090923
14	29	0	0.386101	-0.547886	0.276117

15	17	0	1.581702	-1.936329	-1.025599
16	6	0	3.139958	0.311796	1.258849
17	6	0	4.383081	0.983684	0.671069
18	6	0	4.008203	1.071008	-0.822403
19	6	0	2.514427	1.413644	-0.770836
20	1	0	3.165563	-0.784136	1.111614
21	1	0	2.948788	0.547764	2.319262
22	1	0	4.526035	1.991748	1.102206
23	1	0	5.298141	0.394838	0.852194
24	1	0	4.592491	1.825457	-1.376042
25	1	0	4.144181	0.088353	-1.306154
26	1	0	2.328211	2.503607	-0.736033
27	1	0	1.943039	0.958902	-1.597034
28	8	0	2.013948	0.852082	0.491463

Zero-point correction= 0.219884 (Hartree/Particle)

Thermal correction to Energy= 0.235514

Thermal correction to Enthalpy= 0.236458

Thermal correction to Gibbs Free Energy= 0.172978

Sum of electronic and zero-point Energies= -1159.054055

Sum of electronic and thermal Energies= -1159.038425

Sum of electronic and thermal Enthalpies= -1159.037481

Sum of electronic and thermal Free Energies= -1159.100960

BP86/6-311++G(d,p)/SDD/SMD// BP86/6-31G(d)/LANL2DZ energy in THF= -1160.68575557

TS2''

Center Number	Atomic Number	Atomic Type	Coordinates (Angstroms) X	Y	Z
1	6	0	0.323888	0.834780	0.534359
2	1	0	0.732708	0.739487	1.555480
3	6	0	-0.075077	2.202974	0.225686
4	6	0	0.034644	3.242027	1.191183
5	6	0	-0.647069	2.516522	-1.038703
6	6	0	-0.441241	4.529012	0.918844
7	1	0	0.487669	3.016418	2.164279
8	6	0	-1.098143	3.809045	-1.320564
9	1	0	-0.720170	1.719183	-1.788514
10	6	0	-1.002326	4.814718	-0.339742
11	1	0	-0.366716	5.315730	1.677678
12	1	0	-1.530590	4.038003	-2.300514
13	1	0	-1.364210	5.825890	-0.558012
14	6	0	3.387971	-1.308393	0.472201

15	6	0	4.891630	-1.432794	0.775720
16	1	0	5.087037	-2.400404	1.270305
17	1	0	5.482388	-1.393956	-0.154847
18	1	0	5.231252	-0.622022	1.443184
19	6	0	2.905552	-2.423743	-0.463407
20	1	0	2.969887	-3.396696	0.056386
21	1	0	1.857512	-2.270344	-0.776635
22	1	0	3.536416	-2.471638	-1.367208
23	6	0	2.574020	-1.236832	1.767180
24	1	0	2.816930	-0.329059	2.347367
25	1	0	1.490499	-1.273381	1.570015
26	1	0	2.830694	-2.113153	2.388160
27	8	0	3.357414	-0.041655	-0.324426
28	7	0	2.271579	0.874541	-0.270794
29	8	0	2.454384	1.772958	-1.043303
30	29	0	-0.394133	-0.677641	-0.334318
31	17	0	-0.694162	-2.270630	-1.836750
32	6	0	-3.254619	-0.368925	1.312254
33	6	0	-4.363498	-0.847708	0.341887
34	6	0	-3.949613	-2.310474	-0.003835
35	6	0	-2.759581	-2.584521	0.931184
36	1	0	-2.868754	0.641796	1.103085
37	1	0	-3.601874	-0.407719	2.366260
38	1	0	-5.356799	-0.791968	0.819821
39	1	0	-4.395623	-0.224650	-0.567570
40	1	0	-4.770373	-3.032710	0.145049
41	1	0	-3.603697	-2.378680	-1.048058
42	1	0	-3.083576	-2.995654	1.911148
43	1	0	-1.987715	-3.228031	0.482924
44	8	0	-2.149617	-1.287562	1.149010

Zero-point correction= 0.349926 (Hartree/Particle)

Thermal correction to Energy= 0.375306

Thermal correction to Enthalpy= 0.376250

Thermal correction to Gibbs Free Energy= 0.288117

Sum of electronic and zero-point Energies= -1521.880432

Sum of electronic and thermal Energies= -1521.855052

Sum of electronic and thermal Enthalpies= -1521.854108

Sum of electronic and thermal Free Energies= -1521.942241

BP86/6-311++G(d,p)/SDD/SMD// BP86/6-31G(d)/LANL2DZ energy in THF= -1523.75735092

INT4’’

Center Atomic Atomic Coordinates (Angstroms)

Number	Number	Type	X	Y	Z
1	6	0	1.098696	1.010078	0.970193
2	1	0	1.196004	0.607380	1.977450
3	6	0	-0.113772	1.713041	0.565863
4	6	0	-1.050778	2.065943	1.595243
5	6	0	-0.398726	2.137985	-0.784323
6	6	0	-2.173751	2.839364	1.311884
7	1	0	-0.851560	1.737184	2.621357
8	6	0	-1.557979	2.918342	-1.039870
9	1	0	0.353390	1.994789	-1.563585
10	6	0	-2.432634	3.273024	-0.010854
11	1	0	-2.857143	3.115161	2.122649
12	1	0	-1.749197	3.245693	-2.067049
13	1	0	-3.316237	3.883660	-0.224425
14	6	0	4.146571	-0.661456	0.206457
15	6	0	4.895641	-1.344880	1.364479
16	1	0	5.639097	-2.051536	0.956307
17	1	0	5.427657	-0.600175	1.981123
18	1	0	4.197982	-1.903558	2.011374
19	6	0	5.127368	0.129508	-0.676603
20	1	0	5.890477	-0.556134	-1.087999
21	1	0	4.608651	0.623924	-1.509830
22	1	0	5.643785	0.898235	-0.075743
23	6	0	3.314191	-1.678955	-0.586206
24	1	0	2.609894	-2.208630	0.078935
25	1	0	2.745430	-1.197178	-1.397070
26	1	0	3.986388	-2.429378	-1.038749
27	8	0	3.266899	0.265351	0.969350
28	7	0	2.178989	0.863589	0.206413
29	8	0	2.360181	1.199765	-0.980108
30	29	0	-1.042778	0.086085	-0.552293
31	17	0	-1.357701	-1.187214	-2.335271
32	6	0	-3.152474	-1.005258	1.416213
33	6	0	-3.839918	-1.947477	0.424693
34	6	0	-2.868986	-3.157842	0.370514
35	6	0	-1.527179	-2.590719	0.901058
36	1	0	-3.389708	0.063708	1.291834
37	1	0	-3.343705	-1.299025	2.469925
38	1	0	-4.859968	-2.225277	0.740727
39	1	0	-3.888464	-1.465948	-0.566652
40	1	0	-3.220590	-3.988558	1.006952
41	1	0	-2.760306	-3.525634	-0.661555
42	1	0	-1.229083	-3.052519	1.862363

43	1	0	-0.705328	-2.670944	0.173847
44	8	0	-1.734148	-1.159912	1.135327

Zero-point correction= 0.353439 (Hartree/Particle)
Thermal correction to Energy= 0.378497
Thermal correction to Enthalpy= 0.379441
Thermal correction to Gibbs Free Energy= 0.294509
Sum of electronic and zero-point Energies= -1521.936974
Sum of electronic and thermal Energies= -1521.911917
Sum of electronic and thermal Enthalpies= -1521.910973
Sum of electronic and thermal Free Energies= -1521.995905
BP86/6-311++G(d,p)/SDD/SMD// BP86/6-31G(d)/LANL2DZ energy in THF= -1523.81299417

CuCl-THF

Center Number	Atomic Number	Atomic Type	Coordinates (Angstroms) X	Y	Z
1	29	0	1.062162	-0.001963	-0.009536
2	17	0	3.171927	0.001822	0.015212
3	6	0	-1.697164	1.201188	-0.133880
4	6	0	-3.106164	0.736034	0.248252
5	6	0	-3.112635	-0.733542	-0.222892
6	6	0	-1.698807	-1.198251	0.136432
7	1	0	-1.638189	1.563160	-1.176489
8	1	0	-1.271103	1.960740	0.541153
9	1	0	-3.249435	0.792684	1.342063
10	1	0	-3.887405	1.346274	-0.233916
11	1	0	-3.886866	-1.341249	0.273603
12	1	0	-3.273200	-0.791626	-1.314231
13	1	0	-1.617875	-1.529866	1.188070
14	1	0	-1.292048	-1.979350	-0.525538
15	8	0	-0.865090	-0.003423	-0.030532

Zero-point correction= 0.116792 (Hartree/Particle)
Thermal correction to Energy= 0.124761
Thermal correction to Enthalpy= 0.125705
Thermal correction to Gibbs Free Energy= 0.081864
Sum of electronic and zero-point Energies= -888.870907
Sum of electronic and thermal Energies= -888.862938
Sum of electronic and thermal Enthalpies= -888.861993
Sum of electronic and thermal Free Energies= -888.905835
BP86/6-311++G(d,p)/SDD/SMD// BP86/6-31G(d)/LANL2DZ energy in THF= -890.32376157

TS2c

Center Number	Atomic Number	Atomic Type	Coordinates (Angstroms) X	Y	Z
1	6	0	-1.774504	2.989479	0.927072
2	6	0	-0.771589	2.014605	0.874066
3	6	0	-0.635364	1.197103	-0.273337
4	6	0	-1.544536	1.371726	-1.345995
5	6	0	-2.555815	2.337768	-1.278429
6	6	0	-2.671693	3.154656	-0.141472
7	1	0	-1.864323	3.619292	1.819098
8	1	0	-0.080144	1.877615	1.708458
9	1	0	-1.446741	0.743867	-2.239676
10	1	0	-3.246453	2.461542	-2.120185
11	1	0	-3.459329	3.914467	-0.087664
12	6	0	0.402913	0.171061	-0.439257
13	6	0	3.846241	-0.203408	-0.559775
14	6	0	4.596321	-1.411826	-1.141985
15	1	0	5.631855	-1.125212	-1.395303
16	1	0	4.631937	-2.236775	-0.410093
17	1	0	4.100152	-1.776947	-2.057581
18	6	0	4.506945	0.278870	0.742649
19	1	0	5.545376	0.597446	0.539328
20	1	0	3.960975	1.129384	1.180838
21	1	0	4.528017	-0.533453	1.488355
22	6	0	3.730632	0.929609	-1.591543
23	1	0	3.246318	0.568634	-2.516061
24	1	0	3.138782	1.770060	-1.190547
25	1	0	4.732641	1.312932	-1.854129
26	8	0	2.516068	-0.781834	-0.293865
27	7	0	1.576652	0.200178	0.255536
28	8	0	1.559733	0.270451	1.538634
29	6	0	-0.305359	-1.609662	0.853023
30	6	0	0.437001	-1.340820	2.010705
31	1	0	-0.076433	-0.884390	2.861913
32	1	0	0.517102	-0.251430	-1.441852
33	1	0	0.058550	-2.337506	0.120872
34	6	0	-1.760693	-1.340642	0.857419
35	8	0	-2.381426	-0.728352	1.722638
36	8	0	-2.355215	-1.871182	-0.263173
37	6	0	-3.790587	-1.659993	-0.342466
38	1	0	-3.986182	-0.573916	-0.395538
39	1	0	-4.260489	-2.035440	0.583969

40	6	0	-4.291907	-2.395279	-1.577473
41	1	0	-5.383448	-2.261109	-1.677911
42	1	0	-3.811078	-2.009017	-2.492807
43	1	0	-4.081255	-3.476268	-1.507129
44	1	0	1.346294	-1.907178	2.221797

Zero-point correction= 0.357998 (Hartree/Particle)

Thermal correction to Energy= 0.380720

Thermal correction to Enthalpy= 0.381664

Thermal correction to Gibbs Free Energy= 0.304704

Sum of electronic and zero-point Energies= -978.707651

Sum of electronic and thermal Energies= -978.684929

Sum of electronic and thermal Enthalpies= -978.683985

Sum of electronic and thermal Free Energies= -978.760944

BP86/6-311++G(d,p)/SMD//BP86/6-31G(d) energy in THF= -979.36339460

INT3-a

Center Number	Atomic Number	Atomic Type	Coordinates (Angstroms) X	Y	Z
1	6	0	-1.597906	3.203061	0.836924
2	6	0	-0.707340	2.119209	0.830306
3	6	0	-0.721363	1.193722	-0.233099
4	6	0	-1.645464	1.378548	-1.281901
5	6	0	-2.542978	2.457195	-1.268845
6	6	0	-2.520494	3.374983	-0.207291
7	1	0	-1.570699	3.917722	1.667399
8	1	0	0.013335	1.991183	1.643181
9	1	0	-1.658512	0.668591	-2.117789
10	1	0	-3.251278	2.585783	-2.095406
11	1	0	-3.215426	4.222311	-0.196293
12	6	0	0.211858	-0.007114	-0.297328
13	6	0	3.779008	-0.271419	-0.571141
14	6	0	4.474579	-1.549014	-1.066041
15	1	0	5.525966	-1.332432	-1.323752
16	1	0	4.460523	-2.328902	-0.285078
17	1	0	3.970501	-1.945591	-1.964540
18	6	0	4.430039	0.248685	0.720677
19	1	0	5.484766	0.518308	0.530636
20	1	0	3.904049	1.141937	1.094075
21	1	0	4.401058	-0.523278	1.508302
22	6	0	3.750353	0.809146	-1.664159
23	1	0	3.290311	0.415107	-2.587757

24	1	0	3.166897	1.680586	-1.324369
25	1	0	4.774779	1.145901	-1.904317
26	8	0	2.411860	-0.746749	-0.308209
27	7	0	1.575238	0.334466	0.206781
28	8	0	1.458052	0.139572	1.624742
29	6	0	-0.202319	-1.248264	0.588197
30	6	0	0.617050	-1.031831	1.871146
31	1	0	-0.024368	-0.788803	2.732928
32	1	0	0.329309	-0.317171	-1.345934
33	1	0	0.129550	-2.153597	0.054449
34	6	0	-1.702422	-1.352630	0.829982
35	8	0	-2.286102	-1.077049	1.868723
36	8	0	-2.323679	-1.799892	-0.298987
37	6	0	-3.778141	-1.881750	-0.209127
38	1	0	-4.172548	-0.865991	-0.031182
39	1	0	-4.042945	-2.502014	0.664805
40	6	0	-4.282042	-2.477261	-1.514864
41	1	0	-5.383159	-2.554022	-1.489105
42	1	0	-4.000258	-1.845541	-2.374432
43	1	0	-3.868860	-3.487663	-1.675756
44	1	0	1.254780	-1.902334	2.101699

Zero-point correction= 0.362058 (Hartree/Particle)
Thermal correction to Energy= 0.384187
Thermal correction to Enthalpy= 0.385131
Thermal correction to Gibbs Free Energy= 0.308993
Sum of electronic and zero-point Energies= -978.751490
Sum of electronic and thermal Energies= -978.729361
Sum of electronic and thermal Enthalpies= -978.728417
Sum of electronic and thermal Free Energies= -978.804555
BP86/6-311++G(d,p)/SMD//BP86/6-31G(d) energy in THF= -979.40706179
